# Identification of new driver and passenger mutations within APOBEC-induced hotspot mutations in bladder cancer

**DOI:** 10.1186/s13073-020-00781-y

**Published:** 2020-09-28

**Authors:** Ming-Jun Shi, Xiang-Yu Meng, Jacqueline Fontugne, Chun-Long Chen, François Radvanyi, Isabelle Bernard-Pierrot

**Affiliations:** 1 Department of Urology, Beijing Friendship Hospital, Capital Medical University, Beijing, China; 2 Institut Curie, CNRS, UMR144, Molecular Oncology team, PSL Research University, 26 Rue d’Ulm, 75005 Paris, France; 3 Paris-Saclay University, Paris, France; 4 Department of Urology, Zhongnan Hospital of Wuhan University, Wuhan, China; 5 Institut Curie, CNRS, UMR3244, PSL Research University, Paris, France; 6 Sorbonne Université, Paris, France

**Keywords:** Bladder cancer, APOBEC, Mutagenesis, Stem-loop, Driver mutation, Passenger mutation, Oncogene, Tumour suppressor gene, Aryl hydrocarbon receptor

## Abstract

**Background:**

APOBEC-driven mutagenesis and functional positive selection of mutated genes may synergistically drive the higher frequency of some hotspot driver mutations compared to other mutations within the same gene, as we reported for *FGFR3* S249C. Only a few APOBEC-associated driver hotspot mutations have been identified in bladder cancer (BCa). Here, we systematically looked for and characterised APOBEC-associated hotspots in BCa.

**Methods:**

We analysed 602 published exome-sequenced BCas, for part of which gene expression data were also available. APOBEC-associated hotspots were identified by motif-mapping, mutation signature fitting and APOBEC-mediated mutagenesis comparison. Joint analysis of DNA hairpin stability and gene expression was performed to predict driver or passenger hotspots. Aryl hydrocarbon receptor (AhR) activity was calculated based on its target genes expression. Effects of AhR knockout/inhibition on BCa cell viability were analysed.

**Results:**

We established a panel of 44 APOBEC-associated hotspot mutations in BCa, which accounted for about half of the hotspot mutations. Fourteen of them overlapped with the hotspots found in other cancer types with high APOBEC activity. They mostly occurred in the DNA lagging-strand templates and the loop of DNA hairpins. APOBEC-associated hotspots presented systematically a higher prevalence than the other mutations within each APOBEC-target gene, independently of their functional impact. A combined analysis of DNA loop stability and gene expression allowed to distinguish known passenger from known driver hotspot mutations in BCa, including loss-of-function mutations affecting tumour suppressor genes, and to predict new candidate drivers, such as *AHR* Q383H. We further characterised *AHR* Q383H as an activating driver mutation associated with high AhR activity in luminal tumours. High AhR activity was also found in tumours presenting amplifications of *AHR* and its co-receptor *ARNT*. We finally showed that BCa cells presenting those different genetic alterations were sensitive to AhR inhibition.

**Conclusions:**

Our study identified novel potential drivers within APOBEC-associated hotspot mutations in BCa reinforcing the importance of APOBEC mutagenesis in BCa. It could allow a better understanding of BCa biology and aetiology and have clinical implications such as AhR as a potential therapeutic target. Our results also challenge the dogma that all hotspot mutations are drivers and mostly gain-of-function mutations affecting oncogenes.

## Background

Bladder cancer (BCa) shows one of the highest overall mutation loads across various cancer types [1]. Identifying driver mutations within these mutations could help further our understanding of bladder cancer biology and provide new therapeutic targets. The frequency of driver hotspot mutations results from two factors: the mutation rate at a given position and the functional advantage the mutation provides to tumour cells, leading to clonal expansion. Mutation rate is partially impacted by endo-/exogenous mutagenic processes, which can leave characteristic fingerprints on the cancer genome in a DNA sequence context-dependent manner, such as apolipoprotein B mRNA-editing enzyme, catalytic polypeptide-like (APOBEC)-mediated mutagenesis, related to APOBEC deaminase activity [2, 3]. A pan-cancer analysis has revealed that APOBEC-mediated mutagenesis significantly contributes to the overall mutations in several cancer types, in particular in BCa, which exhibits the second highest abundance of APOBEC-induced mutations after cervical cancer [2]. Not surprisingly, APOBEC could contribute to the emergence of driver hotspot mutations in BCa. In line with this hypothesis, we recently showed that compared to other *FGFR3* recurrent mutations, the higher prevalence of *FGFR3* S249C mutation (one of the most common mutations in bladder cancer) was likely due to an increased mutation rate induced by APOBEC, rather than selection related to an increased tumorigenicity of this mutation [4–6]. Studies conducted in another cancer type (head and neck) [7, 8] or at a pan-cancer scale [9–11] have recently proposed lists of hotspot mutations putatively associated with APOBEC. In the most recent study, Buisson et al. [9] showed that a subset of hotspot mutations were passenger mutations, occurring through the preference of *APOBEC3A* for DNA hairpin loops. Though we and others have previously studied certain APOBEC-associated coding and non-coding hotspot mutations in BCa specifically [4–6, 12–14], a systematic investigation of APOBEC-associated hotspot mutations and their oncogenic driver effects in BCa have not been reported thus far.

We found here that APOBEC is a major source of hotspot mutations and identified a panel of 44 APOBEC-associated hotspot mutations in BCa, 14 of which overlapped with those found in other tumour types with high APOBEC activity. In the genes with APOBEC hotspot mutations, we report a consistently higher prevalence of the APOBEC-associated mutation compared to the other mutations within the same gene. Additionally, APOBEC-associated mutations preferentially occurred on the lagging-strand template of DNA replication and in the loop of DNA hairpins. We confirmed that the APOBEC-associated hotspot mutations included not only driver (activating as well as loss of function mutations) but also passenger hotspot mutations. Furthermore, we proposed a model to predict the classification of APOBEC-associated hotspot mutations as passenger or driver, based on a combined analysis of the stability of DNA hairpin structures and of the gene expression level. We then characterised one of the predicted driver mutations targeting the aryl hydrocarbon receptor, *AHR* Q383H, predicted as an activating mutation. We determined that this mutation, as well as amplifications of *AHR* and *ARNT* (the nuclear translocator of AhR, dimerizing with AhR to regulate gene transcription), were associated with high *AHR* mRNA expression and activity. We finally showed that BCa cells presenting those genetic alterations affecting the AhR pathway were dependent on AhR for their viability, suggesting AhR as a potential therapeutic target for these tumours.

## Methods

### SNV data

All available tumour datasets for single nucleotide variants (SNVs) from whole-exome sequencing (WES) were downloaded from cBioPortal for Cancer Genomics [15, 16] for 602 BCa [17–21], 281 cervical cancer [22], 648 head and neck cancer [22–25], 1575 breast cancer [22, 26–28] and 1247 lung cancer [22, 29, 30] samples. Duplicated samples from time series or multiple-position sampling from the same subject were removed.

### Identification of hotspot mutations

We ranked the frequency of mutations for all SNVs in the BCa tumours (*n* = 602). The mutation frequencies followed a long-tail distribution. An empirical threshold was determined to distinguish the ‘head’ from the ‘tail’ within the distribution. This threshold was defined as the smallest integer for which the ratio of the number of mutations with a frequency larger than this integer to the number of mutations with a frequency equal to this integer was > 1. We visualised the ratios across mutation frequencies and for the determined threshold. Mutations with a frequency that was equal or above this threshold (≥ 4) were considered as hotspot mutations. We repeated this analysis for the dataset combining other cancer types presenting a relatively high APOBEC-mediated mutagenesis (cervical, head and neck, breast and lung cancer) (*n* = 3751) [2, 3, 31–34] and identified the same threshold (*n* = 4).

### Mutational signature fitting

We first conducted non-negative matrix factorisation (NMF) by fitting the SNVs obtained for the primary BCa dataset (*n* = 602), the other APOBEC-related cancer types [2, 3, 31–34] (*n* = 3744), to the 30 established Catalogue Of Somatic Mutations In Cancer (COSMIC) signatures.

Then, for each patient, we calculated the arithmetic sum of the fraction scores for COSMIC signatures 2 and 13, which have been demonstrated to be associated with APOBEC activity [3]. This sum was defined as the parameter to evaluate APOBEC-mediated mutagenesis. R version 3.5.2 and Bioconductor package *sigfit*version 1.3.1 were used for these analyses and for the associated visualisation.

### Association between the APOBEC signature and mutations

To identify the APOBEC-associated hotspot mutations in BCa (*n* = 602) and other APOBEC-related cancer types [2, 3, 31–34] (*n* = 3744), we compared the fraction score of APOBEC-mediated mutagenesis in tumours bearing one of the given hotspot mutations corresponding to an APOBEC-type motif (TCN → T [G/T] N mutations, N = any base) with tumours free of any these hotspot mutations. Recently, Letouzé and colleagues have also developed a method which estimates the probability of each mutation being due to each mutational process without initial stringent restriction to certain motifs [35]. We therefore applied this alternative method in BCa to double check for the association between hotspot mutations and various mutational signatures, including APOBEC mutagenesis.

### Clonality of APOBEC-associated hotspot mutations

The data on mutation event clonality was extracted from TCGA BCa WES dataset [17] which was evaluated by using ABSOLUTE algorithm [36]. We compared the probability of being a clonal event between the 44 APOBEC-associated hotspot mutations and all other mutations occurring within the 33 APOBEC-target genes, using Fisher’s exact test. Considering that mutation frequency may also be associated with clonality, we further conducted multivariate analysis using generalised linear mixed model (GLMM) taking mutation frequency as covariate and genes as random effects.

### Replication fork directionality (RFD) profiling and stem-loop structures for ssDNA

We performed analysis of published genome-wide replication fork directionality (RFD) data in nine human cancer/normal cell lines—HeLa, IMR90, TLSE19, K562, TF1, GM06990, BL79, IARC385 and Raji cells [37, 38]—to identify the strand that would be favoured as the lagging-strand template. Considering APOBEC enzymes specifically deaminated ‘C’ to ‘U’, we expected the complementary strand to be the lagging-strand template if mutations were of the NGA → N [C/A] A type. Data availability and interpretation have been described elsewhere [4, 37, 38]. RFD profiles were determined by mapping Okazaki fragments to C (Crick) and W (Watson) DNA strands. Positive (negative) RFD values indicate the regions in which Watson (Crick) strands are replicated mostly as lagging-strand templates. We simply assigned a value of ‘1’ (or ‘− 1’) to mutations occurring on Watson (or Crick) strands replicated mostly as lagging-strand templates for each cell line (Additional file 1: Table S1, Additional file 2: Table S2). For each hotspot mutation in BCa and APOBEC-associated hotspots in other cancer types, we calculated the probability of locating in lagging-strand template across all the nine cell lines. We then compared the probabilities of APOBEC-associated hotspot mutations either against the ones that were not associated to APOBEC or against random, i.e. 0.5, using Wilcoxon test or one-side Wilcoxon signed-rank test (‘greater’ hypothesis). Figures were visualised with Integrative Genomics Viewer (IGV) software.

The Mfold tool with the default parameters for DNA folding [39] was used to evaluate secondary structures of single-stranded DNA (ssDNA) for all hotspot mutations in BCa and APOBEC-associated hotspots in other cancer types, with 25-nucleotide (nt) sequences centred on the mutation sites as input. A thermodynamic parameter [40]—free energy (*Δ*G) —widely used to evaluate the stabilities of stem-loop structures, was calculated, as summarised in Additional file 1: Table S1, and in Additional file 2: Table S2). We rationalised 25 nt as the appropriate sequence length for stem-loop structure prediction as well as *Δ*G calculation. In particular, we found that with the sequence length increase (starting from 13 nt, 4 nt increase per escalation, centred around the mutation site), the completion of the primary stem-loop structure harbouring the mutation site always occurred before or at 25 nt length. Furthermore, the formation of neighbouring/secondary stem-loop structure not associated with the mutation site always occurred after 25 nt, considering all the 6 APOBEC-associated passenger mutations in BCa (except for *CAMK2G* I132I mutation which was not located in loops of any sequence length we tested) probably locating on a stem-loop structure when ssDNA formed (Additional file 3: Fig. S1a-b). We then predicted the stem-loop structure formation as well as the *Δ*G value for both APOBEC-associated and non APOBEC-associated hotspot mutations, and tested whether the probability that hotspot mutations were located in a loop was significantly higher for APOBEC-associated ones than those not APOBEC-associated or than random, i.e. 0.5, using Fisher’s exact test or logit transformation and z test.

### Gene expression analysis

The RNA-seq transcriptome (RSEM) of more than 10,000 samples involving 32 cancer types from the TCGA project was downloaded from the cBioPortal database. We compared the APOBEC family (*AIDA, APOBEC1, APOBEC2, APOBEC3A, APOBEC3B, APOBEC3C, APOBEC3D, APOBEC3F, APOBEC3G, APOBEC3H,* and *APOBEC4*) gene expression levels between those with any APOBEC-associated mutations and those without in BCa (*n* = 406). Comparisons were also made between BCa tumours bearing a given APOBEC-associated hotspot mutation of a known/suspected tumour suppressor gene (TSG) and BCa tumours devoid of any mutation (wild-type) of this gene, in terms of its expression level. Wilcoxon rank sum test was applied for comparisons.

A recent comprehensive pan-cancer study has functionally annotated 299 cancer genes and 579 driver mutations [41]. Association between gene functional importance (known oncogenes/TSGs vs. genes of unknown function, which were annotated with aforementioned reference) and their expression level were analysed in BCa tumours (*n* = 406). In brief, the expression value of each gene (a total of > 20,000 genes) in a given sample was first divided by that of the housekeeping gene *GAPDH*, and the genes were then ranked on a percentile scale according to the median relative expression (normalised against *GAPDH*) level across BCa tumours (*n* = 406). We then compared this parameter between known oncogenes/TSGs and genes of unknown function, using Wilcoxon rank sum test. Further, to verify the universality of known oncogenes/TSGs being generally highly expressed, we investigated all well-annotated 299 cancer gene [41] in pan-cancer types. For each cancer type, the expression of all genes was rank-transformed as described above, and finally a gene expression rank by cancer type matrix was obtained. We then extracted the rank of 299 cancer genes accordingly (Additional file 4: Table S3). For cancer genes annotated as PANCAN [41], the median of its expression rank across all cancer types was used. We compared the distribution of rank of cancer genes and all genes (the background) using Wilcoxon rank sum test.

### Stem-loop stability

For the standardised evaluation of mutation sites in terms of ssDNA structure-related APOBEC mutagen accessibility, we calculated the normalised loop stability score for the 44 APOBEC-associated hotspot mutations in BCa, as shown below:

$$ \mathrm{Normalised}\ \mathrm{loop}\ \mathrm{stability}\ \mathrm{score}=\left\{\begin{array}{c}\frac{\Delta \mathrm{G}-\min \left(\boldsymbol{\Delta} \mathbf{G}\right)}{\max \left(\boldsymbol{\Delta} \mathbf{G}\right)-\min \left(\boldsymbol{\Delta} \mathbf{G}\right)},\mathrm{when}\ \mathrm{mutation}\ \mathrm{site}\ \mathrm{in}\ \mathrm{loop}\\ {}\ 1,\mathrm{when}\ \mathrm{mutation}\ \mathrm{site}\ \mathrm{not}\ \mathrm{in}\ \mathrm{loop}\end{array}\right. $$

in which *Δ*G denotes the exact *Δ*G value of the loop structure in which a given mutation is located, and ***Δ*G** denotes the vector containing all *Δ*G values. Lower values are associated with easier formation of more stable loops, with greater accessibility for APOBEC mutagens. Loops with *Δ*G value ≥ 0 were considered same as not in loops. We compared normalised loop stability score (*Δ*G) between APOBEC-associated known driver and passenger hotspot mutations. Additionally, we sought to evaluate the stringency of loop stability as a feature for distinguishing APOBEC-associated drivers from passengers. We examined if it was rare for a non-recurrent mutated cytosine base within an APOBEC motif to occur within the loop of a stable hairpin structure by chance. To adjust for gene expression as a potential confounding factor, we only considered non-recurrent APOBEC-motif mutations in genes with an expression level matching that of the genes with a known passenger mutation (± 1%). From a total of 14,565 mutations satisfying the above criteria in the BCa cohort (*n* = 602), we randomly selected 1000 mutations, predicted for each the ± 12 bp stem-loop structure, and obtained the *Δ*G (a mutated cytosine not in a loop and/or *Δ*G > 0 were given a zero *Δ*G). We then mapped the quantile locations of the *Δ*Gs of the 7 known passenger mutations in the *Δ*G distribution of these 1000 gene-expression-matched non-recurrent APOBEC-motif mutations.

### Similarity-based driver/passenger prediction by joint analysis of stem-loop stability and gene expression

Genes that are not expressed are unlikely to be driver genes. Given the findings that the normalised loop stability score and gene expression rank can distinguish functional importance of mutations, we combined these two parameters to predict the ‘driverness’ for the remaining mutations, using an iterative similarity-based approach. In brief, initially for each mutation, we calculated the mean difference between the Euclidean distance with known drivers and passengers and determined the statistical significance using two-sided Student’s *t* test with heteroscedastic variances, given the known drivers were close to each other but the known passengers more dispersed in the two-dimensional space. For mutations showing statistically significant difference between distances with drivers and passengers, we predicted them as drivers if closer to known drivers and otherwise as passengers. We then repeated this process iteratively for the mutations not determined in previous iterations, by taking into consideration also the driver/passenger labels predicted in the previous iterations. The iteration was stopped once any of the following criteria reached: (i) all mutations were predicted as driver or passenger; (ii) remaining mutations had no significant difference between distances with drivers and passengers both known and predicted.

As for false discovery rate (FDR) estimation for the driver / passenger predictions, we took a permutation-based approach. In brief, we first calculated for each of the predicted drivers and passengers the probability by chance of having a mean difference of distance (MDoD) to known drivers and passengers equal to and larger (for predicted passengers) / smaller (for predicted drivers) than the observed MDoD, by randomising the labels of the known driver and passenger mutations (number of known drivers = 9; number of known passengers = 7) till the full combinatorial set (number of all possible combinations = ${C}_{16}^9$, ie 114,400). Based on the coordinates (loop stability and gene expression) of the predicted drivers / passengers and the fully randomised coordinate sets for the known drivers and passengers, we built for each prediction a distribution of expected MDoD which was then compared against the actually observed MDoD to derive the by chance probability. This probability was further subjected to Benjamin-Hochberg adjustment for multiple testing to produce the corrected final FDR estimation.

### Oncoprint of known and predicted driver mutations

To visualise the presence of and potential interaction between the known and predicted driver mutations, we plotted the oncoprint for all known and predicted driver mutations and calculated the co-occurrence and mutual exclusivity relationships among them, using the *maf tools* R package.

### Cancer effect size

Cannataro et al. [42] proposed an appropriate ranking—the cancer effect size—which is the selection intensity for somatic variants in cancer cell lineages and can be used as measurements to estimate functional importance of each mutation. We applied this method to *TBC1D12* and *AHR* mutations to compare selection intensity of different hotspot mutations within these genes. The lower value represents relatively less important function.

### AhR regulon activity in BCa tumours

We first collected a set of genes that were potentially involved in the AhR transcriptional program in BCa tumours. We included the genes whose expression level was positively correlated (measured by Spearman correlation analysis) with both *AHR* and *CYP1A1*, the established prototypical target of AhR [43], at a strength no less than the correlation between these two genes (Spearman’s rho = 0.29, *P* = 1.6 × 10^− 9^) (within the genes with RSEM normalised RNA-seq data of TCGA BCa, *n* = 406, after excluding two samples without clear consensus classification [44] and the bottom 20% of genes with small variance). Then, to validate that the candidate genes (*n* = 196) were regulated by AhR, we performed a differential expression analysis using the DESeq2 approach [45] in a public data set containing *AHR* siRNA and negative control siRNA-treated MCF-7 breast cancer cell lines (GSE52036, RNA-seq transcriptome in raw count with 37,640 non-zero features and 4 biological replicates for each treatment group [46]). Differentially expressed genes were ranked by taking into account both log2 fold change and FDR-adjusted significance, with genes most significantly downregulated by *AHR* knockdown ranked on top. As demonstrated by 1,000,000 times randomisation test, the observed rank sum of the 196 candidate genes was significantly much lower than random (*P* < 1 × 10^− 6^), indicating an enrichment of these genes at the top of the ranking by response to *AHR* knockdown. We then took the intersection of genes potentially involved in the AhR transcriptional program and genes significantly downregulated (FDR < 0.05) by *AHR* knockdown as the final gene set of the AhR regulon in BCa tumours (*n* = 25; Additional file 5: Table S4). We applied the recently published consensus classification of muscle-invasive BCa [17] to 406 TCGA BCa tumours [44]. Considering only genes expressed by both tumours and cell lines, we adapted this classification and classified 28 BCa cell lines (data from Cancer Cell Line Encyclopedia (CCLE) project) [47]. We grouped luminal papillary, luminal unstable and luminal non-specified tumours as luminal subtype and others as non-luminal subtype. AhR activity, calculated using gene set variation analysis (GSVA) [48] based on aforementioned AhR regulon, was compared between luminal and non-luminal groups of BCa tumours.

### APOBEC signature in luminal and non-luminal BCa tumours

The fraction of APOBEC mutations for each of 404 patients and their corresponding molecular classifications were described as above. The fraction of APOBEC mutations were compared between luminal and non-luminal groups of BCa tumours using the Wilcoxon rank sum test.

### Cell culture

The human bladder cancer-derived cell lines UMUC7, UMUC14, RT112, RT4, VMCUB1, SCaBER, UMUC6, T24 and HT1197 were obtained from DSMZ (Heidelberg, Germany). KMBC2 cells were purchased from JCRB cell bank (Japan). KMBC2 cells were cultured at 37 °C in an atmosphere of 5% CO_2_ in Ham F12 medium, RT112 and RT4 cells were cultured in RPMI medium and all the other cells were cultured in DMEM medium. All cell media were supplemented with 10% fetal bovine serum (FBS). Cells were routinely tested for mycoplasma contamination.

### Cell viability assay

KMBC2, UMUC7, UMUC14, RT112, RT4, VMCUB1, SCaBER, UMUC6, T24 and HT1197cells were seeded in triplicate in 96-well plates and left to adhere overnight. Afterwards, cells were treated for 72 h with gradient concentrations: from 1.25, 2.5, 5, 10 to 20 μM for AhR inhibitor (CH-223191). Control cells were treated with DMSO. Cell viability was assessed with the CellTiter-Glo assay (Promega) after 72 h of treatment. The CH-223191 inhibitor was purchased from Selleckchem (Cat.S7711, EUROMEDEX, France).

### Response of BCa cell lines to AhR/ARNT knockout/inhibition

We explored cell viability dependency to *AHR* and *ARNT* knockout in BCa cell lines available from the DepMap data repository (20Q2 version, *n* = 28) [49]. We investigated the correlation between *AHR* and *ARNT* dependency scores (CERES). We calculated for each cell line the average of these two scores, as the measurement for its dependency on the AhR/ARNT complex. KMBC2, UMUC7, UMUC14, RT112, UMUC1 and UMUC9 were classified as luminal subtype and others classified as non-luminal subtype. We plotted the AhR/ARNT dependency scores against corresponding cumulative fractions, separately for the luminal and non-luminal subtypes, and compared luminal cell lines’ AhR/ARNT dependency scores and their quantile counterparts in non-luminal cell lines (directly extracted or obtained using localised linear interpolation), using the Wilcoxon signed-rank test.

We also measured the sensitivity to an AhR-specific inhibitor (CH-223191) in aforementioned cell lines (*n* = 10) that were cultured in the host lab. Of note, KMBC2 cells harboured *AHR* Q383H mutation; UMUC7 and HT1376 cells harboured both *AHR* and *ARNT* amplification; 647V cells presented *AHR* amplification; and JMSU1 and UMUC10 presented *ARNT* amplification. Cell viability was normalised relative to DMSO control.

### Statistical and bioinformatics analysis

Wilcoxon’s rank sum test, Fisher’s exact test and Kruskal-Wallis tests were used for the comparisons. A value of *P* < 0.05 in two-tailed tests was considered statistically significant. R version 3.5.2 and the *ggpubr*version 0.2 package were used for all analyses and for the associated visualisations.

## Results

### Identification of 44 APOBEC-associated hotspot mutations in BCa

**Fig. 1:**
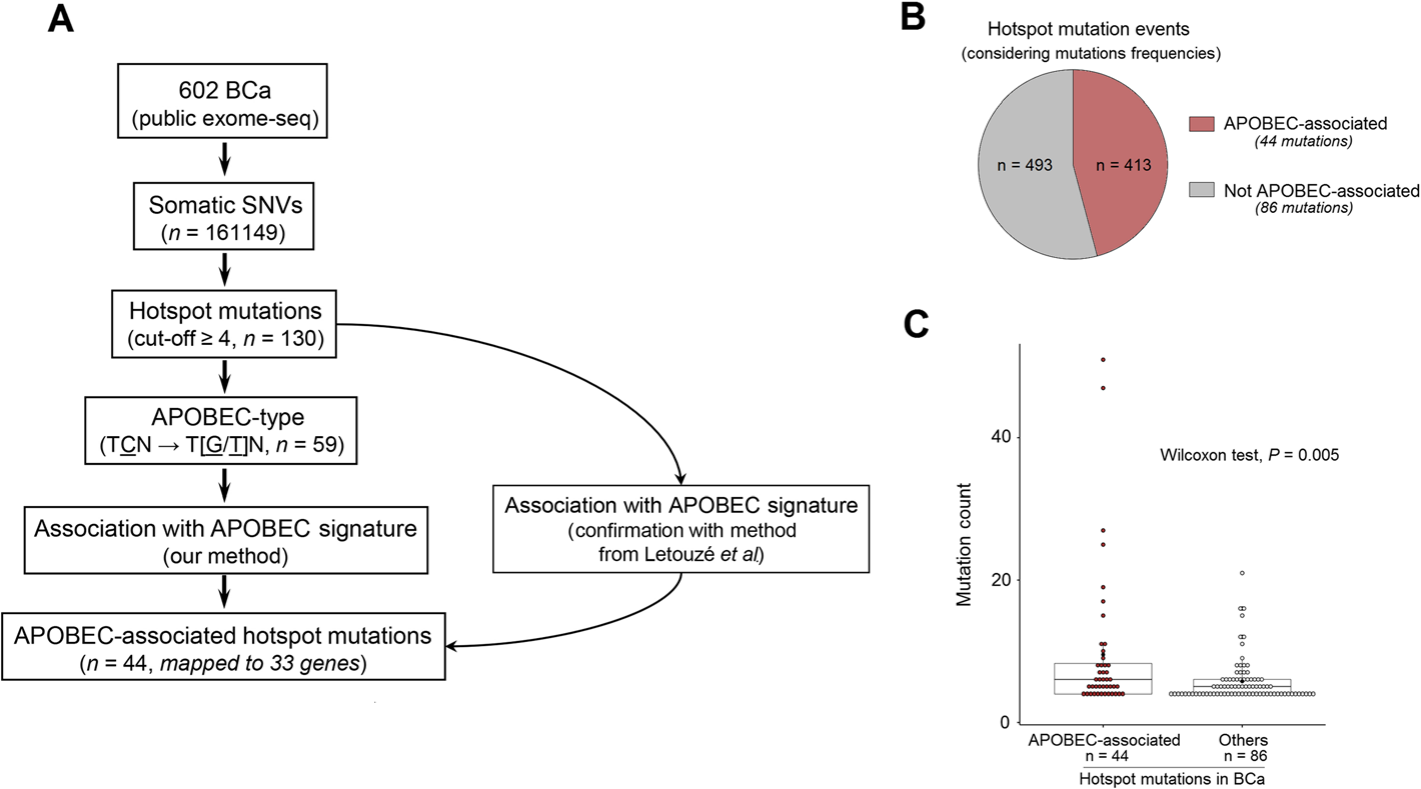
Identification of APOBEC-associated hotspot mutations and their landscape in bladder cancer. **a** Workflow to identify APOBEC-associated hotspot mutations (*n* = 44) in 602 published exome-sequenced bladder cancers. Hotspot mutations were defined as counts ≥ 4 (‘Methods’). APOBEC signature was quantified by the sum of fraction scores of COSMIC signatures 2 and 13 [3]. In our method, we compared the sum of APOBEC signature fraction scores between tumours bearing a given candidate hotspot mutation corresponding to an APOBEC-type motif (TCN → T [G/T]N) and tumours without any of such candidate mutations. The method from Letouzé et al. estimates the probability of each mutation being due to each mutational process without restriction of stringent motifs [35]. **b** Proportion of APOBEC-associated hotspot mutation events among overall hotspot mutation events in tumours bearing at least one of 130 hotspot mutations (counts ≥ 4) in BCa. **c** Comparison of mutation frequencies between APOBEC-associated hotspot mutations (*n* = 44) and other hotspot mutations (*n* = 86). *P* value: Wilcoxon test

The strategy to identify APOBEC-associated hotspot mutations is presented in Fig. 1a. We analysed publicly available whole-exome sequencing data for somatic SNVs in 602 BCa tumours. We identified 161,149 different mutations and their frequency followed a long-tail distribution, as reported for pan-cancer genome-wide mutation profiles [50] (Additional file 3: Fig. S2a). We determined that a frequency ≥ 4 was an optimal threshold to distinguish the ‘head’ (defined as hotspot mutations, *n* = 130 mapped to 75 genes, Table S1) from the ‘tail’ within the distribution (Additional file 3: Fig. S2b and ‘Methods’). To pinpoint to APOBEC-associated hotspot mutations, we first selected hotspot mutations presenting an APOBEC-type motif (TCN → T [G/T] N, N = any base) as candidate APOBEC-associated hotspot mutations (*n* = 59) (Additional file 1: Table S1). Although TCW (W = A or T) is commonly considered for APOBEC-type motifs, we did not restrict our search to TCW context given the growing evidence of APOBEC-induced mutations corresponding to TCN but non-TCW motifs [9, 10, 51, 52], including *FGFR3* S249C which we recently proved to be APOBEC-induced using a deamination assay [4–6]. Then, we hypothesised that tumours with a genuine APOBEC-associated hotspot mutation should present high fraction scores of previously defined mutational signatures of APOBEC-mediated mutagenesis (namely COSMIC mutational signatures 2 and 13) [3]. We thus compared the APOBEC-mediated mutagenesis fraction scores of the tumours with any of the 59 candidate APOBEC-associated hotspot mutations to those without any of them (Additional file 3: Fig. S3). Using this approach, we identified 44 hotspots (mapping to 33 genes) with significantly higher fraction scores, further classifying them as APOBEC-associated hotspot mutations (Fig. 1 and Table 1; ‘Methods’).

**Table 1:** List of 44 APOBEC-associated hotspot mutations and their frequencies in bladder cancer (*n* = 602)

***Gene*** mutation	N. of mutated samples
*FGFR3* S249C	51
*PIK3CA* E545K	47
*ERBB2* S310F	27
*PIK3CA* E542K	25
*RXRA* S427F	19
*TP53* E285K	17
*TP53* R280T	15
*KDM6A* Q555*	11
*TBC1D12* *1*	11
*C3orf70* S6L	10
*RHOB* E172K	9
*AHR* Q383H	8
*LPAR6* F316F	8
*TP53* Q331*	8
*TP53* E271K	8
*RARS2* R6C	7
*SF3B1* E902K	7
*TP53* R280K	7
*ERBB3* E332K	6
*MROH2B* E1109K	6
*PIK3CA* E545Q	6
*PPCS* S113L	6
*STAG2* Q593*	6
*ACSS3* S290L	5
*CELSR3* E356K	5
*KCNF1* E158K	5
*PDE3A* L275L	5
*PIK3CA* E726K	5
*PLXNA2* E1480K	5
*RHOB* E47K	5
*TFPI2* R222C	5
*TP53* K132N	5
*CAMK2G* I132I	4
*CELSR1* E1382K	4
*EP300* Q1082*	4
*ERBB4* E317K	4
*FAM90A1* L251L	4
*FURIN* R693W	4
*KDM6A* S1061*	4
*PBX2* E70K	4
*RB1* Q217*	4
*RREB1* Q392*	4
*TP53* Q192*	4
*TTC23L* Q263Q	4

To confirm these results, we used another approach developed by Letouzé and colleagues [35] to infer the mutagenic processes accounting for each of the 130 hotspot mutations. In accordance with our method, the 44 mutations we highlighted were attributable to APOBEC mutagenesis whereas other hotspot mutations were mostly associated with other mutagenic processes such as ageing (70/86, Additional file 3: Fig. S4). We found that these APOBEC-associated hotspot mutations accounted for almost half of the total number of overall hotspot mutation events (Fig. 1b), and their recurrence rate was significantly higher than that of non APOBEC-associated hotspots (Fig. 1c), indicating that APOBEC is a major source of hotspot mutations in BCa.

### Known and new characteristics of APOBEC-associated hotspot mutations

**Fig. 2:**
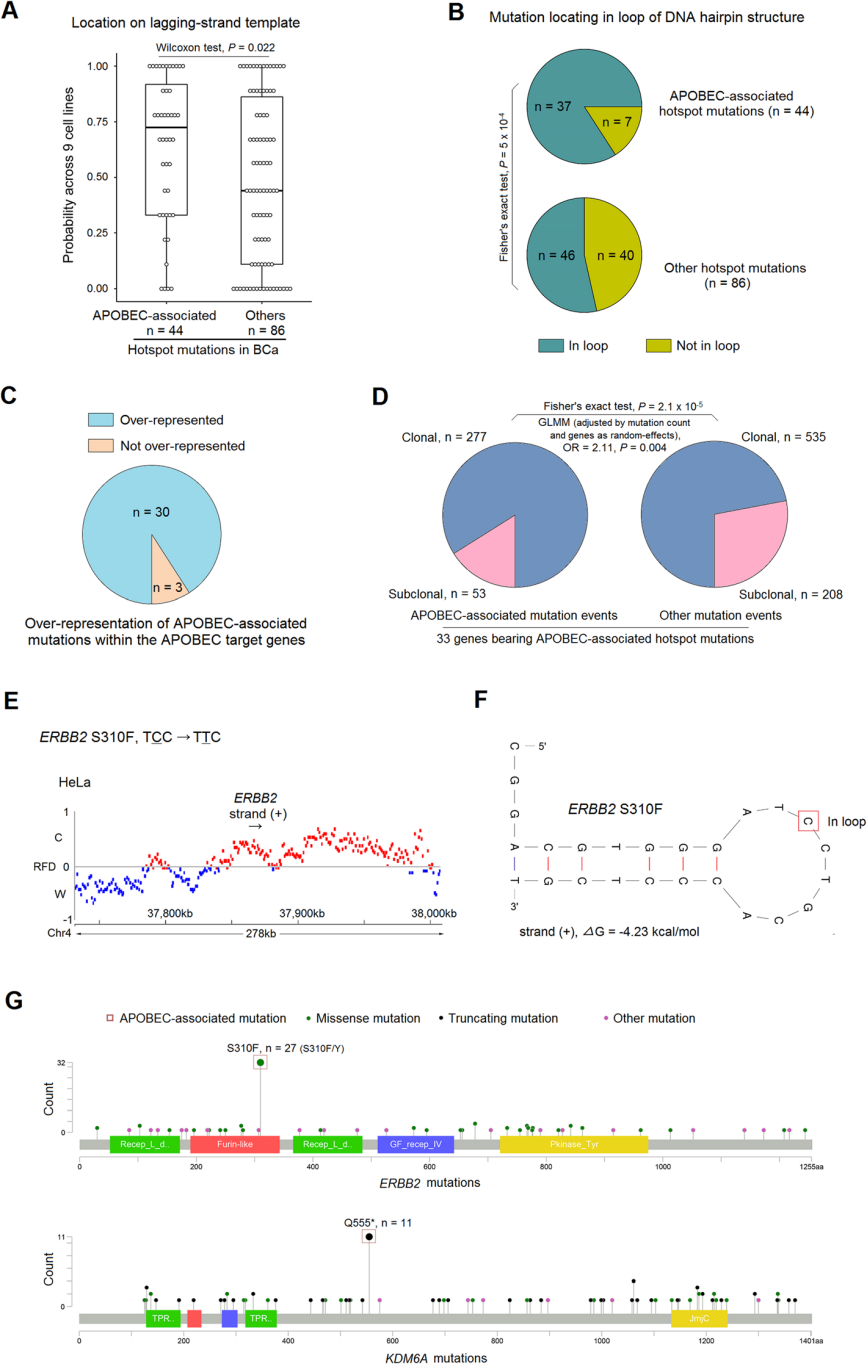
Features of APOBEC-associated hotspot mutations. **a** Comparison for probability of locating on lagging-strand-template between 44 APOBEC-associated hotspot mutations and 86 other hotspots across nine cancer/normal cell lines [37, 38]. **b** Proportion of hotspot mutations located in DNA hairpin loop structures (25 nt ssDNA centred on mutated site) for APOBEC-associated hotspot mutations and other hotspots. *P* value: Fisher’s exact test. **c** Proportion of APOBEC-associated hotspot mutations presenting a higher prevalence than the other mutations within the same genes (*n* = 33). **d** Distribution of clonal and subclonal events for APOBEC-associated hotspots and other mutations within the genes targeted by APOBEC (*n* = 33). Clonality data was extracted from TCGA BCa publication [17] which was evaluated by using ABSOLUTE algorithm [36]. *P* value: Fisher’s exact test. GLMM, generalised linear mixed model, with mutation count as covariate and genes as random effects; OR, odds ratio. **e** Representative replication fork directionality (RFD) around the *ERBB2* gene in HeLa cells, as determined by mapping Okazaki fragments to C (Crick) and W (Watson) DNA strands. Red (blue) RFD profile marks indicate the regions in which Watson (Crick) strands are replicated mostly as lagging-strand-templates. Black arrow under gene symbol for transcriptional direction. **f** Representative predicted stem-loop structure for the *ERBB2* gene. Red rectangle marks the mutation site. Free energy parameter—*Δ*G (kcal/mol) for loop stability. Strand (+) indicates cytosine (C) mutation, whereas strand (−) indicates guanine (G) mutation. **g** Representative mutation spectra for *ERBB2* and *KDM6A* genes in 602 BCa. Red rectangles indicate APOBEC-associated hotspot mutations; green dots mark missense mutations; black dots mark truncating mutations; pink dots mark other mutations. *ERBB2* S310F and *KDM6A* Q555* are APOBEC-associated hotspot mutations and have a higher prevalence compare to the other mutations within their gene sequence

In addition to motif specificity, APOBEC targets are also characterised by structural specificities. In particular, APOBEC enzymes preferably target ssDNA and hairpin loops, which enable spatial accessibility; APOBEC-related mutations are dominated by replicative but not transcriptional mutational asymmetries [33, 53–58]. Accordingly, we did not observe a coding strand bias within the 44 classified mutations (Additional file 1: Table S1) and most of the APOBEC-associated hotspots preferentially occurred in lagging-strand templates during DNA replication compared to those non APOBEC-associated hotspot mutations (Fig. 2a, across 9 cell lines [37, 38], ‘Methods’). Additionally, DNA folding predictions indicated that the APOBEC-associated hotspots were preferentially located within the loop of DNA hairpin structures compared to non APOBEC-associated ones (Fig. 2b, ‘Methods’). Comparison of gene expression in tumours with any of the 44 APOBEC-associated mutations to tumours devoid of them revealed that *APOBEC3A* and *APOBEC3H* were significantly upregulated in the mutated group, suggesting that the proteins encoded by these two genes might act as mutagens in tumours harbouring an APOBEC-associated mutation (Additional file 3: Fig. S5). We had already identified these two enzymes as potential mutagens for the *FGFR3* S249C mutation in BCa [4], reflecting an immune response-stimulated induction of APOBEC3 that may stem from infectious aetiologies of BCa [59, 60]. Additionally, in line with the fact that *APOBEC3A* favours YTCN (Y = C or T) sites whereas *APOBEC3B* favours RTCN (R = A or G) sites [61], we found that 38 of 44 (86.4%) APOBEC-associated mutations identified were YTCN types (Additional file 1: Table S1). Taken together, these structural and expression-level data provide further evidence that the 44 APOBEC-associated hotspots are indeed likely induced by APOBEC enzymes.

Strikingly, as previously observed for *FGFR3* S249C, most genes (30/33, except for *TP53*, *ERBB3* and *ERBB4*) bore APOBEC-associated hotspot mutations that presented a significantly higher prevalence than the other mutations within the same gene (Fig. 2c, g, Additional file 3: Fig. S6), suggesting that APOBEC-mediated mutagenesis shapes the mutation spectra in its target genes in BCa. In addition, within all mutation events mapped to the 33 APOBEC target genes, APOBEC-associated hotspot mutations showed higher probability of being clonal events than the other ones (Fig. 2d, ‘Methods’), indicating that APOBEC-mediated mutagenesis is an early event in BCa tumorigenesis, as previously reported [12].

Representative examples of genome-wide replication fork directionality (RFD) and DNA hairpin structure are shown for the *ERBB2* S310F mutation (Fig. 2e, f); representative examples of mutation spectra are shown for the *ERBB2* and *KDM6A* genes (Fig. 2g); and details for other mutations are shown in Additional file 3: Fig. S6 and Additional file 1: Table S1.

### Identification of APOBEC-associated hotspot mutations in other APOBEC-related cancer types

**Fig. 3:**
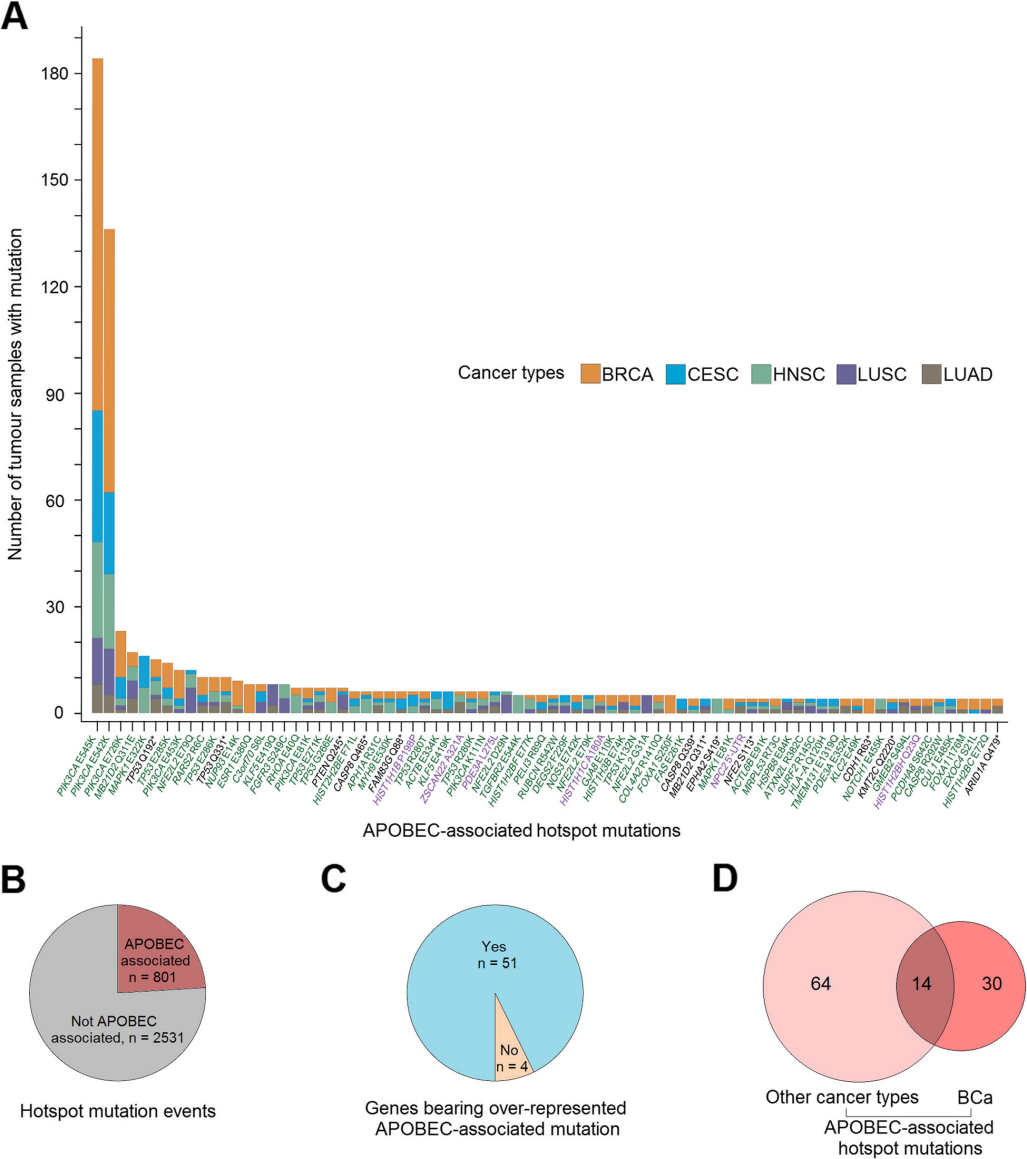
Identification of APOBEC-associated hotspot mutations in other cancer types presenting relatively high APOBEC-mediated mutagenesis, and comparison with those observed in bladder cancer (BCa). **a** Distribution of the frequencies of 78 APOBEC-associated hotspot mutations in 3744 tumours from cervical, head and neck, breast and lung cancer. **b** Proportion of APOBEC-associated hotspot mutation events among all hotspot mutation events in tumours bearing 344 hotspot mutations (counts ≥ 4) identified in four cancer types (‘Methods’). **c** Proportion of APOBEC-associated hotspot mutations presenting a higher prevalence compared to the other mutations within the same gene (*n* = 55). **d** Intersection between APOBEC-associated hotspot mutations identified in BCa and those identified in other cancer types. BRCA = breast cancer; CESC = cervical squamous cell carcinoma; HNSC = head and neck squamous cell carcinoma; LUAD = lung adenocarcinoma; LUSC = lung squamous cell carcinoma and BCa = bladder cancer

We investigated whether these features of APOBEC-associated hotspot mutations could be generalised to other cancer types presenting relatively high APOBEC-mediated mutagenesis (cervical, head and neck, breast and lung cancer) [2]. We pooled together all mutations from these four cancer types (from 3751 patients) and applied a similar workflow as previously done in BCa. We thereby identified 112 candidate APOBEC-associated hotspots, 78 of which (mapping to 55 genes) had significantly higher APOBEC-mediated mutagenesis and thus were classified as APOBEC-associated hotspot mutations (Fig. 3a, Additional file 2: Table S2 and Methods). As observed in BCa, these mutations were more likely to occur in lagging-strand templates (median probability = 0.78, *P* = 2 × 10^− 6^, ‘Methods’) and/or within loop structures (51/78, probability = 0.65, *P* = 4 × 10^− 4^, ‘Methods’) respectively against random, increasing their likelihood of being induced by APOBEC enzymes (Additional file 2: Table S2, ‘Methods’). Although APOBEC-mediated mutagenesis also significantly contributed to hotspot mutations in these cancer types, it was less common than in BCa, highlighting the particular importance of APOBEC in BCa (Fig. 3b). However, similarly to BCa, in 93% of the cases, the identified APOBEC-associated hotspots were significantly more frequently mutated than the other mutations within the same gene (Fig. 3c and Additional file 3: Fig. S7). Although selective functional advantage of a mutation can be cancer-type specific (e.g. enrichment of *FGFR3* mutations in BCa) and the distribution of attributable mutagenic processes vary from one cancer type to another (e.g. dominant APOBEC mutagenesis in BCa), 32% (14/44) of the APOBEC-associated hotspot mutations identified in BCa were also found in other cancer types with high APOBEC mutagenesis activity (Fig. 3d).

### Prediction to distinguish drivers from passengers within APOBEC-associated hotspot mutations

**Fig. 4:**
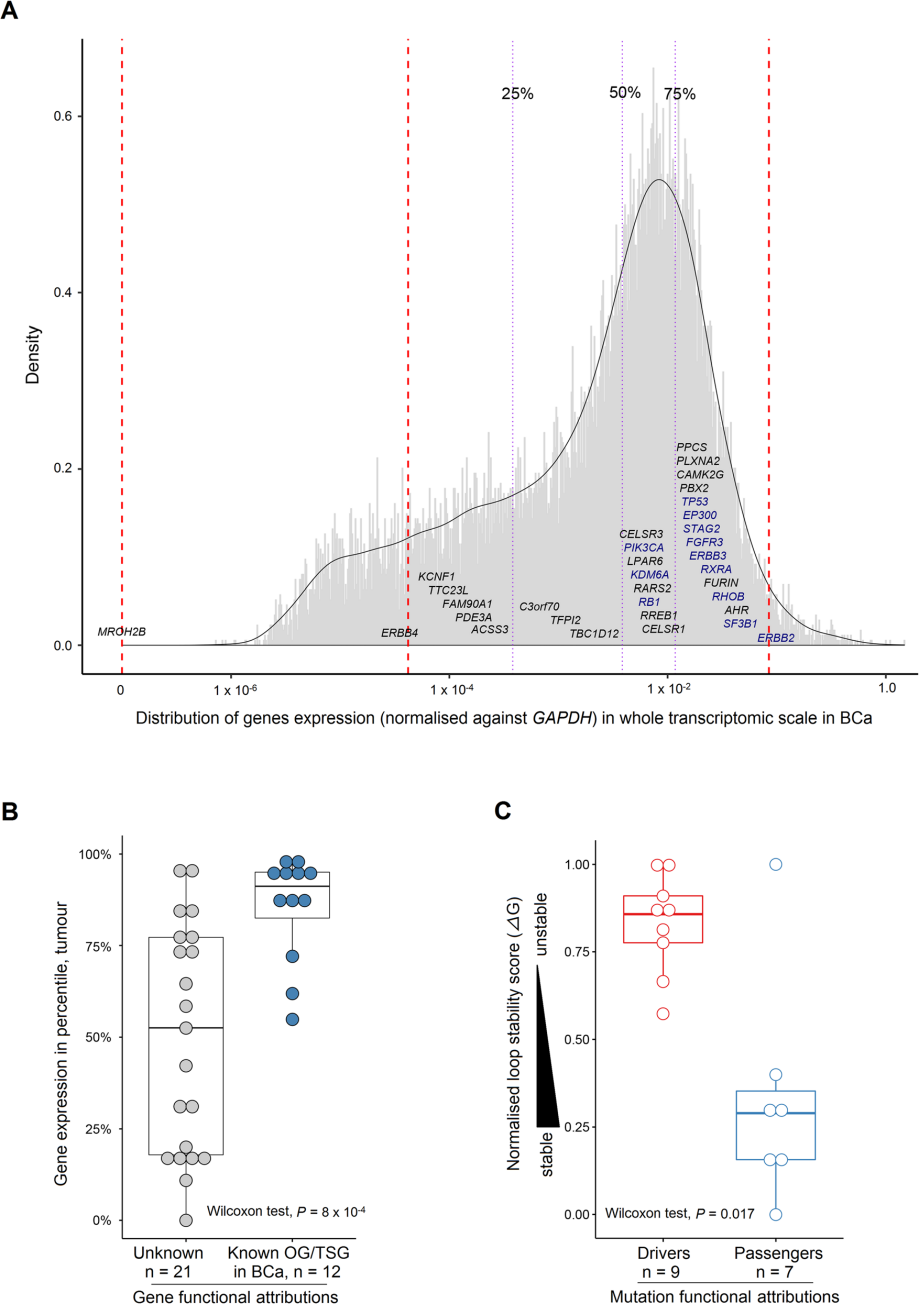
mRNA expression levels of APOBEC-target genes and DNA loop stability of APOBEC-associated known driver and passenger mutations in bladder cancer (BCa). **a** Distribution of mRNA levels (normalised against *GAPDH*) for APOBEC-target genes at the whole-transcriptome scale in BCa from The Cancer Genome Atlas (TCGA) [17]. The percentile ranks of genes were converted into quartiles. Blue and black fonts indicate known OG/TSGs and other genes, respectively. **b** Distribution of the expression ranks in percentile in BCa between APOBEC-target known OG/TSGs and genes with unknown function. **c** Distribution comparison of the loop stability score between APOBEC-associated known driver and passenger mutations. Higher normalised *Δ*G (kcal/mol) scores reflect lower loop stability. Passengers include 5 silent mutations, 1 mutation within a transit peptide and 1 missense mutation on a gene with an absence of mRNA expression. The functional aattributions for mutations and genes are curated from a recent publication [41]. **b**, **c** *P* value: Wilcoxon test. OG, oncogene; TSG, tumour suppressor gene

It is widely assumed that hotspot mutations are likely to be gain-of-function mutations affecting oncogenes and that loss of function mutations affecting tumour suppressor genes (TSGs) are non-recurrent, with the exception of dominant-negative mutations [62, 63]. However, Buisson et al. have very recently highlighted that mesoscale structures could favour APOBEC-associated passenger hotspot mutations [9]. We therefore aimed at investigating the functional properties of APOBEC-associated hotspot mutations in BCa. We classified the 44 APOBEC-associated hotspot mutations in BCa regarding mutation nature (driver or passenger) and/or type of the affected gene (TSG or proto-oncogene), by using the findings of a recent comprehensive study in which 299 cancer genes and 579 driver mutations were functionally annotated (Additional file 1: Table S1) [41]. As expected, we observed gain-of-function driver mutations affecting proto-oncogenes (*n* = 4), such as *FGFR3* S249C and *ERBB2* S310F, and missense mutations of undetermined function but mapping to known oncogenes (*n* = 5), such as *RXRA* S427F, or to known TSGs (*n* = 6), such as *RHOB* E172K/E47K. Interestingly, we also observed hotspot nonsense mutations mapping to five known TSGs (*TP53*, *KDM6A*, *STAG2*, *EP300* and *RB1*). Surprisingly, these TSG hotspot nonsense mutations seemed unlikely to exert dominant-negative activity since the mRNA levels of these TSGs were significantly lower in tumours bearing APOBEC-associated nonsense mutations compared to wild-type tumours (Additional file 3: Fig. S8a) and two TSGs were even located in the X chromosome. We also identified seven hotspot mutations that were very likely to be passengers, including five silent mutations, one mutation affecting a transit peptide (*RARS2* R6C), and another mutation of the *MROH2B* gene showing an absence of mRNA in BCa (Fig. 4a, Additional file 1: Table S1 and Methods).

**Fig. 5:**
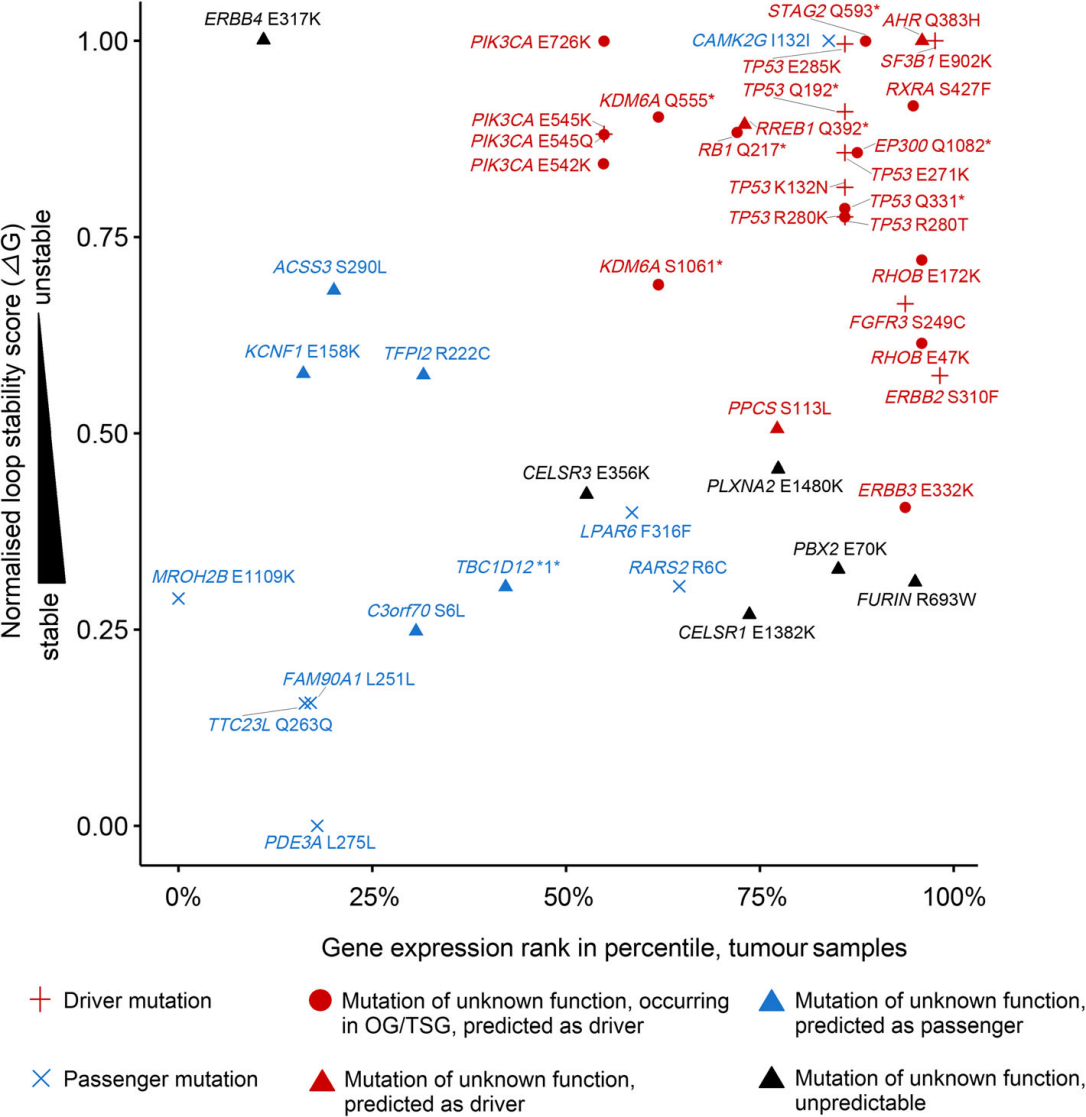
Prediction for new ‘driver’ and ‘passenger’ hotspots using combined analysis of DNA loop stability and mRNA expression levels of APOBEC-target genes in bladder cancer (BCa)**.** Higher normalised *Δ*G (kcal/mol) scores reflect lower loop stability. Gene expression level (normalised against *GAPDH* gene) is presented by rank at the whole-transcriptome scale in tumours samples. Passengers include 5 silent mutations, 1 mutation within a transit peptide and 1 missense mutation on a gene with an absence of mRNA expression. The functional attributions for mutations and genes are curated from a recent publication [41]. OG, oncogene; TSG, tumour suppressor gene

To distinguish APOBEC-associated driver from passenger hotspot mutations in BCa, we evaluated both the stability of DNA hairpin structures (estimated with free energy parameter—*Δ*G [40]) in which APOBEC-associated hotspot mutations occurred and the mRNA expression levels of the genes bearing these mutations. We found that known oncogenes/TSGs were almost systematically expressed at higher levels than average in tumours, whereas expression levels were very heterogeneous for genes of unknown function (Fig. 4b and ‘Methods’). The higher expression of known oncogenes/TSGs was further confirmed across pan-cancer types by exploring the well-annotated 299 cancer genes [41] (Additional file 3: Fig. S9a, ‘Methods’). We also showed that the seven known APOBEC-associated hotspot passenger mutations were significantly located in more stable loops than known driver mutations (Fig. 4c and ‘Methods’). Strengthening this point, we further determined that it was rare that a non-recurrent (therefore likely passenger) APOBEC-motif mutation happened to be in the loop of a stable DNA hairpin by chance (Additional file 3: Fig. S9b, Methods). We then combined the two parameters, loop stability and gene expression level, to predict the nature of mutations of unknown function. As a result, we were able to classify 22 of 28 mutations of unknown nature: 17 as drivers and 5 as passengers (Fig. 5, ‘Methods’). All predictions were found with an FDR < 0.05, except for the *CELSR3* E356K mutation which was predicted as passenger with an FDR = 0.072 (Additional file 3: Fig. S10). Finally, we created an oncoprint of all the driver mutations (known and predicted) observed in BCa (Additional file 3: Fig. S11a) and found that most of them showed low co-occurrence and their average number per tumour was 1.33 (range 1–4) (Additional file 3: Fig. S11b).

Most of the mutations with an unknown functional impact based on a large-scale functional attribution study of mutations [41], but affecting either known oncogenes (such as *RXRA* S427F) or TSGs (such as *RHOB* E172K/E47K), were predicted to be driver mutations. Supporting our prediction model, *RXRA* S427F mutation was recently demonstrated to induce ligand-independent activation of the PPARG/RXR pathway and to display pro-tumorigenic activity in BCa [64]. *RHOB* E172K and *RHOB* E47K mutations were also recently reported to be inactivating mutations impairing the stability of RHOB protein [21]. We also predicted some driver mutations in genes that were not reported to display oncogenic or tumour suppressive properties in BCa, such as *AHR* Q383H and *RREB1* Q392*. We suspected that *RREB1* Q392* might be a hotspot loss-of-function mutation and the gene itself a TSG in BCa, given the fact that samples with the *RREB1* Q392* mutation displayed significantly lower levels of expression for this gene than non-mutated tumours (Additional file 3: Fig. S8b). We also predicted new passenger mutations including *TBC1D12* (c.-1G>A) 5′-UTR mutation which was however recently been suggested to be a driver mutation by Buisson et al. [9]. Of note, previous work showed that although another frequent *TBC1D12* mutation (c.-3C>T) alters *TBC1D12* gene expression, the c.-1G>A mutation does not [65]. Since both mutations predominantly occur in bladder cancer, we computationally quantified their respective selection intensity using the cancer-effect-size approach [42], which measures a mutation’s contribution to tumour fitness, including its effect on cell transformation, proliferation and survival, in 602 exome-sequenced BCa (‘Methods’). The c.-1G>A mutation had a very low selection intensity, while the c.-3C>T had the highest among all *TBC1D12* mutations (353.2 vs. 11,871.1, a 33.6-fold difference, Additional file 3: Fig. S12). These additional results support our prediction of *TBC1D12* (c.-1G>A) as a passenger event.

#### Identification of AhR as a potential therapeutic target in BCa

The ligand-activated transcription factor AhR has been mainly studied so far for its xenobiotic metabolising role in the field of toxicology, but emerging evidence has raised attention to its cancer-related function [66, 67]. Therefore, we further focused on AhR and studied the functional impact of *AHR* Q383H, which we predicted as a driver mutation.

**Fig. 6:**
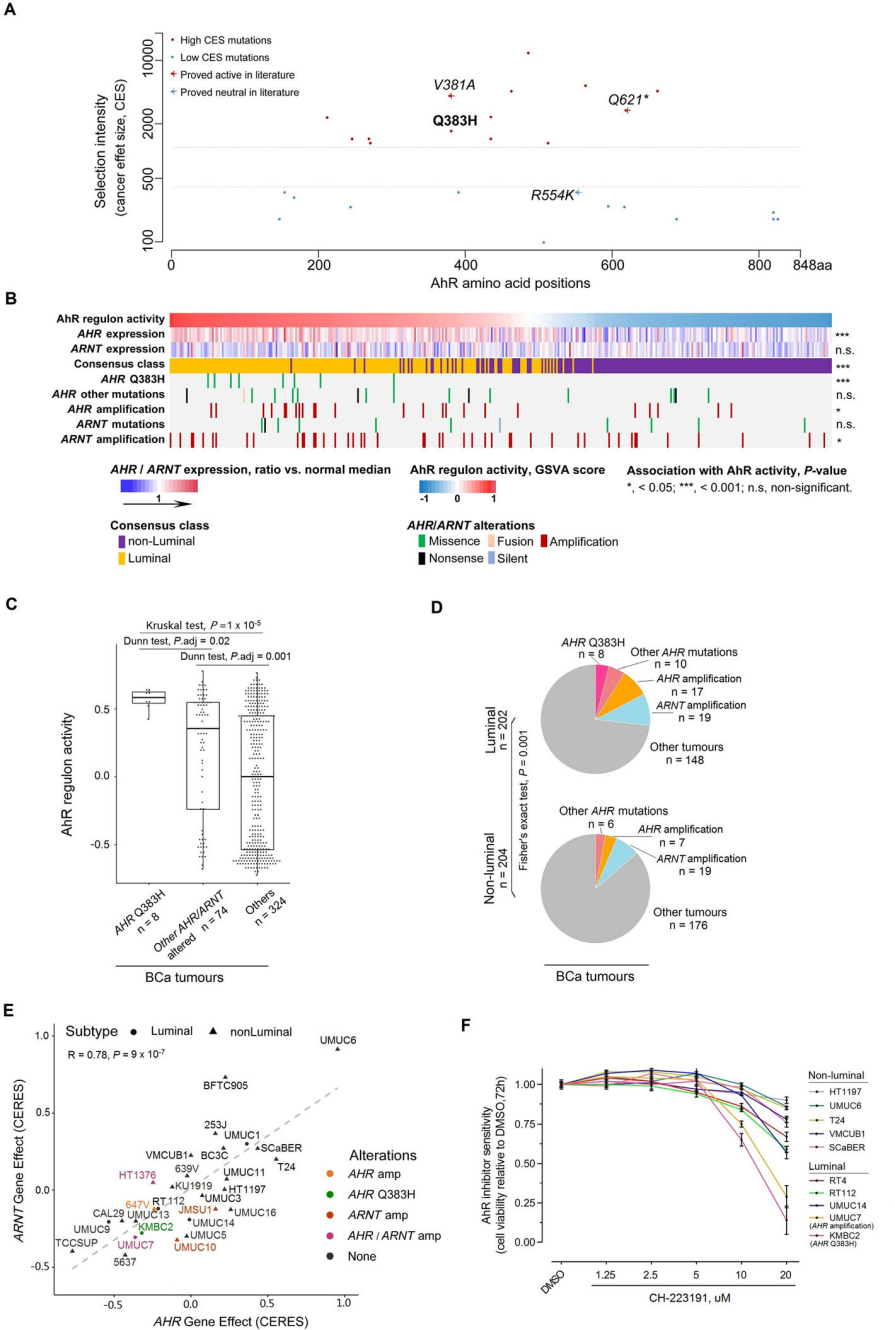
*AhR* displays a pro-tumorigenic activity in luminal bladder cancer (BCa). **a** Cancer effect size for *AHR* mutations in BCa. Mutations were clustered into two groups based on cancer effect sizes (low or high). Mutations with determined functional impact were computationally added for reference. **b** Heatmap showing relationships among tumour molecular classes, *AHR/ARNT* gene expression, *AHR/ARNT* genetic alterations and AhR regulon activity. **c** Association between *AHR/ARNT* genetic alterations (mutations and amplifications) and AhR activity in BCa. AhR activity was calculated using gene set variation analysis (GSVA) based on AhR regulon in BCa tumours (‘Methods’). *P* values were from Kruskal’s test across groups and Dunn’s test with FDR adjustment for pairwise comparisons. **d** Distribution of *AHR/ARNT* genetic alterations (mutations and amplifications) in BCa tumours. Tumour molecular classes are based on a recently published consensus classification of BCa [44], where luminal papillary, luminal unstable and luminal non-specified tumours were grouped as ‘luminal’ (*n* = 202) and others as ‘non-luminal’ (*n* = 204) (‘Methods’). *P* value: Fisher’s exact test. **e** Correlation between *AHR* and *ARNT* dependency among BCa cell lines was evaluated (Pearson’s correlation, *R* = 0.78, *P* = 9 × 10^− 7^). Cell viability dependency scores to *AHR* and *ARNT* knockout (using CRISPR-cas9) in BCa cell lines were available from the DepMap data repository (20Q2 version, *n* = 28) [49]. *AHR*/*ARNT* genetic alterations and subtypes were colour-coded and symbol-coded, respectively. **f** Response to AhR inhibition in BCa-derived cell lines (*n* = 10). CH-223191 is an AhR-specific inhibitor. All cells were treated for 72 h with either DMSO or following inhibitor concentration: 1.25, 2.5, 5, 10, 20 μM. Cell viability is measured by CellTiter-Glo assay and is shown as normalised between inhibitor treatment and DMSO control. KMBC2 and UMUC7 cells, both classified as luminal type, harbour the *AHR* Q383H mutation (in pink) and an *AHR* amplification (in orange), respectively; Other luminal cells included UMUC14, RT112 and RT4, and the remaining cells were classified as non-luminal group (‘Methods’)

Cancer effect sizes (CES) were calculated for all *AHR* mutations in our BCa WES dataset. We applied a weighted univariate clustering algorithm [68] to the scores and identified two distinct groups with low or high CES (Fig. 6a). By computationally adding to the somatic mutation set three *AHR* mutations (randomly selected one sample per mutation) that have been experimentally demonstrated either as active (V381A and Q621*) or neutral (R554K) [69–71], we found that the two experimentally active mutations, as well as the Q383H mutation, co-clustered with the high CES score somatic mutations, while the neutral mutation, R554K clustered with the low CES score group. These data supported *AHR* Q383H as an activating driver mutation. On the other hand, the fact that the frequency of Q383H was the highest among all *AHR* mutations but not its CES further confirmed its link with APOBEC mutagenesis, as previously reported for *FGFR3* S249C [5, 6].

We then sought to determine whether other potential genomic alterations directly affected the AhR pathway in BCa, supporting its oncogenic role. We identified *AHR* amplifications, which were associated with high *AHR* expression, in 6% of BCa patients (Wilcoxon test, *P* = 1.4 × 10^− 6^; Fig. 6b). We also considered genetic alterations of the *AHR* nuclear translocator (*ARNT*) that heterodimerises with *AHR,* thereby allowing the transcription regulation of AhR target genes [72]. We found amplifications of *ARNT* in 11% of BCa, which were associated with *ARNT* overexpression (Wilcoxon test, *P* = 6 × 10^− 8^), further supporting an oncogenic role of AhR pathway activation in BCa (Fig. 6b). Considering that the activity of a transcription factor is not always correlated with its expression, we focused on estimating AhR activity by calculating AhR regulon in BCa (Additional file 5: Table S4, ‘Methods’, Fig. 6b). We showed that tumours presenting a high AhR activity were in fact associated with a high expression of *AHR* mRNA (Fig. 6b). They were also significantly enriched in *AHR* Q383H mutations, *AHR* amplifications and *ARNT* amplifications, indicating that these three genetic alterations could induce AhR activation (Fig. 6b). In good agreement with these results, we observed that tumours bearing these genetic alterations presented higher AhR activity than tumours without, especially the *AHR* Q383H mutation, which displayed the highest AhR activity, supporting its gain-of-function effect (Fig. 6c). Applying the recently published consensus classification for muscle-invasive BCa [44], we classified BCa tumours (*n* = 406, TCGA project [17]) as either luminal or non-luminal subtypes (‘Methods’). We found that tumours presenting high AhR activity were enriched in luminal tumours. Although APOBEC mutagenesis was equally distributed in luminal and non-luminal BCa tumours (Additional file 3: Fig. S13), the *AHR* Q383H mutation (*n* = 8) occurred only in luminal BCa tumours and overall *AHR* genomic alterations (mutations and amplifications) and *ARNT* amplifications were enriched in this subtype (Fig. 6d).

We finally evaluated the functional dependency of BCa tumour cells on these genetic alterations. We first took advantage of publicly available data regarding the effect of CRISPR-Cas9-mediated knockout of *AHR and ARNT* on 28 BCa-derived cell lines (luminal, *n* = 6 and non-luminal, *n* = 22), including 6 with genetic alterations of either *AHR* or *ARNT* (Fig. 6e, ‘Methods’). In good agreement with AhR and ARNT acting as a heterodimer, *AHR* and *ARNT* dependency scores were highly correlated (Pearson correlation test, *R* = 0.78, *P* = 9 × 10^− 7^) (Fig. 6e). KBMC2 cells bearing an *AHR* Q383H mutation were among the most dependent cells on *AHR* and *ARNT* expression for their viability, further supporting the pro-oncogenic driver role of this mutation (Fig. 6e). Three out of five other cell lines presenting *AHR* or *ARNT* amplifications were also impacted by *AHR* and *ARNT* knockout, also suggesting a pro-tumorigenic role of these alterations leading to AhR pathway activation. Of note, 6 cell lines (luminal, *n* = 2 and non-luminal, *n* = 4) out of the 22 without any of the aforementioned genetic alterations within the AhR pathway were also among the more sensitive cells. Two of them presented high *AHR* mRNA expression, comparable to the level observed in UMUC7 bearing an *AHR* amplification. However, no explanation could be provided yet for the 4 other ones, which expressed *AHR* at similar levels to some other much less-sensitive cell lines (Additional file 3: Fig. S14). Considering molecular subtypes, the impact of *AHR* and *ARNT* knockout was significantly stronger in the luminal BCa cells than in the non-luminal cells (Wilcoxon signed-rank test, *P* = 0.03, Additional file 3: Fig. S15, ‘Methods’), as expected given the enrichment of the genomic alterations of the AhR pathway and its higher activity in luminal BCa. We next tested the sensitivity to an AhR inhibitor (CH-223191) of a panel of 10 cell lines (including KBMC2 and UMUC7 presenting *AHR* genetic alterations) by evaluating cell viability after 72 h of treatment (Fig. 6f). We validated that KMBC2 and UMUC7 presented an AhR dependency for cell survival and the tendency of luminal cells to be more dependent on AhR than non-luminal ones. Collectively, these data confirmed our prediction that *AHR* Q383H is a driver activating mutation which, similarly to *AHR* and *ARNT* amplifications, induces an oncogenic dependency in BCa. Our results thus reveal AhR as a potential therapeutic target for BCa tumours presenting a genetic alteration triggering an AhR pathway activation.

## Discussion

Restricting to mutations occurring within an APOBEC-type motif (TCW or extended to TCN) has been commonly accepted as the first step in the identification of putative APOBEC-associated mutations [2–4, 7–11, 51, 52]. However, it is still challenging to recognise genuine APOBEC-associated mutations among the candidates satisfying the trinucleotide context requirement. Nevertheless, complementary strategies have been deployed in several studies aiming to dissect APOBEC-associated mutations, leading to findings consistent with each other. For example, in a study on HNSC, Cannataro and colleagues integrated estimation of positive selection intensity using the cancer size effect method and APOBEC mutagenesis contribution [7]. In a complementary approach from a pan-cancer analysis by Buisson and colleagues, specific DNA secondary structure features were shown to facilitate APOBEC3A-mediated mutagenesis and further utilised to build a statistical model for prediction of APOBEC-related hotspot passengers [9]. Here, we applied a strategy based on association with high APOBEC-mediated mutagenesis. We compared APOBEC-mediated mutagenesis between tumours bearing a given candidate hotspot mutation corresponding to an APOBEC-type motif and tumours without any of such candidate mutations. We expected that this comparative approach could be more relevant to systematically identify APOBEC-associated mutations than previous studies in which comparisons were performed between tumours harbouring a candidate APOBEC-related mutation and either tumours bearing other recurrent mutations within the same target gene (an advantageous method relevant to genes with multiple recurrent mutations) [4] or wild-type samples [10, 11] regarding APOBEC-mediated mutagenesis. Using our method, we identified 44 APOBEC-associated hotspot mutations in BCa, with 33% (14/44) overlapping with other cancers presenting high APOBEC activity. Among these mutations, 48% (21/44) were already reported as APOBEC-associated in BCa or other cancer types [4–9]. The 44 APOBEC-associated hotspot mutations were also confirmed using an alternative algorithm [35], which predicts sources of mutagenic processes without initial restriction to certain motifs, strengthening the confidence of attribution of the 44 hotspot mutations to APOBEC mutagenesis.

Strikingly, the 44 identified APOBEC-associated hotspot mutations had almost systematically a higher prevalence compared to the other mutations within the same APOBEC-target gene, not only in BCa but also in other APOBEC-related cancer types. However, despite being hotspots, not all of them were gain-of-function mutations affecting oncogenes. We also found hotspot loss-of-function nonsense or missense mutations affecting TSGs without obvious dominant-negative properties, as well as passenger mutations. These results imply that APOBEC-associated drivers arise from a synergistic combination of functional advantage and the mutagenic process, whereas the mutagenic process alone induces APOBEC-associated passenger hotspots. The fact that APOBEC-mediated mutagenesis seems to be an early event during BCa tumorigenesis could additionally contribute to this phenomenon. Altogether, our findings further our understanding of BCa biology and aetiology and challenge the dogma that recurrent mutations are likely drivers and mostly gain-of-function mutations affecting oncogenes. Therefore, caution is advised regarding the way candidate driver mutations are identified from high-throughput sequencing data analyses, especially in tumours bearing APOBEC mutations. This conclusion was already raised by Buisson et al. who first described the existence of passenger recurrent APOBEC-associated mutations. Here, we emphasised also on the fact that driver recurrent mutations are not a hallmark of activating mutations affecting oncogenes but can also be inactivating mutations affecting TSGs.

Buisson et al. [9] calculated DNA substrate optimality by finely considering stem length, loop position, loop size and GC content to identify APOBEC3A-associated passenger mutations. However, the stability of the loop can also be measured with a simple and easily accessible parameter (free energy—*Δ*G, kcal/mol) [40] calculated by the Mfold tool. Considering an optimal ssDNA sequence (25 nt length) centred on the mutated nucleotide for APOBEC-associated hotspot mutations, we noted that most APOBEC-associated hotspots were located within the loop of DNA hairpins. However, the loop stability differed between likely passenger and known driver mutations, the former mostly occurring in very stable loops whereas the latter in less stable loops. By applying a similarity-based iterative joint analysis of the loop stability parameter and the mRNA expression level of genes bearing these mutations, followed by permutation-based FDR estimation, we were able to consistently distinguish APOBEC-associated driver from passenger hotspot mutations. We thereby highlighted 17 new drivers that could be potential therapeutic targets in BCa and will be worth further validating through functional studies. An easier to interpret methodological alternative could be a two-step generalised linear regression (GLR) modelling. For each APOBEC-associated mutation, a conditional ‘driverness’ probability could be calculated as the multiplication of two independent probabilities predicted from two logistic-regression models, one for the gene hosting the mutation being a gene of functional impact (based on gene expression) and the other for the mutation itself being a driver (based on loop stability). Of note, although this standard linear regression approach may suffer from sub-optimality associated with imprecise training labels in the first GLR model (i.e. known OG/TSG vs. unknown) and difficulties in determining a threshold to distinguish drivers from passengers, it outputted results close to those from our methods, with mutations predicted to be drivers by our methods systematically having the highest GLR probability compared to the other two categories (Additional file 3: Fig. S16).

Our study focused on BCa and then extended the analysis to other APOBEC-related cancer types, revealing a relatively low overlap of APOBEC-associated hotspot mutations between BCa and other cancer types, which may reflect tissue specificity of the functional advantage induced by driver mutations. Of note, we also found a low overlap for the passenger mutations that we identified in BCa and that were not systematically found in other APOBEC-related cancers, which could raise the question of false positive identification of the APOBEC-associated hotspot mutations, although identified by two independent methods. However, among the five passenger mutations validated via deamination assay to be induced by APOBEC by Buisson et al. [9], two (*NUP93* and *MB21D2*) were not found in BCa. Additionally, two silent mutations (*COL6A6* L1125L and *ALPK3* Q860Q) reported by Cannataro et al. [7], which were APOBEC-type and showed very low selection intensity, were not found in BCa either. This observation indicates that even passengers can be cancer-type specific. A possible explanation is that not only local sequences or structure are involved in defining APOBEC optimal substrate, but also large-scale parameters (such as DNA replication, gene transcription and chromatin organisation) which could differ between tumour types.

Dysregulation of AhR signalling has been involved in many cancer types, but the pro-tumoral or anti-tumoral effect of AhR is largely context-dependent. Overexpression of constitutively active AhR in transgenic mice models has been reported to induce gastric [73] and hepatocellular [74] cancer, whereas decreased AhR activity promotes neuroblastoma metastasis [75]. Here, through the study of the candidate driver mutation *AHR* Q383H, we provided a validation of the accuracy of our prediction. The importance of Q383 site in AhR activity (Q377 as the mouse equivalent) was previously suggested by its structural impact on AhR ligand binding affinity and preference by shape and H-bond [76–78]. We showed that the *AHR* Q383H mutation and other genetic alterations affecting the AhR pathway (*AHR* and *ARNT* amplifications) were associated with dependency to *AHR* expression or activity and were enriched in the luminal subtype of BCa. These findings allowed us to point-out a pro-tumorigenic activation of AhR pathway in BCa, and to propose AhR as a therapeutic target for tumours presenting such alterations. Very similar results were described, by us and others, for another transcription factor, *PPARG*. High activation of PPARγ, associated with luminal BCa, is linked to *PPARG* amplifications or activating mutations of *PPARG* or of its co-receptor *RXRA* [64, 79–82]. All of these genetic alterations leading to PPARγ pathway activation were demonstrated to be pro-tumorigenic and allowed proposing PPARγ as a therapeutic target for luminal BCa. The potential interplay between these two transcription factors involved in luminal BCa would be worth further investigations.

## Conclusions

In summary, we report new APOBEC-associated driver hotspot mutations in BCa, hence contributing to a better understanding of BCa biology and aetiology. We highlight a general feature of APOBEC-mediated mutagenesis, namely the higher prevalence of APOBEC-associated hotspot mutations compared to other mutations within a given APOBEC-target gene. Our work also shows that APOBEC can favour passenger hotspot mutations located in optimal DNA hairpin loops, challenging the dogma that all recurrent mutations are likely drivers. Finally, we identified driving genetic alterations of *AHR* and its binding partner *ARNT*, and an oncogenic dependency associated with these alterations, suggesting AhR as a potential therapeutic target in BCa.

## Supplementary information

**Additional file 1:** **Table S1.** List of 130 hotspot mutations identified in 602 bladder cancers.

**Additional file 2:** **Table S2.** List of 112 candidate APOBEC-associated hotspot mutations identified in other cancer types presenting relatively high APOBEC mutagenesis (a total of 3751 cervical, head and neck, breast and lung cancer tumours).

**Additional file 3.** Supplementary Figures S1-S16.

**Additional file 4:** **Table S3.** Matrix of previously annotated cancer genes’ expression ranks across 32 TCGA cancer types.

**Additional file 5:** **Table S4.** List of 25 genes identified as the gene set to establish aryl hydrocarbon receptor (AhR) regulon.

## Data Availability

The tumour whole-exome sequencing (WES) and RNA-seq data are publically available from cBioPortal for Cancer Genomics (http://www.cbioportal.org/) [15, 16]. Genome-wide replication fork directionality (RFD) data are available in the original publication [37, 38]. RNA-seq data of *AHR* siRNA and negative control siRNA-treated MCF-7 breast cancer cell lines are publically available in the Gene Expression Omnibus (GSE52036; https://www.ncbi.nlm.nih.gov/geo/query/acc.cgi?acc=GSE52036). Gene dependency and transcriptome data of BCa cell lines are available from The Cancer Dependency Map Project data repository (https://depmap.org). All data generated to support the conclusions of this study are included in the main manuscript and its additional supporting files.
