# Supplementary material for: Identification of new driver and passenger mutations within APOBEC-induced hotspot mutations in bladder cancer
Source: Genome Med. 2020 Sep 28;12:85. doi: 10.1186/s13073-020-00781-y (PMC7646471; doi:10.1186/s13073-020-00781-y)
Supplement: Supplementary file 3 — Additional file 3. Supplementary Figures S1-S16. [file 13073_2020_781_MOESM3_ESM.pdf]

SUPPLEMENTARY FIGURE LEGENDS

Fig. **S1.** Optimal ssDNA sequence length – 25nt – for stem-loop structure prediction. **a)** Loop stability scores (*Δ*G, kcal/mol) with gradually increased sequence length of ssDNA (starting from 13nt, 4nt increase per escalation, centred on the mutation site) considering 6 passenger mutations. Passengers include 4 silent mutations, 1 mutation within transit peptide and 1 missense mutation on gene with an absence of mRNA expression in bladder cancer. Colours represent different passenger mutations. Solid and dashed arrows represent the completion of primary and occurrence of neighbouring/ secondary stem-loop structures, respectively. **b)** Examples of predicted stem-loop structures for *PDE3A* L275L mutation across series of ssDNA lengths. *In PDF file page 4.*

Fig. S2. The frequency of all mutations in 602 bladder cancers follows a long-tail distribution **(a)** and identification of the optimal frequency threshold (counts ≥ 4) for 130 hotspot mutations **(b)**. Ratios of the number of mutations with a count larger than this integer (n(c>x)) to the number of mutations with a count equal to this integer (n(c=x)). Details of these hotspot mutations are summarised in Supplementary Tables 1. Related to Figure 1a and Table 1. *In PDF file page 5.*

Fig. S3. Identification of 44 APOBEC-associated hotspot mutations in BCa. Hotspot mutations corresponding to an APOBEC-type motif (TCN → T[G/T]N mutations, N = any base) were considered as candidate APOBEC-associated hotspot mutations (n=59). Comparisons were made for the fraction score of APOBEC-mediated mutagenesis in tumours bearing one of the given candidate APOBEC-associated hotspot mutation with tumours free of any these hotspot mutations. *P* value: Wilcoxon test. ***, *P*<0.001;**,*P*<0.01;*,*P*<0.05 and N.S. for not significant. Color scale for *P* value from lowest to highest. X axis: 44 APOBEC-associated hotspot mutations are in red and others in black. Related to Figure 1a and Table 1. *In PDF file page 6.*

Fig. S4. Attributable mutagenic process to each of 130 hotspot mutations identified from 602 bladder cancers using Letouzé *et al.* algorithm **(a)** 44 hotspot mutations classified as APOBEC-associated one according to our initial method and **(b)** 86 other hotspot mutations. Red represents tumour sample bearing the given mutation that was attributed to APOBEC mutagenesis; Blue for Age-associated mutagenesis; Green for ERCC2-related mutagenesis. *In PDF file page 7.*

Fig. S5. *AID/APOBEC* gene expression (RSEM, log2) in relation to APOBEC-associated mutations – tumours with *vs.* without any of 44 APOBEC-associated mutations in 602 bladder cancers. *P* value: Wilcoxon test between two groups. *In PDF file page 8.*

Fig. S6. APOBEC-mediated mutagenesis confers special characteristics to its target mutations, containing 43 figures in total from **a)** to **aq)**. Plots related to Figure 2a-c. For each of the included 43 APOBEC-associated hotspot mutations (mapping to 32 genes) identified in bladder cancer, we show replication fork directionality (RFD) during DNA replication in the HeLa cell line (upper panel),

predicted stem-loop structure (middle panel) and the mutation spectrum of the corresponding gene (lower panel). DNA stem-loop structures were predicted with 25 nt length ssDNA centred on mutated site. The interpretation of the results is similar to that of the example of *ERBB2* S310F shown in the main figures (Figure 2e-g). The RFDs of eight other cell lines are summarised in Supplementary Table 1. *In PDF file page 10-52.*

Fig. S7. Mutation spectra for the 55 APOBEC-target genes identified from other cancer types, including 55 figures in total. Related to Figure 3c. Red rectangles indicate APOBEC-associated hotspot mutations. The other cancer types corresponded to 3,751 tumours from patients with cervical, head and neck, breast and lung cancer. Most of the over-represented mutations are associated with APOBEC-mediated mutagenesis within its target genes. *In PDF file page 54-108.*

Fig. S8. mRNA levels (RSEM, log2) for **(a)** known and **(b)** suspected tumour suppressor genes (TSGs) according to the APOBEC-associated mutations of these TSGs – tumours with a given APOBEC-associated hotspot mutation *vs.* tumours with wild-type alleles for each known/suspected TSG in bladder cancer. *RREB1* as a suspected TSG. RNA-Seq data are available in 406 bladder cancers from The Cancer Genome Atlas (TCGA). *P* value: Wilcoxon test between two groups. *In PDF file page 109.*

Fig. S9. Evaluation for the stringency of expression and loop stability for cancer gene and likely passenger mutation. **a)** Distribution comparison of mRNA levels between known cancer genes and all genes at the whole-transcriptome scale (as background) across 32 cancer types from The Cancer Genome Atlas (TCGA). All genes' expression was rank-transformed and functional annotation for cancer genes are curated from a recent publication (Method). Cancer genes' expression ranks in 32 cancer types were shown in Supplementary Table 3. **b)** Stem-loop deltaG distribution of 1000 expression-matched non-recurrent mutations and the 7 known passenger mutations. 1000 mutations were randomly selected from non-recurrent mutations (mutated only once in the cohort) in genes with matched expression level (± 1%) to the genes hosting the known passengers (n = 7) (Methods). *In PDF file page 110.*

Fig. S10. Permutation-based FDR estimation for predicted APOBEC-associated drivers and passengers. Benjamin-Hochberg adjustment was applied for multiple testing to produce the corrected final FDR estimation (Methods). Predictions with FDR < 0.05 were considered as confident. *In PDF file page 111.*

Fig. S11. Oncoprint (A) and interaction plot (B) of the 26 driver mutations. Driver mutations include the known drivers (n=9) and predicted ones (n=17). *P*-value for probability of mutations being co-occurred or mutual exclusive. *In PDF file page 112.*

Fig. S12. Functionality evaluation for *TBC1D12* mutations in bladder cancer (BCa). **a)** *TBC1D12* Mutation spectrum in BCa. Red rectangle marked *TBC1D12* (c.-1G>A) 5'-UTR mutation which was APOBEC-associated. **b)** Computed selection intensity of *TBC1D12* mutations. Selection intensity was calculated by cancer effect size algorithm that estimates functional importance of each mutation (Method). Red dot marked *TBC1D12* (c.-1G>A) 5'-UTR mutation which was APOBEC-associated and showed a very low intensity, indicating less important function of this mutation. *In PDF file page 113.*

Fig. S13. Distribution of the fraction of APOBEC signature mutations between tumors of luminal and non-luminal subtypes. *P*-value for wilcoxon rank-sum test. *In PDF file page 114.*

Fig. S14. Correlation between *AHR* gene expression and GRISPR-mediated AhR knock-out effect. TPM, transcripts per million . *In PDF file page 115.*

Fig. S15. Scatter plot showing BCa cell lines' AHR/ARNT dependency scores against corresponding cumulative fractions by subtype. P-value from Wilcoxon signed-rank test comparing luminal cell lines' dependency scores and their quantile conterparts in nonLuminal cell lines, either directly extracted or obtained by localized linear interpolation. *In PDF file page 116.*

Fig. S16. GLR (generalized linear regression modeling) predicted driver probability by similarity-based classification. *In PDF file page 117.*

Fig. S1

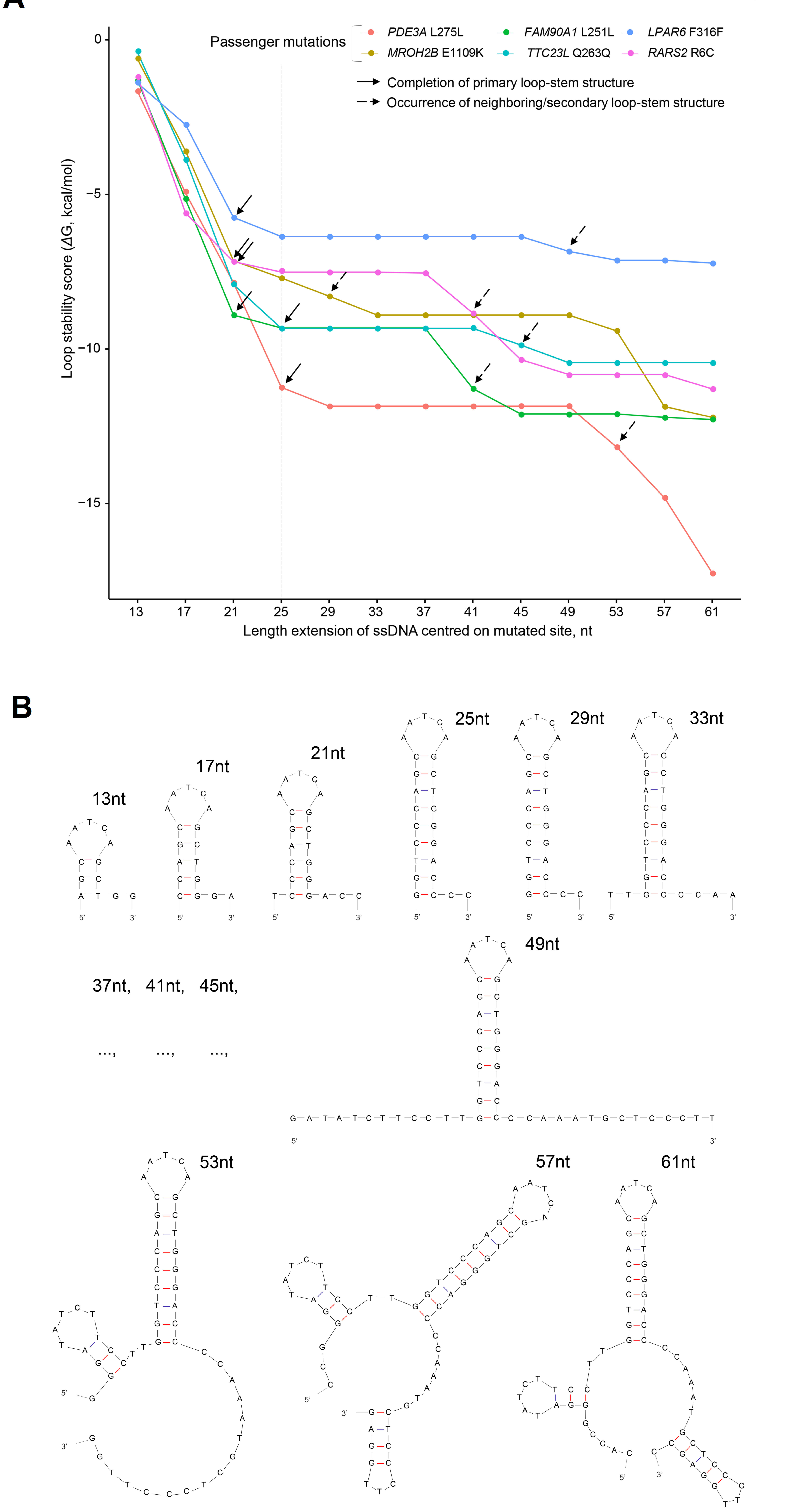
A
Passenger mutations
PDE3A L275L
MROH2B E1109K
FAM90A1 L251L
TTC23L Q263Q
LPAR6 F316F
RARS2 R6C
Completion of primary loop-stem structure
Occurrence of neighboring/secondary loop-stem structure
Loop stability score (ΔG, kcal/mol)
0
−5
−10
−15
13
17
21
25
29
33
37
41
45
49
53
57
61
Length extension of ssDNA centred on mutated site, nt
B
13nt
17nt
21nt
25nt
29nt
33nt
37nt, 41nt, 45nt,
..., ..., ...,
49nt
53nt
57nt
61nt

Fig. S2

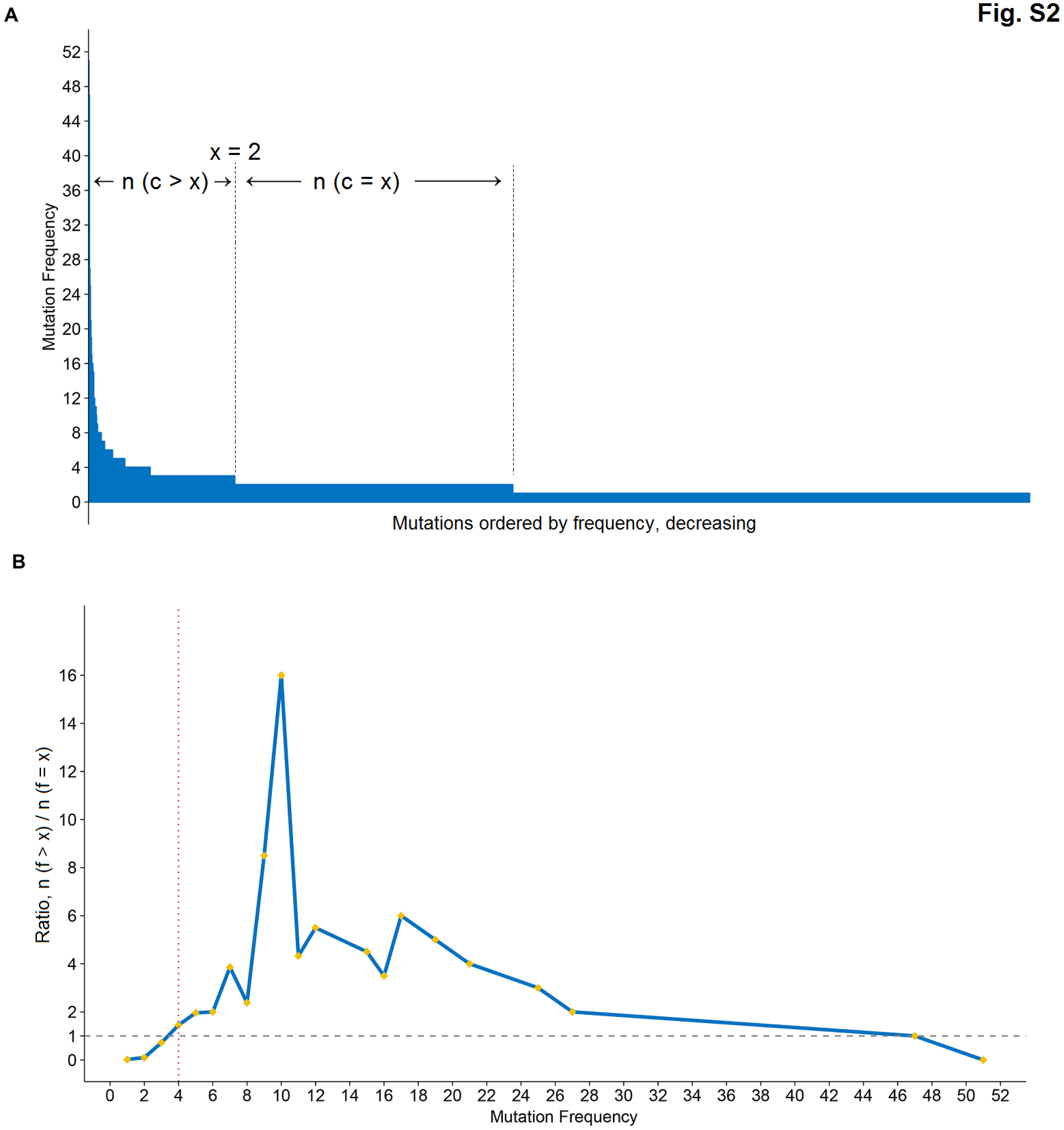
A
Mutation Frequency
52
48
44
40
36
32
28
24
20
16
12
8
4
0
x = 2
← n (c > x) →
← n (c = x) →
Mutations ordered by frequency, decreasing
B
Ratio, n (f > x) / n (f = x)
16
14
12
10
8
6
4
2
1
0
0 2 4 6 8 10 12 14 16 18 20 22 24 26 28 30 32 34 36 38 40 42 44 46 48 50 52
Mutation Frequency

Fig. S3

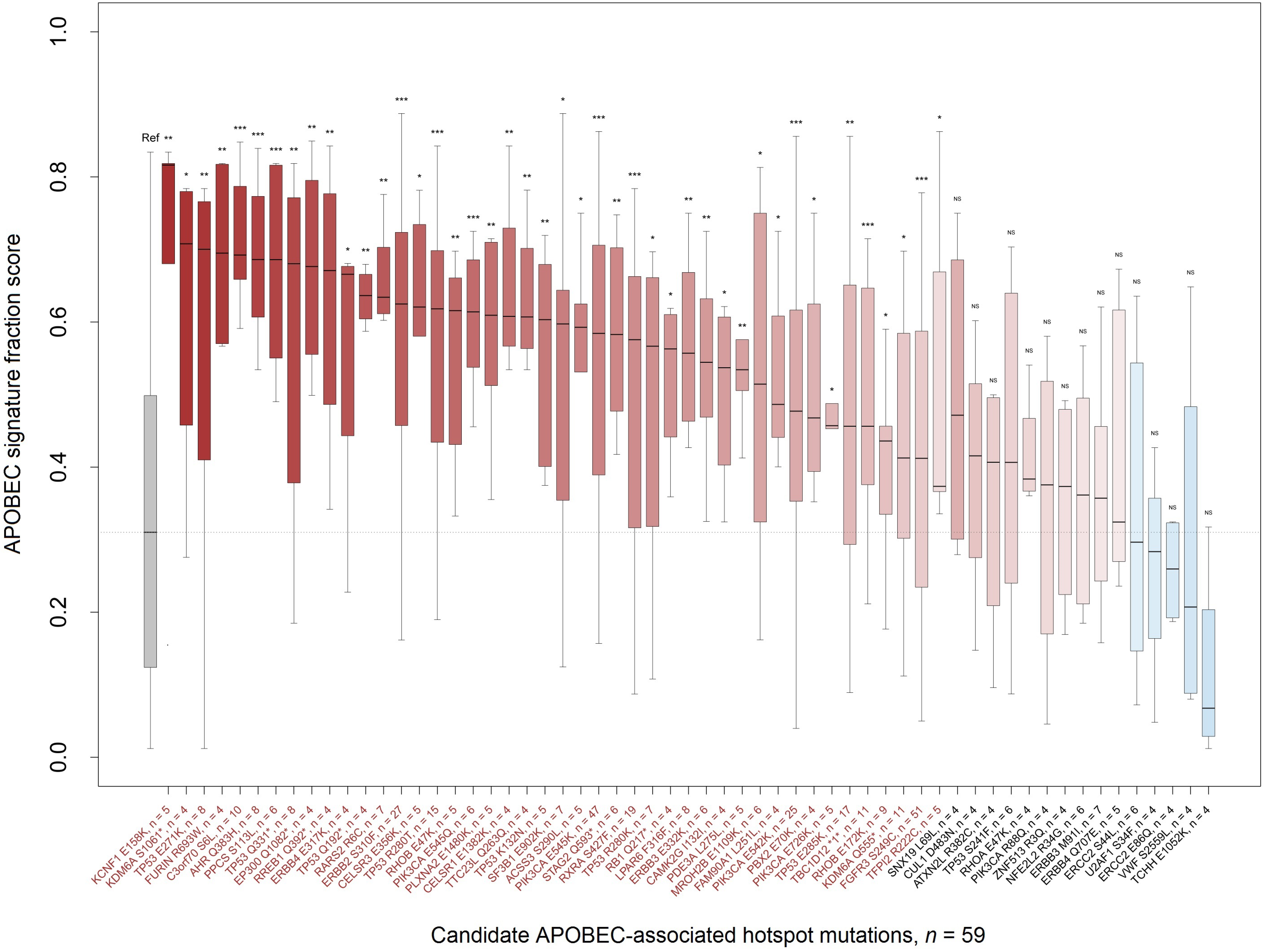
APOBEC signature fraction score
1.0
0.8
0.6
0.4
0.2
0.0
Ref
KCNF1 E158K, n = 5
KDM6A S1061*, n = 4
TP53 E271K, n = 8
FURIN R693W, n = 4
C3orf70 S6L, n = 10
AHR Q383H, n = 8
PPCS S113L, n = 6
TP53 Q331*, n = 8
EP300 Q1082*, n = 4
RREB1 Q392*, n = 4
ERBB4 E317K, n = 4
TP53 Q192*, n = 4
RARS2 R6C, n = 7
ERBB2 S310F, n = 27
CELSR3 E356K, n = 5
TP53 R280T, n = 15
RHOB E47K, n = 5
PIK3CA E545Q, n = 6
PLXNA2 E1480K, n = 5
CELSR1 E1382K, n = 4
TTC23L Q263Q, n = 4
TP53 K132N, n = 5
SF3B1 E902K, n = 7
ACSS3 S290L, n = 5
PIK3CA E545K, n = 47
STAG2 Q593*, n = 6
RXRA S427F, n = 19
TP53 R280K, n = 7
RB1 Q217*, n = 4
LPAR6 F316F, n = 8
ERBB3 E332K, n = 6
CAMK2G I132I, n = 4
PDE3A L275L, n = 5
MROH2B E1109K, n = 6
FAM90A1 L251L, n = 4
PIK3CA E542K, n = 25
PBX2 E70K, n = 4
PIK3CA E726K, n = 5
TP53 E285K, n = 17
TBC1D12 *1*, n = 11
RHOB E172K, n = 9
KDM6A Q555*, n = 11
FGFR3 S249C, n = 51
TFPI2 R222C, n = 5
SNX19 L69L, n = 4
CUL1 D483N, n = 4
ATXN2L R382C, n = 4
TP53 S241F, n = 6
RHOA E47K, n = 4
PIK3CA R88Q, n = 4
ZNF513 R3Q, n = 4
NFE2L2 R34G, n = 6
ERBB3 M91I, n = 7
ERBB4 Q707E, n = 5
ERCC2 S44L, n = 6
U2AF1 S34F, n = 4
ERCC2 E86Q, n = 4
VWF S2559L, n = 4
TCHH E1052K, n = 4
Candidate APOBEC-associated hotspot mutations, n = 59

Fig.S4

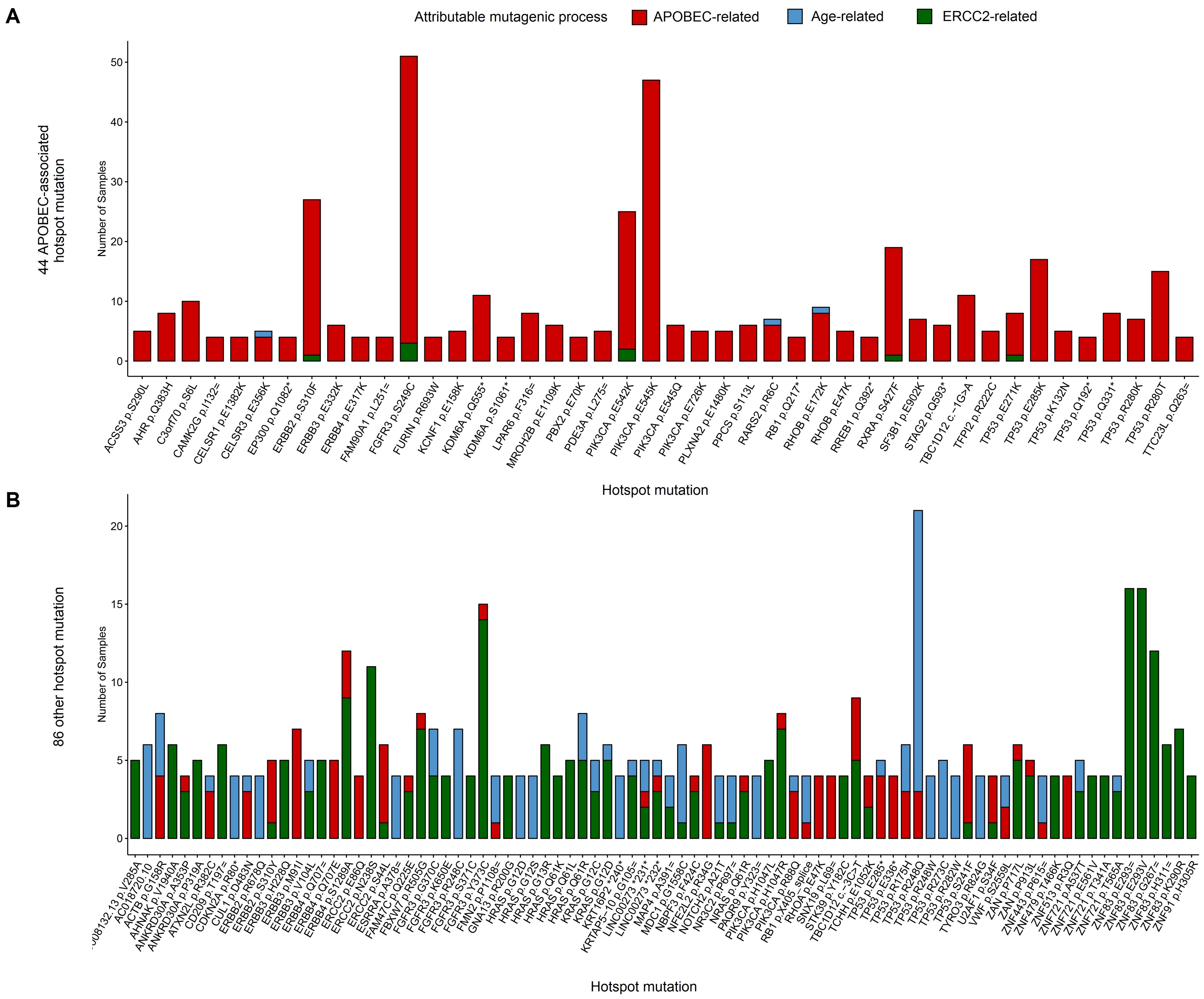
A
Attributable mutagenic process
APOBEC-related
Age-related
ERCC2-related
44 APOBEC-associated hotspot mutation
Number of Samples
0
10
20
30
40
50
ACSS3 p.S290L
AHR p.Q383H
C3orf70 p.S6L
CAMK2G p.I132=
CELSR1 p.E1382K
CELSR3 p.E356K
EP300 p.Q1082*
ERBB2 p.S310F
ERBB3 p.E332K
ERBB4 p.E317K
FAM90A1 p.L251=
FGFR3 p.S249C
FURIN p.R693W
KCNF1 p.E158K
KDM6A p.Q555*
KDM6A p.S1061*
LPAR6 p.F316=
MROH2B p.E1109K
PBX2 p.E70K
PDE3A p.L275=
PIK3CA p.E542K
PIK3CA p.E545K
PIK3CA p.E545Q
PIK3CA p.E726K
PLXNA2 p.E1480K
PPCS p.S113L
RARS2 p.R6C
RB1 p.Q217*
RHOB p.E172K
RHOB p.E47K
RREB1 p.Q392*
RXRA p.S427F
SF3B1 p.E902K
STAG2 p.Q593*
TBC1D12 c.-1G>A
TFPI2 p.R222C
TP53 p.E271K
TP53 p.E285K
TP53 p.K132N
TP53 p.Q192*
TP53 p.Q331*
TP53 p.R280K
TP53 p.R280T
TTC23L p.Q263=
Hotspot mutation
B
86 other hotspot mutation
Number of Samples
0
5
10
15
20
008132.13 p.V285A
AC018720.10
ACTB p.G158R
AHNAK p.V1940A
ANKRD30A p.A353P
ANKRD30A p.P319A
ATXN2L p.R382C
CD209 p.T197=
CDKN2A p.R80*
CUL1 p.D483N
ERBB2 p.R678Q
ERBB2 p.S310Y
ERBB3 p.H228Q
ERBB3 p.M91I
ERBB3 p.V104L
ERBB4 p.Q707=
ERBB4 p.Q707E
ERBB4 p.S1289A
ERCC2 p.E86Q
ERCC2 p.N238S
ERCC2 p.S44L
ESRRA p.A378=
FAM47C p.Q225E
FBXW7 p.R505G
FGFR3 p.G370C
FGFR3 p.K650E
FGFR3 p.R248C
FGFR3 p.S371C
FGFR3 p.Y373C
FMN2 p.P1108=
GNA13 p.R200G
HRAS p.G12D
HRAS p.G12S
HRAS p.G13R
HRAS p.Q61K
HRAS p.Q61L
HRAS p.Q61R
KRAS p.G12C
KRAS p.G12D
KRT16P2 *240*
KRTAP5−10 p.G105=
LINC00273 *231*
LINC00273 *232*
MAP4 p.A391=
MDC1 p.G1558C
NBPF3 p.F424C
NFE2L2 p.R34G
NOTCH2 p.A21T
NR3C2 p.P697=
NRAS p.Q61R
PAQR9 p.Y323=
PIK3CA p.H1047L
PIK3CA p.H1047R
PIK3CA p.R88Q
RB1 p.X405_splice
RHOA p.E47K
SNX19 p.L69=
STK39 p.Y182C
TBC1D12 c.−3C>T
TCHH p.E1052K
TP53 p.E285*
TP53 p.E336*
TP53 p.R175H
TP53 p.R248Q
TP53 p.R248W
TP53 p.R273C
TP53 p.R282W
TP53 p.S241F
TYRO3 p.R824G
U2AF1 p.S34F
VWF p.S2559L
ZAN p.P717L
ZAN p.P913L
ZNF443 p.P615=
ZNF479 p.T466K
ZNF513 p.R3Q
ZNF721 p.A537T
ZNF721 p.E561V
ZNF721 p.T341A
ZNF721 p.T565A
ZNF83 p.E293=
ZNF83 p.E293V
ZNF83 p.G267=
ZNF83 p.H311=
ZNF83 p.K290R
ZNF91 p.H305R
Hotspot mutation

Fig.S5

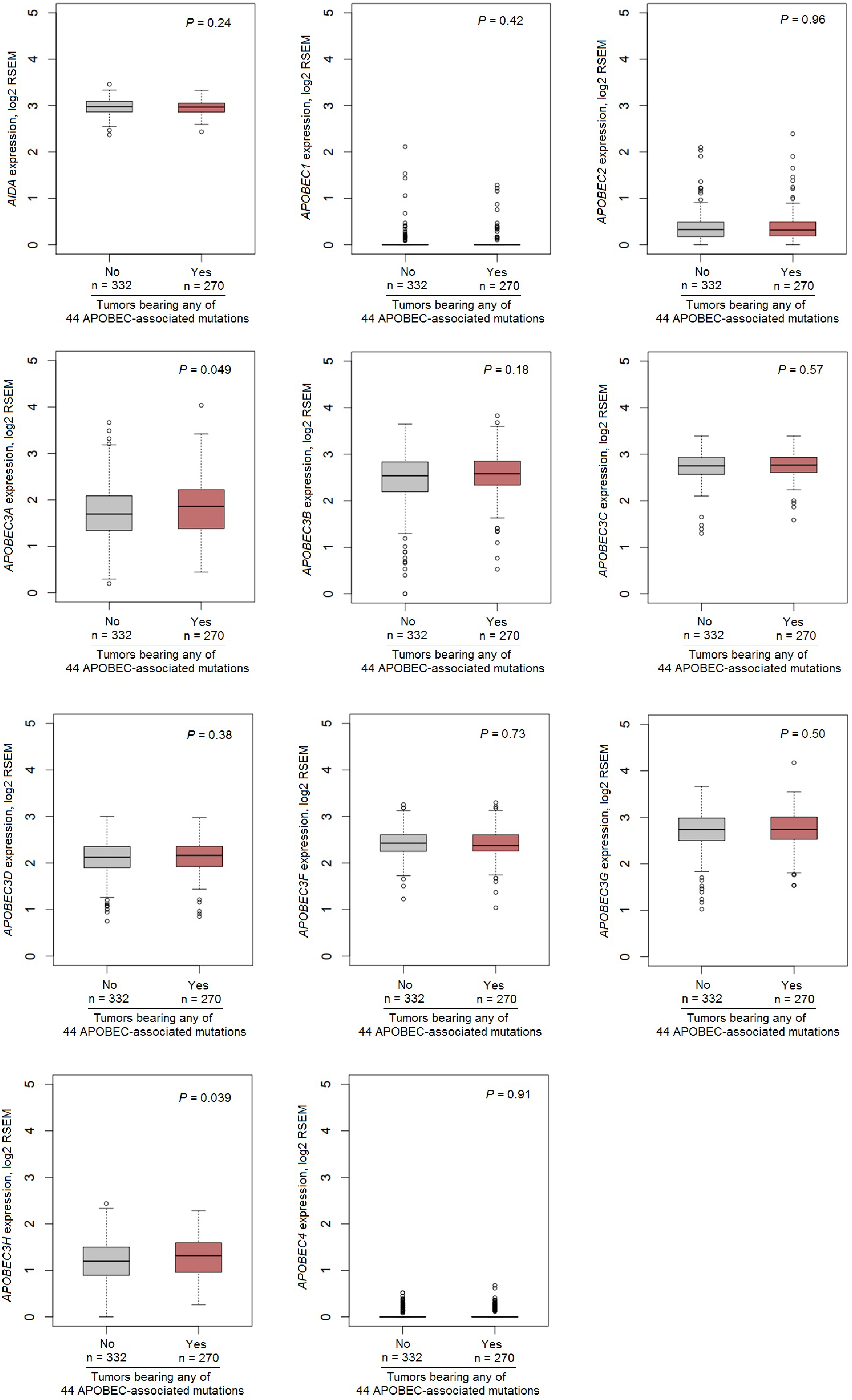

AIDA expression, log2 RSEM
P = 0.24
APOBEC1 expression, log2 RSEM
P = 0.42
APOBEC2 expression, log2 RSEM
P = 0.96
APOBEC3A expression, log2 RSEM
P = 0.049
APOBEC3B expression, log2 RSEM
P = 0.18
APOBEC3C expression, log2 RSEM
P = 0.57
APOBEC3D expression, log2 RSEM
P = 0.38
APOBEC3F expression, log2 RSEM
P = 0.73
APOBEC3G expression, log2 RSEM
P = 0.50
APOBEC3H expression, log2 RSEM
P = 0.039
APOBEC4 expression, log2 RSEM
P = 0.91
0 1 2 3 4 5
No
n = 332
Yes
n = 270
Tumors bearing any of
44 APOBEC-associated mutations

Fig. S6

(Including 43 subfigures)

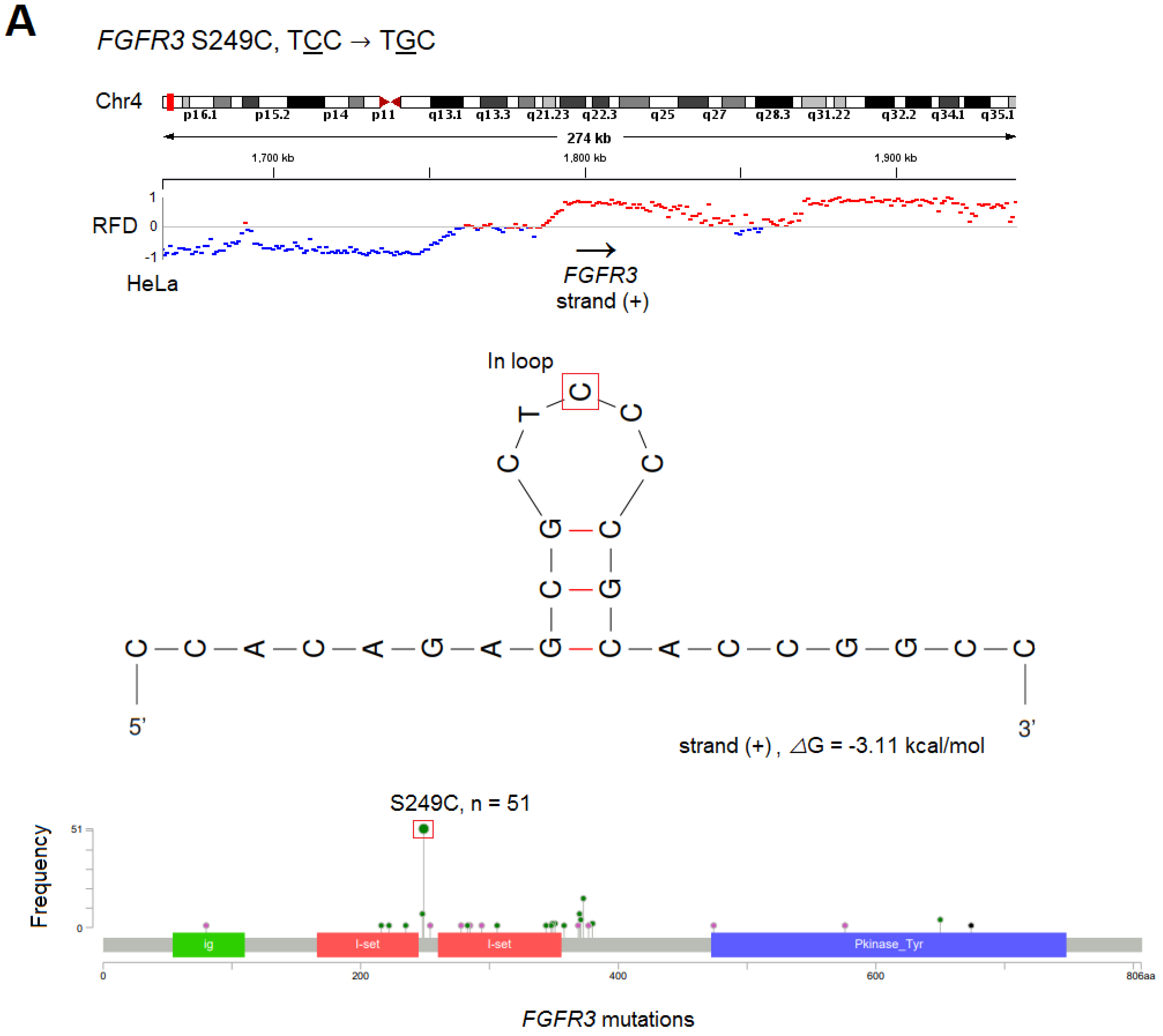
A
FGFR3 S249C, TCC → TGC
Chr4
p16.1
p15.2
p14
p11
q13.1
q13.3
q21.23
q22.3
q25
q27
q28.3
q31.22
q32.2
q34.1
q35.1
274 kb
1,700 kb
1,800 kb
1,900 kb
RFD
1
0
-1
HeLa
FGFR3
strand (+)
In loop
5'
3'
strand (+) , ΔG = -3.11 kcal/mol
S249C, n = 51
51
0
Frequency
ig
I-set
I-set
Pkinase_Tyr
0
200
400
600
806aa
FGFR3 mutations

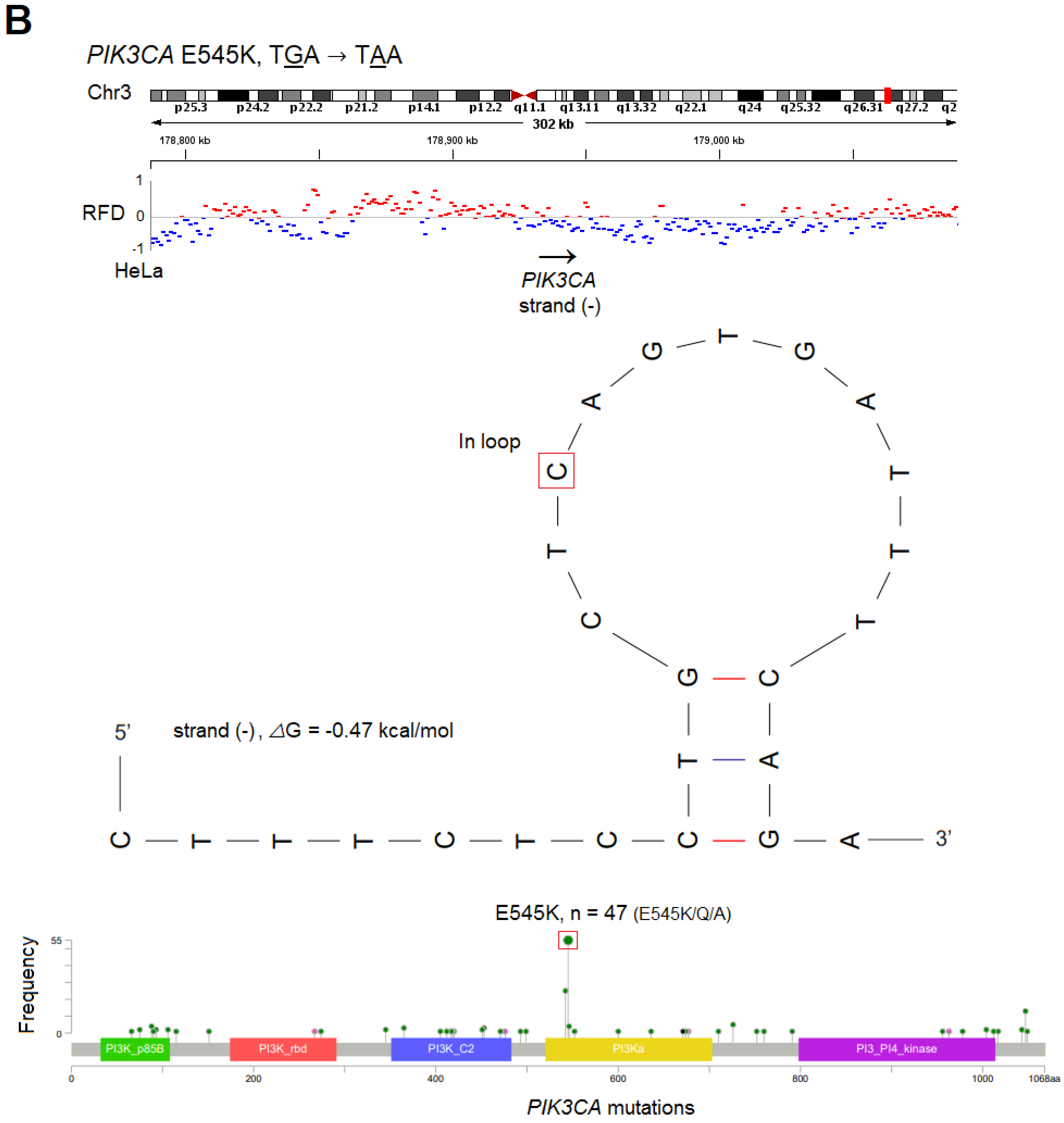
B
PIK3CA E545K, TGA → TAA
Chr3
p25.3 p24.2 p22.2 p21.2 p14.1 p12.2 q11.1 q13.11 q13.32 q22.1 q24 q25.32 q26.31 q27.2 q2
302 kb
178,800 kb
178,900 kb
179,000 kb
RFD
1
0
-1
HeLa
PIK3CA
strand (-)
In loop
5'
strand (-), ΔG = -0.47 kcal/mol
3'
E545K, n = 47 (E545K/Q/A)
Frequency
55
0
PI3K_p85B
PI3K_rbd
PI3K_C2
PI3Ka
PI3_PI4_kinase
0
200
400
600
800
1000
1068aa
PIK3CA mutations

C

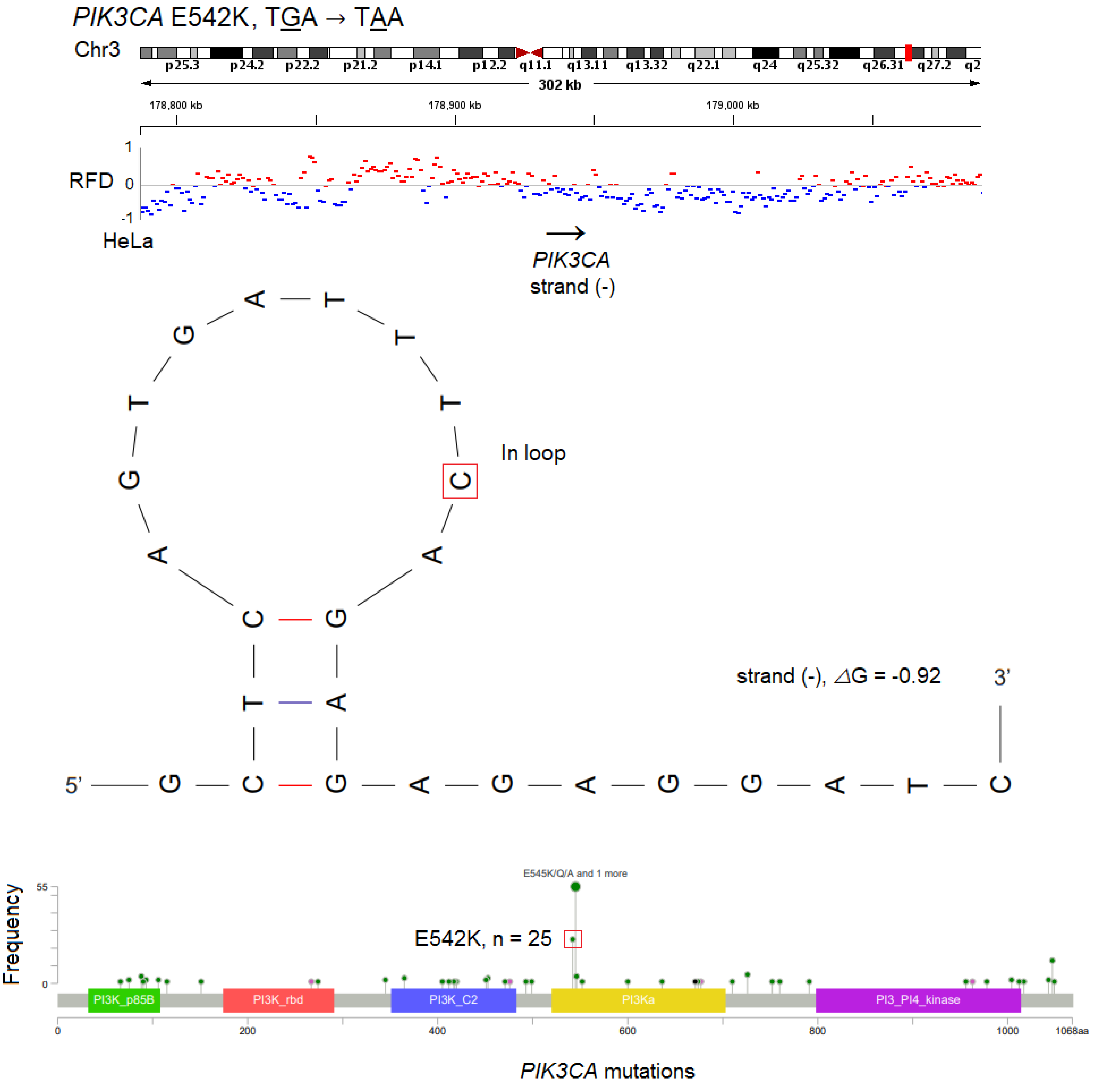

PIK3CA E542K, TGA → TAA
Chr3
p25.3 p24.2 p22.2 p21.2 p14.1 p12.2 q11.1 q13.11 q13.32 q22.1 q24 q25.32 q26.31 q27.2 q2
302 kb
178,800 kb
178,900 kb
179,000 kb
RFD
1
0
-1
HeLa
PIK3CA
strand (-)
In loop
strand (-), ⊿G = -0.92
5'
3'
E545K/Q/A and 1 more
55
0
Frequency
E542K, n = 25
PI3K_p85B
PI3K_rbd
PI3K_C2
PI3Ka
PI3_PI4_kinase
0
200
400
600
800
1000
1068aa
PIK3CA mutations

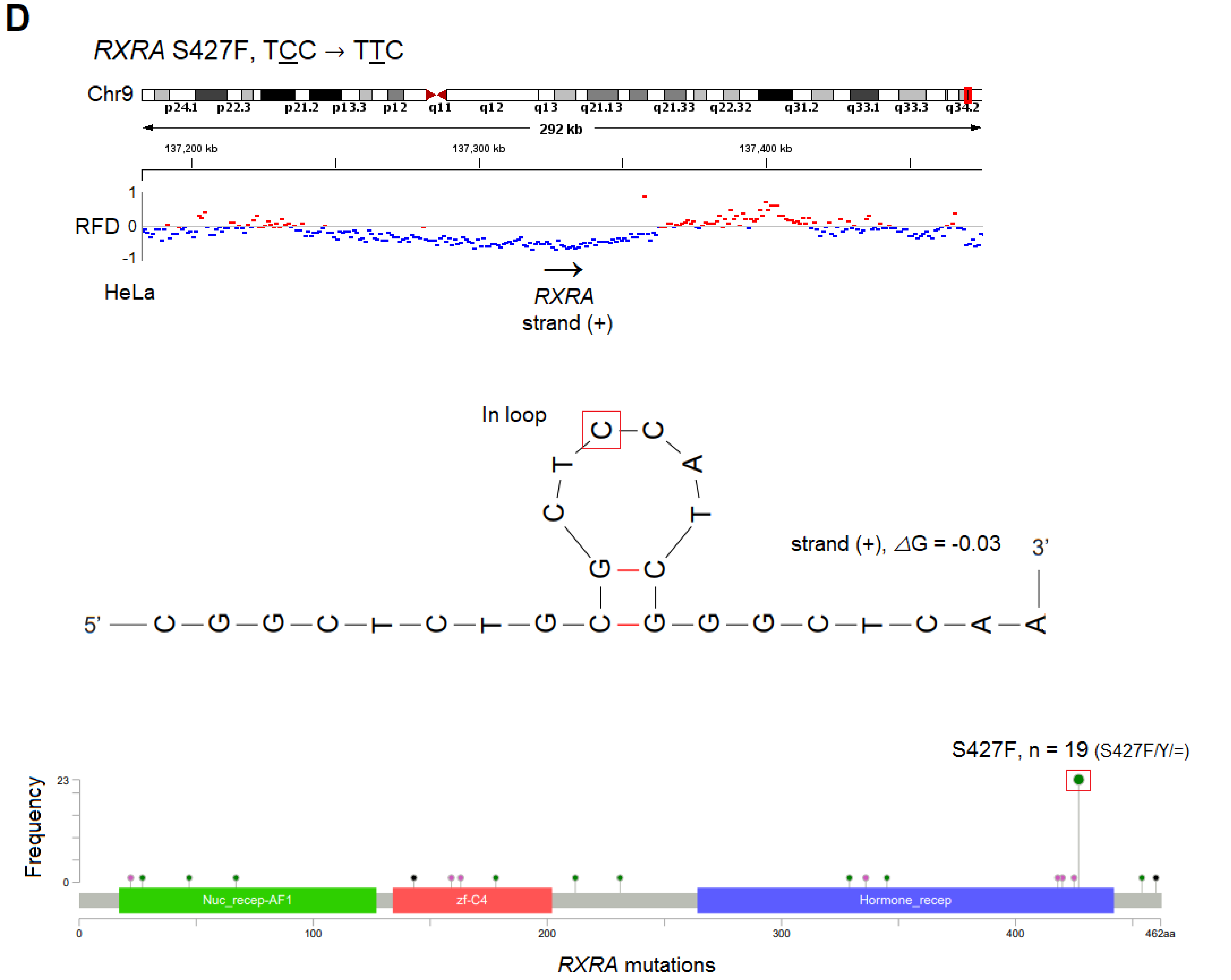
D
RXRA S427F, TCC → TTC
Chr9
p24.1 p22.3 p21.2 p13.3 p12 q11 q12 q13 q21.13 q21.33 q22.32 q31.2 q33.1 q33.3 q34.2
292 kb
137,200 kb
137,300 kb
137,400 kb
RFD
1
0
-1
HeLa
RXRA
strand (+)
In loop
strand (+), ΔG = -0.03
5'
3'
S427F, n = 19 (S427F/Y/=)
Frequency
23
0
Nuc_recep-AF1
zf-C4
Hormone_recep
0
100
200
300
400
462aa
RXRA mutations

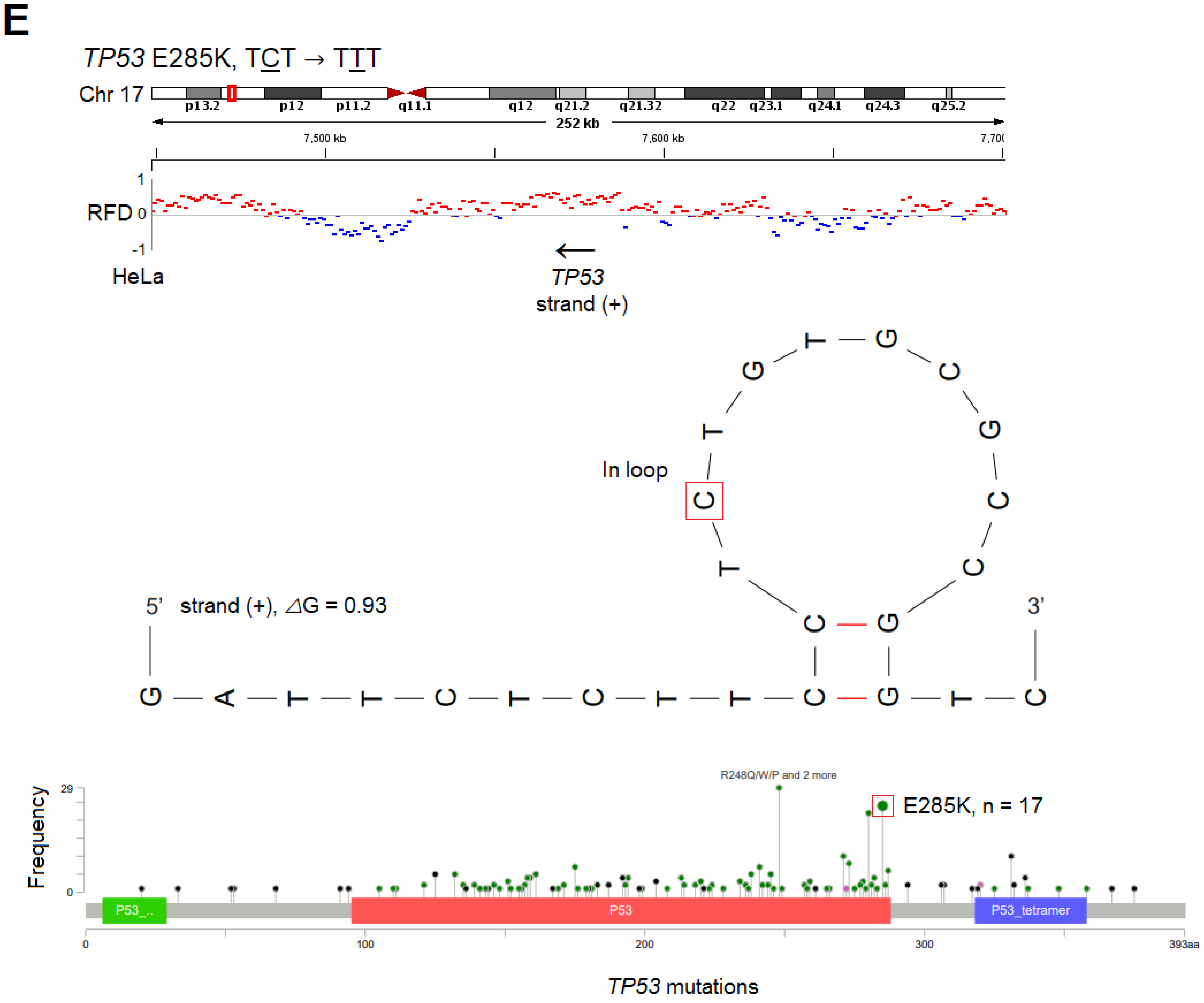
E
TP53 E285K, TCT → TTT
Chr 17
p13.2
p12
p11.2
q11.1
q12
q21.2
q21.32
q22
q23.1
q24.1
q24.3
q25.2
252 kb
7,500 kb
7,600 kb
RFD
1
0
-1
HeLa
TP53
strand (+)
In loop
5'
strand (+), ΔG = 0.93
3'
G A T T C T C T T C G T C
R248Q/W/P and 2 more
E285K, n = 17
Frequency
29
0
P53_..
P53
P53_tetramer
0
100
200
300
393aa
TP53 mutations

F

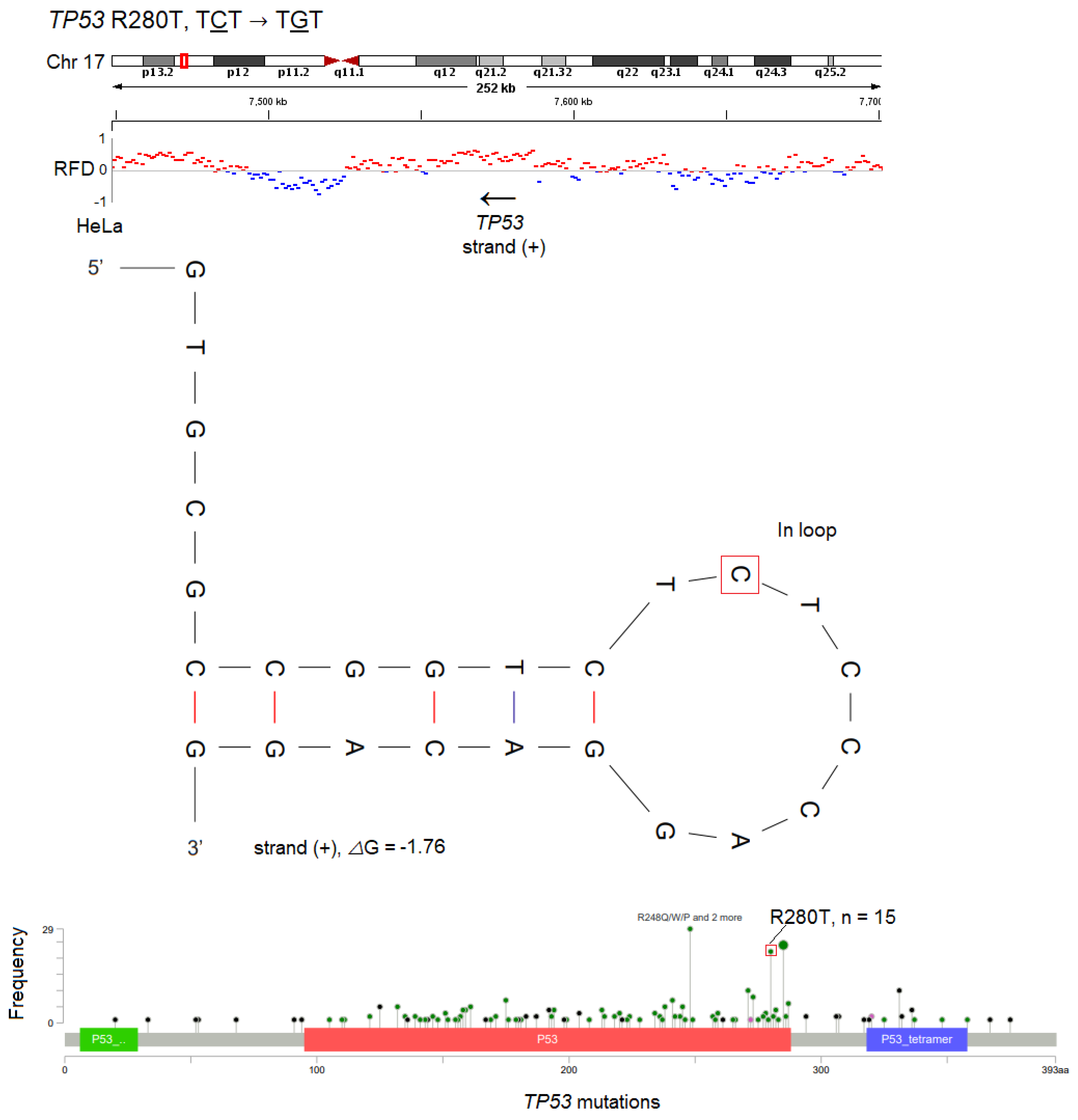

TP53 R280T, TCT → TGT
Chr 17
p13.2
p12
p11.2
q11.1
q12
q21.2
q21.32
q22
q23.1
q24.1
q24.3
q25.2
252 kb
7,500 kb
7,600 kb
RFD
1
0
-1
HeLa
TP53
strand (+)
5'
3'
In loop
strand (+), ⊿G = -1.76
R248Q/W/P and 2 more
R280T, n = 15
29
0
Frequency
P53_..
P53
P53_tetramer
0
100
200
300
393aa
TP53 mutations

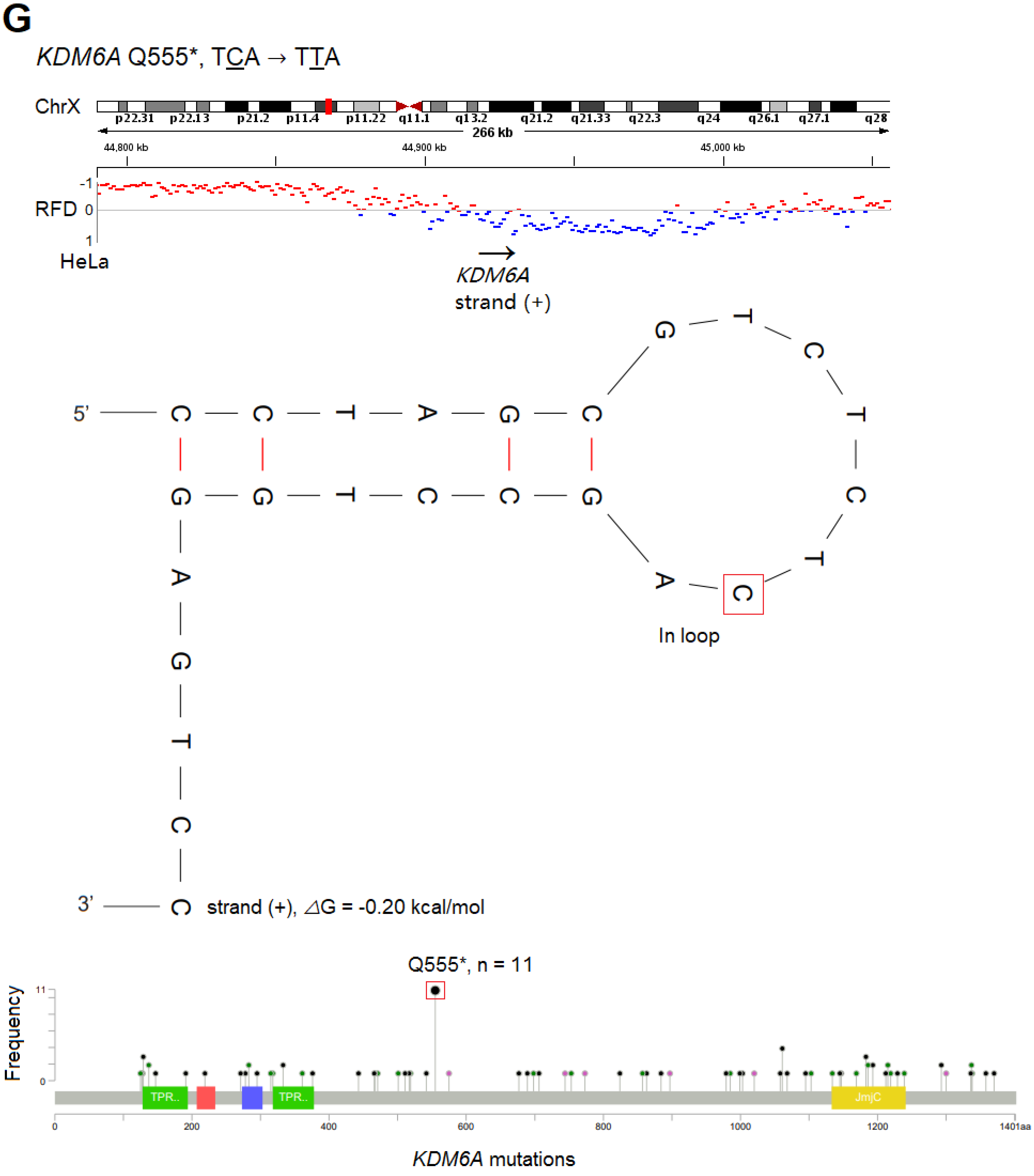
G
KDM6A Q555*, TCA → TTA
ChrX
p22.31 p22.13 p21.2 p11.4 p11.22 q11.1 q13.2 q21.2 q21.33 q22.3 q24 q26.1 q27.1 q28
266 kb
44,800 kb
44,900 kb
45,000 kb
RFD
-1
0
1
HeLa
KDM6A
strand (+)
5'
3'
In loop
strand (+), ΔG = -0.20 kcal/mol
Q555*, n = 11
Frequency
TPR..
TPR..
JmjC
KDM6A mutations

H

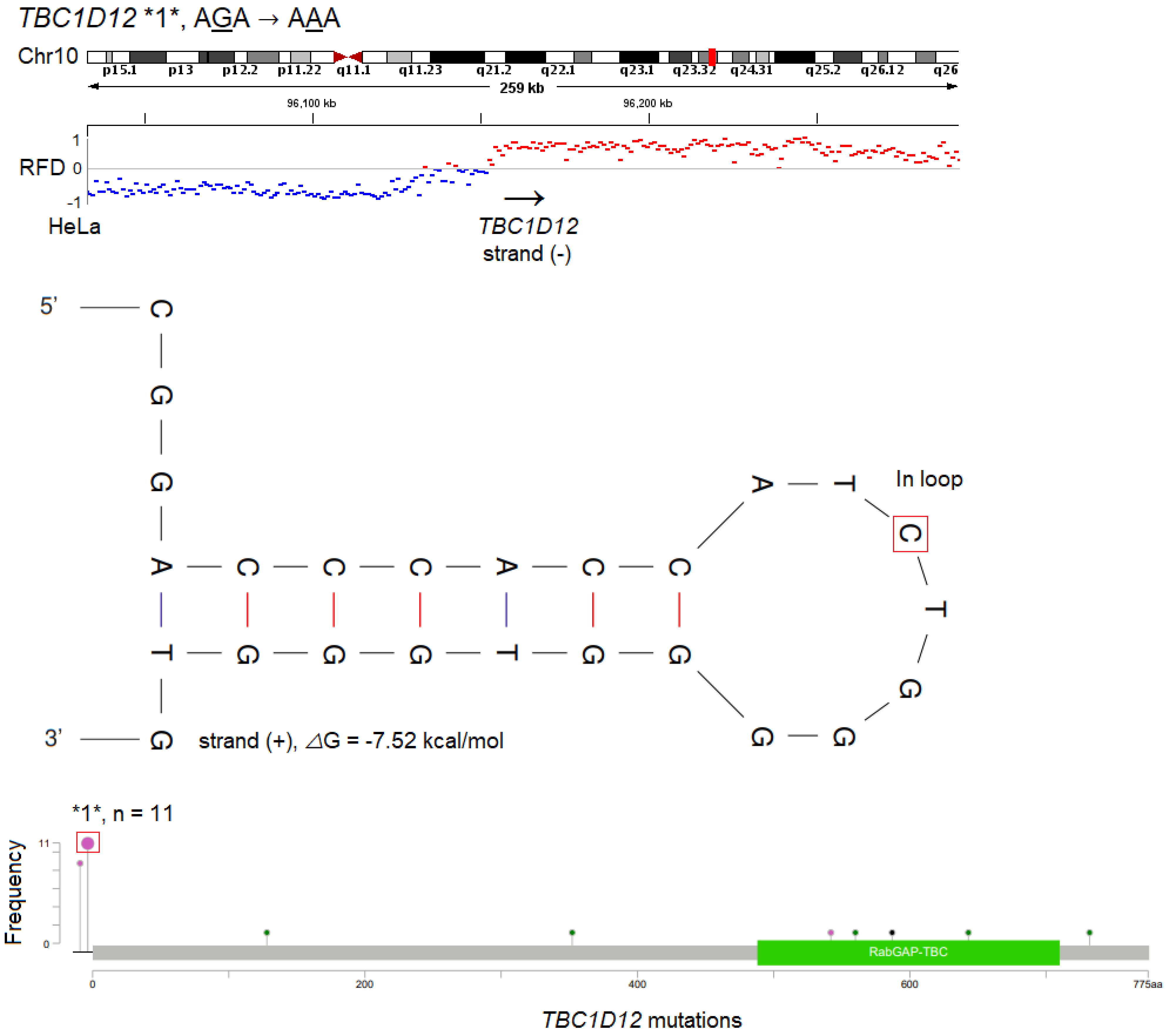

TBC1D12 *1*, AGA → AAA
Chr10
p15.1
p13
p12.2
p11.22
q11.1
q11.23
q21.2
q22.1
q23.1
q23.32
q24.31
q25.2
q26.12
q26
259 kb
96,100 kb
96,200 kb
RFD
1
0
-1
HeLa
TBC1D12
strand (-)
5'
3'
In loop
strand (+), ⊿G = -7.52 kcal/mol
1, n = 11
Frequency
11
0
RabGAP-TBC
0
200
400
600
775aa
TBC1D12 mutations

I

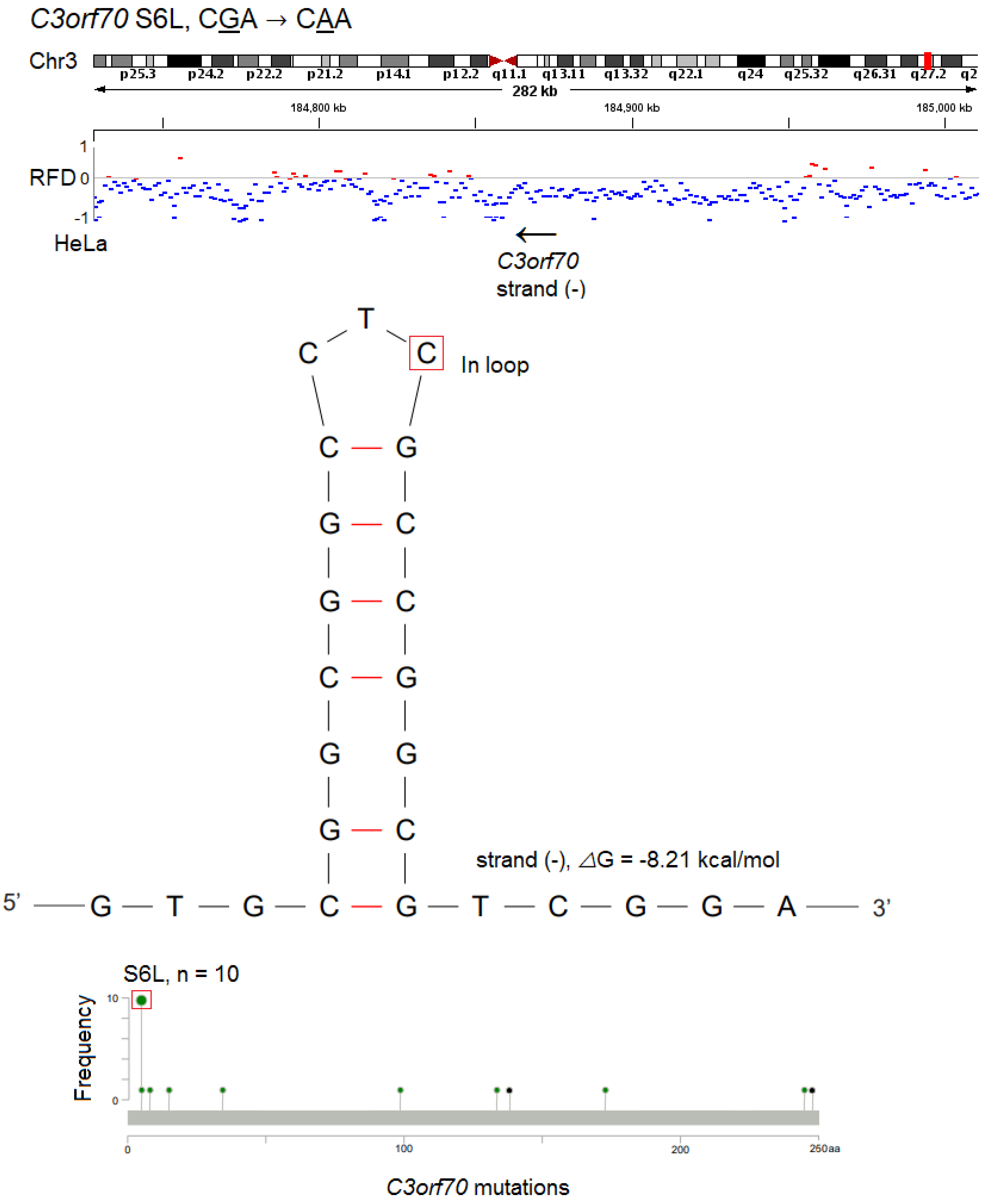
C3orf70 S6L, CGA → CAA
Chr3
p25.3
p24.2
p22.2
p21.2
p14.1
p12.2
q11.1
q13.11
q13.32
q22.1
q24
q25.32
q26.31
q27.2
282 kb
184,800 kb
184,900 kb
185,000 kb
RFD
1
0
-1
HeLa
C3orf70
strand (-)
In loop
strand (-), ⊿G = -8.21 kcal/mol
5'
G T G C G T C G G A
3'
S6L, n = 10
Frequency
10
0
100
200
260aa
C3orf70 mutations

J

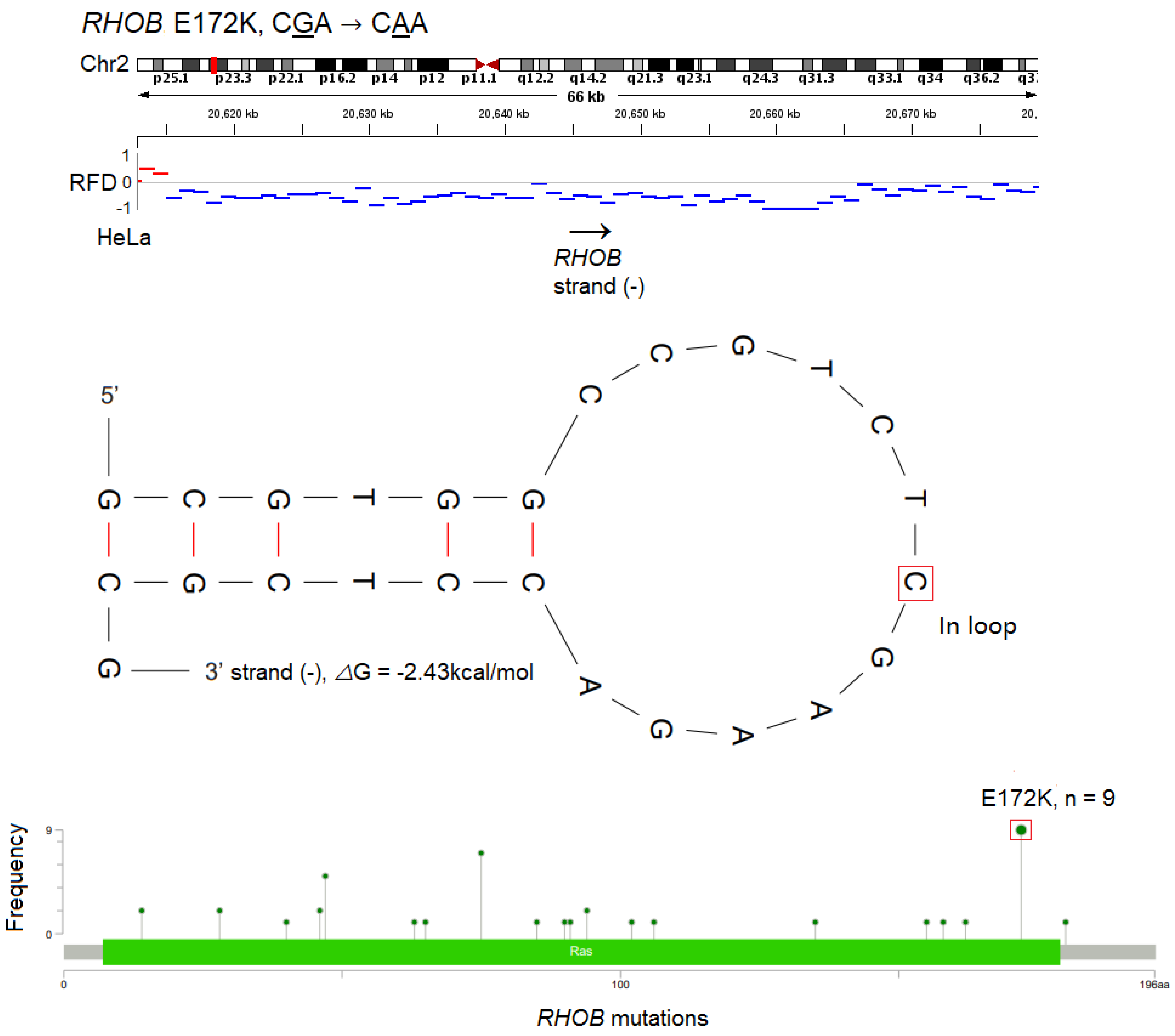

RHOB E172K, CGA → CAA
Chr2
p25.1
p23.3
p22.1
p16.2
p14
p12
p11.1
q12.2
q14.2
q21.3
q23.1
q24.3
q31.3
q33.1
q34
q36.2
66 kb
20,620 kb
20,630 kb
20,640 kb
20,650 kb
20,660 kb
20,670 kb
RFD
1
0
-1
HeLa
RHOB
strand (-)
5'
3' strand (-), ΔG = -2.43kcal/mol
In loop
E172K, n = 9
Frequency
9
0
Ras
0
100
196aa
RHOB mutations

K

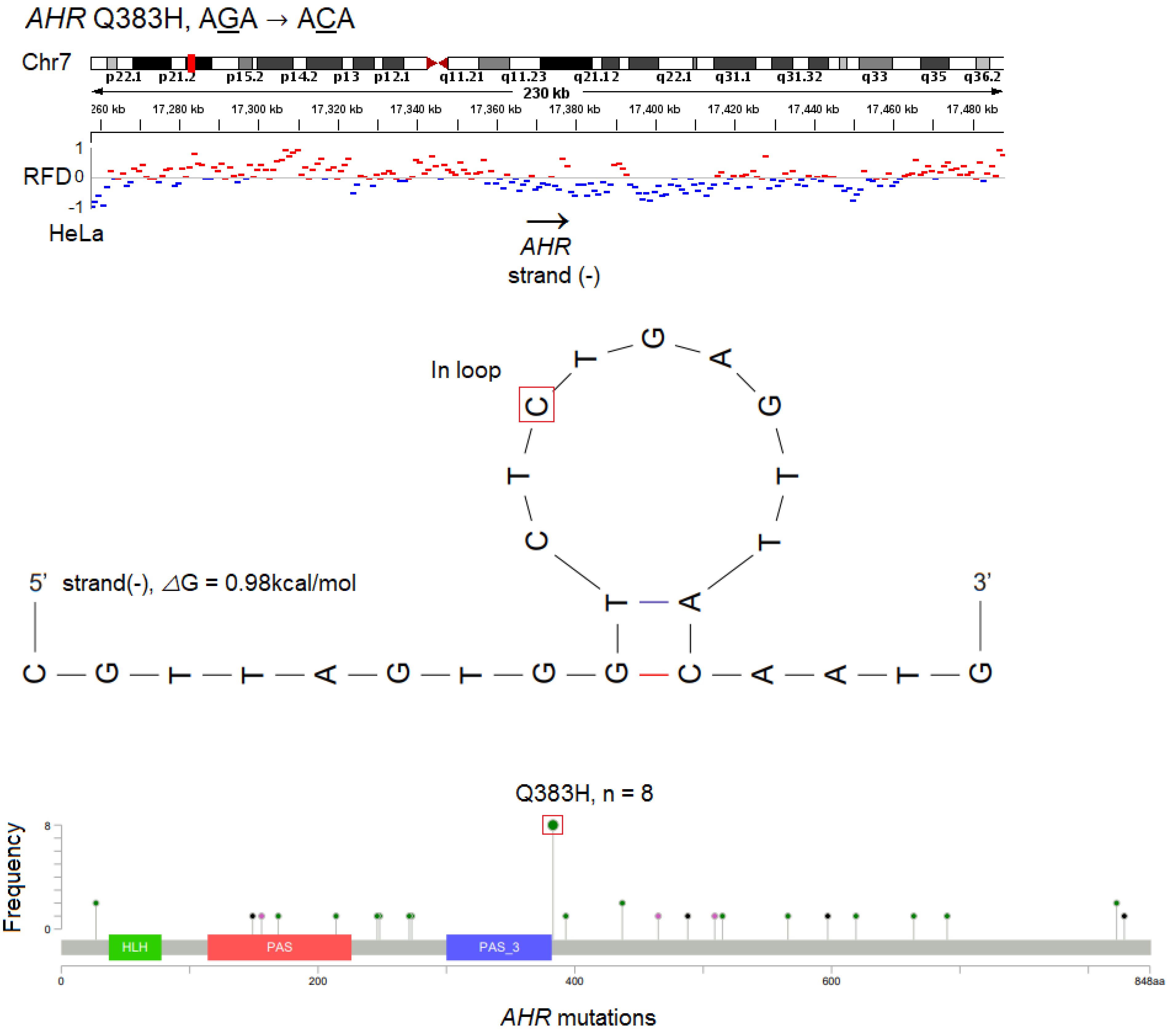
AHR Q383H, AGA → ACA
Chr7
p22.1 p21.2 p15.2 p14.2 p13 p12.1 q11.21 q11.23 q21.12 q22.1 q31.1 q31.32 q33 q35 q36.2
230 kb
17,280 kb 17,300 kb 17,320 kb 17,340 kb 17,360 kb 17,380 kb 17,400 kb 17,420 kb 17,440 kb 17,460 kb 17,480 kb
RFD
1
0
-1
HeLa
AHR
strand (-)
In loop
5'
strand(-), ΔG = 0.98kcal/mol
3'
C G T T A G T G G C A A T G
T C T C T G A G T T T A
Q383H, n = 8
Frequency
8
0
HLH
PAS
PAS_3
0
200
400
600
848aa
AHR mutations

L

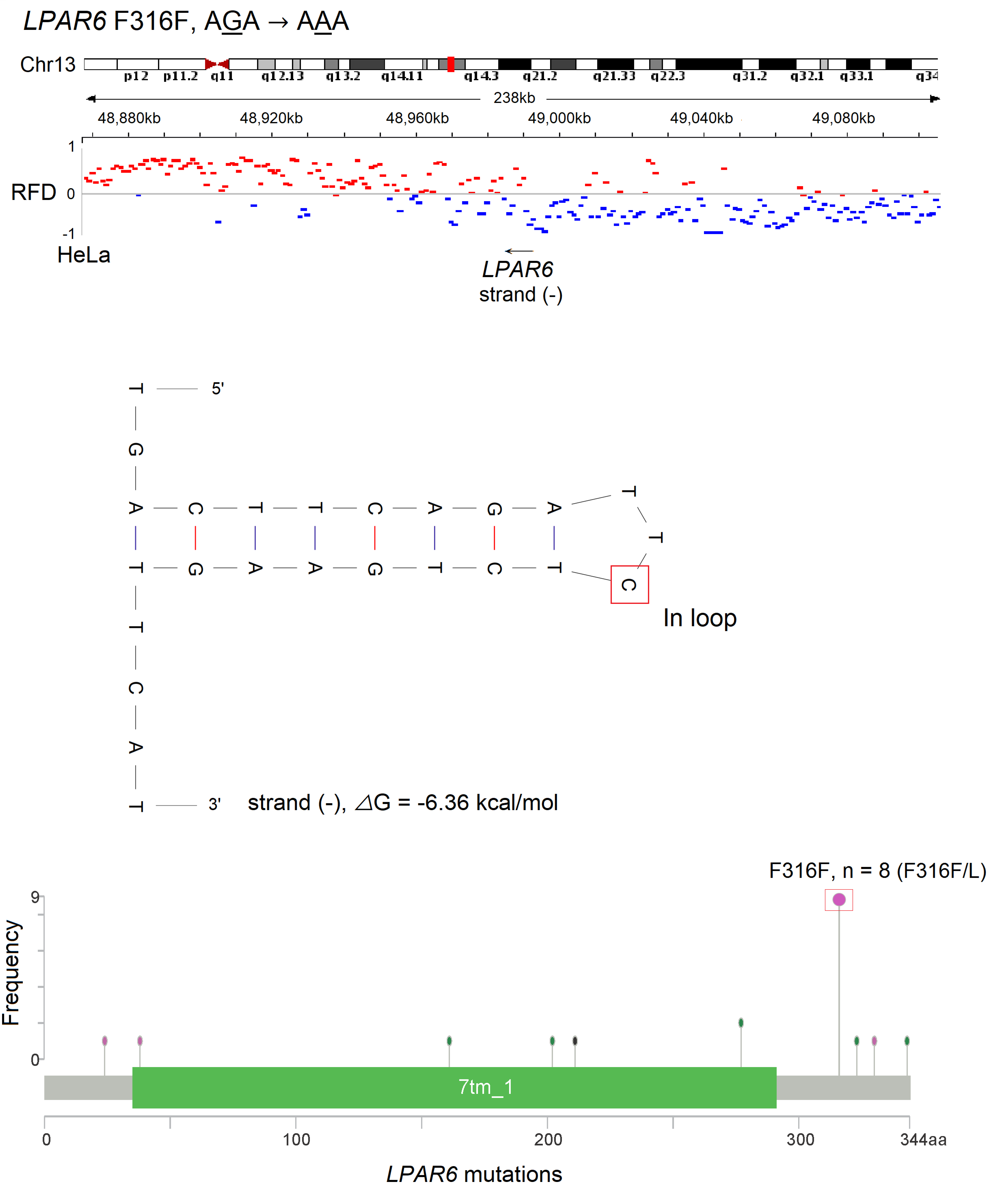
LPAR6 F316F, AGA → AAA
Chr13
p12 p11.2 q11 q12.13 q13.2 q14.11 q14.3 q21.2 q21.33 q22.3 q31.2 q32.1 q33.1 q34
238kb
48,880kb 48,920kb 48,960kb 49,000kb 49,040kb 49,080kb
RFD
1
0
-1
HeLa
LPAR6
strand (-)
5'
3'
In loop
strand (-), ΔG = -6.36 kcal/mol
F316F, n = 8 (F316F/L)
Frequency
9
0
7tm_1
0
100
200
300
344aa
LPAR6 mutations

M

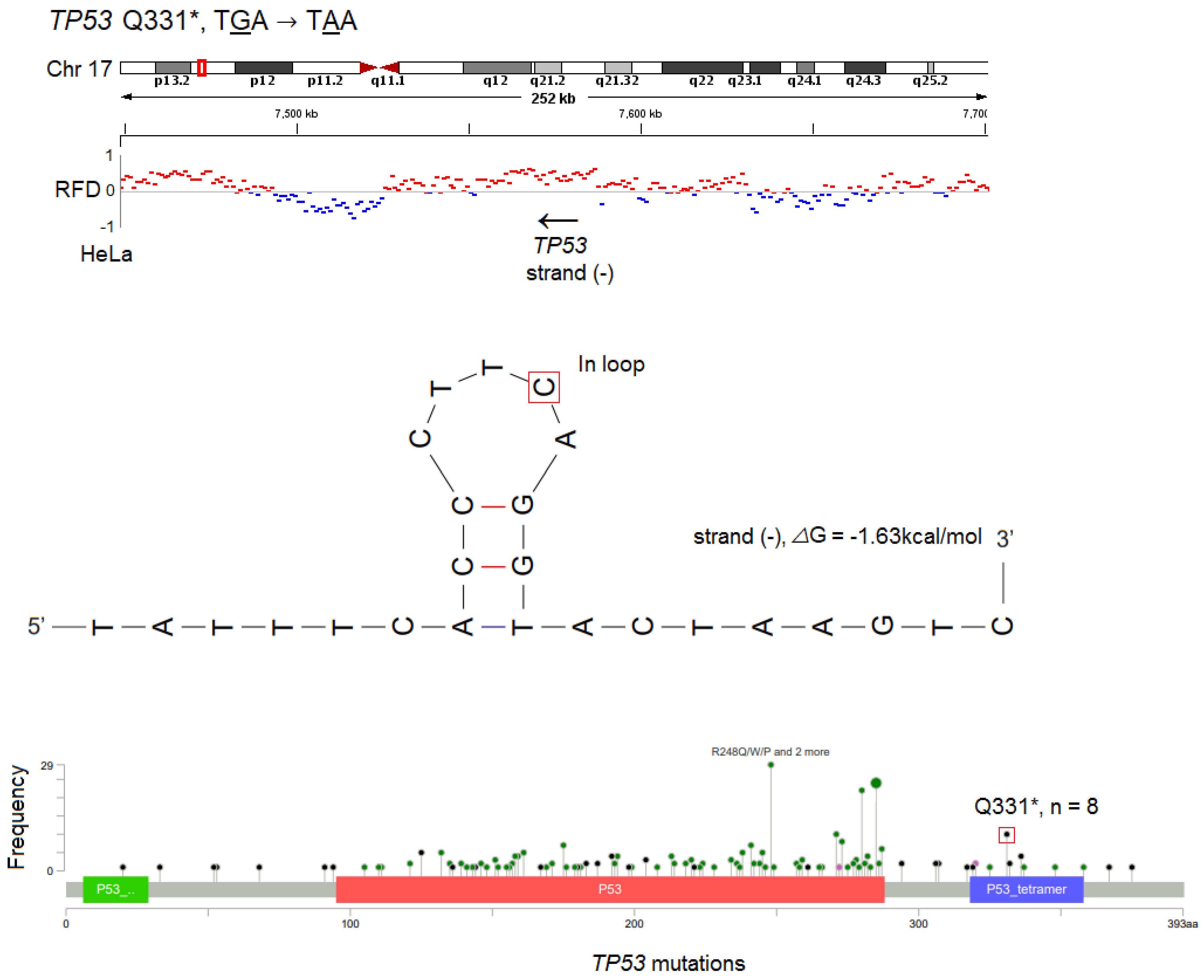

TP53 Q331*, TGA → TAA
Chr 17
p13.2
p12
p11.2
q11.1
q12
q21.2
q21.32
q22
q23.1
q24.1
q24.3
q25.2
252 kb
7,500 kb
7,600 kb
RFD
1
0
-1
HeLa
TP53
strand (-)
In loop
strand (-), ⊿G = -1.63kcal/mol
3'
5'
R248Q/W/P and 2 more
Q331*, n = 8
Frequency
29
0
P53_...
P53
P53_tetramer
0
100
200
300
393aa
TP53 mutations

N

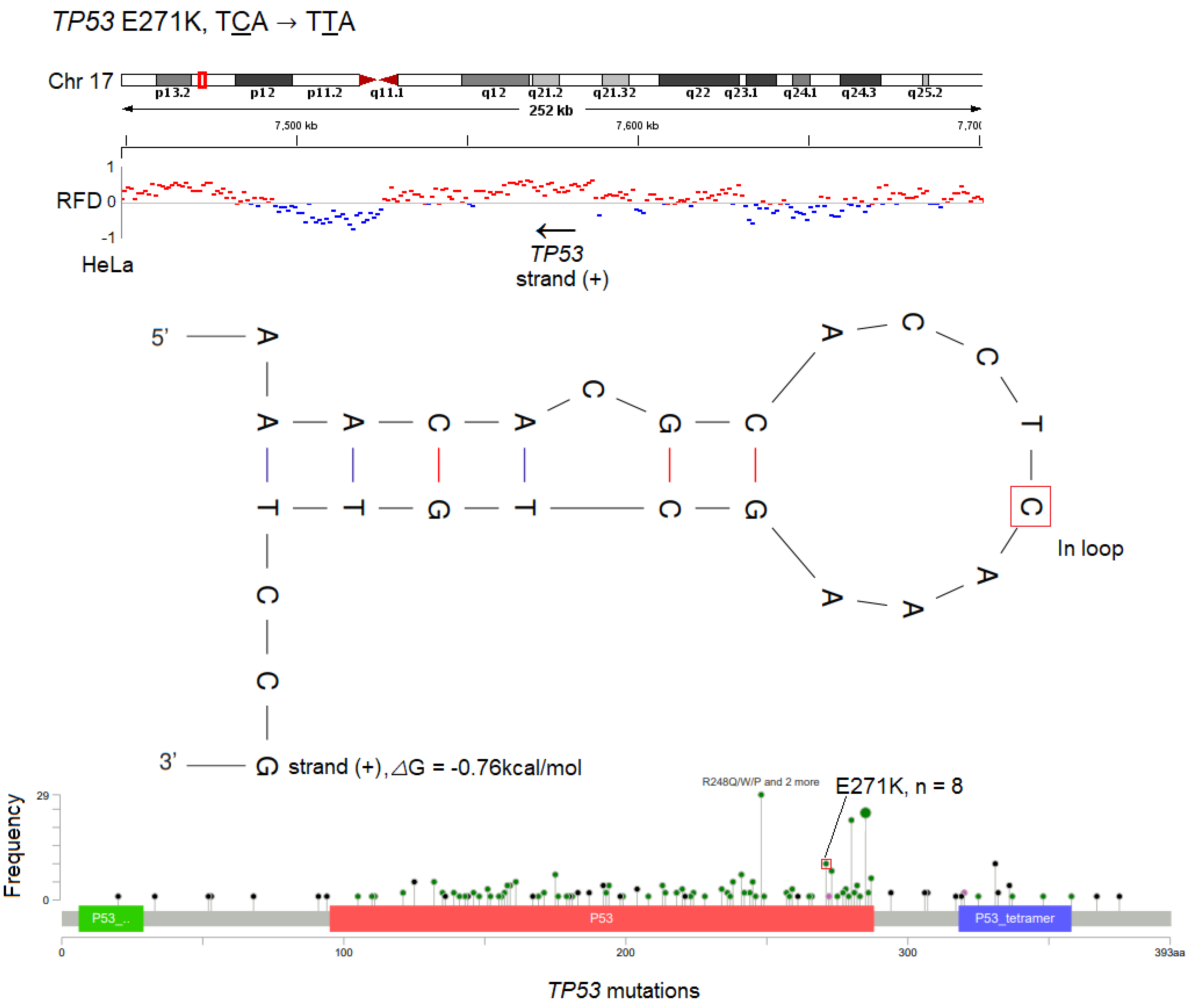
TP53 E271K, TCA → TTA
Chr 17
p13.2
p12
p11.2
q11.1
q12
q21.2
q21.32
q22
q23.1
q24.1
q24.3
q25.2
252 kb
7,500 kb
7,600 kb
RFD
HeLa
TP53
strand (+)
5'
3'
In loop
strand (+), ΔG = -0.76kcal/mol
R248Q/W/P and 2 more
E271K, n = 8
Frequency
29
0
P53_..
P53
P53_tetramer
100
200
300
393aa
TP53 mutations

O

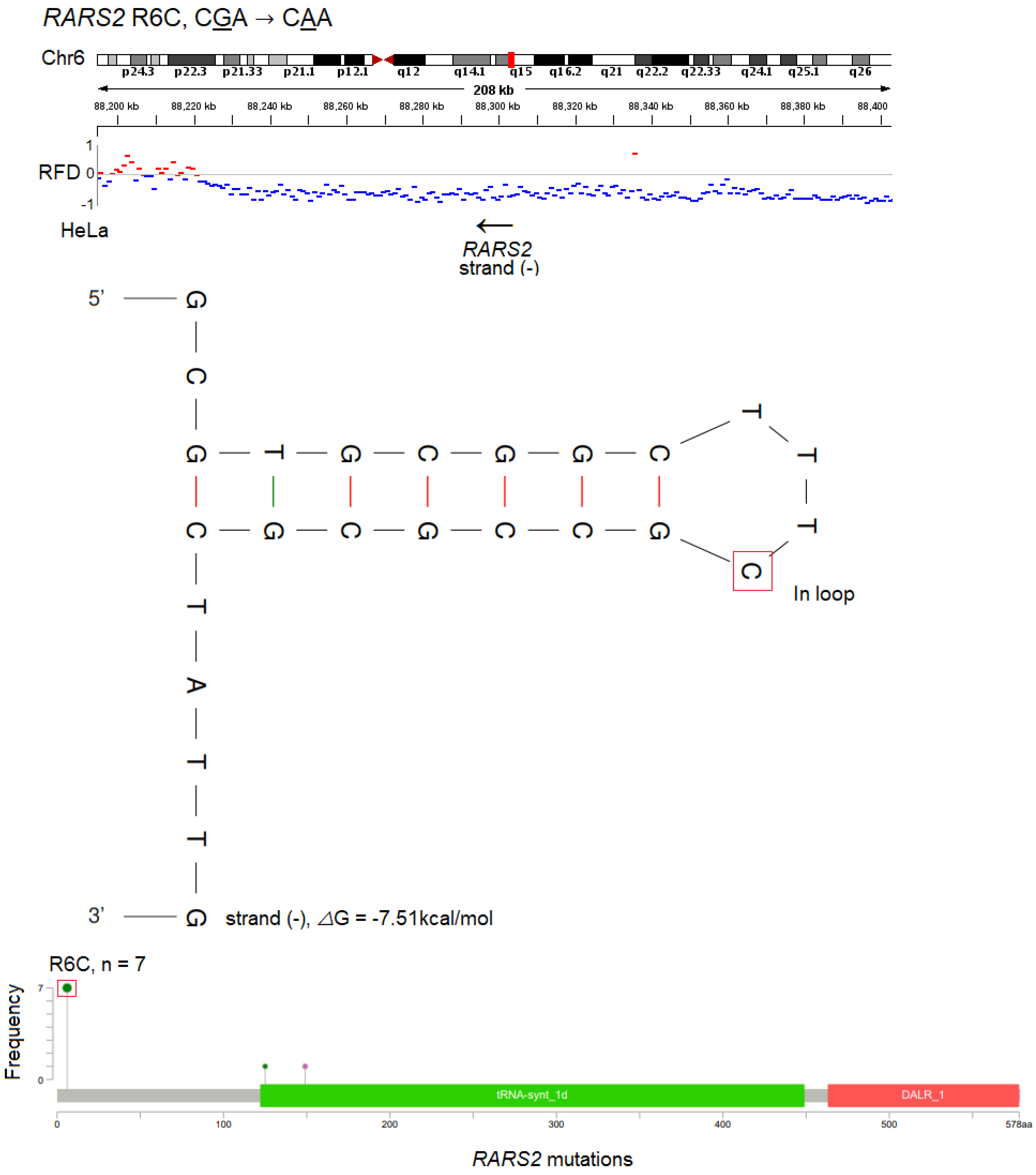

RARS2 R6C, CGA → CAA
Chr6
p24.3 p22.3 p21.33 p21.1 p12.1 q12 q14.1 q15 q16.2 q21 q22.2 q22.33 q24.1 q25.1 q26
208 kb
88,200 kb 88,220 kb 88,240 kb 88,260 kb 88,280 kb 88,300 kb 88,320 kb 88,340 kb 88,360 kb 88,380 kb 88,400
RFD
1
0
-1
HeLa
RARS2
strand (-)
5'
3'
In loop
strand (-), ⊿G = -7.51kcal/mol
R6C, n = 7
Frequency
tRNA-synt_1d
DALR_1
0 100 200 300 400 500 578aa
RARS2 mutations

P

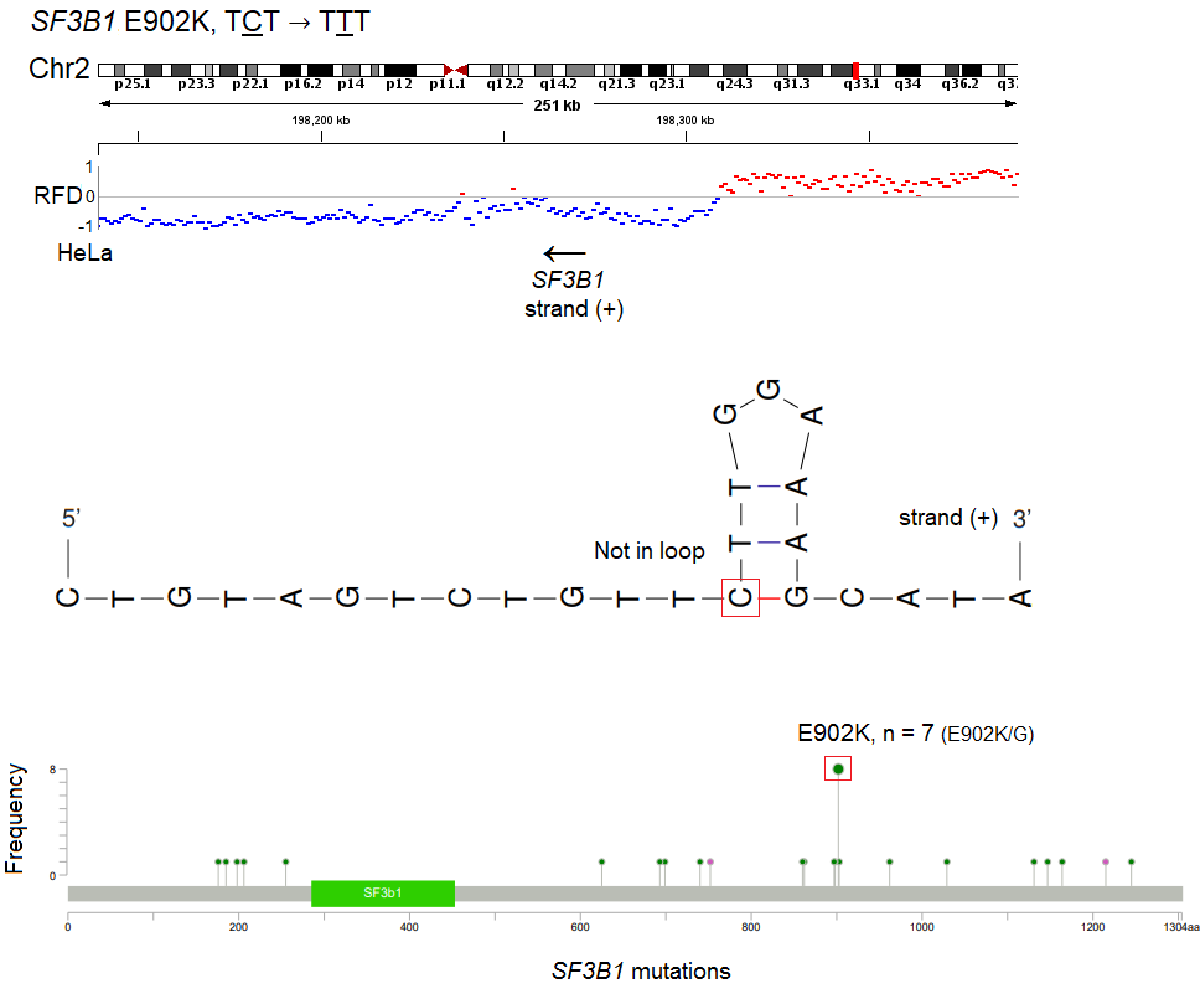

SF3B1 E902K, TCT → TTT
Chr2
p25.1 p23.3 p22.1 p16.2 p14 p12 p11.1 q12.2 q14.2 q21.3 q23.1 q24.3 q31.3 q33.1 q34 q36.2
251 kb
198,200 kb
198,300 kb
RFD
1
0
-1
HeLa
SF3B1
strand (+)
5'
Not in loop
strand (+)
3'
E902K, n = 7 (E902K/G)
Frequency
8
0
SF3b1
0
200
400
600
800
1000
1200
1304aa
SF3B1 mutations

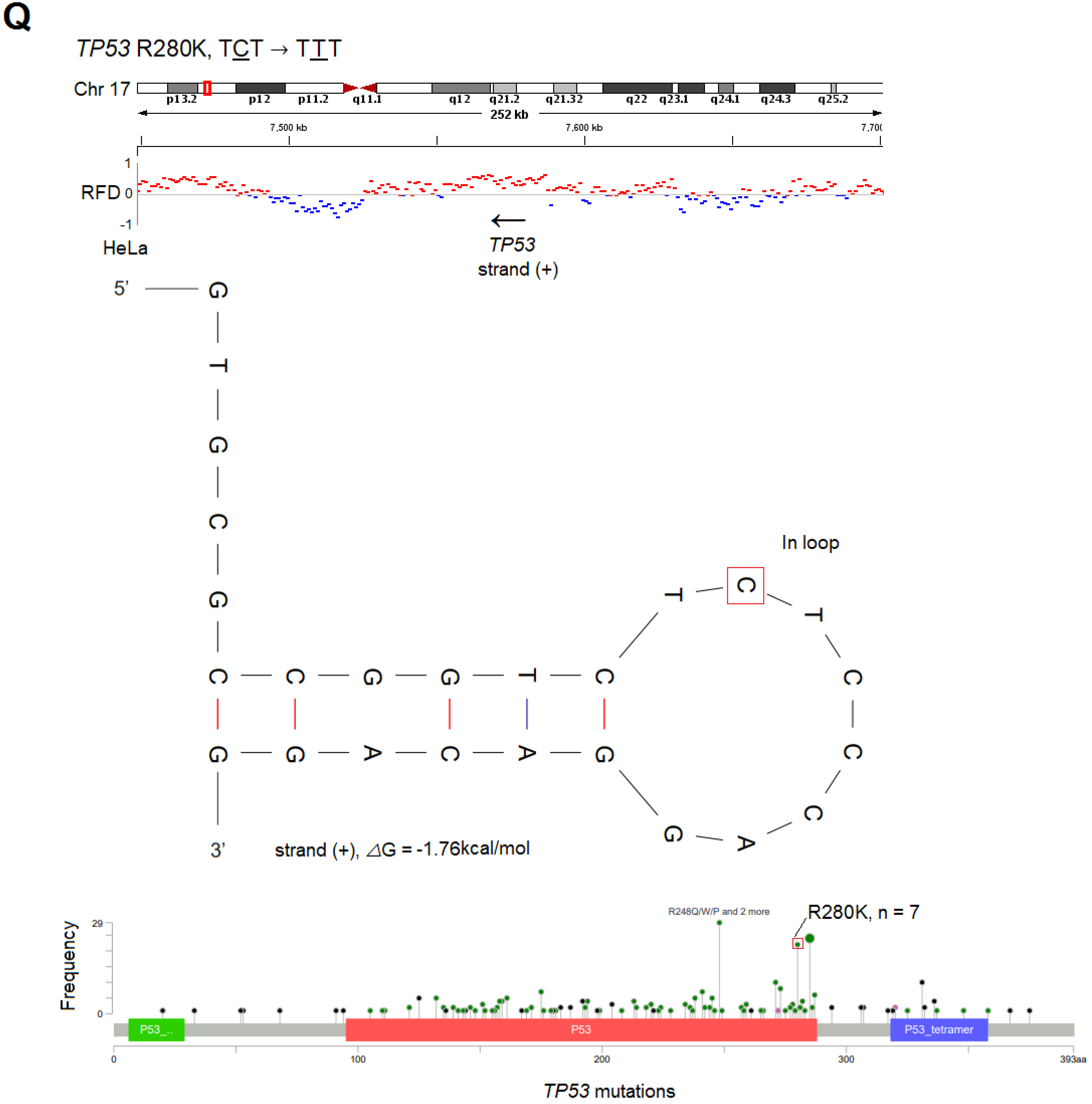

Q
TP53 R280K, TCT → TTT
Chr 17
p13.2
p12
p11.2
q11.1
q12
q21.2
q21.32
q22
q23.1
q24.1
q24.3
q25.2
252 kb
7,500 kb
7,600 kb
RFD
HeLa
TP53
strand (+)
5'
3'
In loop
strand (+), ⊿G = -1.76kcal/mol
R248Q/W/P and 2 more
R280K, n = 7
Frequency
29
0
P53_..
P53
P53_tetramer
100
200
300
393aa
TP53 mutations

R

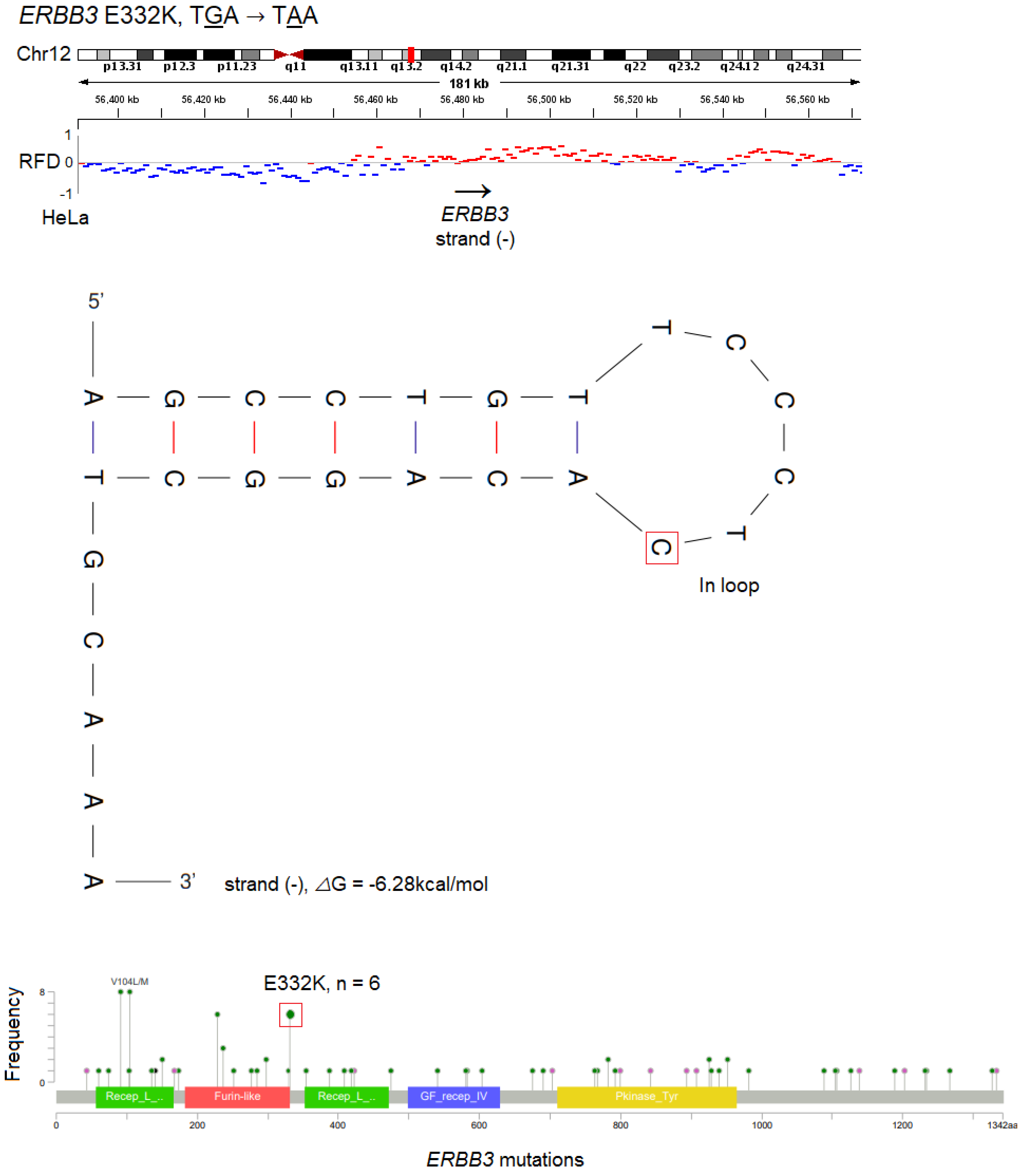

ERBB3 E332K, TGA → TAA
Chr12
p13.31 p12.3 p11.23 q11 q13.11 q13.2 q14.2 q21.1 q21.31 q22 q23.2 q24.12 q24.31
181 kb
56,400 kb 56,420 kb 56,440 kb 56,460 kb 56,480 kb 56,500 kb 56,520 kb 56,540 kb 56,560 kb
RFD
1
0
-1
HeLa
ERBB3
strand (-)
5'
3'
In loop
strand (-), ⊿G = -6.28kcal/mol
Frequency
V104L/M
E332K, n = 6
Recep_L_..
Furin-like
Recep_L_..
GF_recep_IV
Pkinase_Tyr
0
200
400
600
800
1000
1200
1342aa
ERBB3 mutations

S

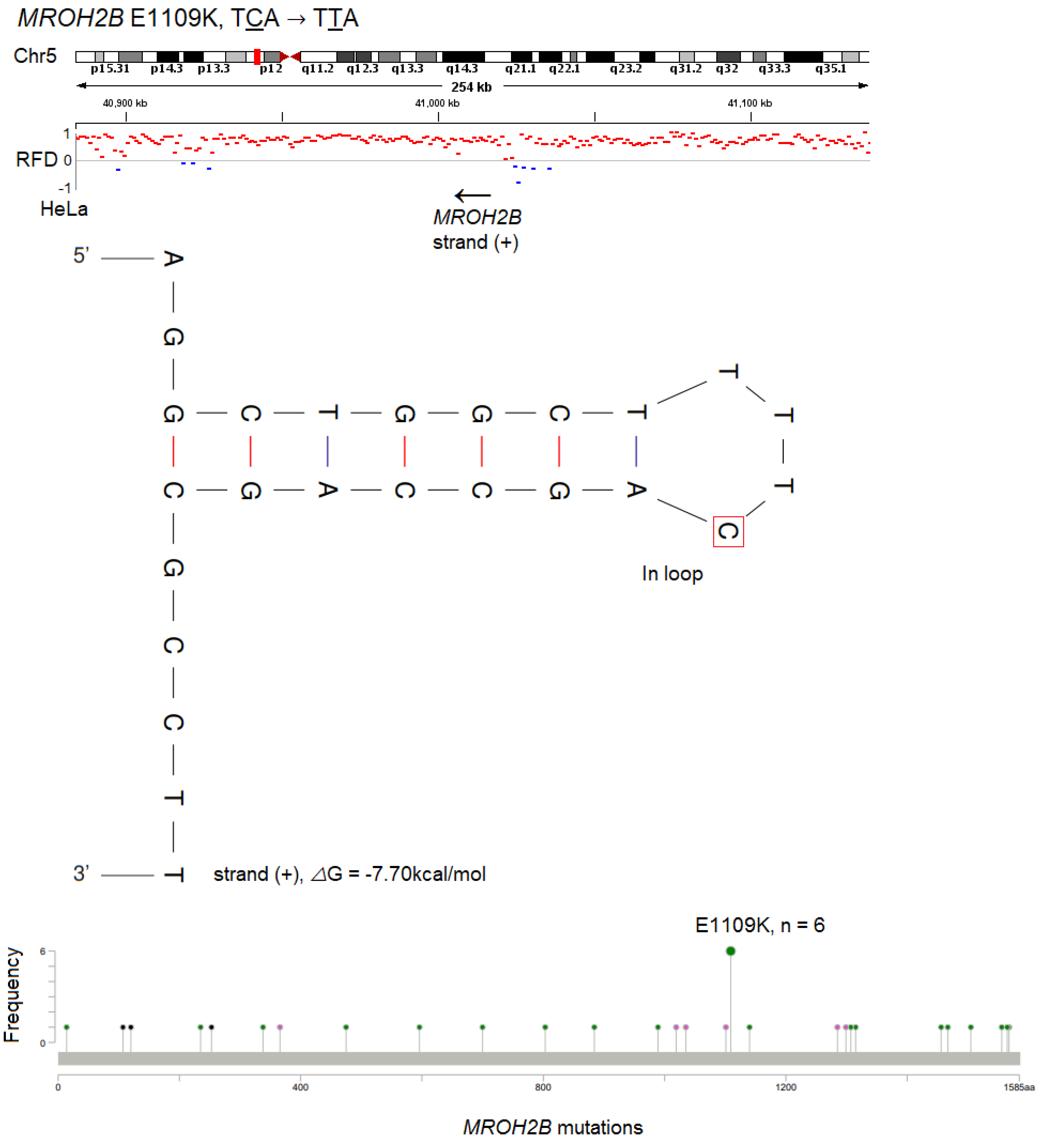
MROH2B E1109K, TCA → TTA
Chr5
p15.31
p14.3
p13.3
p12
q11.2
q12.3
q13.3
q14.3
q21.1
q22.1
q23.2
q31.2
q32
q33.3
q35.1
254 kb
40,900 kb
41,000 kb
41,100 kb
RFD
1
0
-1
HeLa
MROH2B
strand (+)
5'
3'
In loop
strand (+), ⊿G = -7.70kcal/mol
E1109K, n = 6
Frequency
6
0
400
800
1200
1585aa
MROH2B mutations

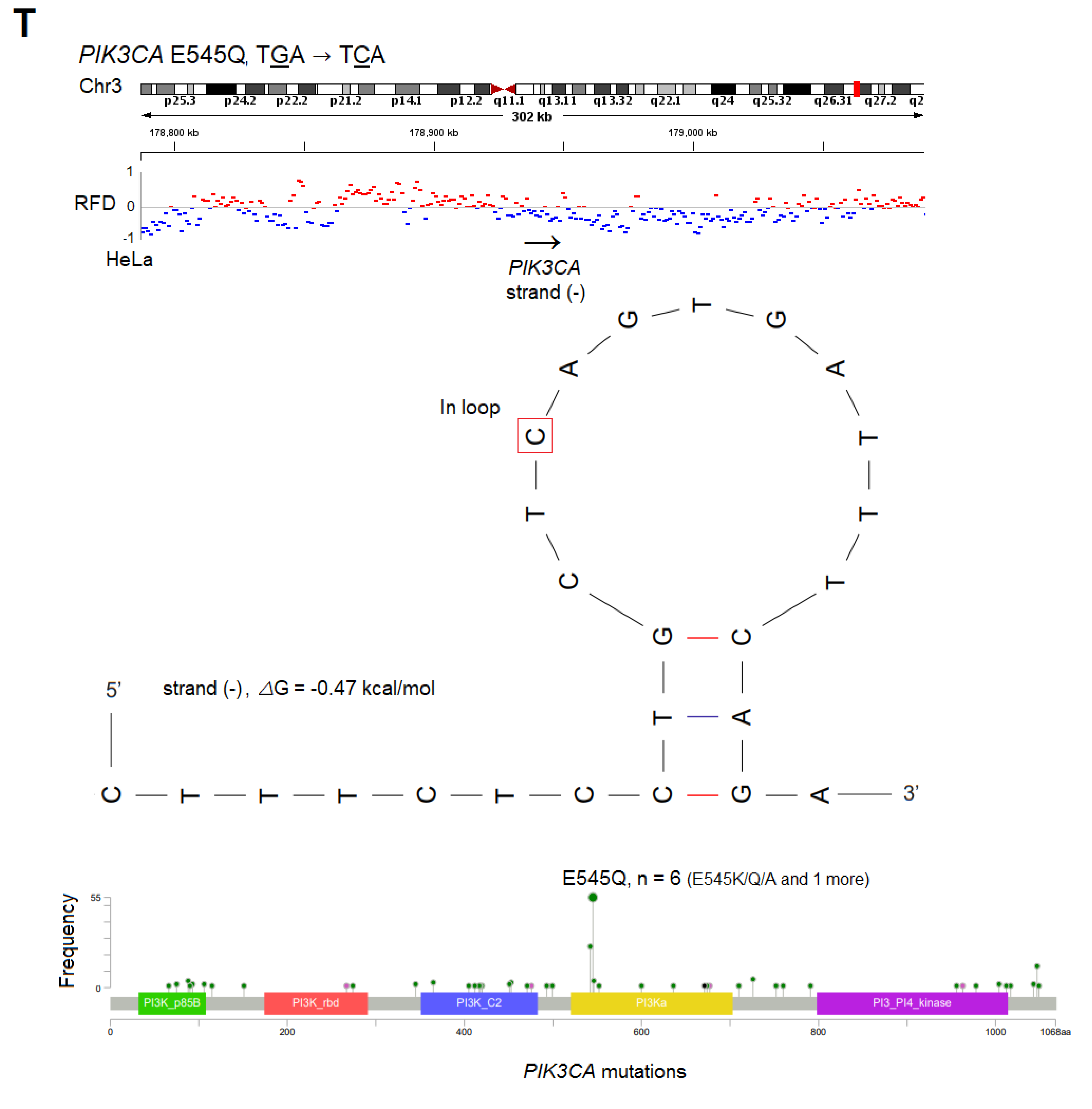

T
PIK3CA E545Q, TGA → TCA
Chr3
p25.3
p24.2
p22.2
p21.2
p14.1
p12.2
q11.1
q13.11
q13.32
q22.1
q24
q25.32
q26.31
q27.2
302 kb
178,800 kb
178,900 kb
179,000 kb
RFD
1
0
-1
HeLa
PIK3CA
strand (-)
In loop
5'
strand (-), ΔG = -0.47 kcal/mol
3'
E545Q, n = 6 (E545K/Q/A and 1 more)
55
0
Frequency
PI3K_p85B
PI3K_rbd
PI3K_C2
PI3Ka
PI3_PI4_kinase
0
200
400
600
800
1000
1068aa
PIK3CA mutations

U

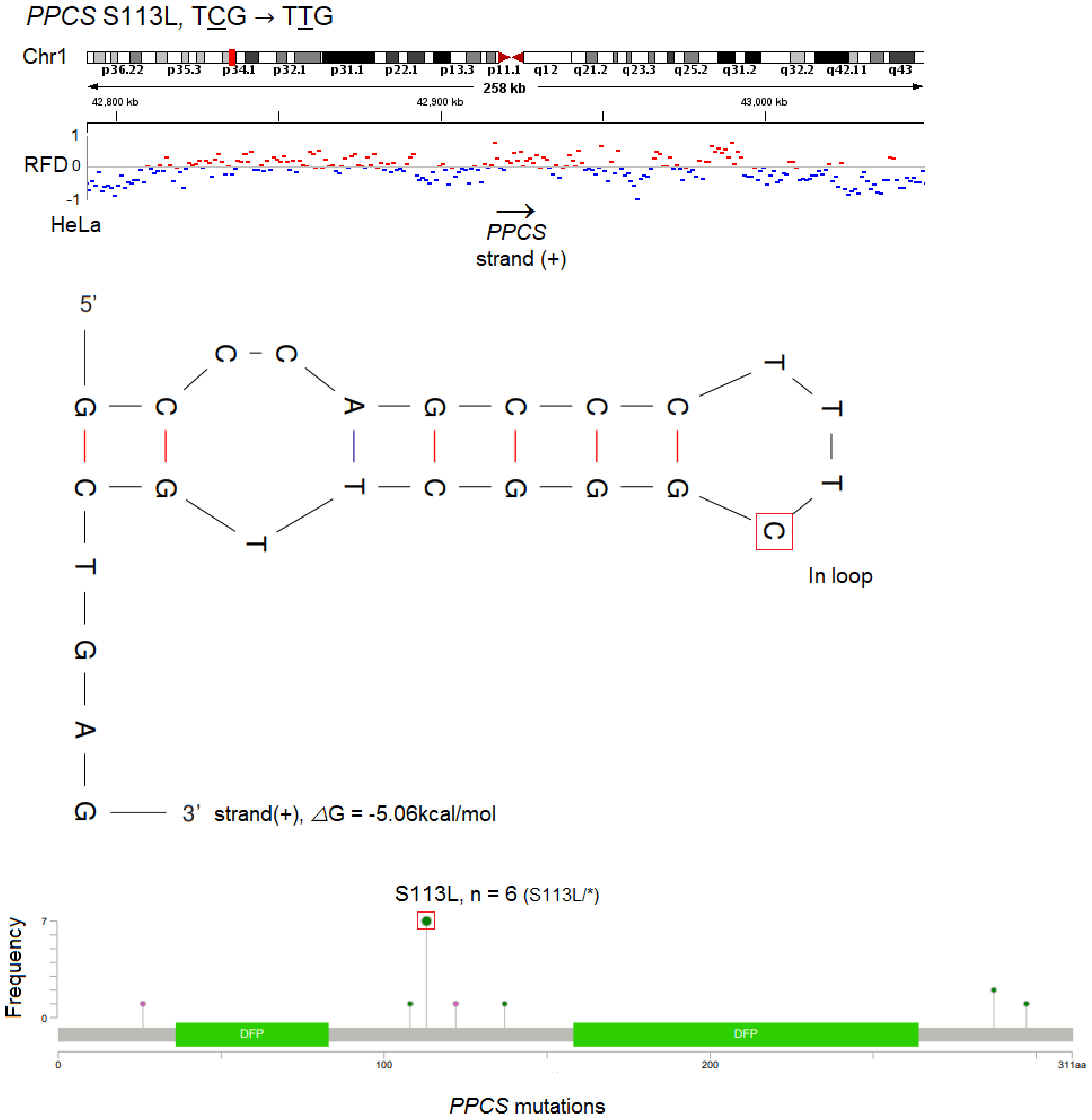

PPCS S113L, TCG → TTG
Chr1
p36.22 p35.3 p34.1 p32.1 p31.1 p22.1 p13.3 p11.1 q12 q21.2 q23.3 q25.2 q31.2 q32.2 q42.11 q43
258 kb
42,800 kb
42,900 kb
43,000 kb
RFD
1
0
-1
HeLa
PPCS
strand (+)
5'
In loop
3' strand(+), △G = -5.06kcal/mol
S113L, n = 6 (S113L/*)
Frequency
7
0
DFP
DFP
0
100
200
311aa
PPCS mutations

V

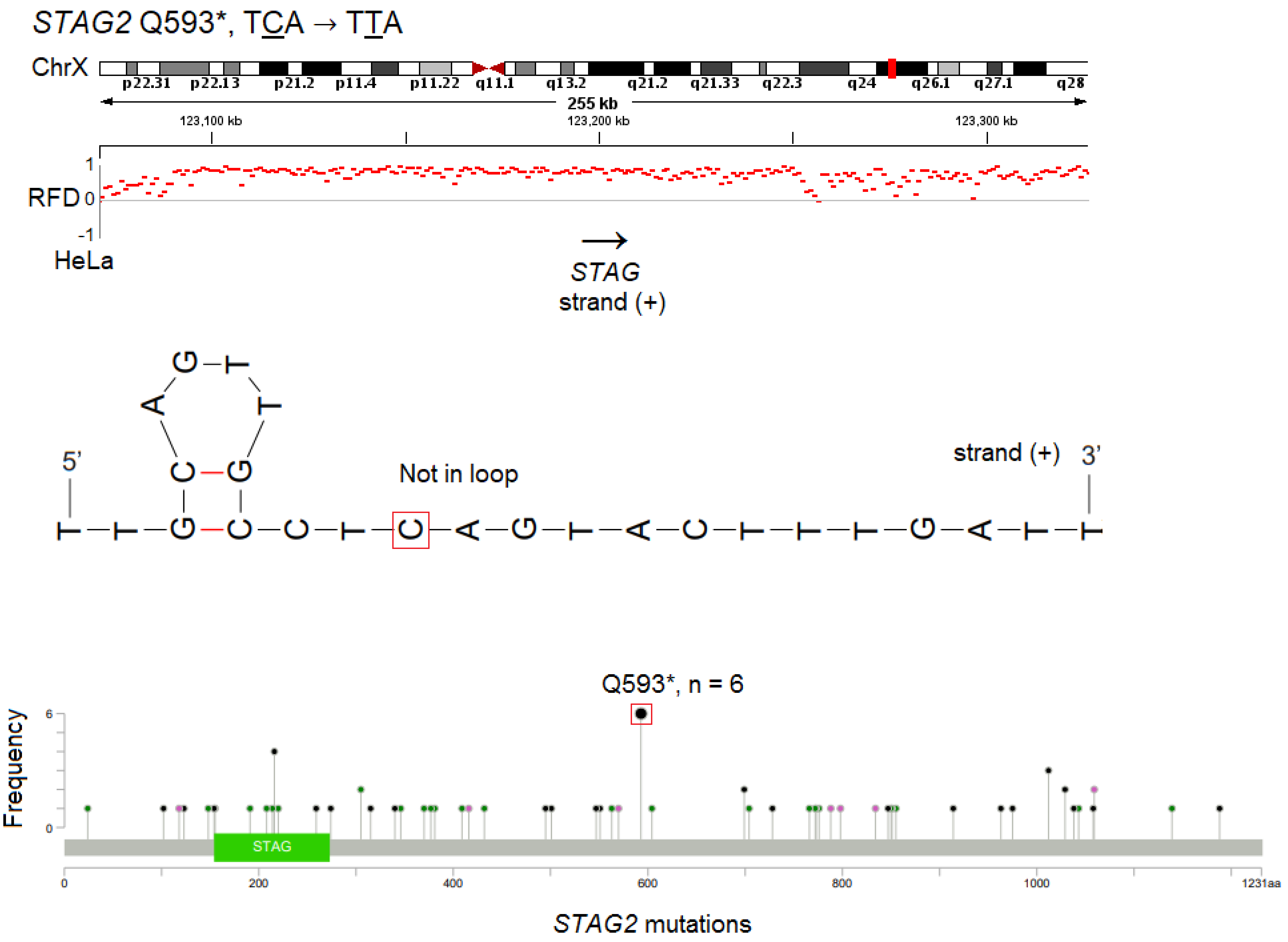
STAG2 Q593*, TCA → TTA
ChrX
p22.31 p22.13 p21.2 p11.4 p11.22 q11.1 q13.2 q21.2 q21.33 q22.3 q24 q26.1 q27.1 q28
255 kb
123,100 kb
123,200 kb
123,300 kb
RFD
1
0
-1
HeLa
STAG
strand (+)
5'
3'
Not in loop
strand (+)
Q593*, n = 6
Frequency
6
0
STAG
0
200
400
600
800
1000
1231aa
STAG2 mutations

W

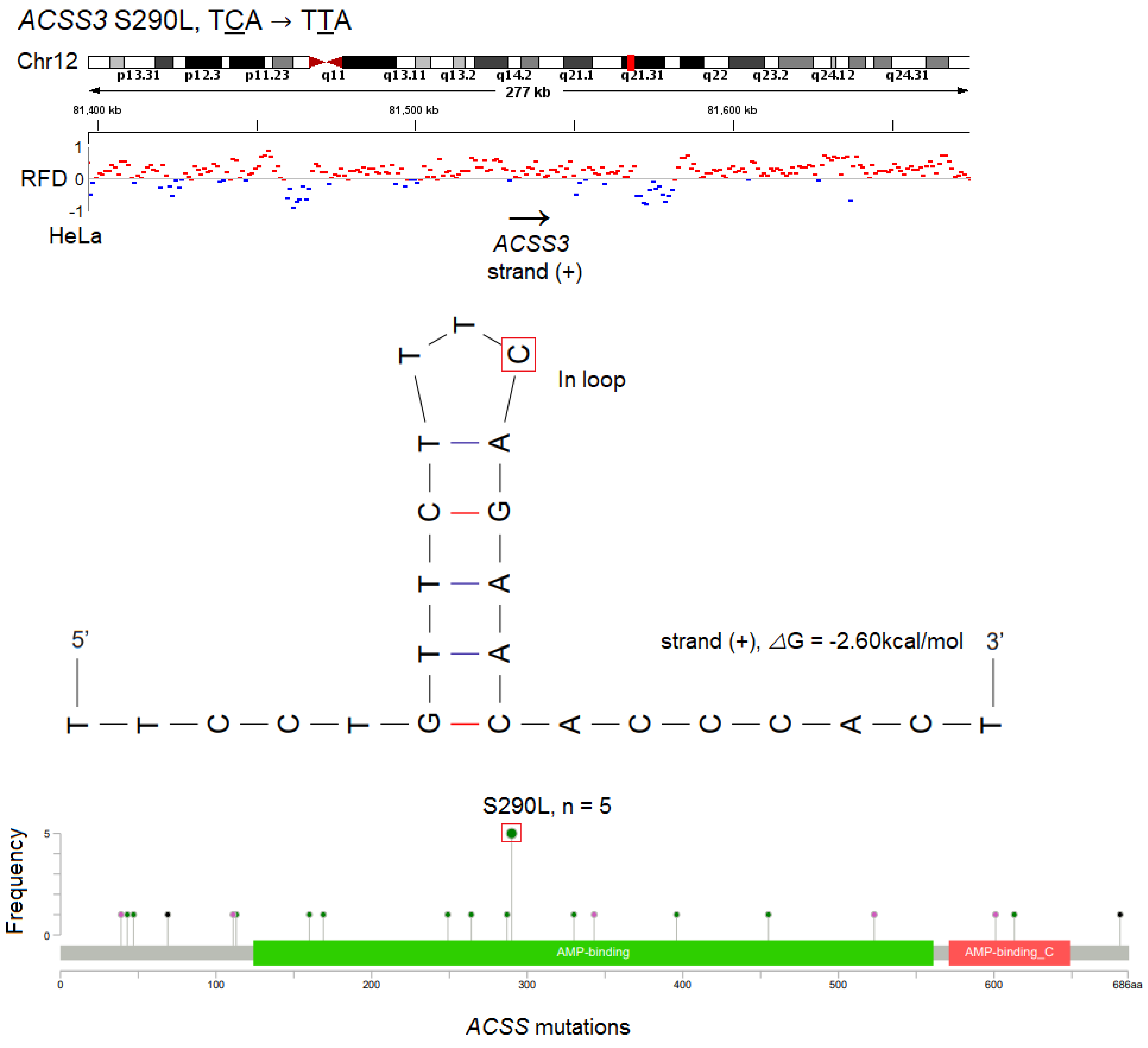

ACSS3 S290L, TCA → TTA
Chr12
p13.31
p12.3
p11.23
q11
q13.11
q13.2
q14.2
q21.1
q21.31
q22
q23.2
q24.12
q24.31
277 kb
81,400 kb
81,500 kb
81,600 kb
RFD
1
0
-1
HeLa
ACSS3
strand (+)
In loop
5'
3'
strand (+), ΔG = -2.60kcal/mol
S290L, n = 5
Frequency
AMP-binding
AMP-binding_C
0
100
200
300
400
500
600
686aa
ACSS mutations

X

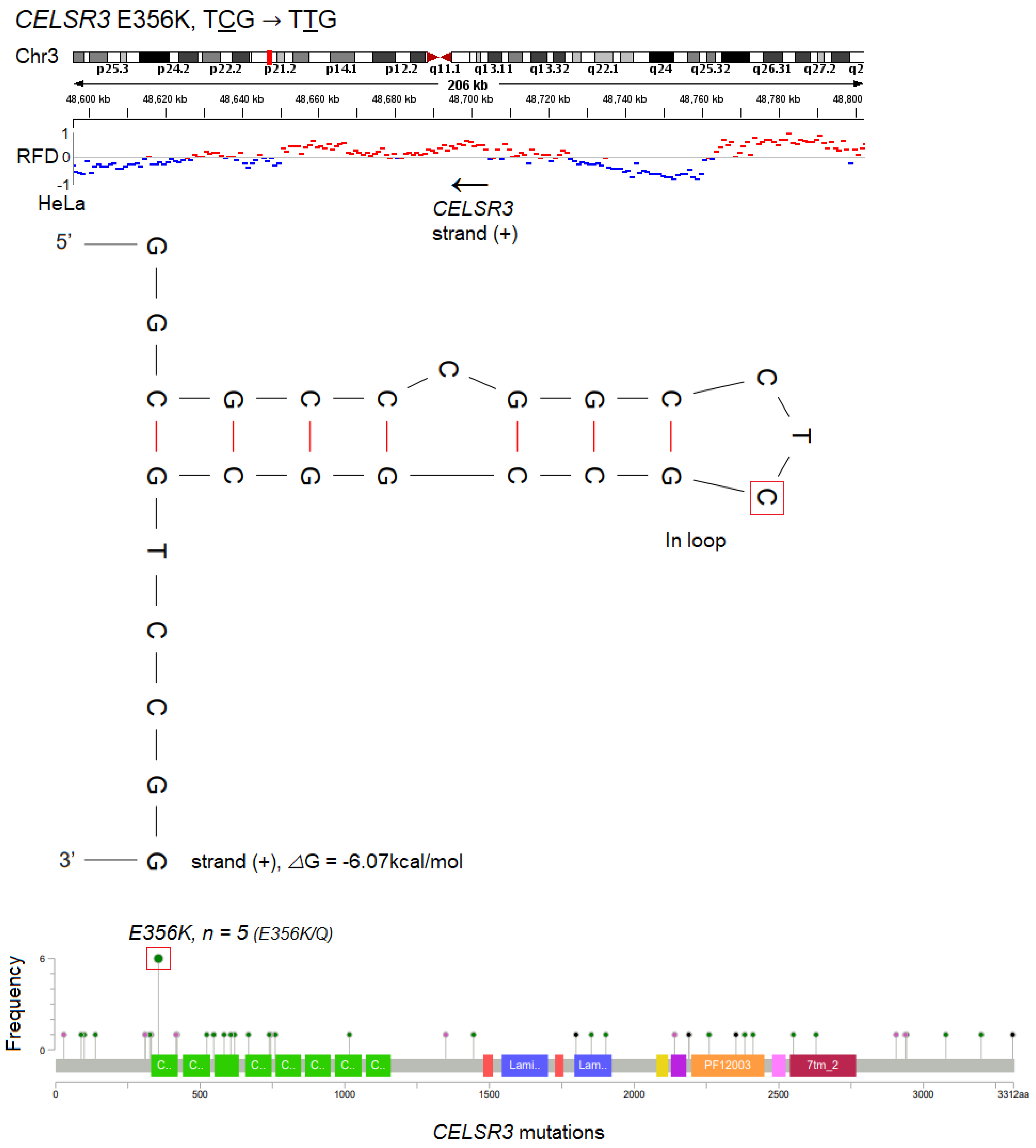

CELSR3 E356K, TCG → TTG
Chr3
p25.3
p24.2
p22.2
p21.2
p14.1
p12.2
q11.1
q13.11
q13.32
q22.1
q24
q25.32
q26.31
q27.2
q2
206 kb
48,600 kb
48,620 kb
48,640 kb
48,660 kb
48,680 kb
48,700 kb
48,720 kb
48,740 kb
48,760 kb
48,780 kb
48,800
RFD
1
0
-1
HeLa
CELSR3
strand (+)
5'
3'
In loop
strand (+), △G = -6.07kcal/mol
E356K, n = 5 (E356K/Q)
Frequency
6
0
C..
Lami..
Lam..
PF12003
7tm_2
0
500
1000
1500
2000
2500
3000
3312aa
CELSR3 mutations

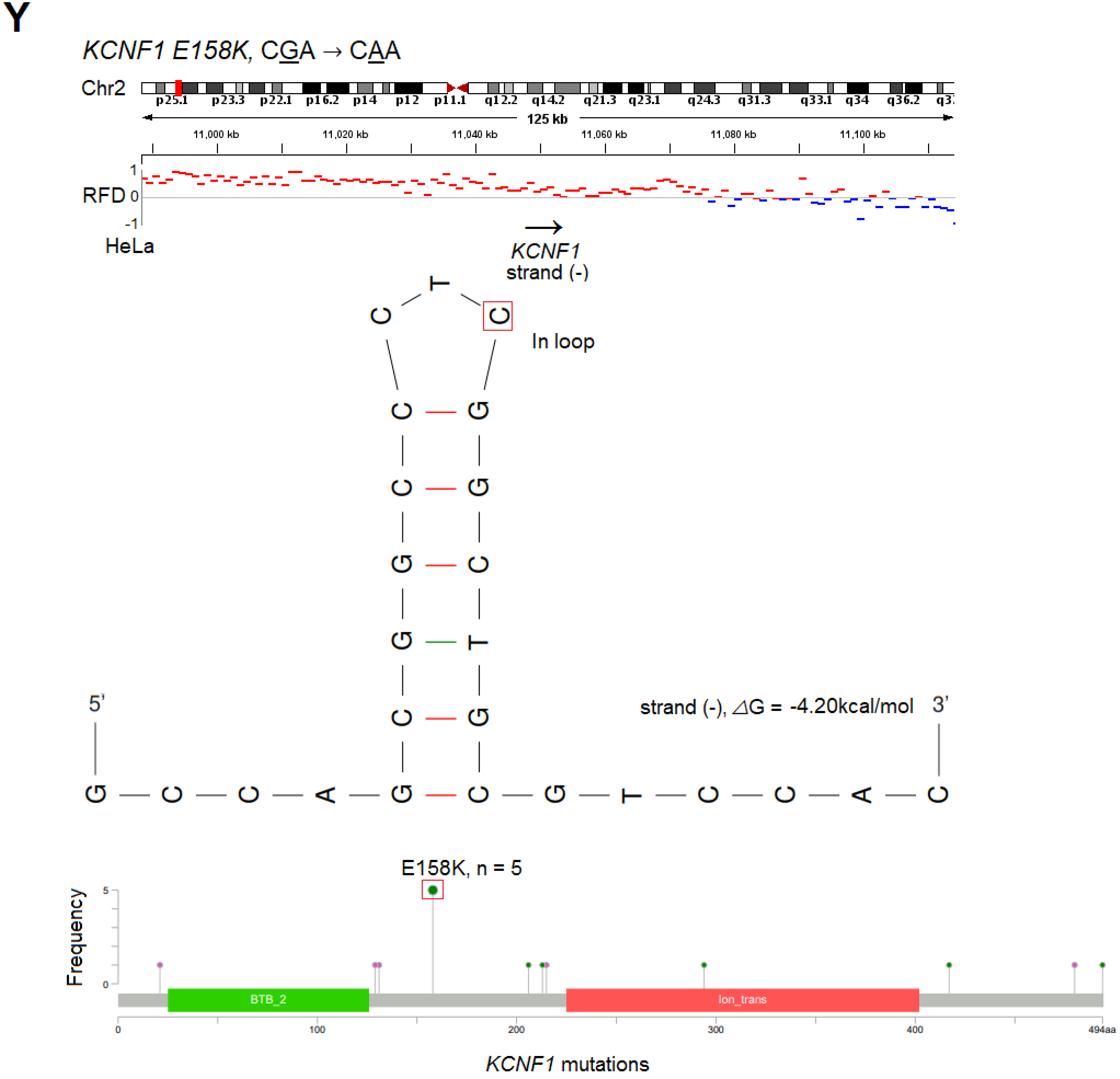
Y
KCNF1 E158K, CGA → CAA
Chr2
p25.1 p23.3 p22.1 p16.2 p14 p12 p11.1 q12.2 q14.2 q21.3 q23.1 q24.3 q31.3 q33.1 q34 q36.2
125 kb
11,000 kb
11,020 kb
11,040 kb
11,060 kb
11,080 kb
11,100 kb
RFD
1
0
-1
HeLa
KCNF1
strand (-)
In loop
5'
3'
strand (-), ΔG = -4.20kcal/mol
E158K, n = 5
Frequency
5
0
BTB_2
Ion_trans
0
100
200
300
400
494aa
KCNF1 mutations

Z

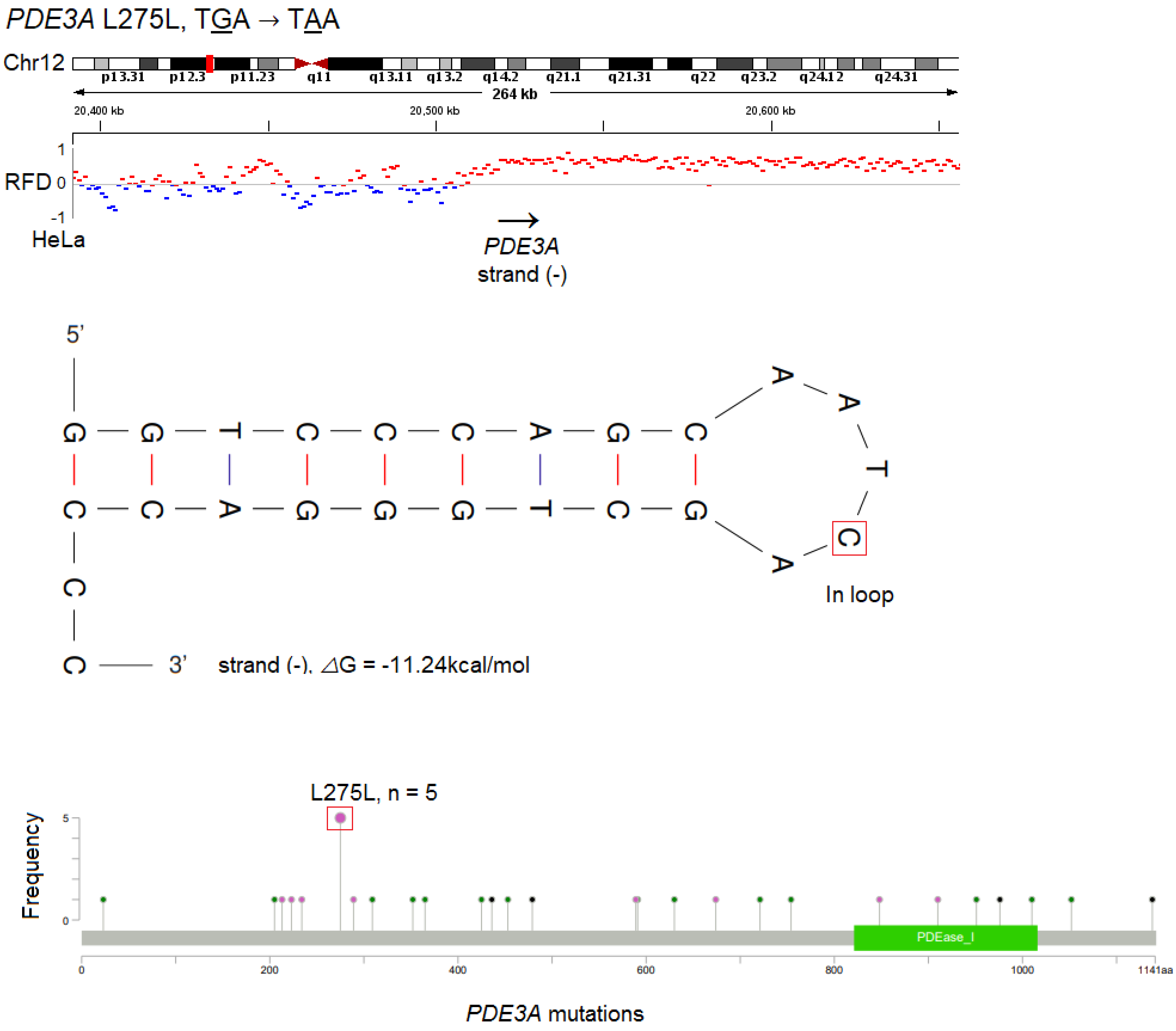

PDE3A L275L, TGA → TAA
Chr12
p13.31
p12.3
p11.23
q11
q13.11
q13.2
q14.2
q21.1
q21.31
q22
q23.2
q24.12
q24.31
264 kb
20,400 kb
20,500 kb
20,600 kb
RFD
1
0
-1
HeLa
PDE3A
strand (-)
5'
3'
strand (-), ΔG = -11.24kcal/mol
In loop
L275L, n = 5
Frequency
5
0
PDEase_I
0
200
400
600
800
1000
1141aa
PDE3A mutations

AA

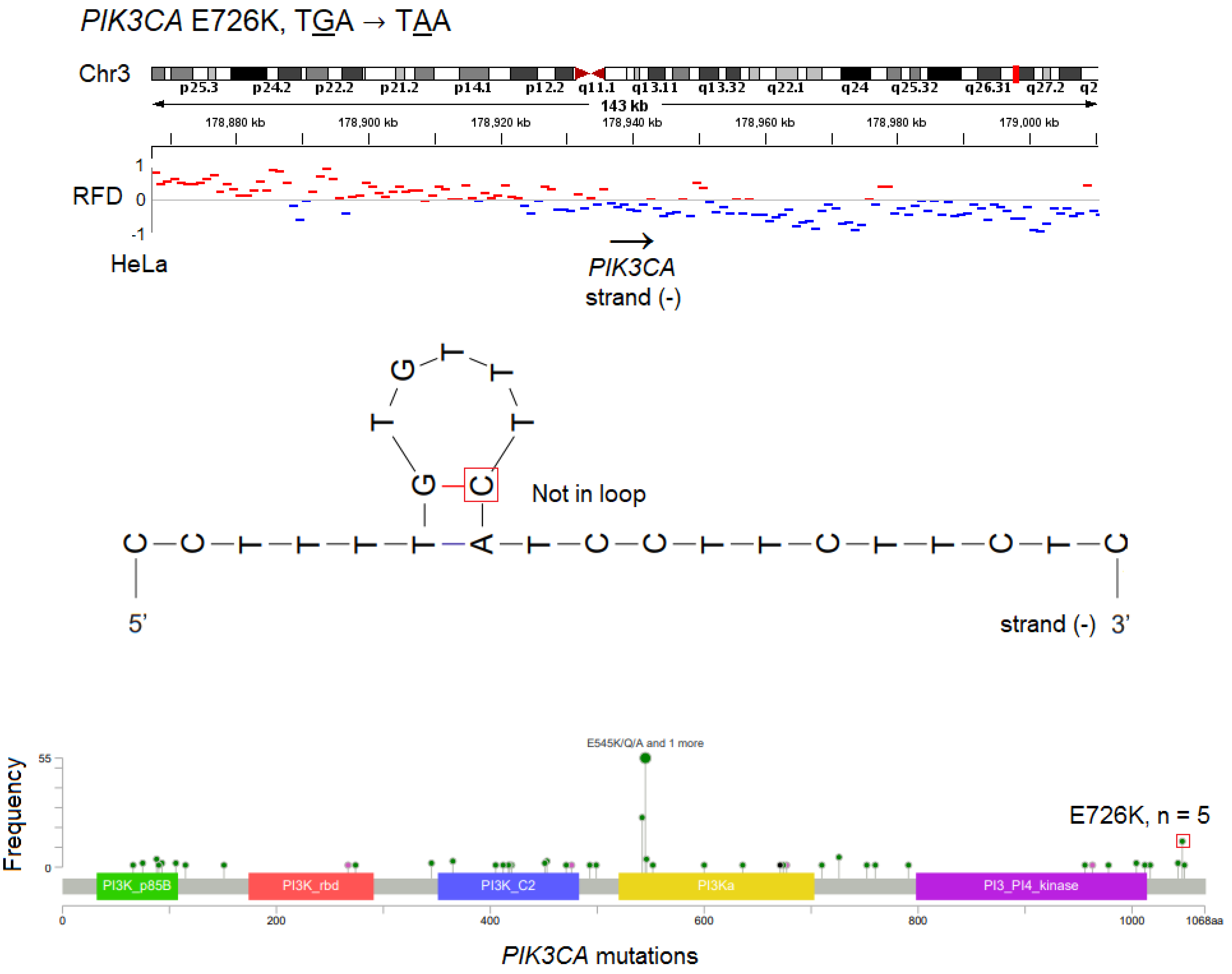

PIK3CA E726K, TGA → TAA
Chr3
p25.3
p24.2
p22.2
p21.2
p14.1
p12.2
q11.1
q13.11
q13.32
q22.1
q24
q25.32
q26.31
q27.2
q2
143 kb
178,880 kb
178,900 kb
178,920 kb
178,940 kb
178,960 kb
178,980 kb
179,000 kb
RFD
1
0
-1
HeLa
PIK3CA
strand (-)
Not in loop
5'
strand (-) 3'
Frequency
55
0
E545K/Q/A and 1 more
E726K, n = 5
PI3K_p85B
PI3K_rbd
PI3K_C2
PI3Ka
PI3_PI4_kinase
0
200
400
600
800
1000
1068aa
PIK3CA mutations

AB

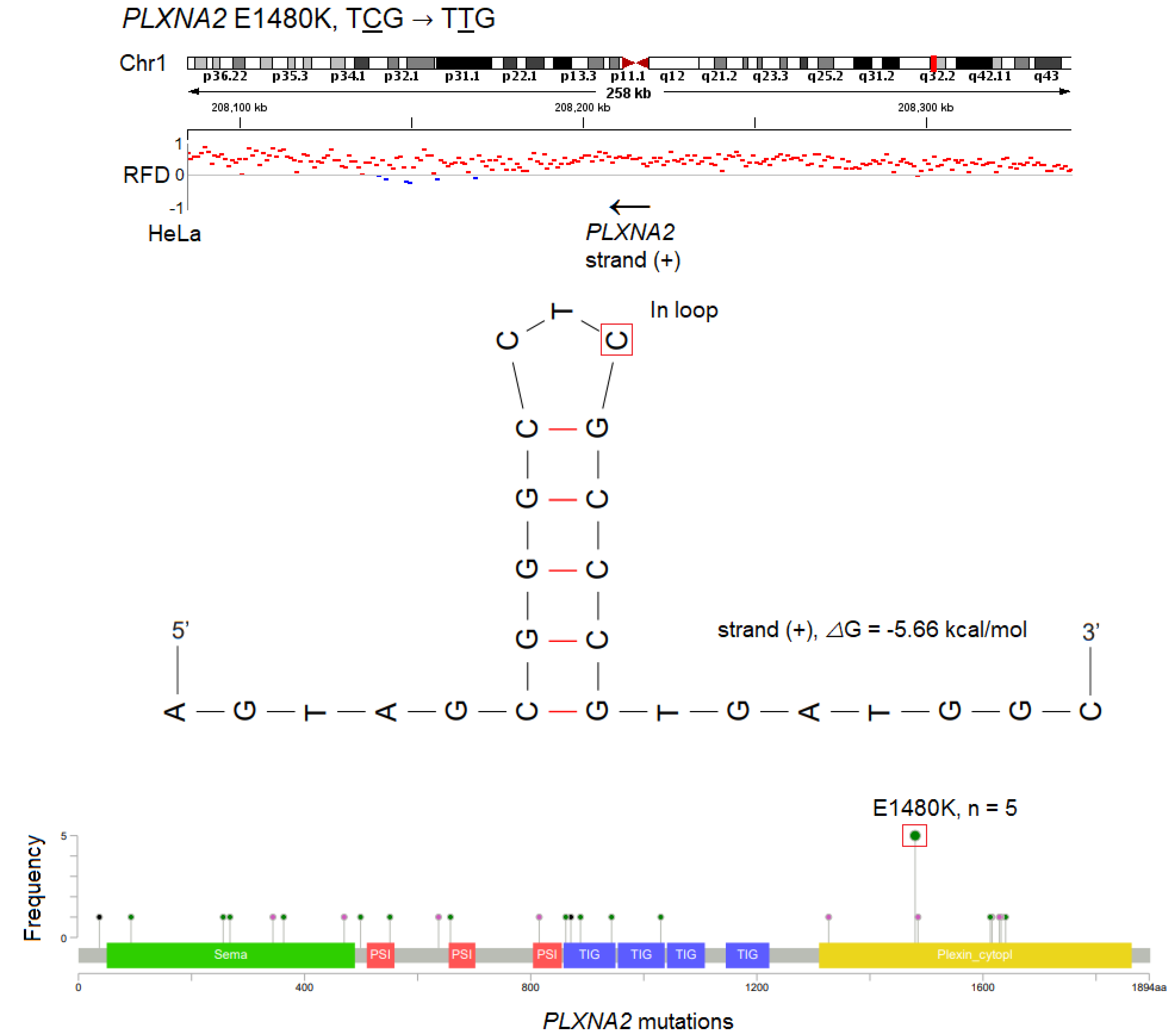

PLXNA2 E1480K, TCG → TTG
Chr1
p36.22 p35.3 p34.1 p32.1 p31.1 p22.1 p13.3 p11.1 q12 q21.2 q23.3 q25.2 q31.2 q32.2 q42.11 q43
258 kb
208,100 kb
208,200 kb
208,300 kb
RFD
1
0
-1
HeLa
PLXNA2
strand (+)
In loop
5'
3'
strand (+), ΔG = -5.66 kcal/mol
E1480K, n = 5
Frequency
Sema
PSI
TIG
Plexin_cytopl
0
400
800
1200
1600
1894aa
PLXNA2 mutations

AC

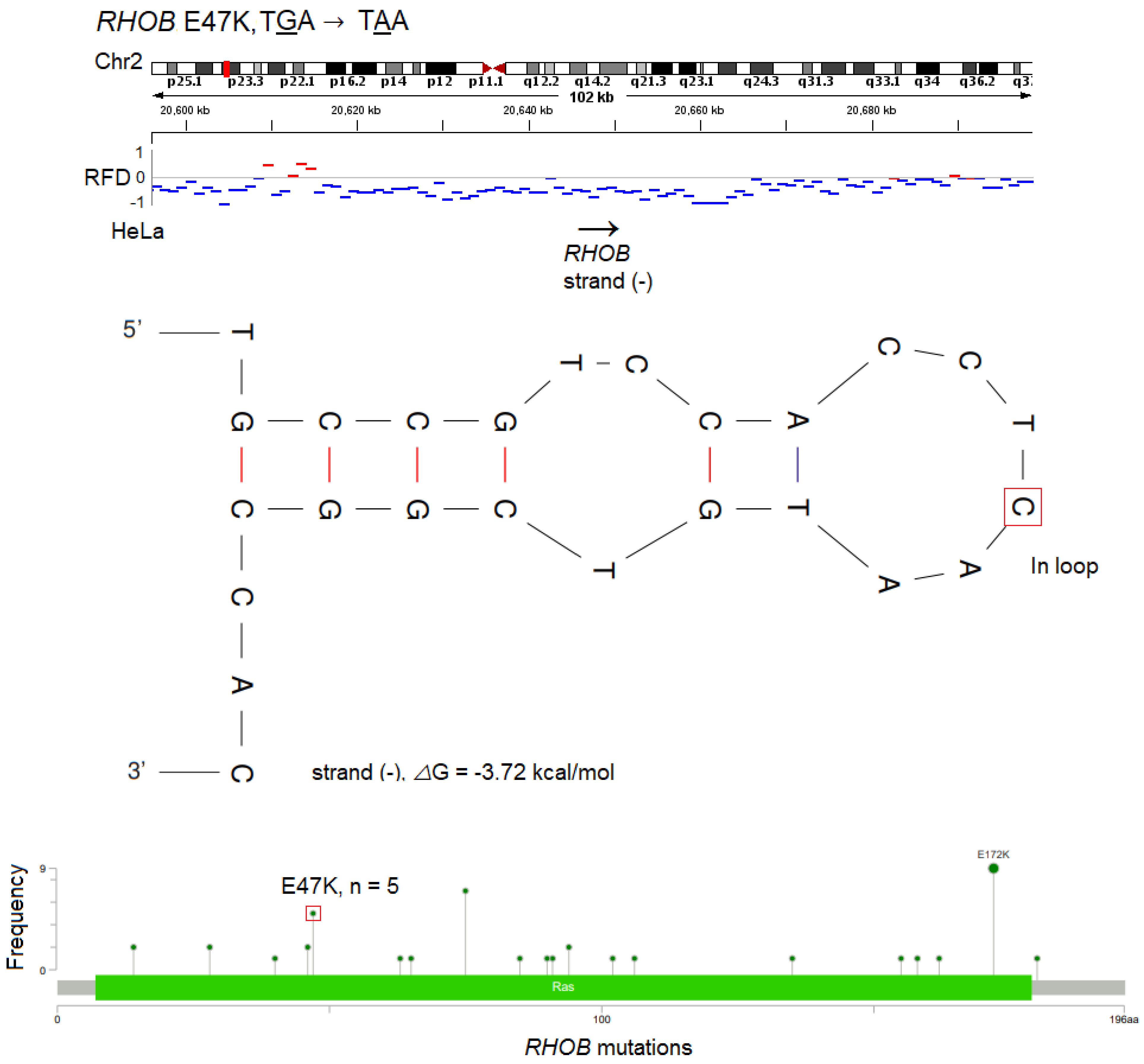

RHOB E47K, TGA → TAA
Chr2
p25.1 p23.3 p22.1 p16.2 p14 p12 p11.1 q12.2 q14.2 q21.3 q23.1 q24.3 q31.3 q33.1 q34 q36.2
102 kb
20,600 kb
20,620 kb
20,640 kb
20,660 kb
20,680 kb
RFD
1
0
-1
HeLa
RHOB
strand (-)
5'
3'
In loop
strand (-), ΔG = -3.72 kcal/mol
E47K, n = 5
E172K
Frequency
9
0
Ras
0
100
196aa
RHOB mutations

AD

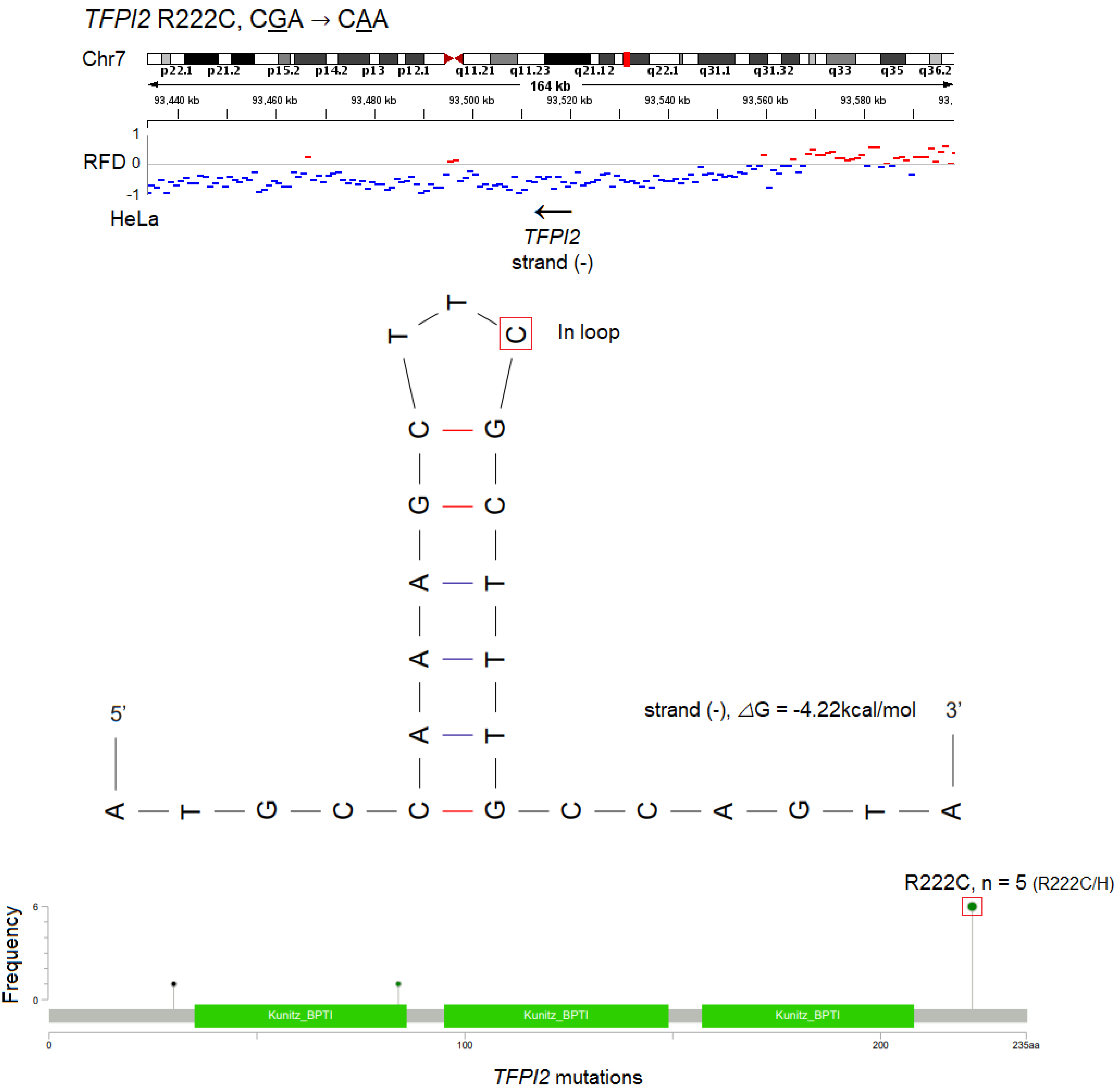

TFPI2 R222C, CGA → CAA
Chr7
p22.1 p21.2 p15.2 p14.2 p13 p12.1 q11.21 q11.23 q21.12 q22.1 q31.1 q31.32 q33 q35 q36.2
164 kb
93,440 kb 93,460 kb 93,480 kb 93,500 kb 93,520 kb 93,540 kb 93,560 kb 93,580 kb
RFD
1
0
-1
HeLa
TFPI2
strand (-)
In loop
5'
3'
strand (-), ΔG = -4.22kcal/mol
A T G C C G C C A G T A
R222C, n = 5 (R222C/H)
Frequency
6
0
Kunitz_BPTI
Kunitz_BPTI
Kunitz_BPTI
0
100
200
235aa
TFPI2 mutations

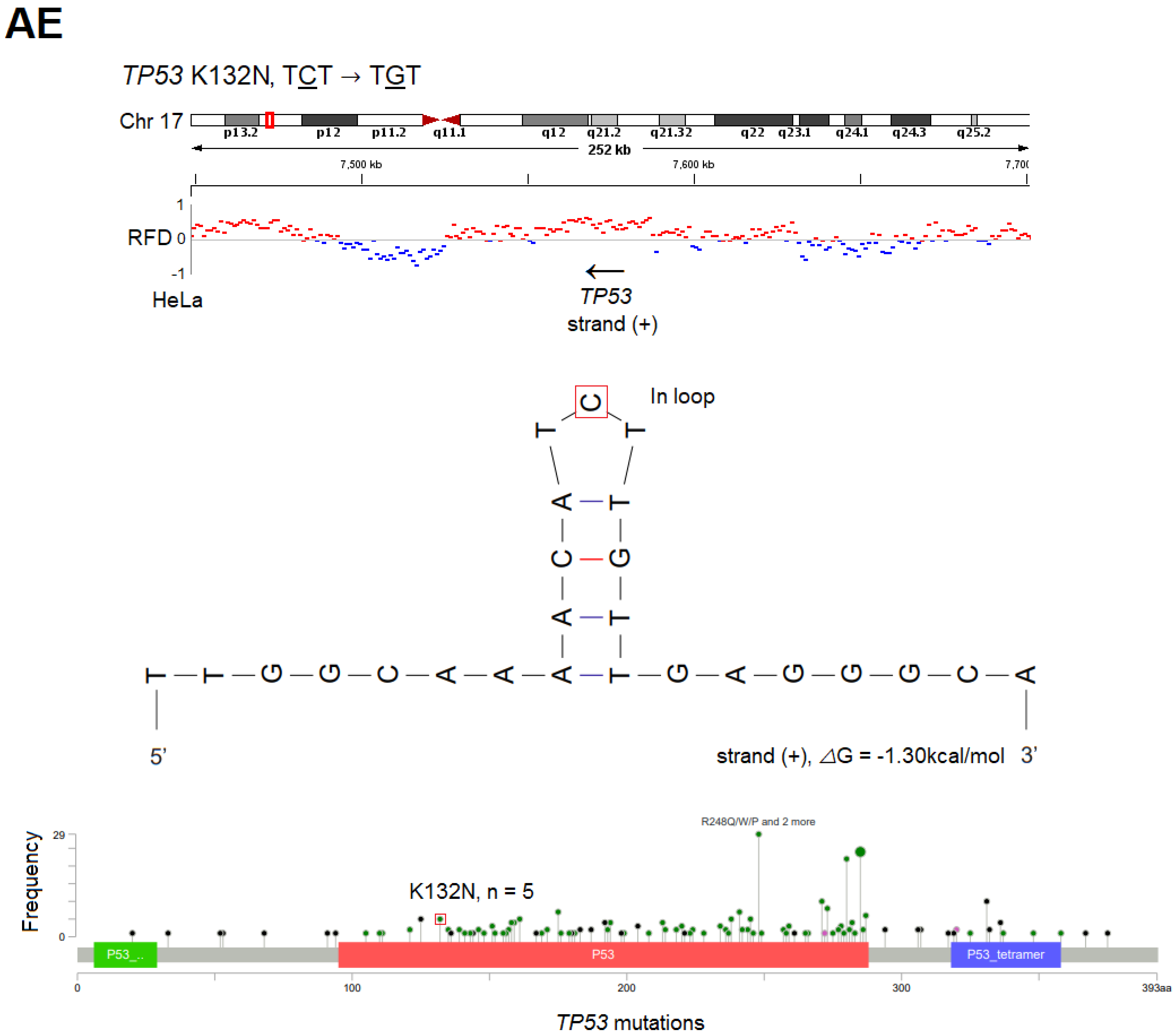

AE
TP53 K132N, TCT → TGT
Chr 17
p13.2
p12
p11.2
q11.1
q12
q21.2
q21.32
q22
q23.1
q24.1
q24.3
q25.2
252 kb
7,500 kb
7,600 kb
RFD
1
0
-1
HeLa
TP53
strand (+)
In loop
5'
3'
strand (+), △G = -1.30kcal/mol
Frequency
29
0
R248Q/W/P and 2 more
K132N, n = 5
P53_..
P53
P53_tetramer
100
200
300
393aa
TP53 mutations

AF

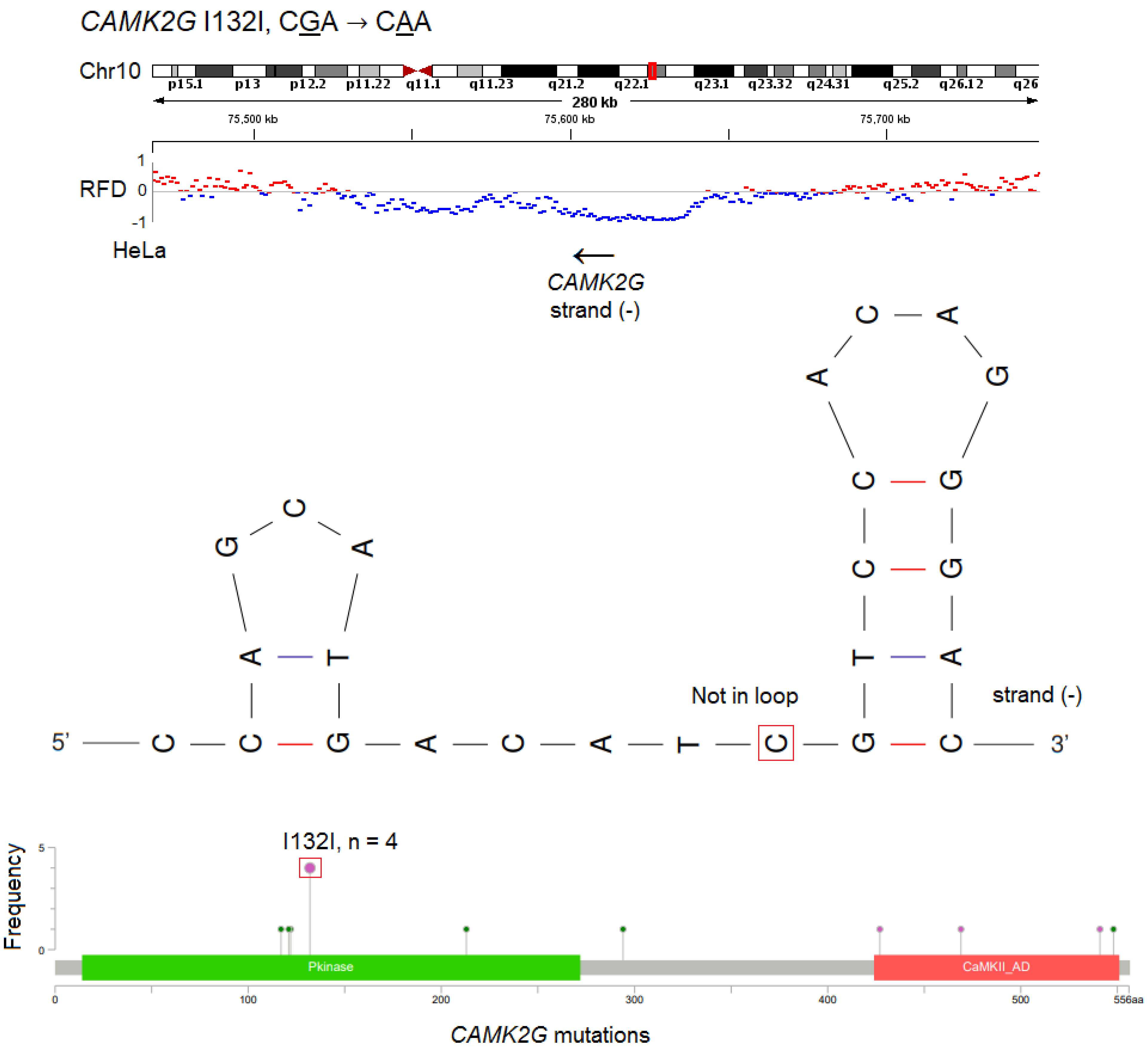

CAMK2G I132I, CGA → CAA
Chr10
p15.1 p13 p12.2 p11.22 q11.1 q11.23 q21.2 q22.1 q23.1 q23.32 q24.31 q25.2 q26.12 q26
280 kb
75,500 kb
75,600 kb
75,700 kb
RFD
1
0
-1
HeLa
CAMK2G
strand (-)
Not in loop
strand (-)
5' C C G A C A T C G C 3'
I132I, n = 4
Frequency
5
0
Pkinase
CaMKII_AD
0
100
200
300
400
500
556aa
CAMK2G mutations

AG

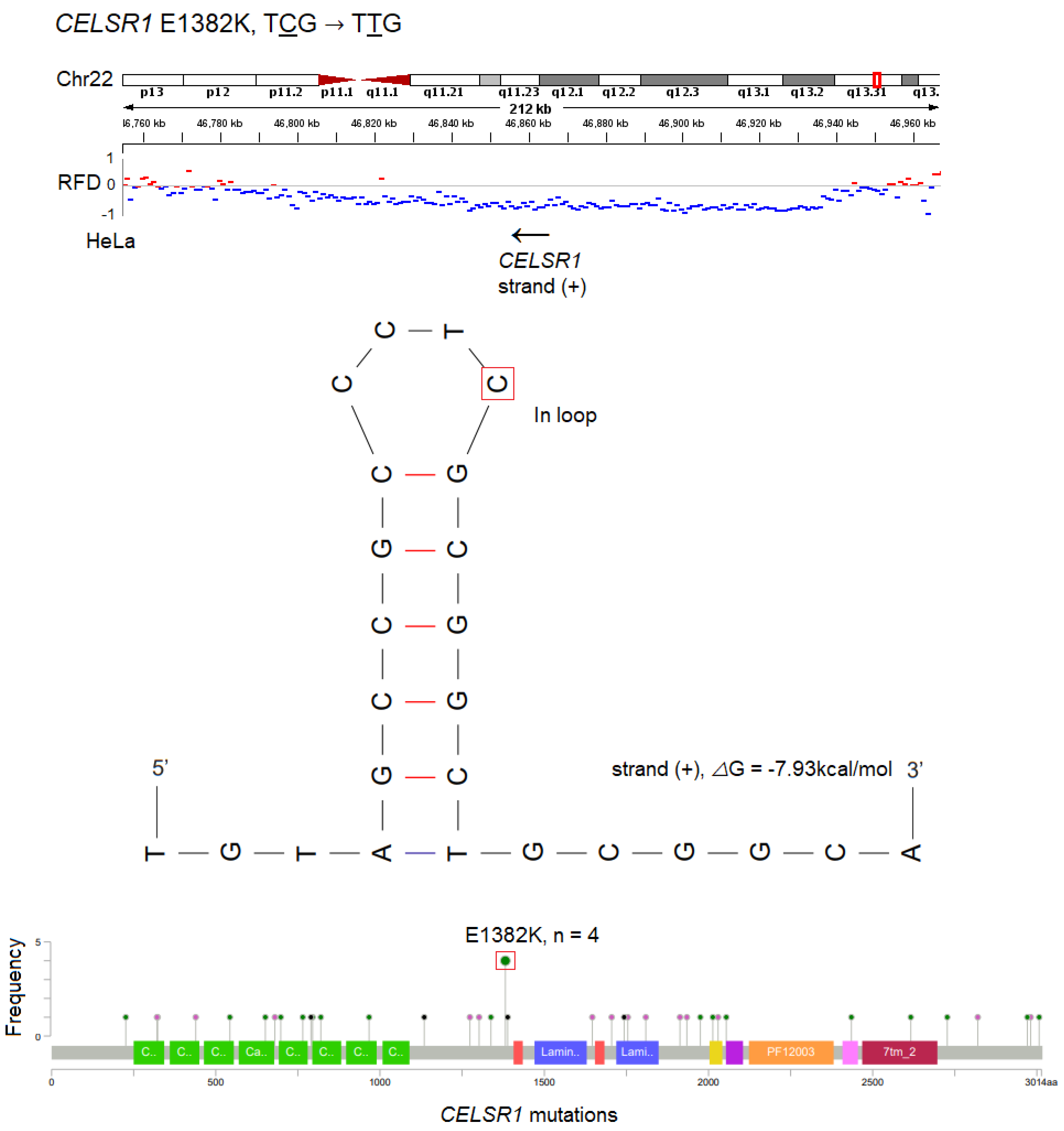

CELSR1 E1382K, TCG → TTG
Chr22
p13 p12 p11.2 p11.1 q11.1 q11.21 q11.23 q12.1 q12.2 q12.3 q13.1 q13.2 q13.31 q13.
212 kb
46,780 kb 46,800 kb 46,820 kb 46,840 kb 46,860 kb 46,880 kb 46,900 kb 46,920 kb 46,940 kb 46,960 kb
RFD
1
0
-1
HeLa
CELSR1
strand (+)
In loop
5'
3'
strand (+), ⊿G = -7.93kcal/mol
E1382K, n = 4
Frequency
Lamin..
Lami..
PF12003
7tm_2
3014aa
CELSR1 mutations

AH

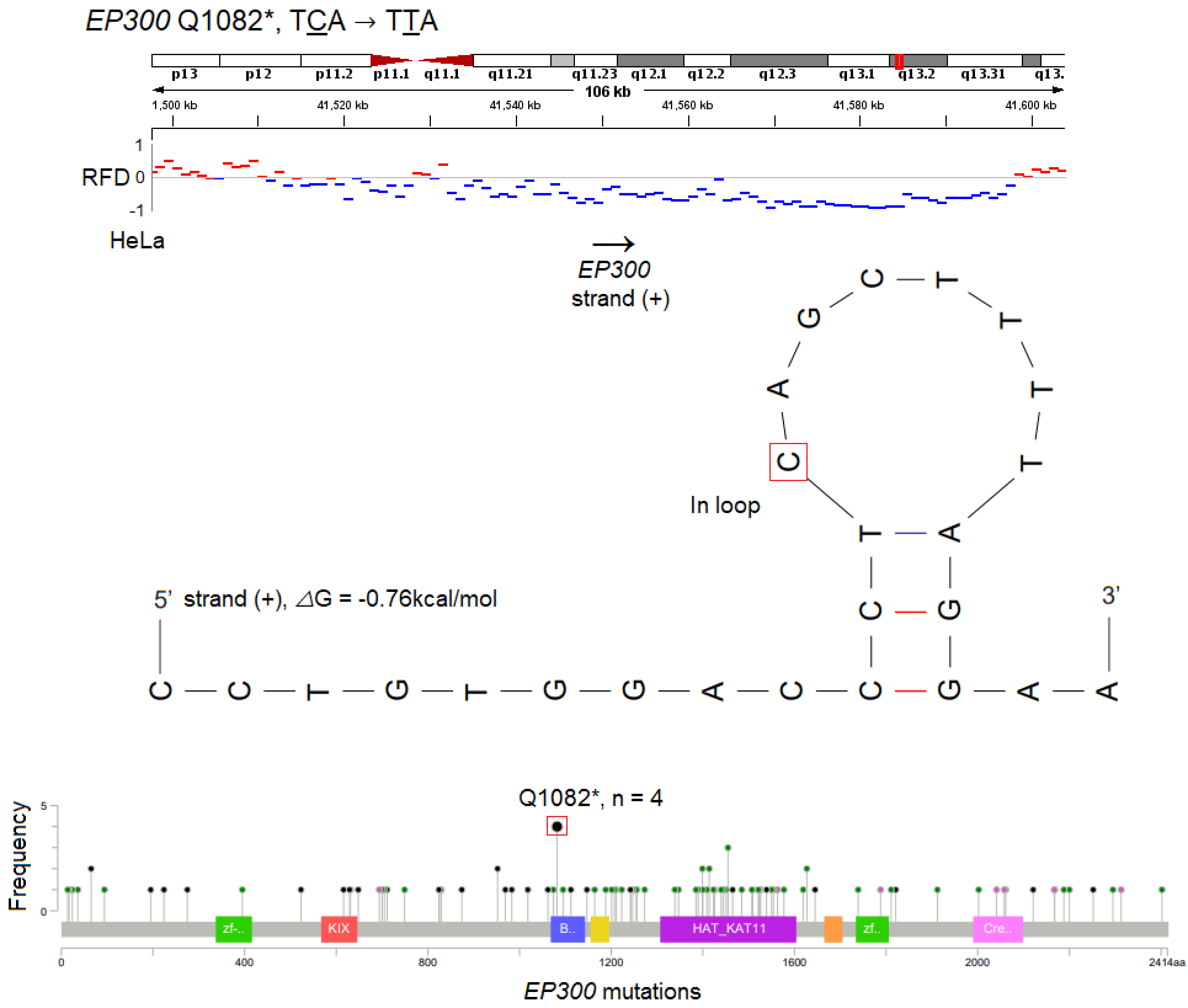
EP300 Q1082*, TCA → TTA
p13
p12
p11.2
p11.1
q11.1
q11.21
q11.23
q12.1
q12.2
q12.3
q13.1
q13.2
q13.31
106 kb
1,500 kb
41,520 kb
41,540 kb
41,560 kb
41,580 kb
41,600 kb
RFD
1
0
-1
HeLa
EP300
strand (+)
In loop
5' strand (+), ΔG = -0.76kcal/mol
3'
Q1082*, n = 4
Frequency
5
0
zf-..
KIX
B..
HAT_KAT11
zf..
Cre..
400
800
1200
1600
2000
2414aa
EP300 mutations

AI

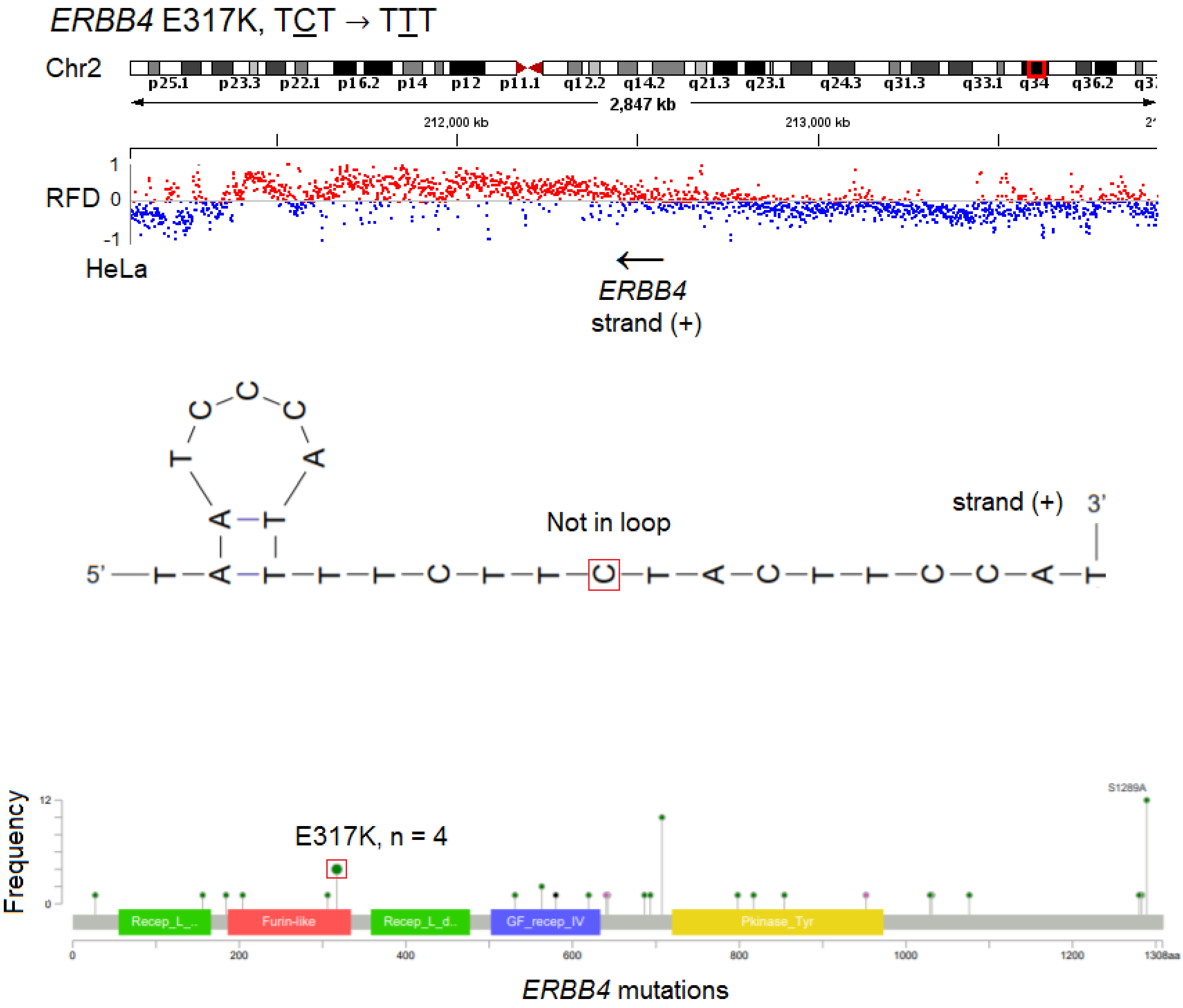
ERBB4 E317K, TCT → TTT
Chr2
p25.1
p23.3
p22.1
p16.2
p14
p12
p11.1
q12.2
q14.2
q21.3
q23.1
q24.3
q31.3
q33.1
q34
q36.2
2,847 kb
212,000 kb
213,000 kb
RFD
1
0
-1
HeLa
ERBB4
strand (+)
Not in loop
strand (+)
5'
3'
E317K, n = 4
S1289A
Frequency
12
0
Recep_L_...
Furin-like
Recep_L_d..
GF_recep_IV
Pkinase_Tyr
0
200
400
600
800
1000
1200
1308aa
ERBB4 mutations

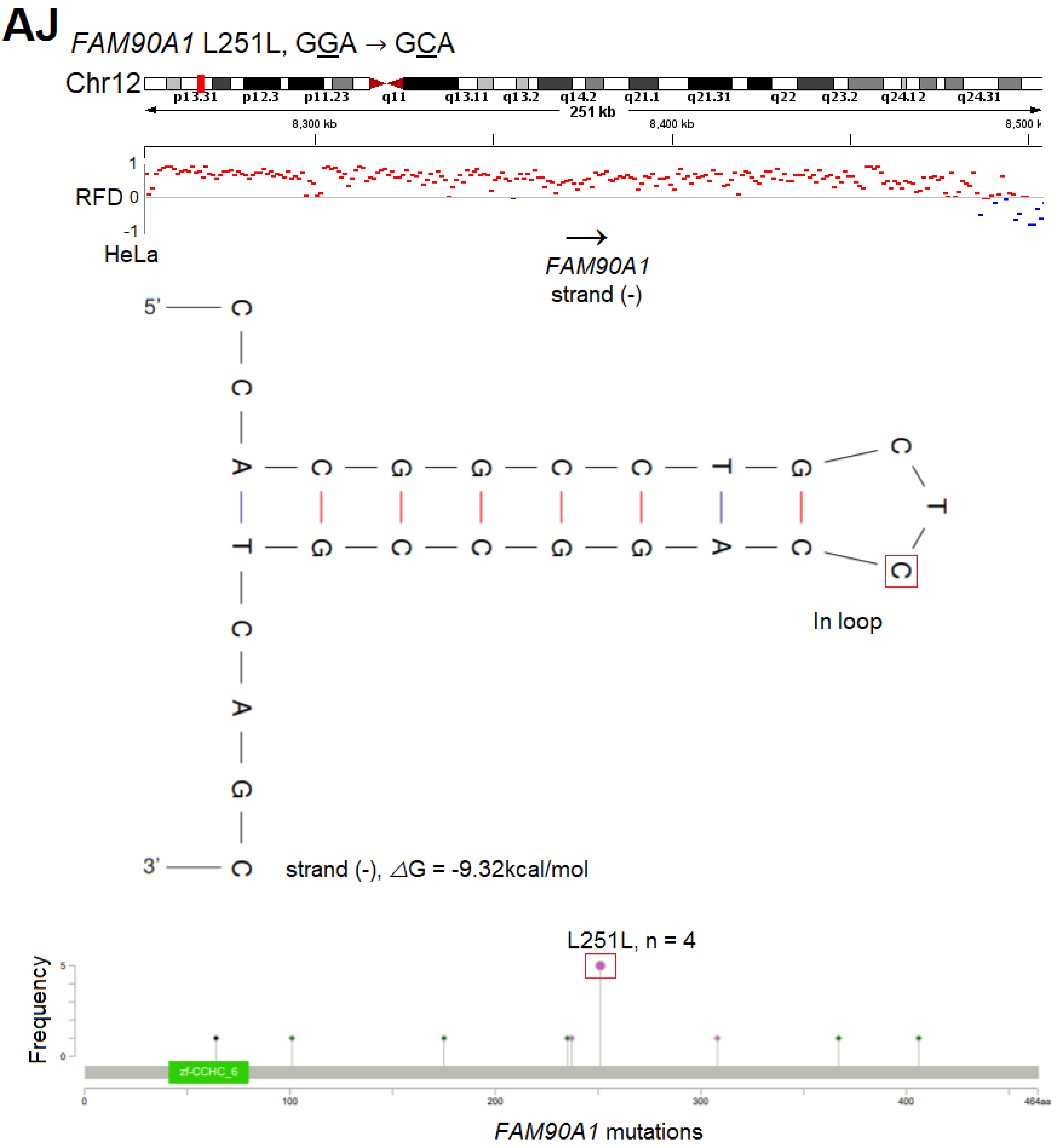

AJ
FAM90A1 L251L, GGA → GCA
Chr12
p13.31
p12.3
p11.23
q11
q13.11
q13.2
q14.2
q21.1
q21.31
q22
q23.2
q24.12
q24.31
251 kb
8,300 kb
8,400 kb
RFD
1
0
-1
HeLa
FAM90A1
strand (-)
5'
3'
In loop
strand (-), ⊿G = -9.32kcal/mol
L251L, n = 4
Frequency
5
0
zf-CCHC_6
0
100
200
300
400
464aa
FAM90A1 mutations

AK

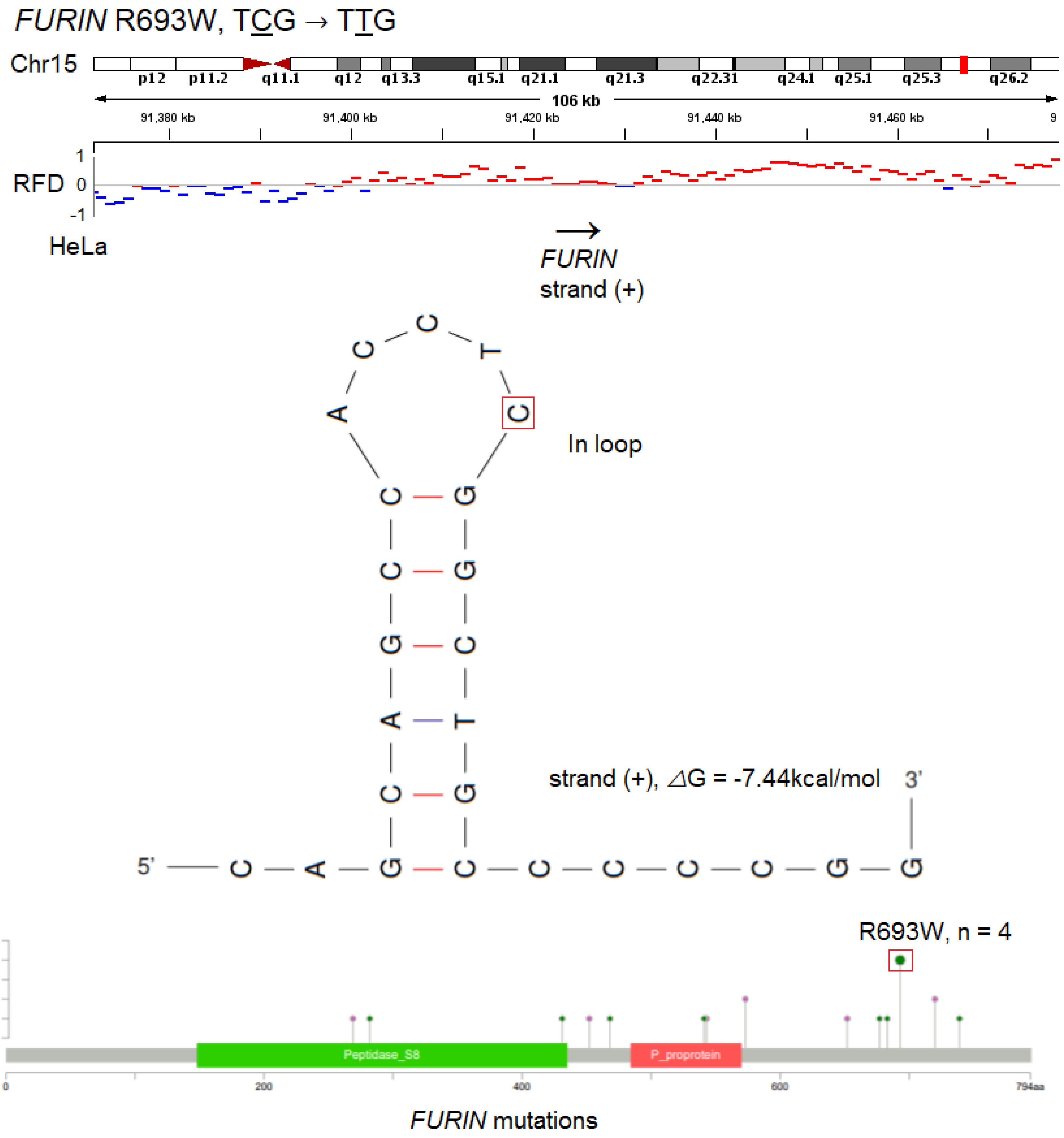

FURIN R693W, TCG → TTG
Chr15
p12
p11.2
q11.1
q12
q13.3
q15.1
q21.1
q21.3
q22.31
q24.1
q25.1
q25.3
q26.2
106 kb
91,380 kb
91,400 kb
91,420 kb
91,440 kb
91,460 kb
RFD
1
0
-1
HeLa
FURIN
strand (+)
In loop
strand (+), △G = -7.44kcal/mol
5'
3'
R693W, n = 4
Frequency
5
0
Peptidase_S8
P_proprotein
0
200
400
600
794aa
FURIN mutations

AL

KDM6A S1061*, TCA → TGA

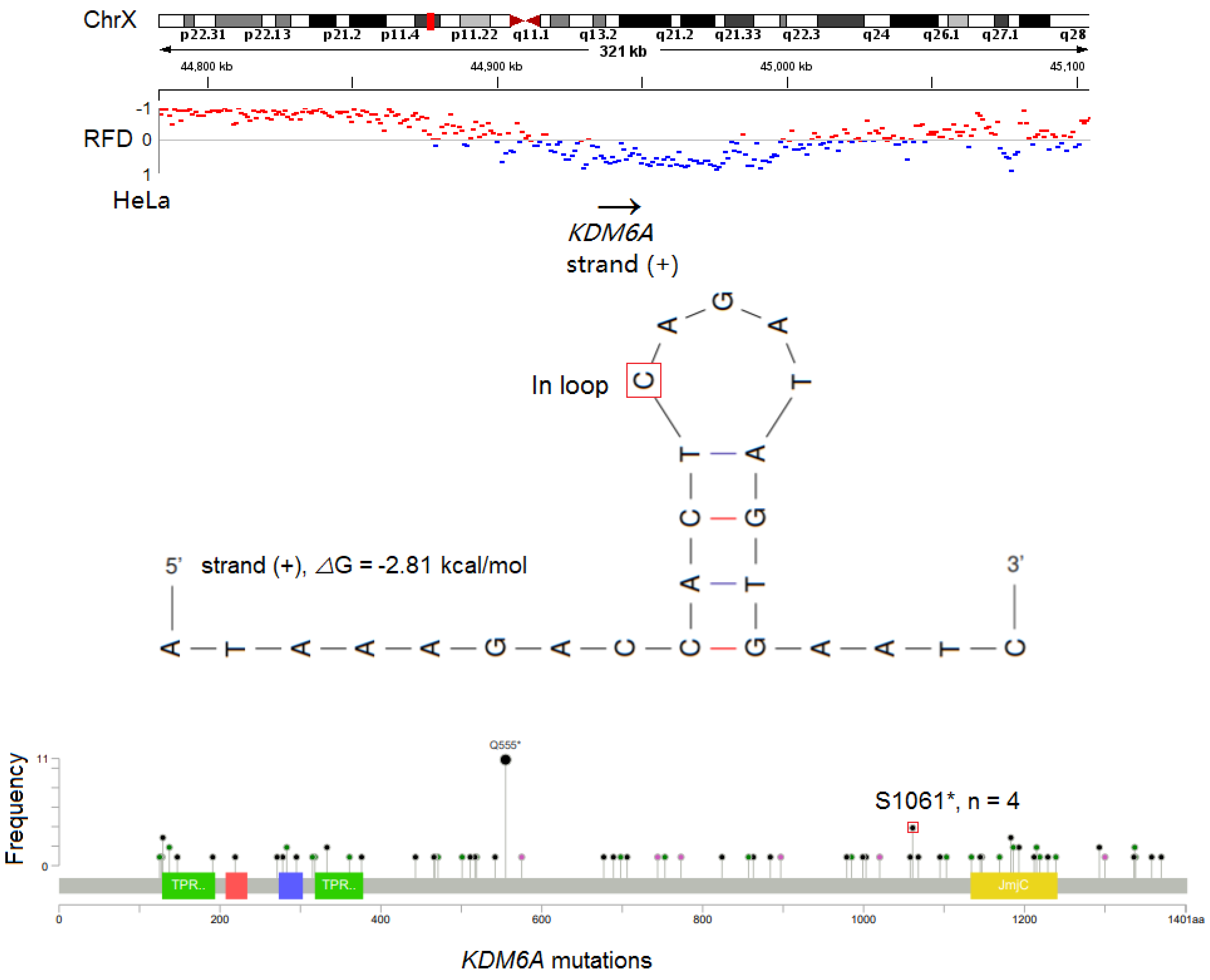

AM

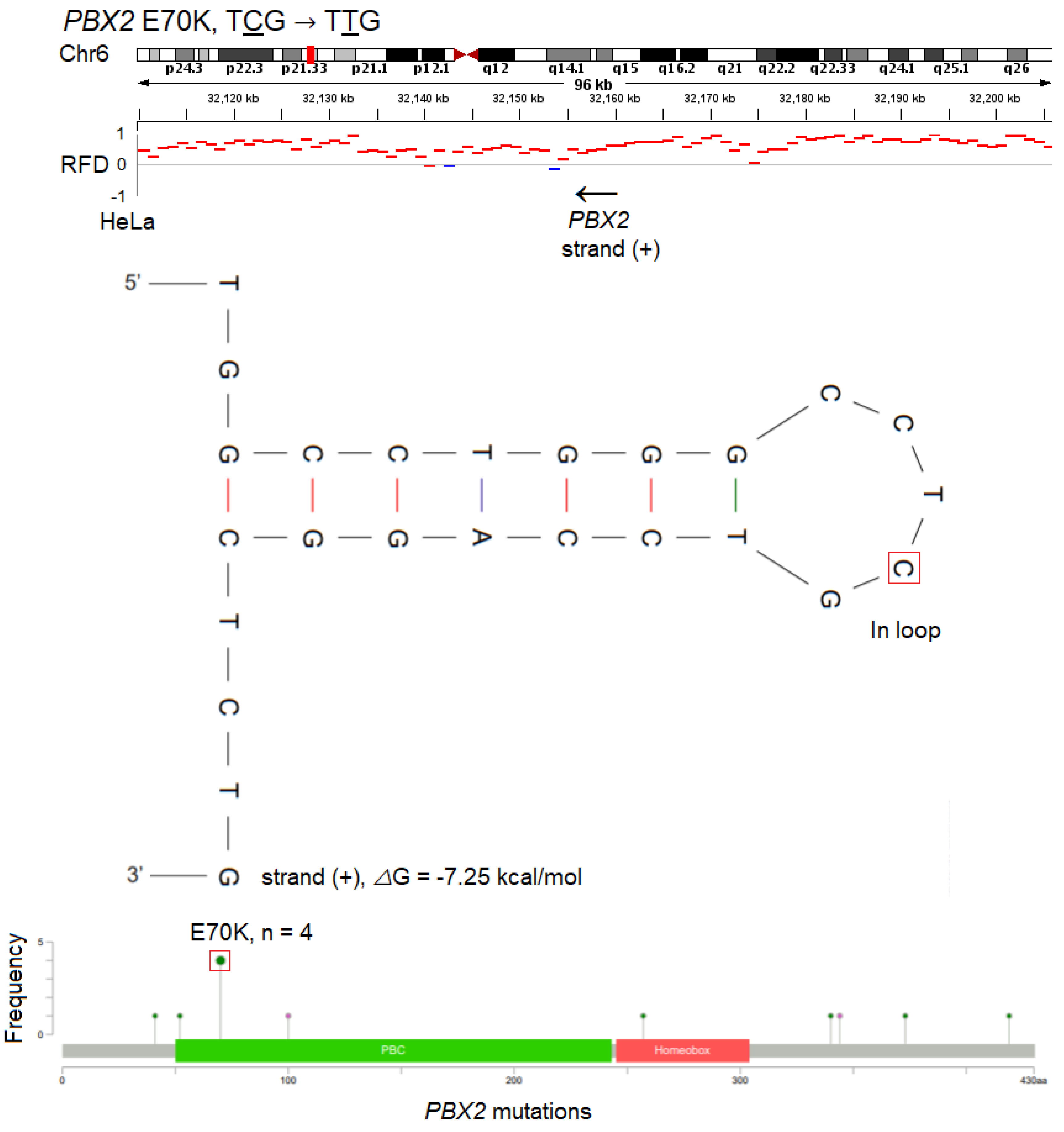

PBX2 E70K, TCG → TTG
Chr6
p24.3
p22.3
p21.33
p21.1
p12.1
q12
q14.1
q15
q16.2
q21
q22.2
q22.33
q24.1
q25.1
q26
96 kb
32,120 kb
32,130 kb
32,140 kb
32,150 kb
32,160 kb
32,170 kb
32,180 kb
32,190 kb
32,200 kb
RFD
1
0
-1
HeLa
PBX2
strand (+)
5'
3'
In loop
strand (+), ⊿G = -7.25 kcal/mol
E70K, n = 4
Frequency
5
0
PBC
Homeobox
0
100
200
300
430aa
PBX2 mutations

AN

RB1 Q217*, TCA → TTA

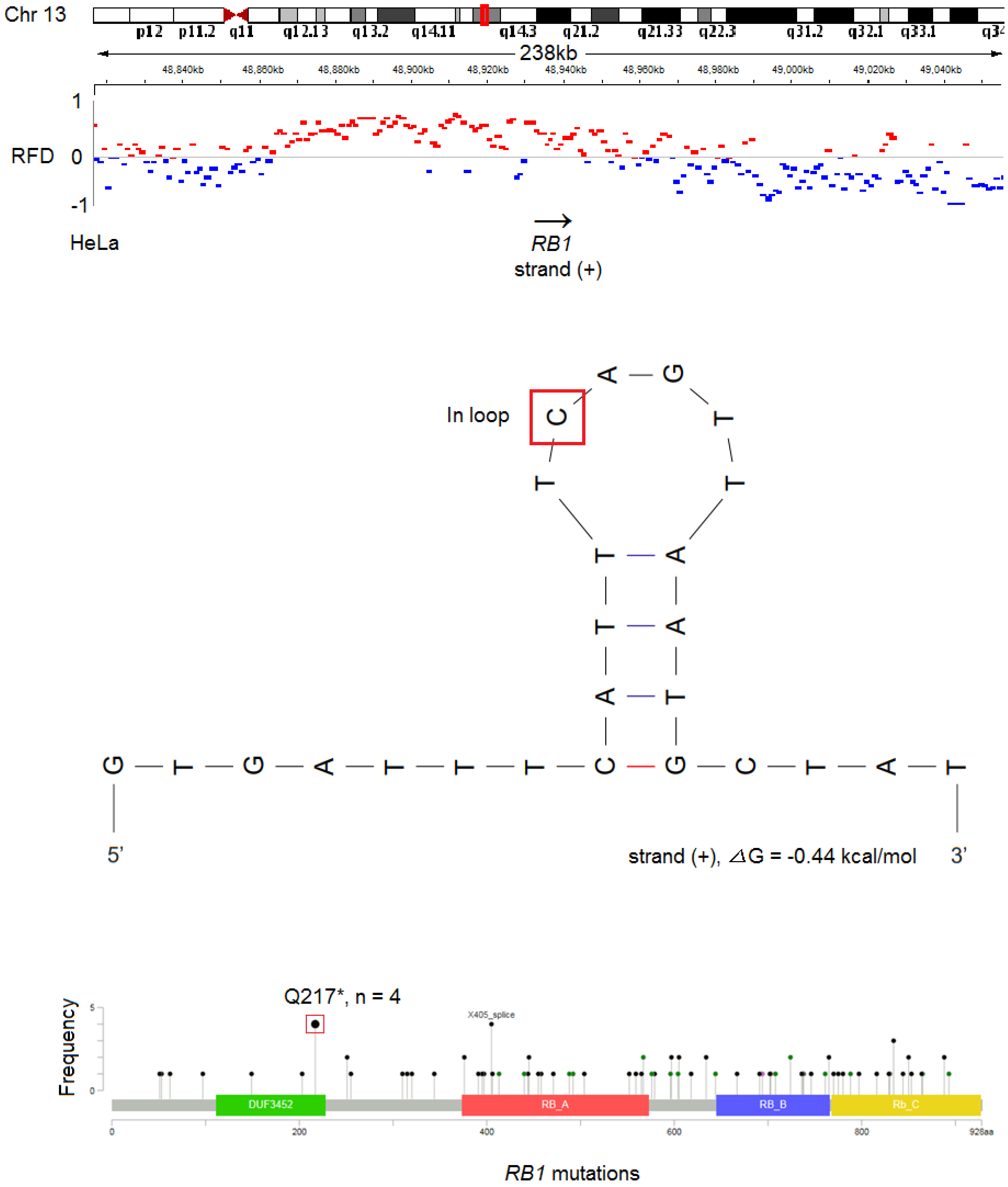

AO

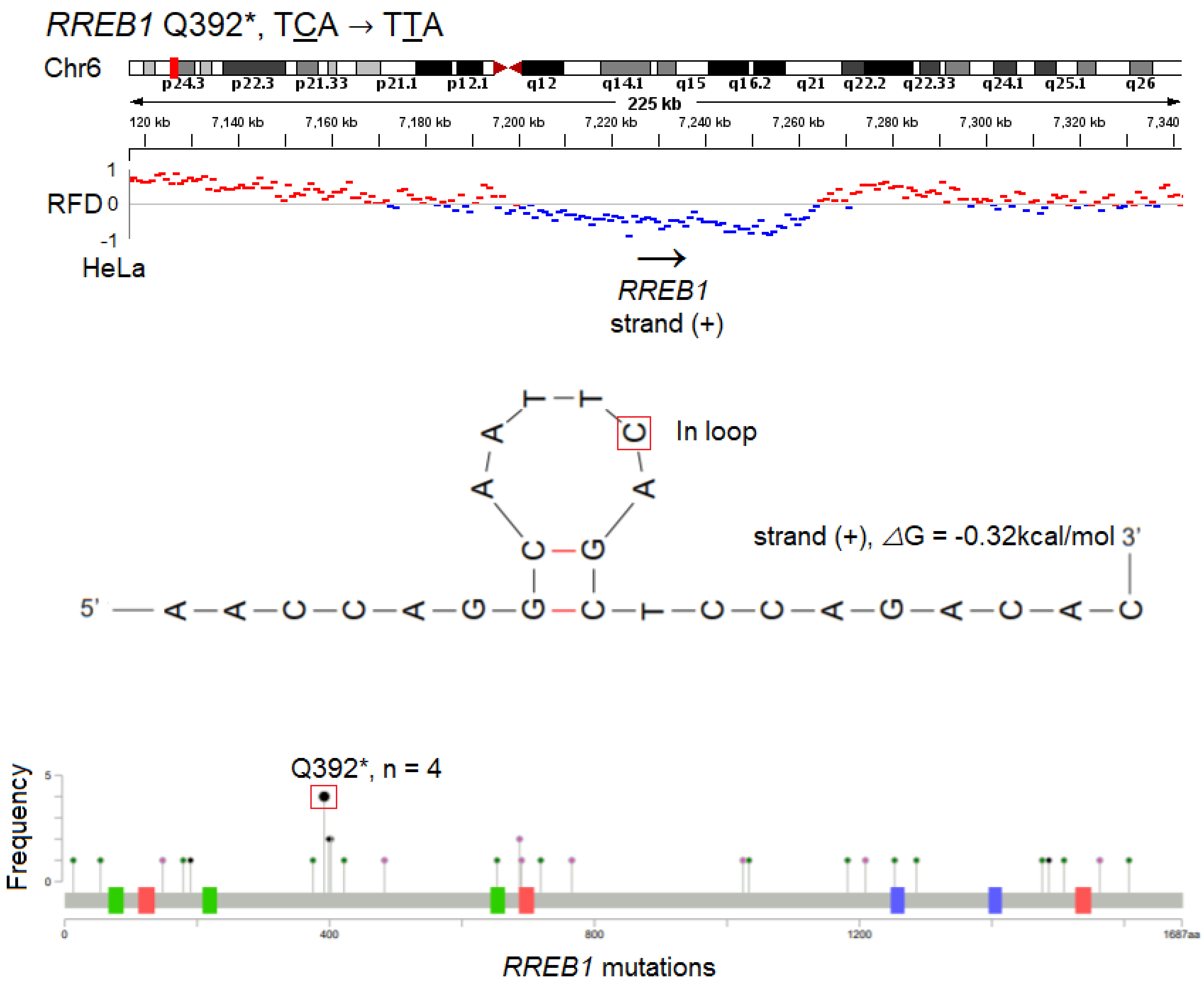
RREB1 Q392*, TCA → TTA
Chr6
p24.3
p22.3
p21.33
p21.1
p12.1
q12
q14.1
q15
q16.2
q21
q22.2
q22.33
q24.1
q25.1
q26
225 kb
120 kb
7,140 kb
7,160 kb
7,180 kb
7,200 kb
7,220 kb
7,240 kb
7,260 kb
7,280 kb
7,300 kb
7,320 kb
7,340
RFD
1
0
-1
HeLa
RREB1
strand (+)
In loop
strand (+), ΔG = -0.32kcal/mol
5'
3'
Q392*, n = 4
Frequency
5
0
400
800
1200
1687aa
RREB1 mutations

AP

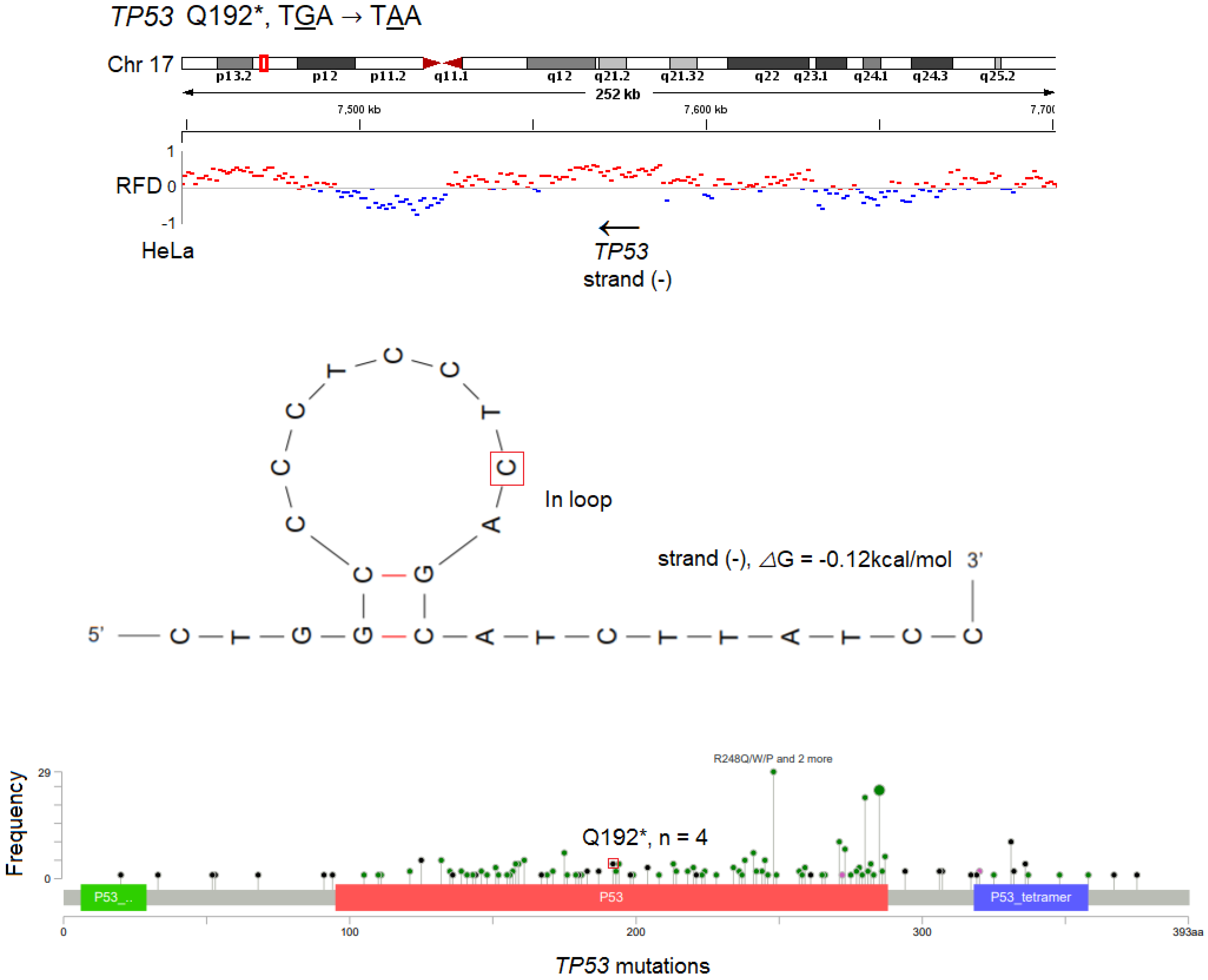

TP53 Q192*, TGA → TAA
Chr 17
p13.2
p12
p11.2
q11.1
q12
q21.2
q21.32
q22
q23.1
q24.1
q24.3
q25.2
252 kb
7,500 kb
7,600 kb
RFD
1
0
-1
HeLa
TP53
strand (-)
In loop
strand (-), △G = -0.12kcal/mol
5'
3'
C T G G C A T C T T A T C C
Frequency
29
0
R248Q/W/P and 2 more
Q192*, n = 4
P53_..
P53
P53_tetramer
0
100
200
300
393aa
TP53 mutations

AQ

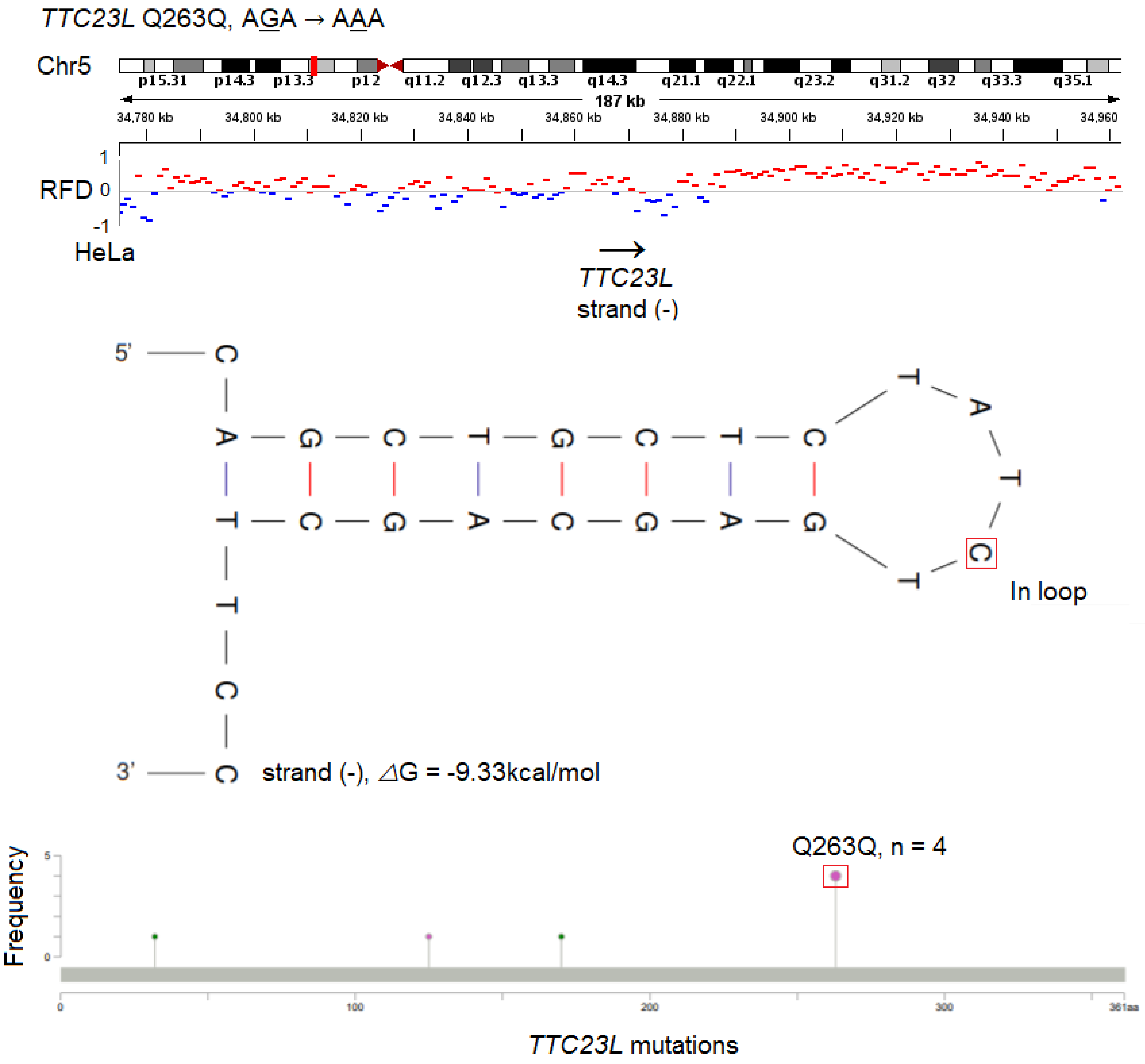

TTC23L Q263Q, AGA → AAA
Chr5
p15.31 p14.3 p13.3 p12 q11.2 q12.3 q13.3 q14.3 q21.1 q22.1 q23.2 q31.2 q32 q33.3 q35.1
187 kb
34,780 kb 34,800 kb 34,820 kb 34,840 kb 34,860 kb 34,880 kb 34,900 kb 34,920 kb 34,940 kb 34,960
RFD
1
0
-1
HeLa
TTC23L
strand (-)
5'
3'
In loop
strand (-), ΔG = -9.33kcal/mol
Q263Q, n = 4
Frequency
5
0
0
100
200
300
361aa
TTC23L mutations

Fig. S7

(Including 55 subfigures)

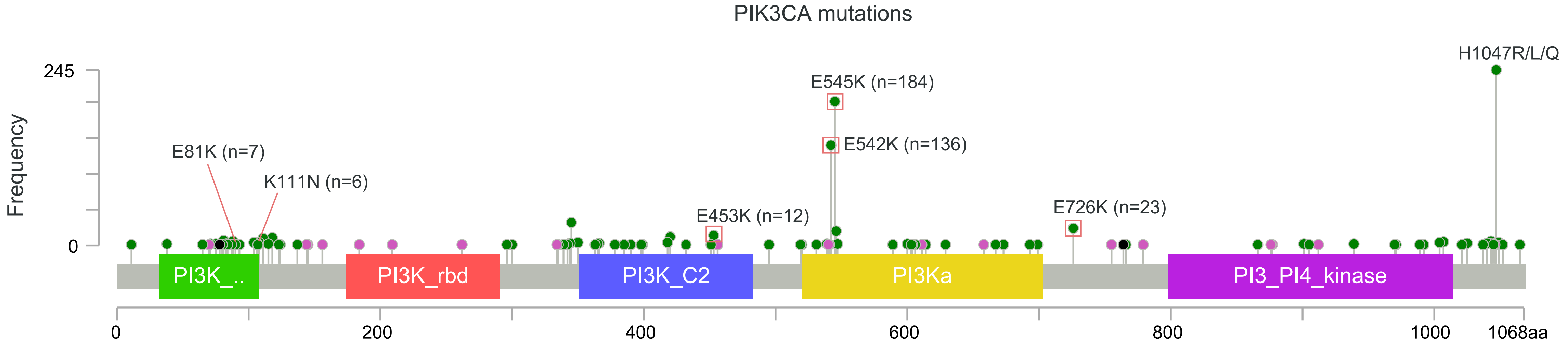

PIK3CA mutations
Frequency
245
0
E81K (n=7)
K111N (n=6)
E453K (n=12)
E545K (n=184)
E542K (n=136)
E726K (n=23)
H1047R/L/Q
PI3K_..
PI3K_rbd
PI3K_C2
PI3Ka
PI3_PI4_kinase
0
200
400
600
800
1000
1068aa

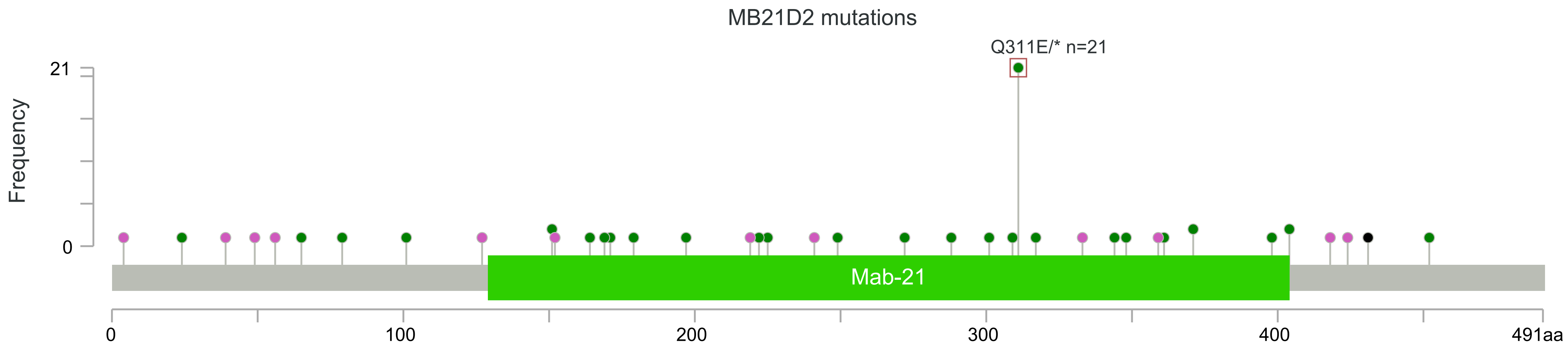

MB21D2 mutations
Q311E/* n=21
21
0
Frequency
Mab-21
0
100
200
300
400
491aa

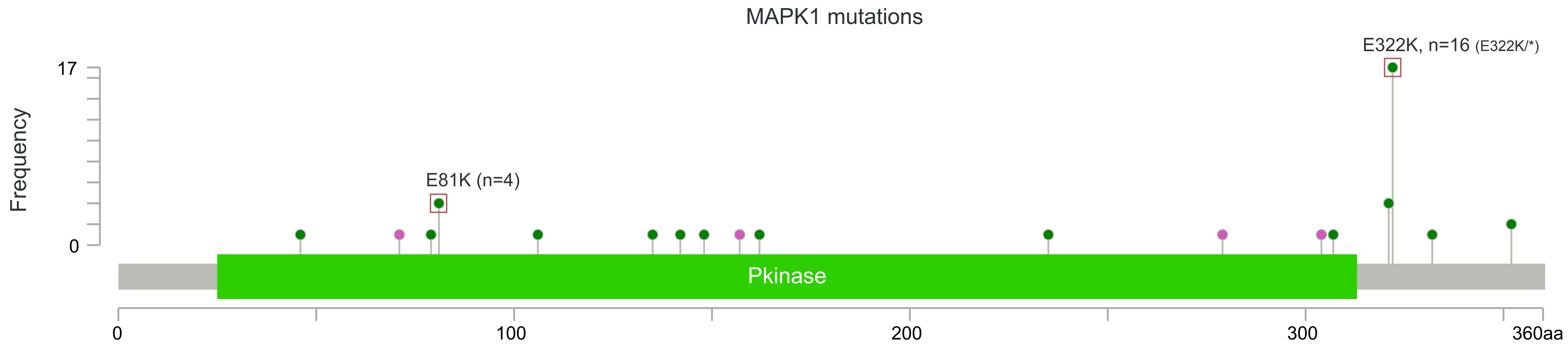
MAPK1 mutations
E322K, n=16 (E322K/*)
E81K (n=4)
17
0
Frequency
Pkinase
0
100
200
300
360aa

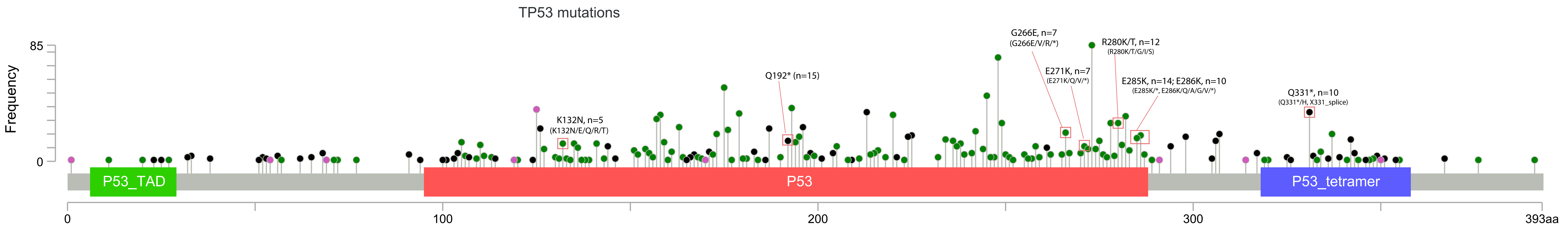

TP53 mutations
85
0
Frequency
K132N, n=5
(K132N/E/Q/R/T)
Q192* (n=15)
G266E, n=7
(G266E/V/R/*)
E271K, n=7
(E271K/Q/V/*)
R280K/T, n=12
(R280K/T/G/I/S)
E285K, n=14; E286K, n=10
(E285K/*, E286K/Q/A/G/V/*)
Q331*, n=10
(Q331*/H, X331_splice)
P53_TAD
P53
P53_tetramer
0
100
200
300
393aa

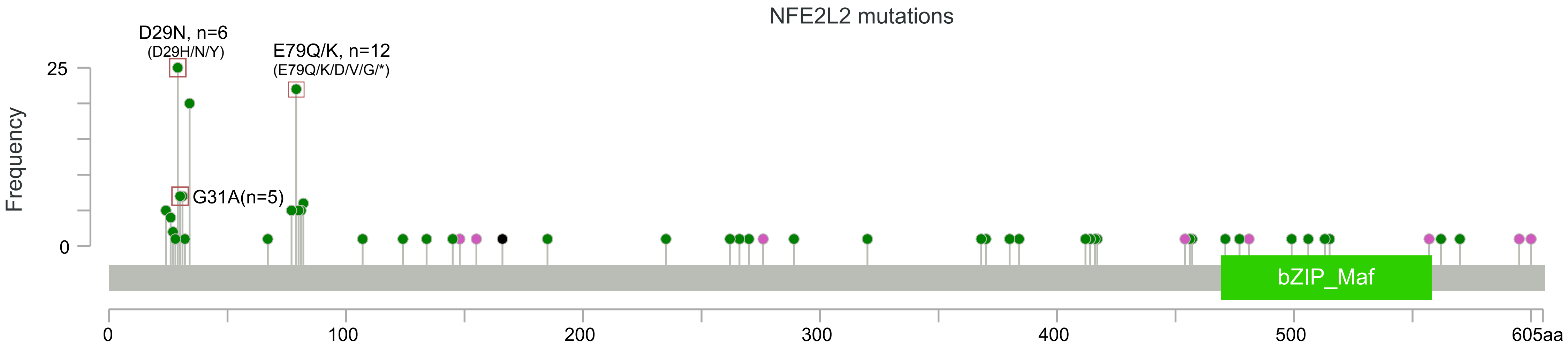
NFE2L2 mutations
D29N, n=6
(D29H/N/Y)
E79Q/K, n=12
(E79Q/K/D/V/G/*)
G31A(n=5)
25
Frequency
0
bZIP_Maf
0
100
200
300
400
500
605aa

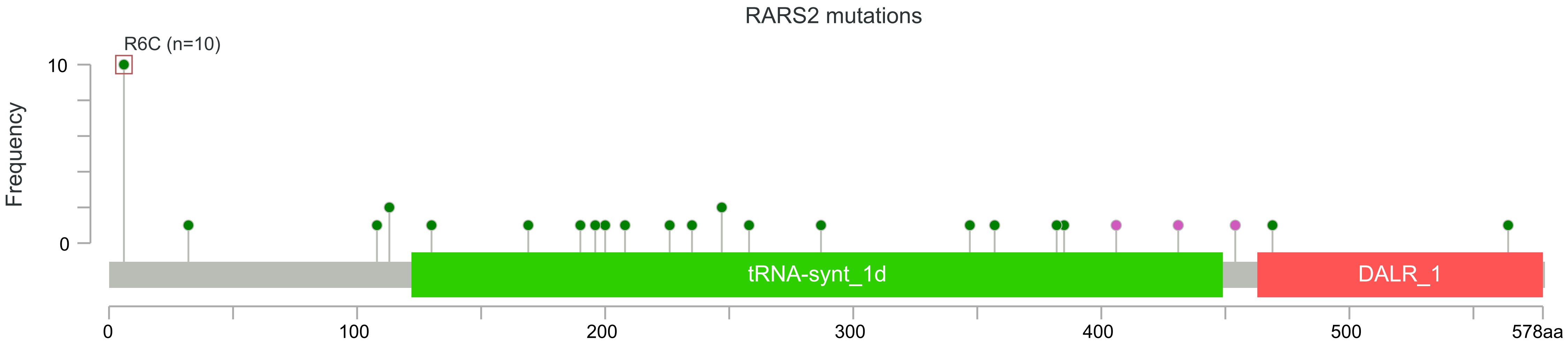

RARS2 mutations
R6C (n=10)
10
Frequency
0
tRNA-synt_1d
DALR_1
0
100
200
300
400
500
578aa

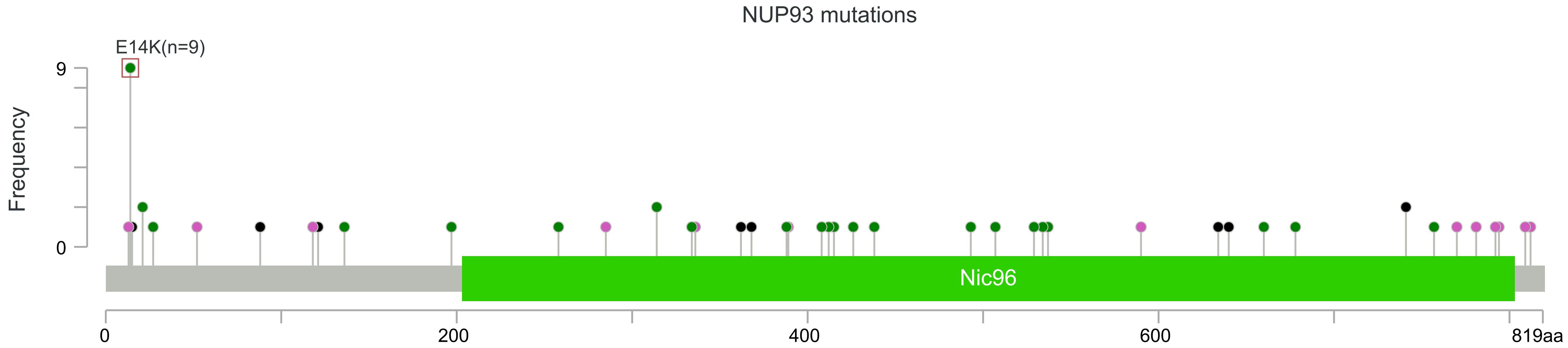

NUP93 mutations
E14K(n=9)
Frequency
9
0
Nic96
0
200
400
600
819aa

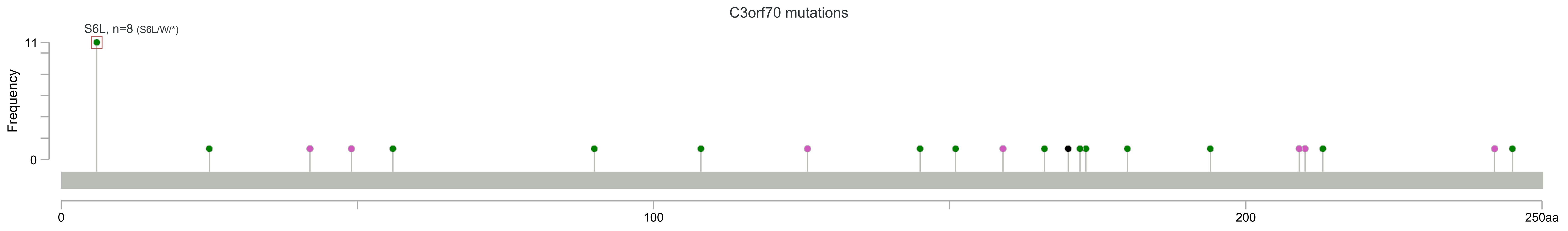
C3orf70 mutations
S6L, n=8 (S6L/W/*)
Frequency
11
0
0
100
200
250aa

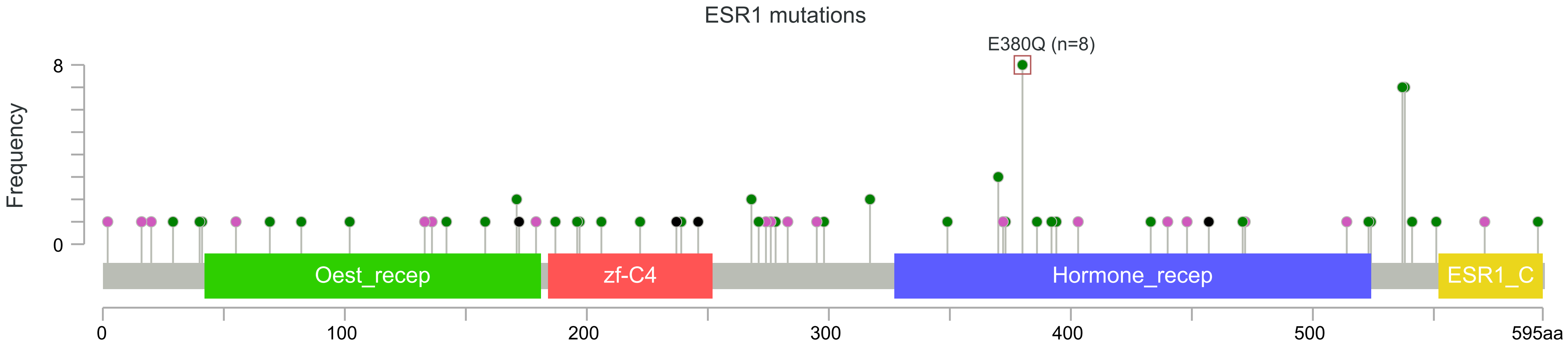

ESR1 mutations
E380Q (n=8)
Frequency
8
0
Oest_recep
zf-C4
Hormone_recep
ESR1_C
0
100
200
300
400
500
595aa

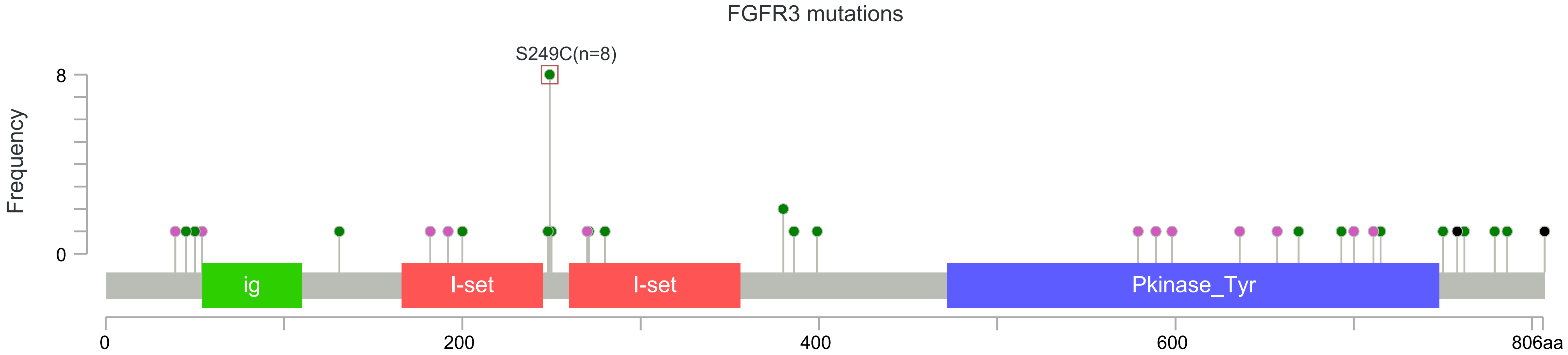

FGFR3 mutations
S249C(n=8)
Frequency
8
0
ig
I-set
I-set
Pkinase_Tyr
0
200
400
600
806aa

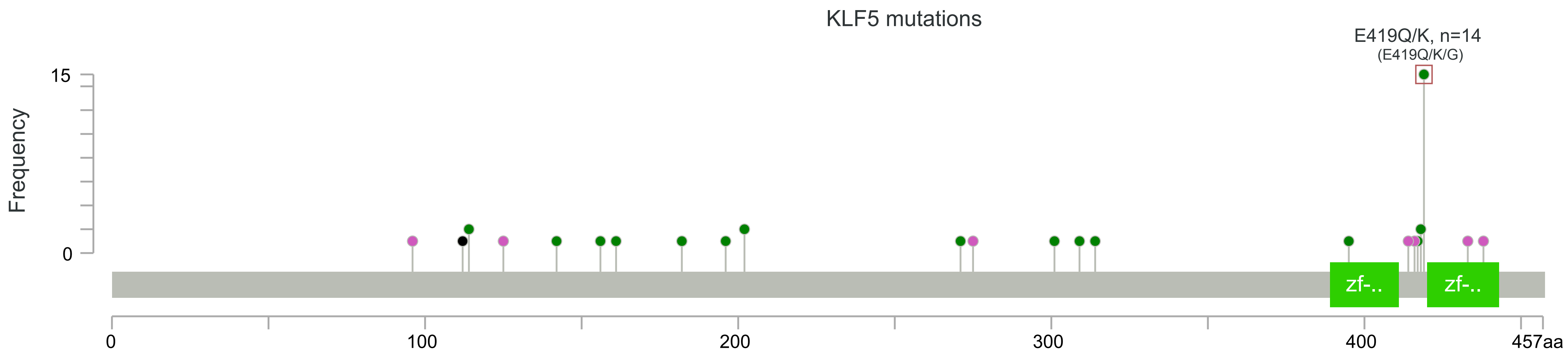

KLF5 mutations
E419Q/K, n=14
(E419Q/K/G)
15
0
Frequency
zf-..
zf-..
0
100
200
300
400
457aa

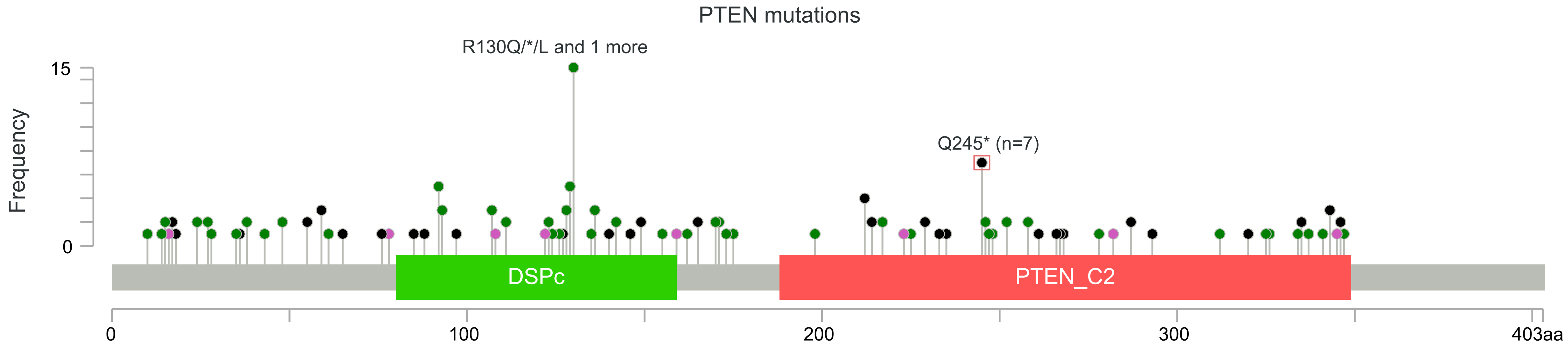

PTEN mutations
R130Q/*/L and 1 more
Q245* (n=7)
Frequency
15
0
DSPc
PTEN_C2
0
100
200
300
403aa

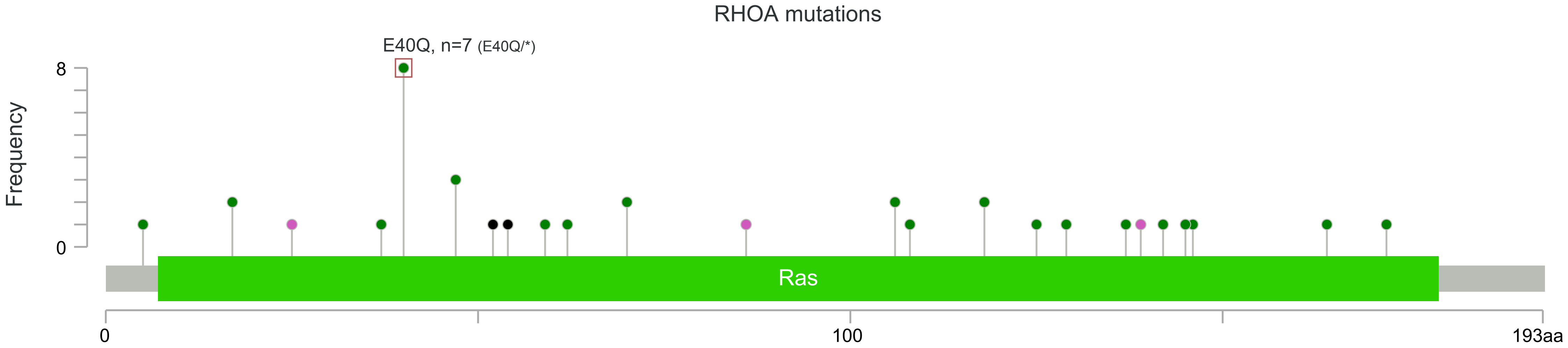
RHOA mutations
E40Q, n=7 (E40Q/*)
8
0
Frequency
Ras
0
100
193aa

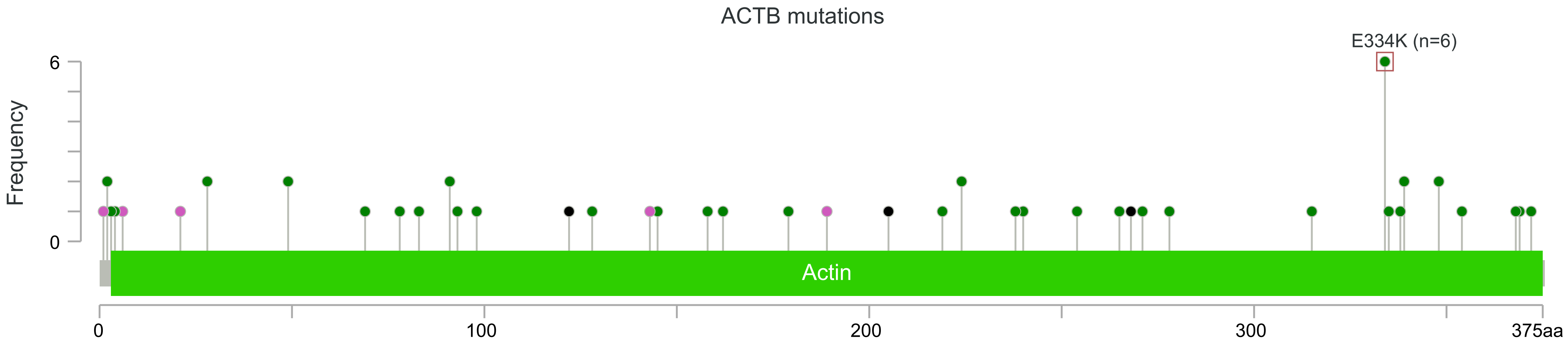

ACTB mutations
E334K (n=6)
Frequency
6
0
Actin
0
100
200
300
375aa

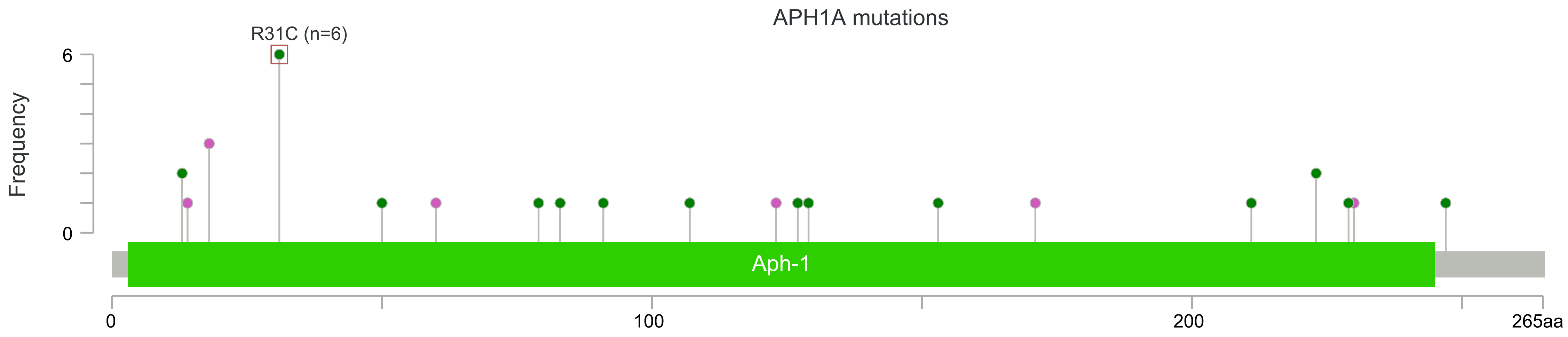
APH1A mutations
R31C (n=6)
Frequency
6
0
Aph-1
0
100
200
265aa

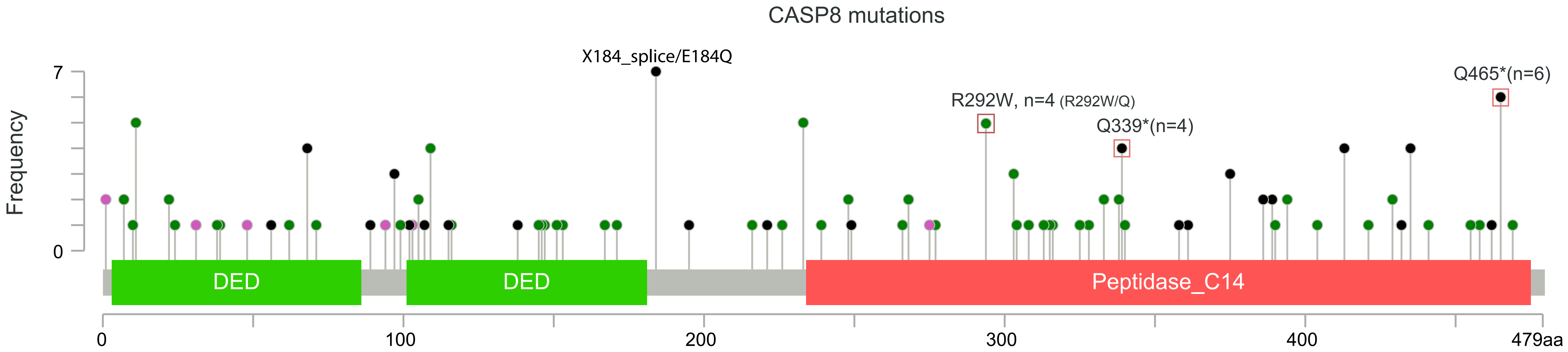

CASP8 mutations
X184_splice/E184Q
R292W, n=4 (R292W/Q)
Q339*(n=4)
Q465*(n=6)
7
0
Frequency
DED
DED
Peptidase_C14
0
100
200
300
400
479aa

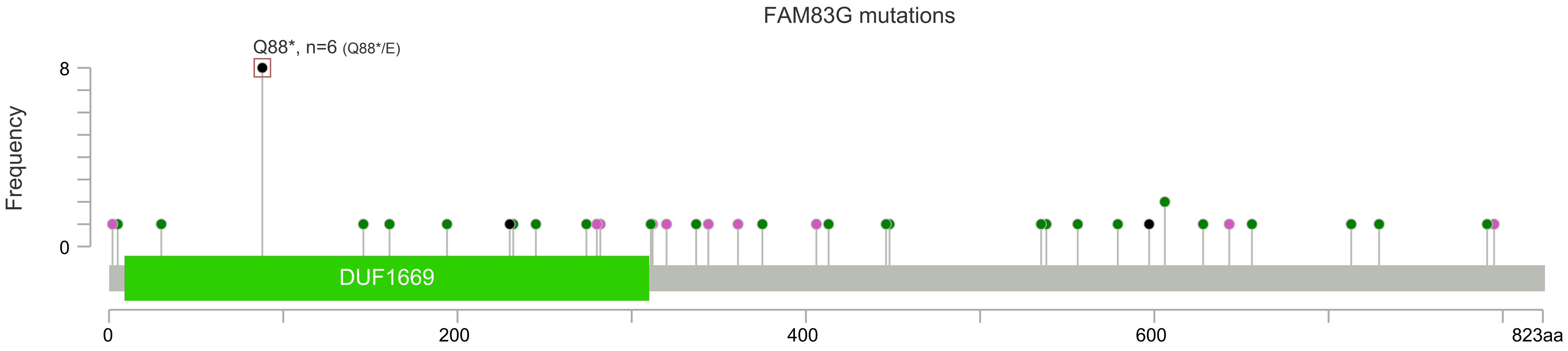

FAM83G mutations
Q88*, n=6 (Q88*/E)
Frequency
8
0
DUF1669
0
200
400
600
823aa

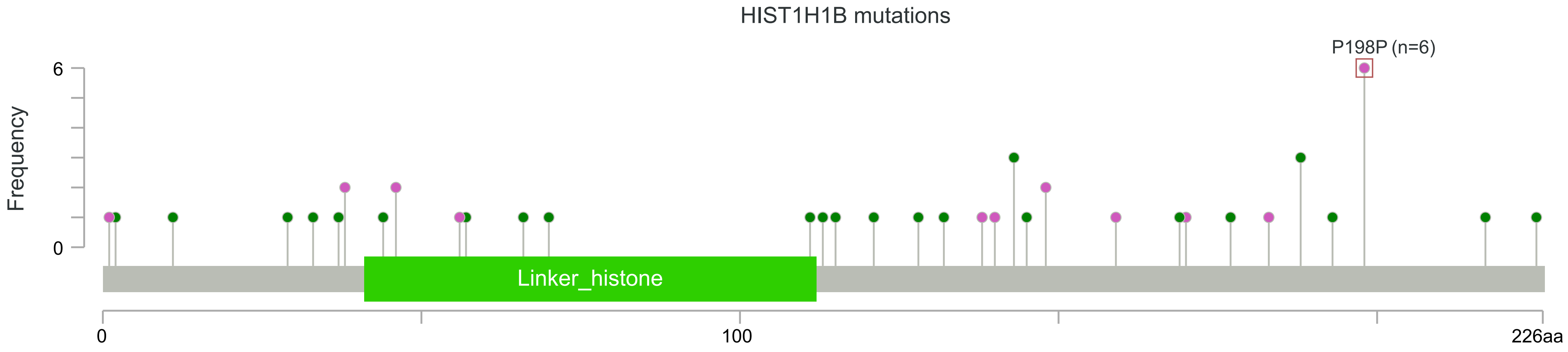

HIST1H1B mutations
P198P (n=6)
Frequency
6
0
Linker_histone
0
100
226aa

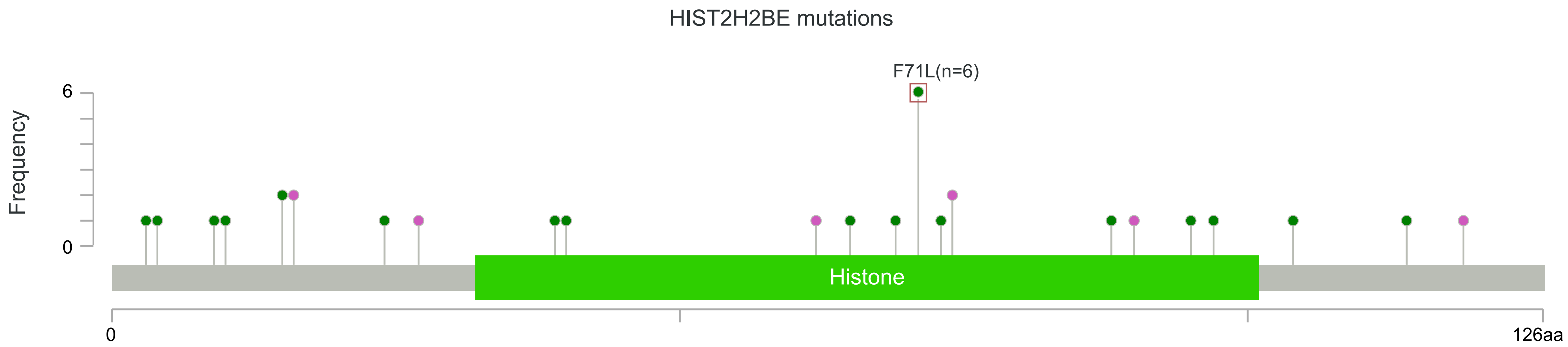

HIST2H2BE mutations
F71L(n=6)
6
0
Frequency
Histone
0
126aa

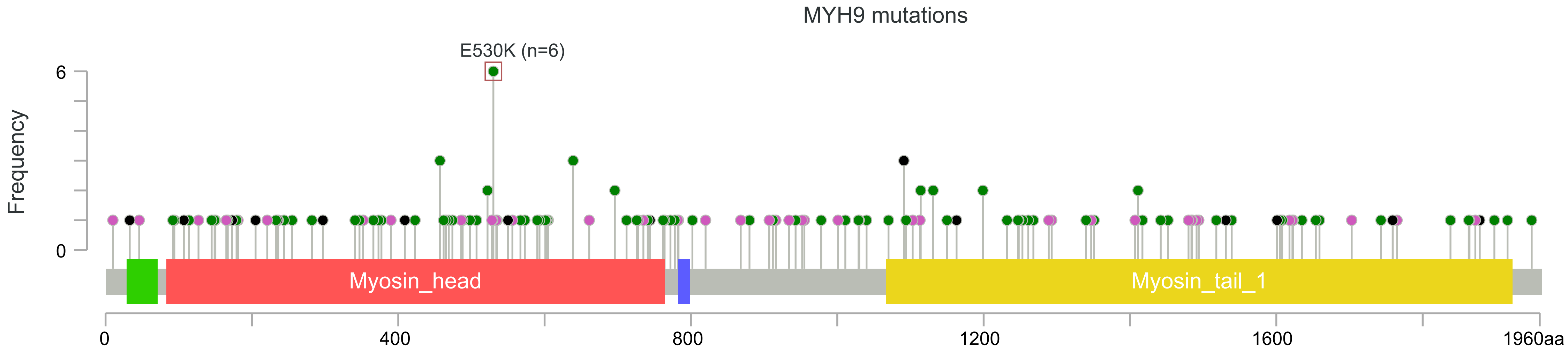
MYH9 mutations
E530K (n=6)
6
0
Frequency
Myosin_head
Myosin_tail_1
0
400
800
1200
1600
1960aa

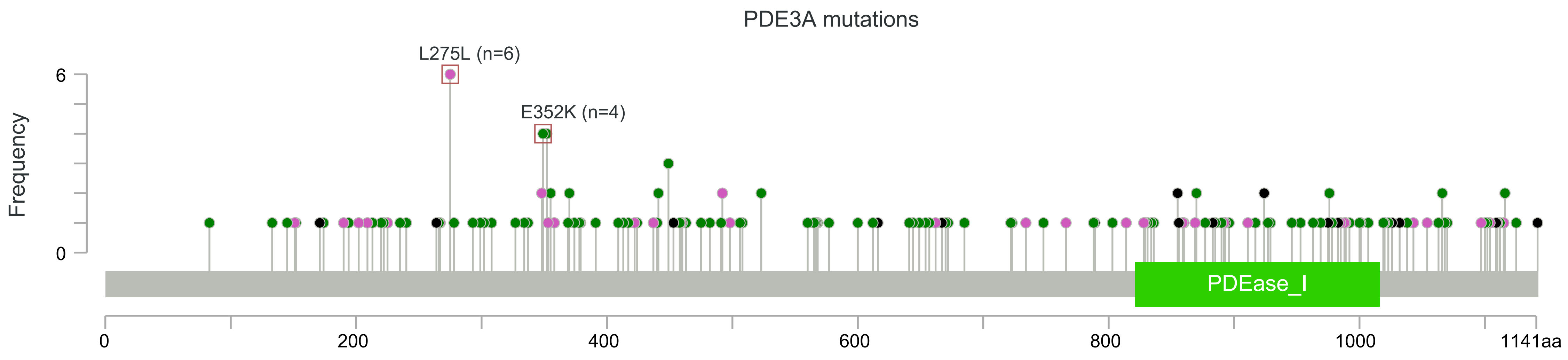

PDE3A mutations
L275L (n=6)
E352K (n=4)
Frequency
6
0
PDEase_I
0
200
400
600
800
1000
1141aa

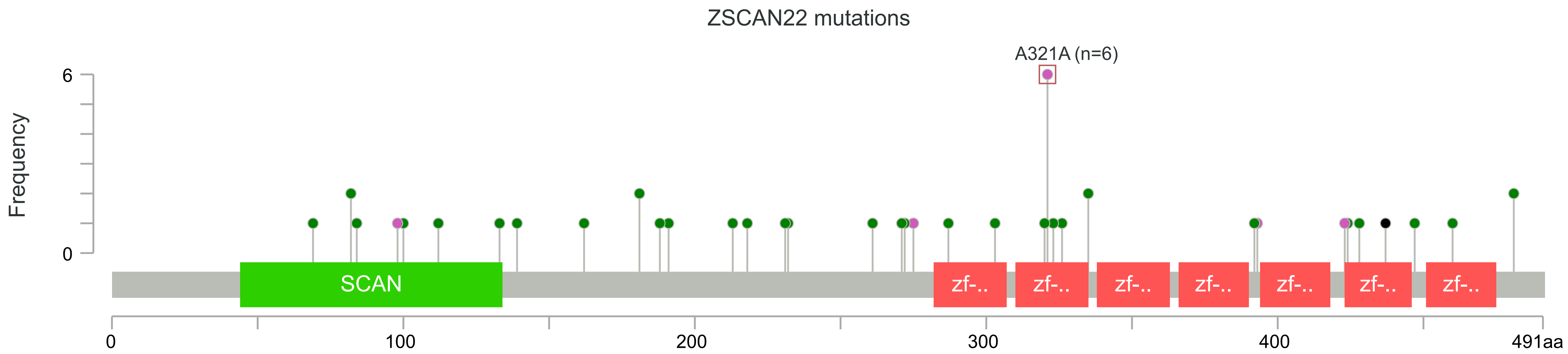
ZSCAN22 mutations
A321A (n=6)
Frequency
6
0
SCAN
zf-..
zf-..
zf-..
zf-..
zf-..
zf-..
zf-..
0
100
200
300
400
491aa

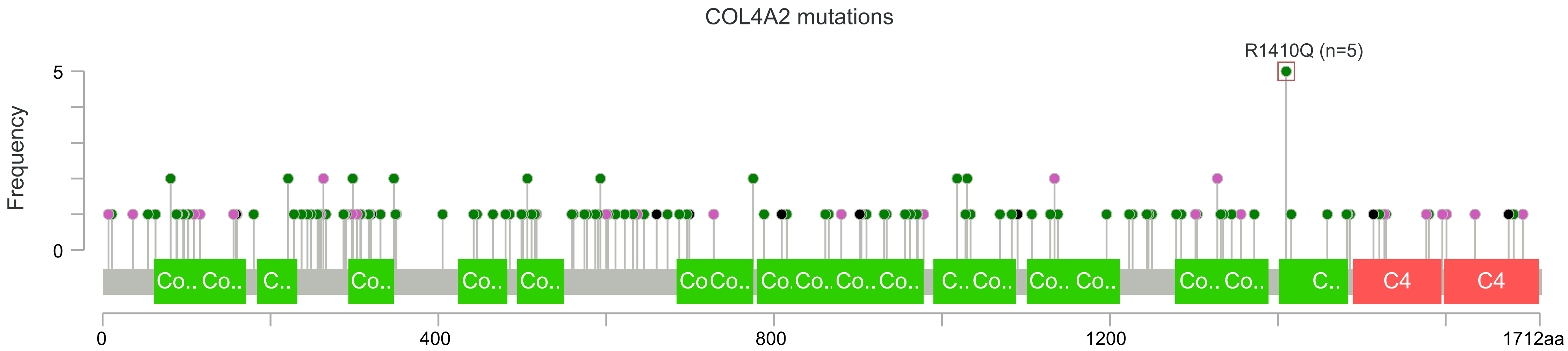

COL4A2 mutations
R1410Q (n=5)
Frequency
5
0
Co..
Co..
C..
Co..
Co..
Co..
Co
Co..
Co.
Co..
Co..
Co..
C..
Co..
Co..
Co..
Co..
Co..
C..
C4
C4
0
400
800
1200
1712aa

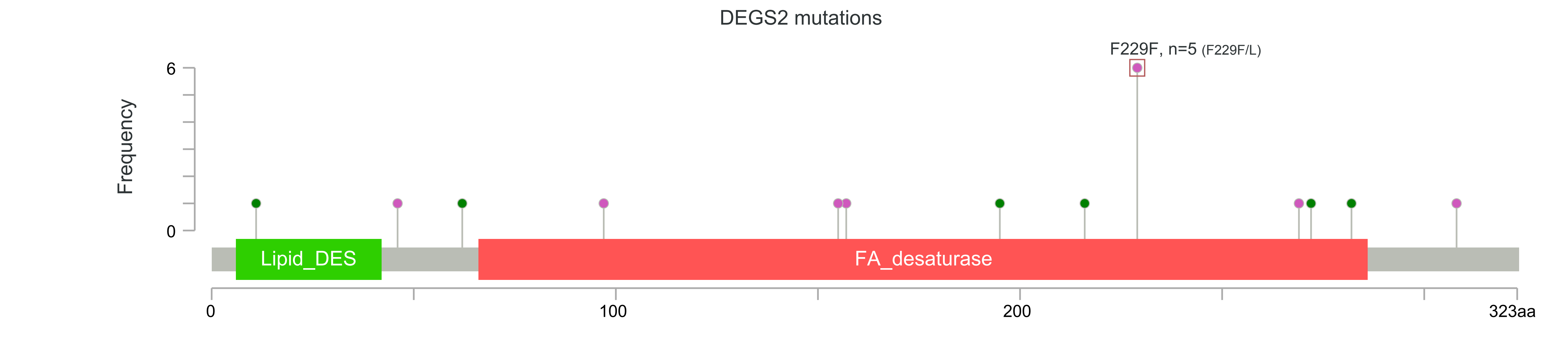

DEGS2 mutations
F229F, n=5 (F229F/L)
Frequency
6
0
Lipid_DES
FA_desaturase
0
100
200
323aa

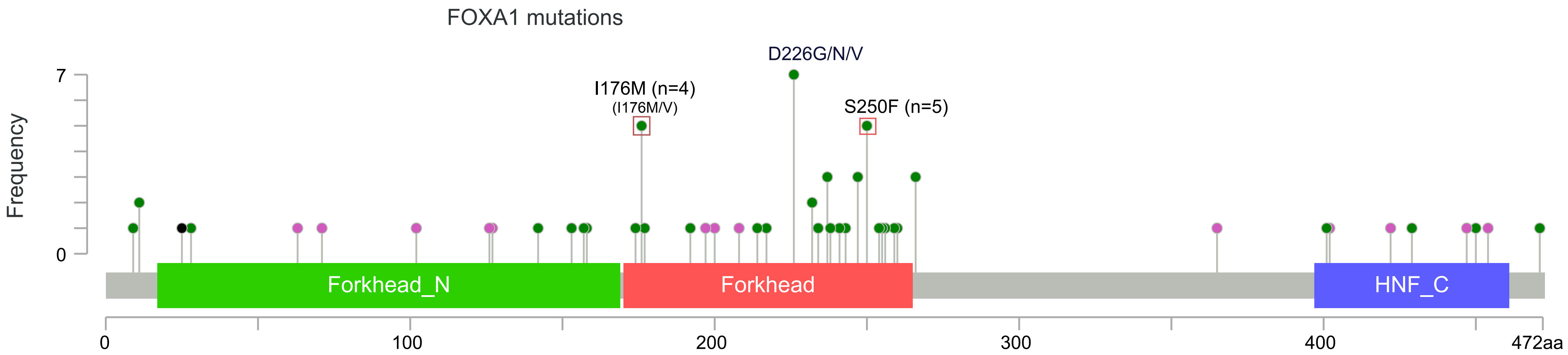
FOXA1 mutations
Frequency
7
0
D226G/N/V
I176M (n=4)
(I176M/V)
S250F (n=5)
Forkhead_N
Forkhead
HNF_C
0
100
200
300
400
472aa

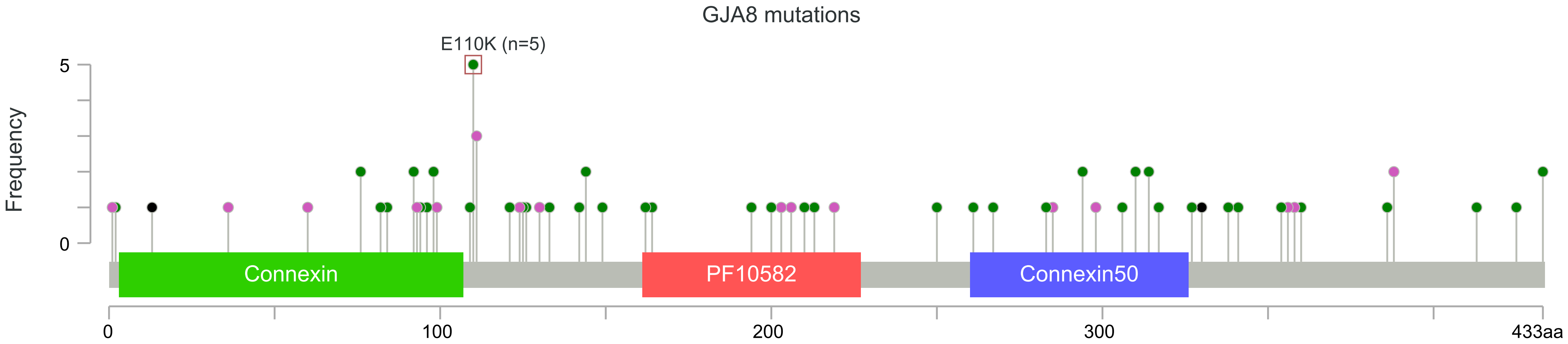
GJA8 mutations
E110K (n=5)
Frequency
5
0
Connexin
PF10582
Connexin50
0
100
200
300
433aa

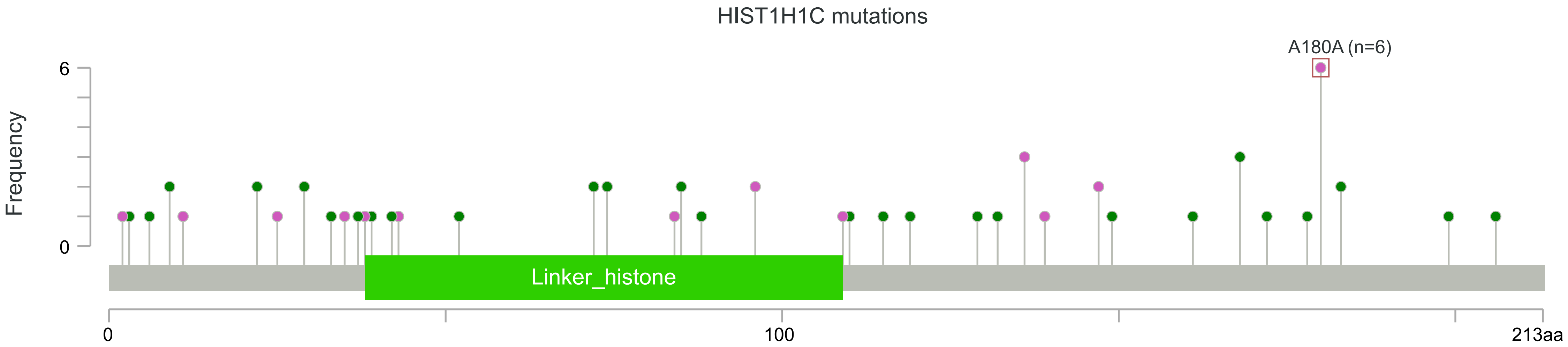

HIST1H1C mutations
A180A (n=6)
6
0
Frequency
Linker_histone
0
100
213aa

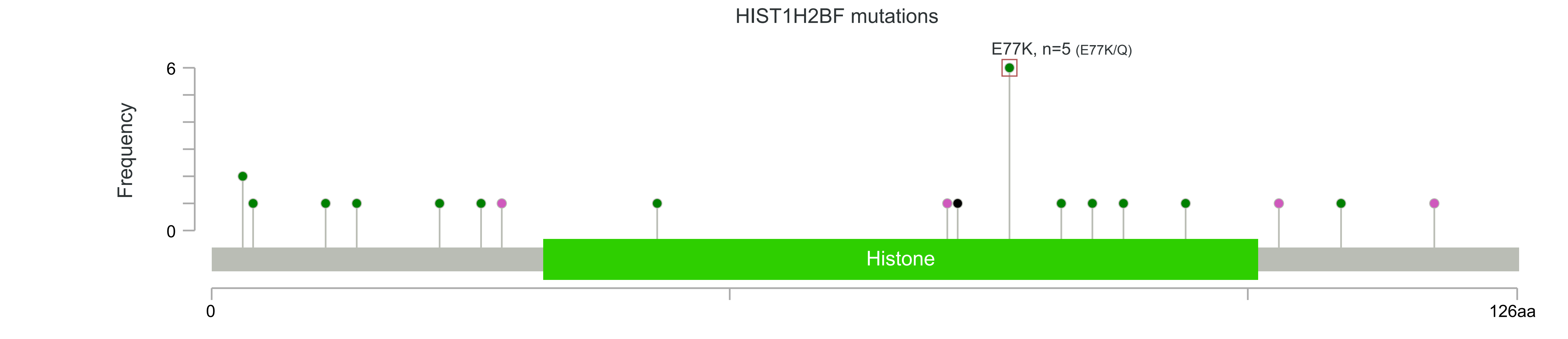

HIST1H2BF mutations
E77K, n=5 (E77K/Q)
6
0
Frequency
Histone
0
126aa

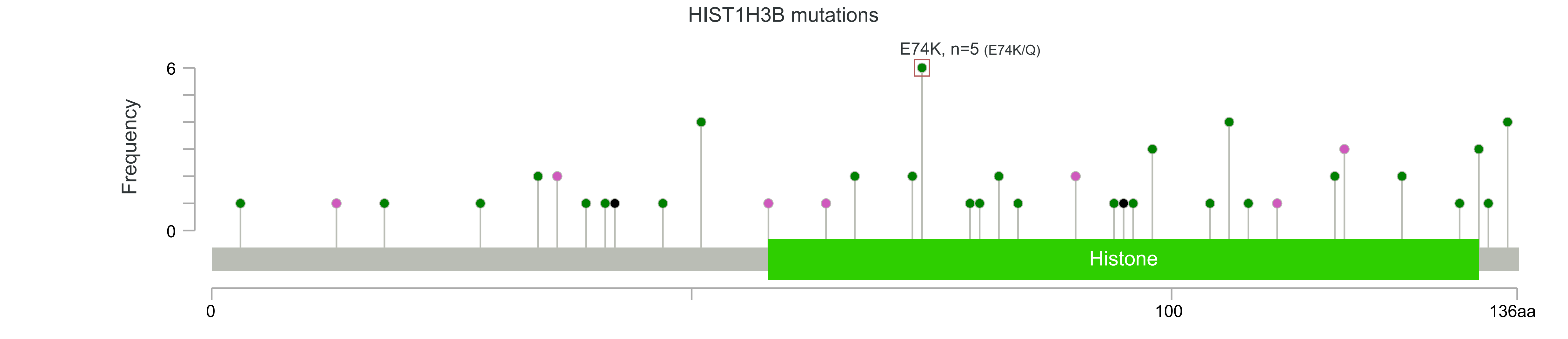
HIST1H3B mutations
E74K, n=5 (E74K/Q)
6
0
Frequency
Histone
0
100
136aa

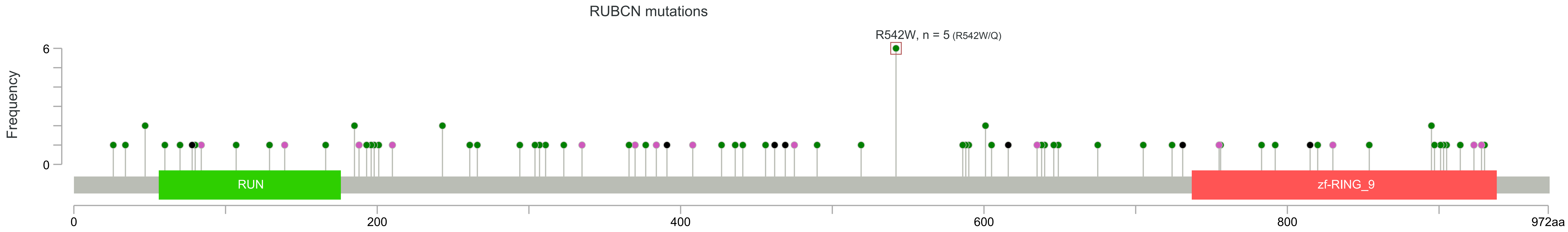
RUBCN mutations
R542W, n = 5 (R542W/Q)
6
0
Frequency
RUN
zf-RING_9
0
200
400
600
800
972aa

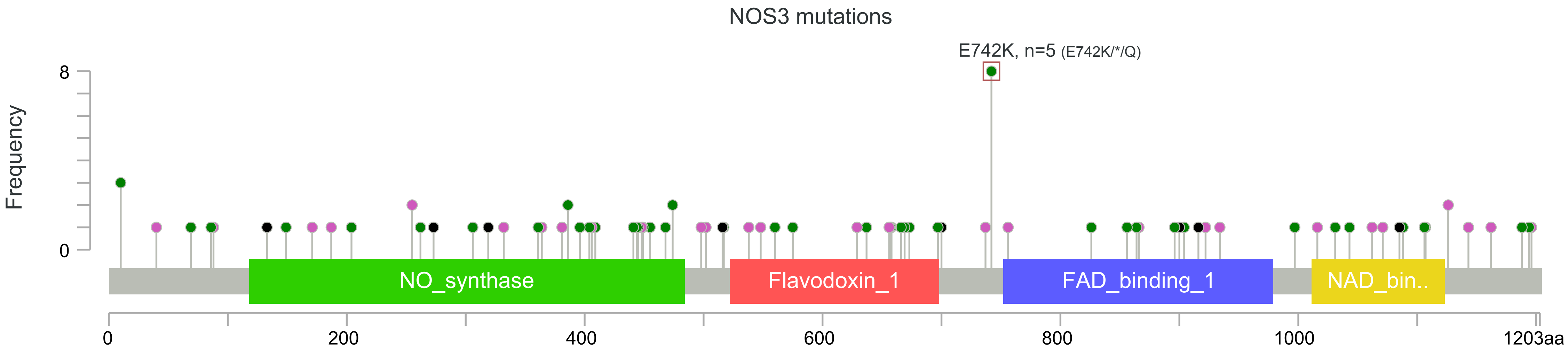

NOS3 mutations
E742K, n=5 (E742K/*/Q)
8
0
Frequency
NO_synthase
Flavodoxin_1
FAD_binding_1
NAD_bin..
0
200
400
600
800
1000
1203aa

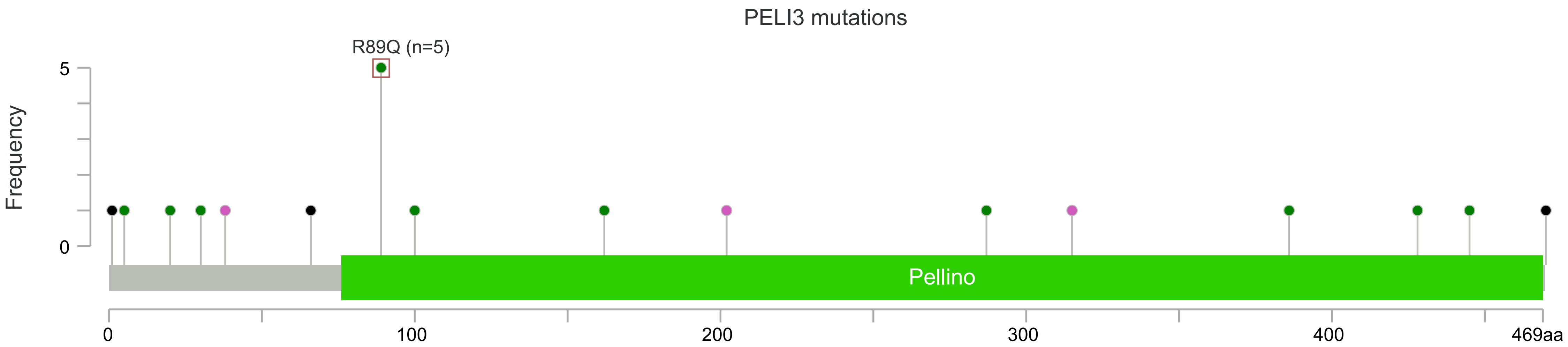
PELI3 mutations
R89Q (n=5)
Frequency
5
0
Pellino
0
100
200
300
400
469aa

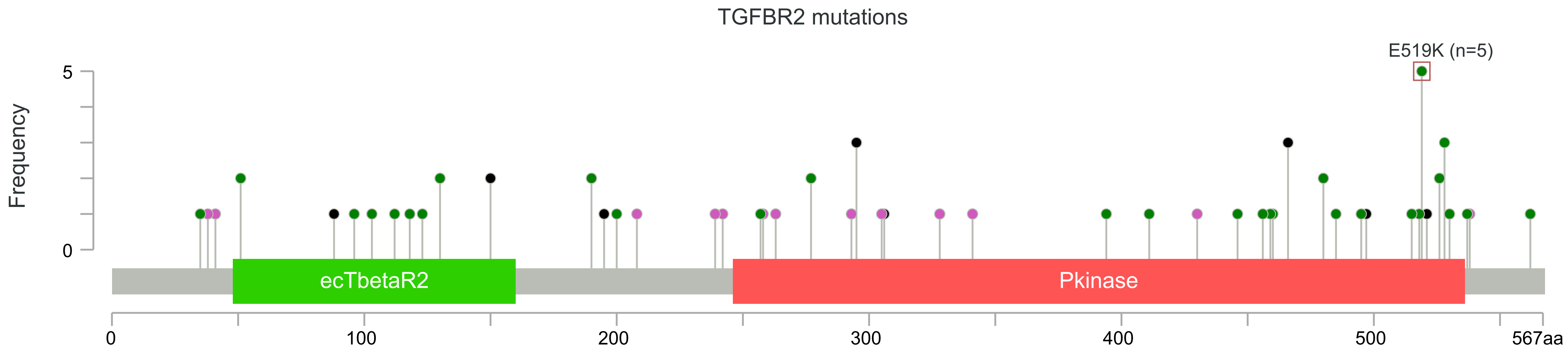

TGFBR2 mutations
E519K (n=5)
Frequency
5
0
ecTbetaR2
Pkinase
0
100
200
300
400
500
567aa

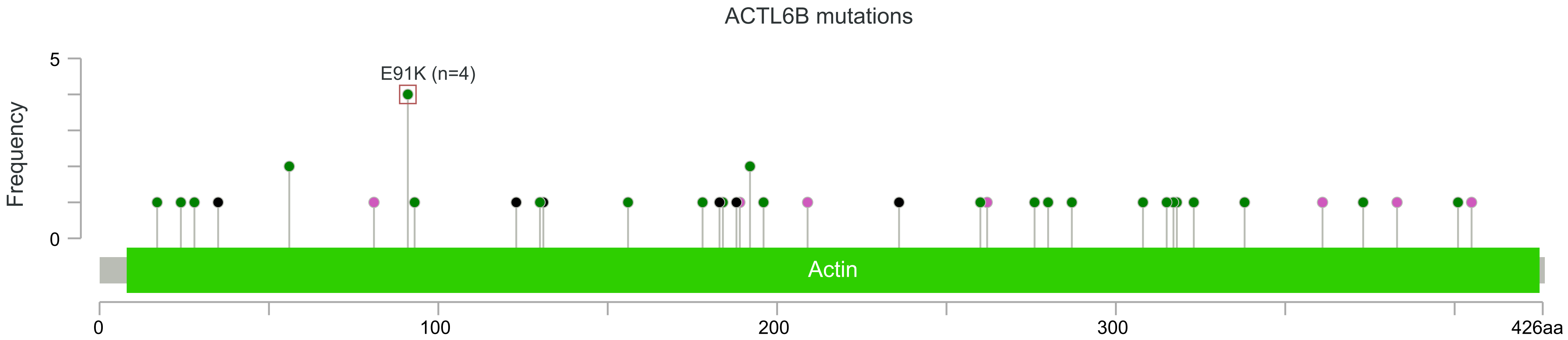

ACTL6B mutations
5
0
Frequency
E91K (n=4)
Actin
0
100
200
300
426aa

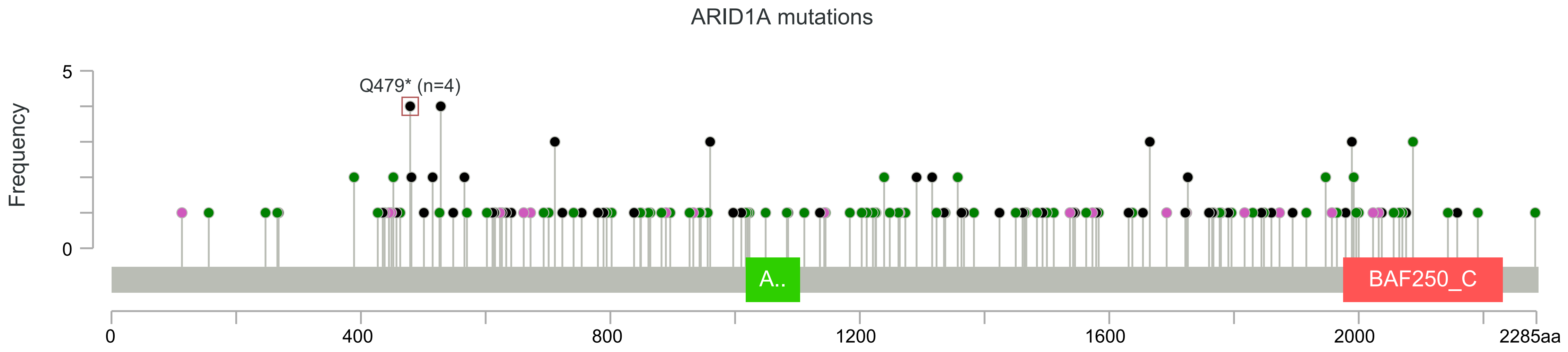

ARID1A mutations
Frequency
5
0
Q479* (n=4)
A..
BAF250_C
0
400
800
1200
1600
2000
2285aa

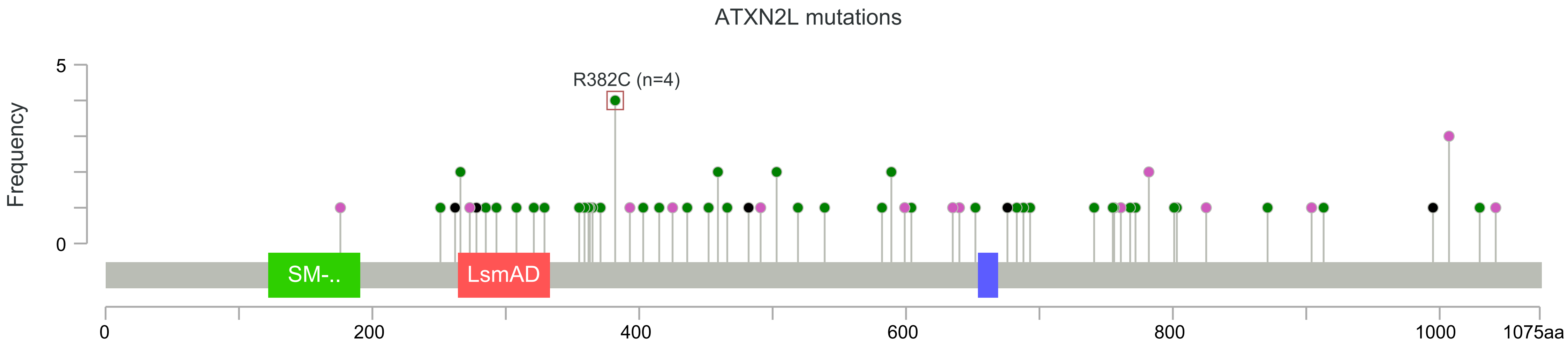
ATXN2L mutations
Frequency
5
0
R382C (n=4)
SM-..
LsmAD
0
200
400
600
800
1000
1075aa

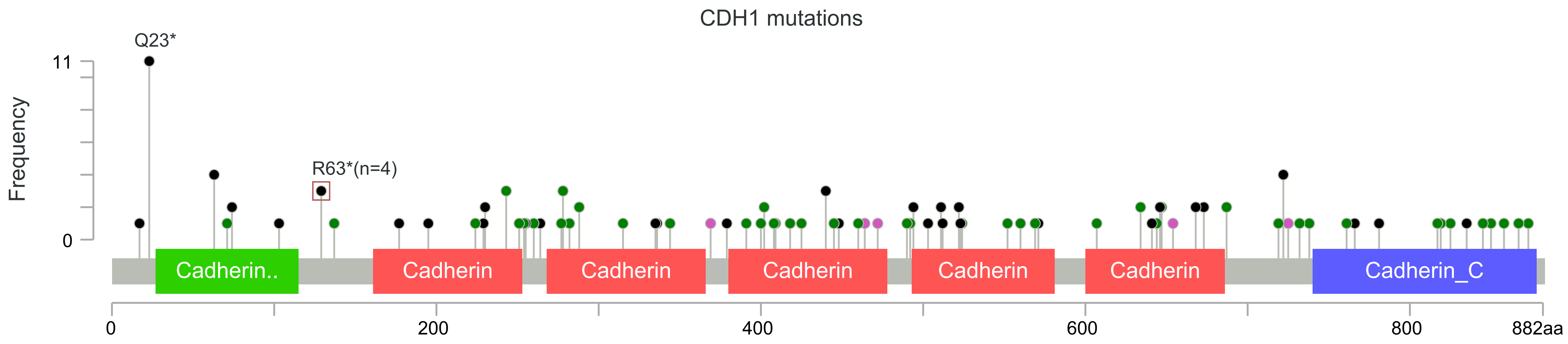

CDH1 mutations
Frequency
11
0
Q23*
R63*(n=4)
Cadherin..
Cadherin
Cadherin
Cadherin
Cadherin
Cadherin
Cadherin_C
0
200
400
600
800
882aa

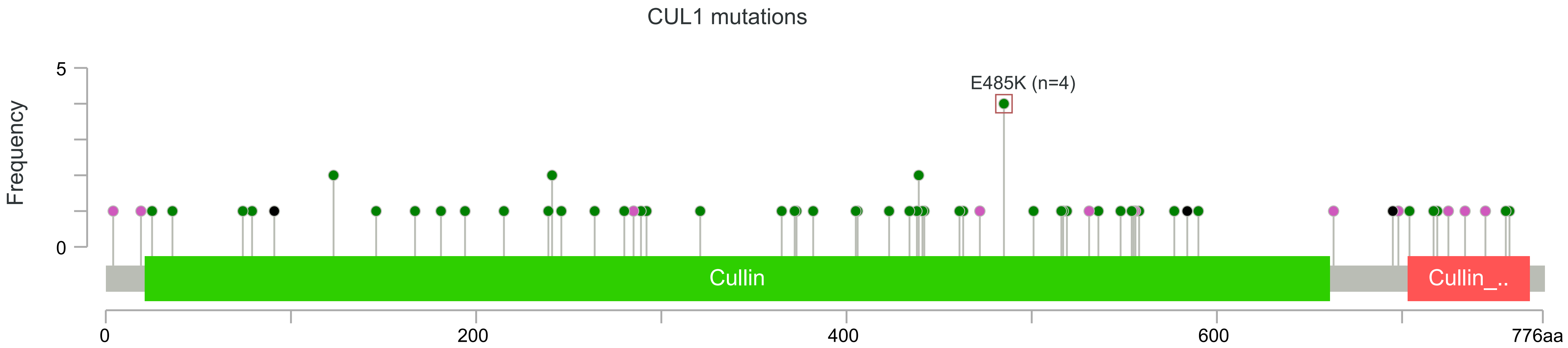

CUL1 mutations
Frequency
5
0
E485K (n=4)
Cullin
Cullin_..
0
200
400
600
776aa

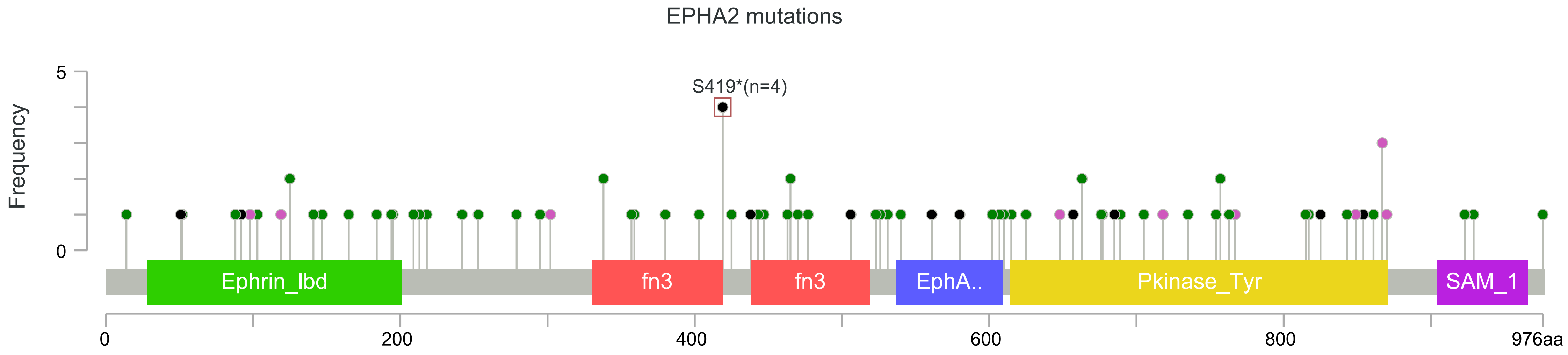

EPHA2 mutations
Frequency
5
0
S419*(n=4)
Ephrin_lbd
fn3
fn3
EphA..
Pkinase_Tyr
SAM_1
0
200
400
600
800
976aa

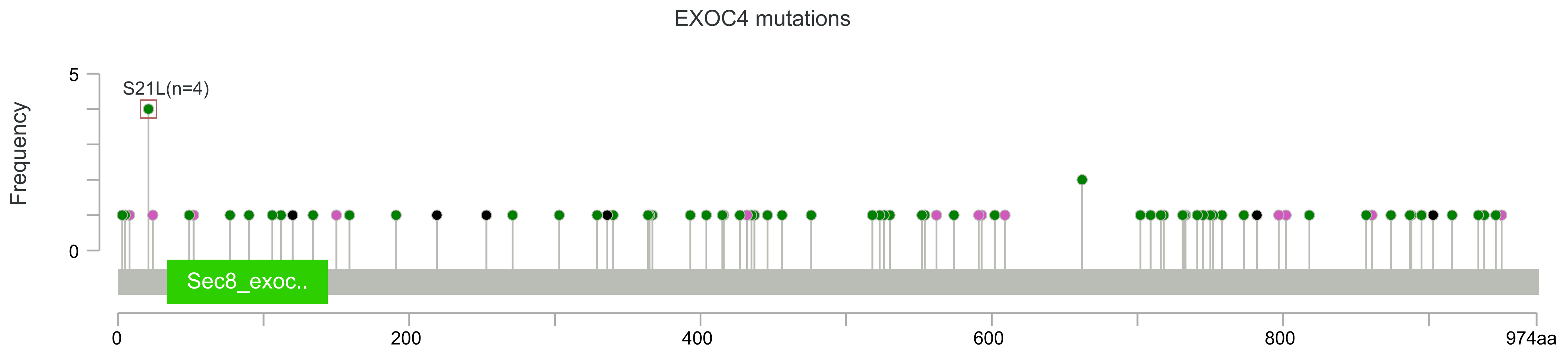
EXOC4 mutations
Frequency
5
0
S21L(n=4)
Sec8_exoc..
0
200
400
600
800
974aa

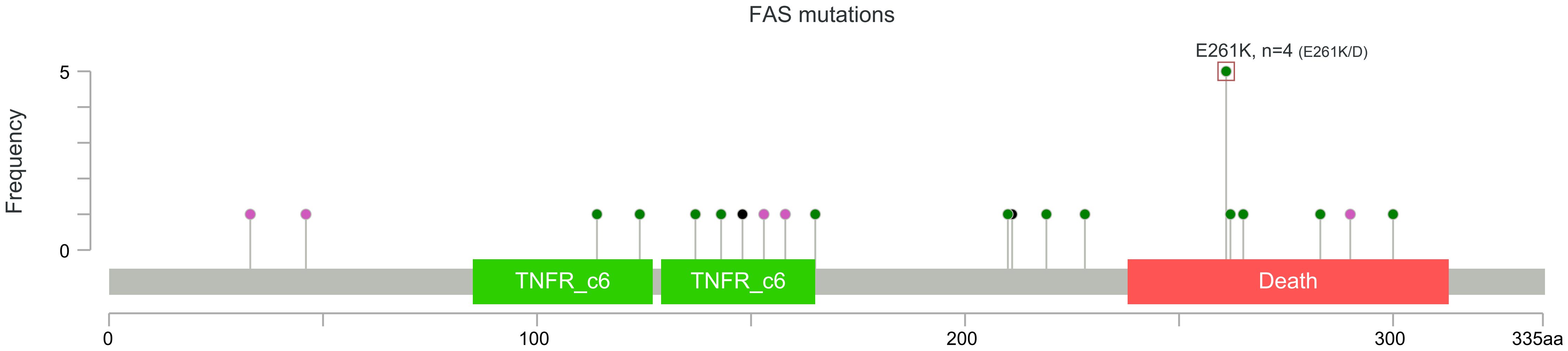
FAS mutations
E261K, n=4 (E261K/D)
Frequency
5
0
TNFR_c6
TNFR_c6
Death
0
100
200
300
335aa

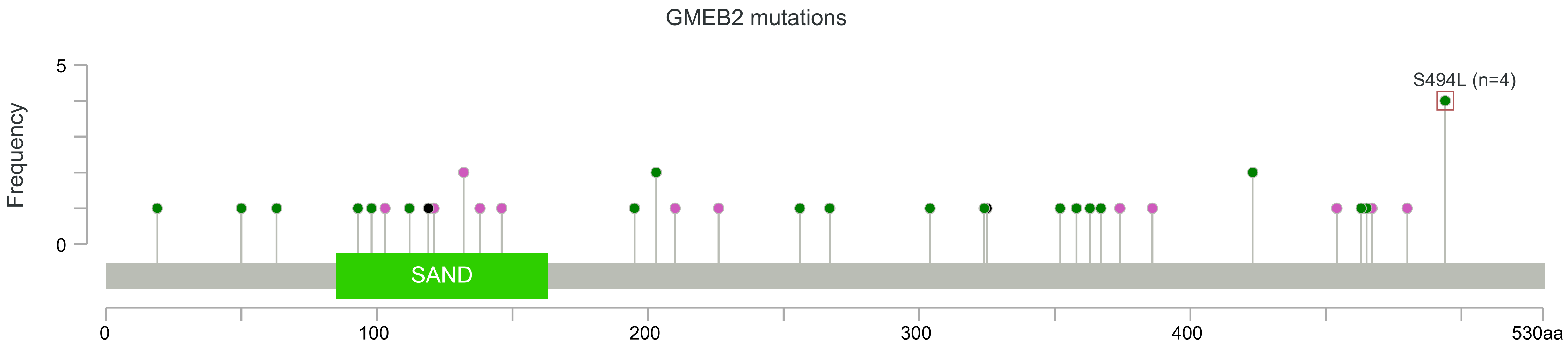

GMEB2 mutations
Frequency
5
0
S494L (n=4)
SAND
0
100
200
300
400
530aa

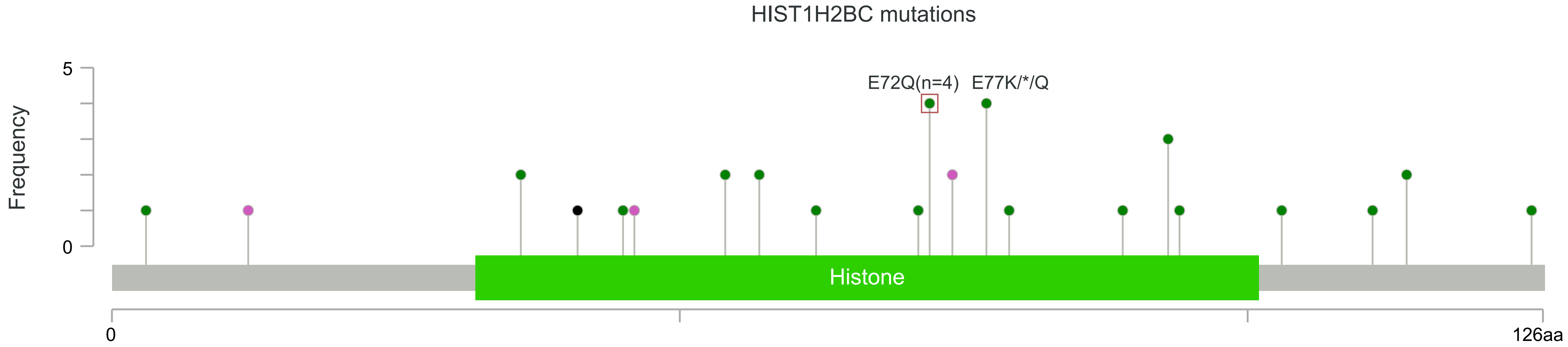
HIST1H2BC mutations
Frequency
5
0
E72Q(n=4)
E77K/*/Q
Histone
0
126aa

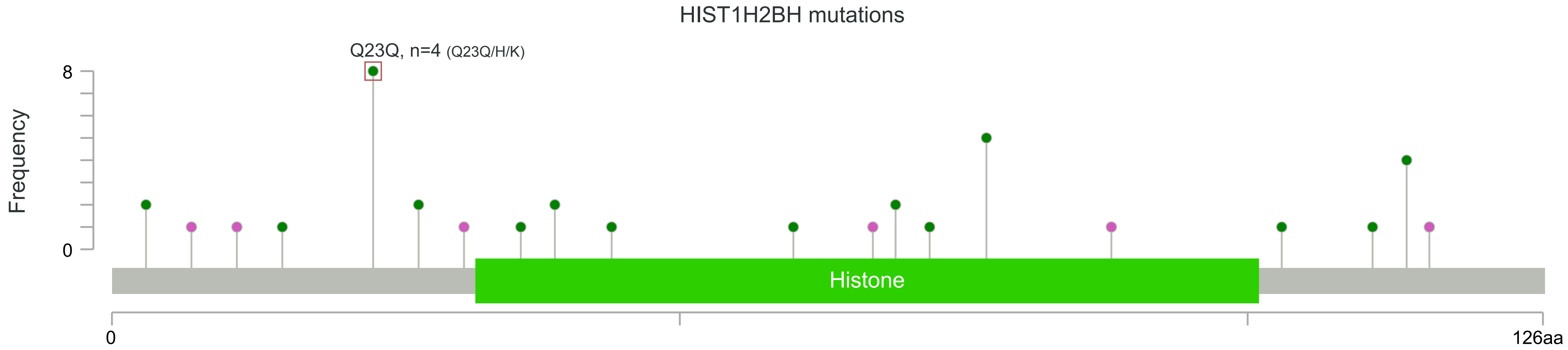

HIST1H2BH mutations
Q23Q, n=4 (Q23Q/H/K)
8
Frequency
0
Histone
0
126aa

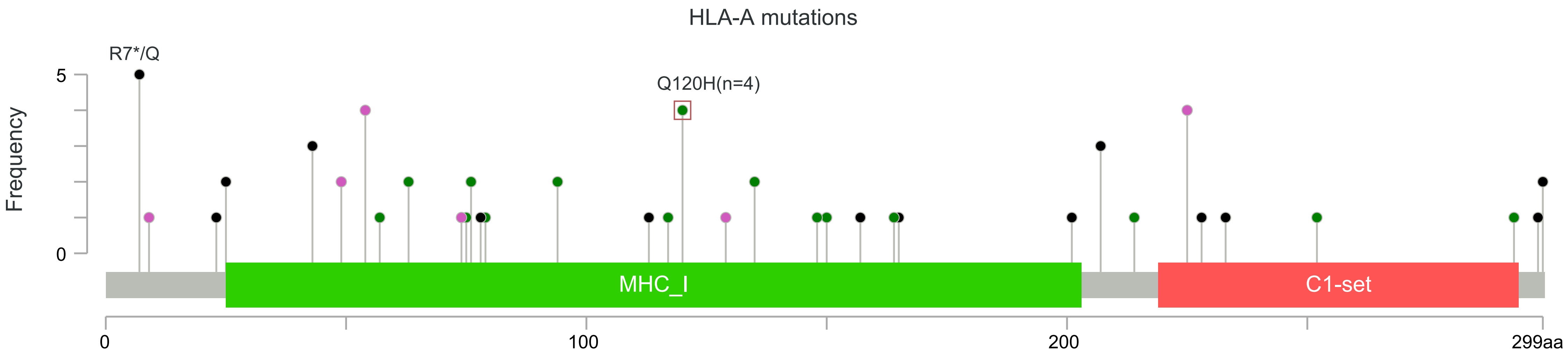
HLA-A mutations
R7*/Q
Q120H(n=4)
Frequency
5
0
MHC_I
C1-set
0
100
200
299aa

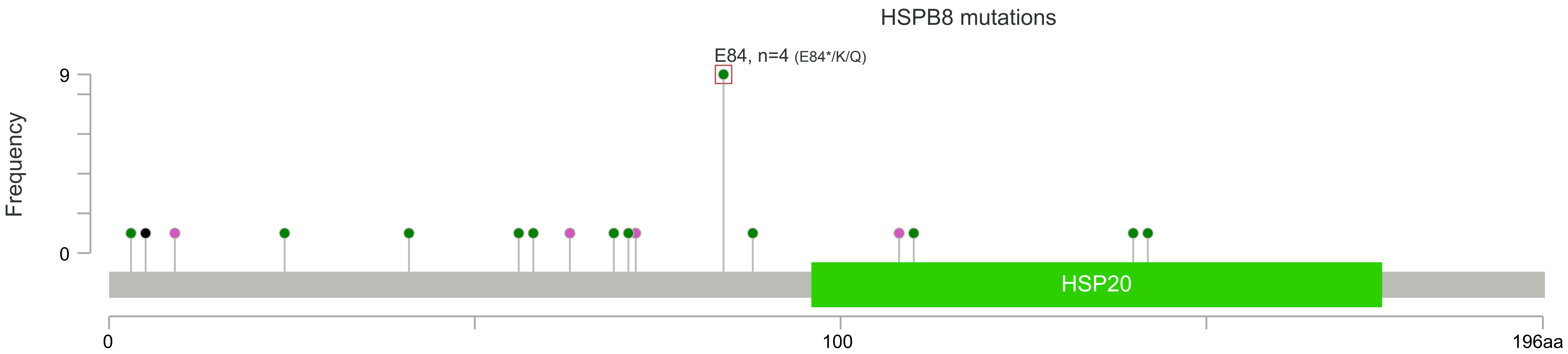
HSPB8 mutations
E84, n=4 (E84*/K/Q)
Frequency
9
0
HSP20
0
100
196aa

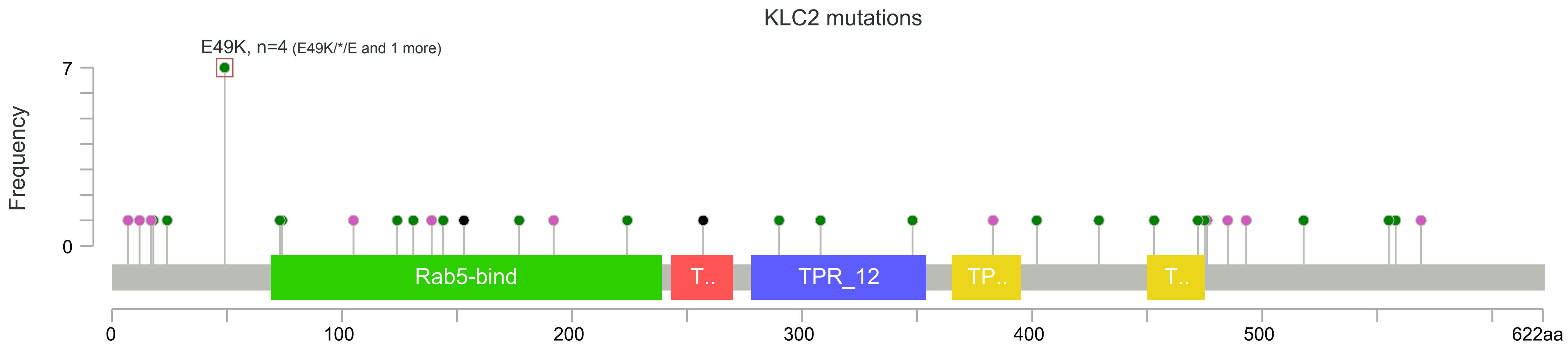

KLC2 mutations
E49K, n=4 (E49K/*/E and 1 more)
7
0
Frequency
Rab5-bind
T..
TPR_12
TP..
T..
0
100
200
300
400
500
622aa

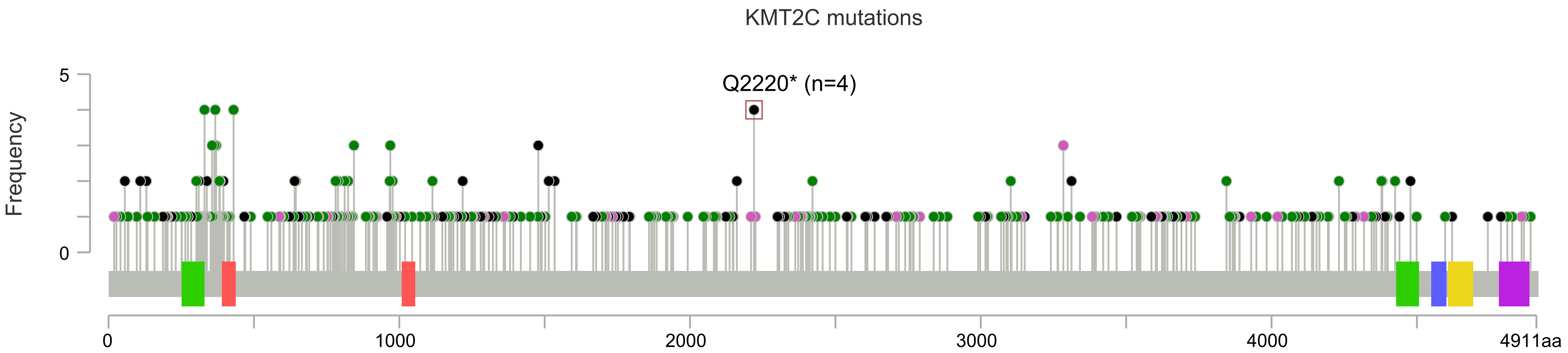

KMT2C mutations
Q2220* (n=4)
Frequency
5
0
0
1000
2000
3000
4000
4911aa

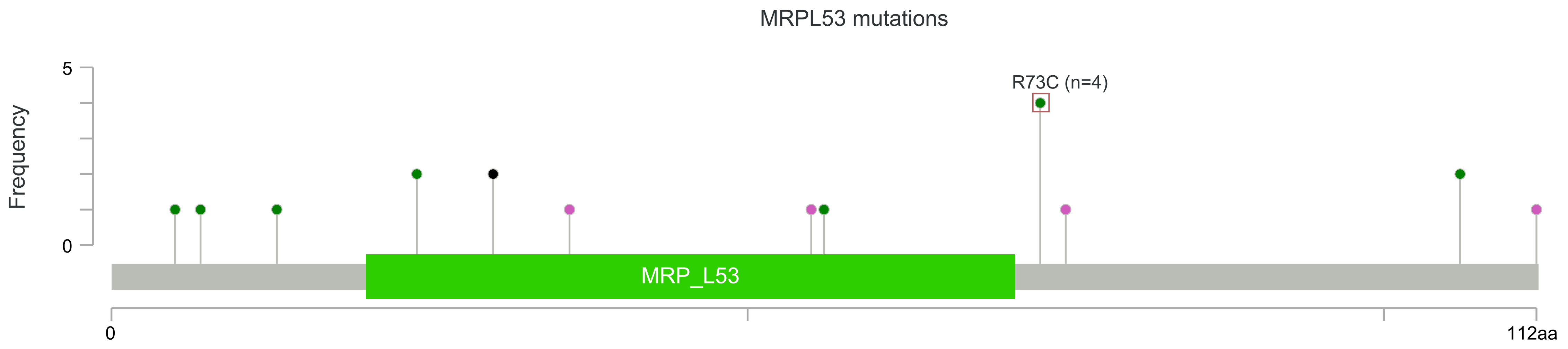

MRPL53 mutations
5
0
Frequency
R73C (n=4)
MRP_L53
0
112aa

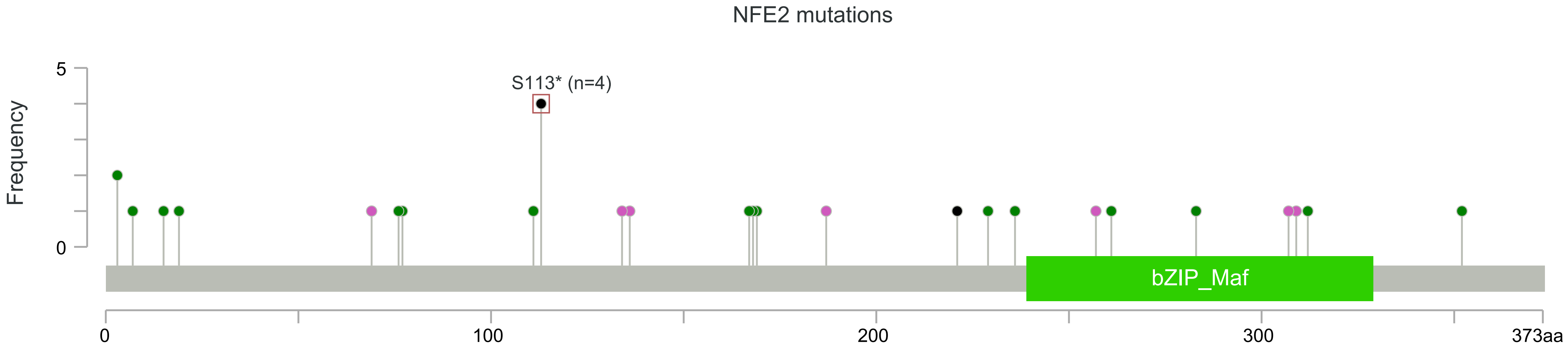
NFE2 mutations
Frequency
5
0
S113* (n=4)
bZIP_Maf
0
100
200
300
373aa

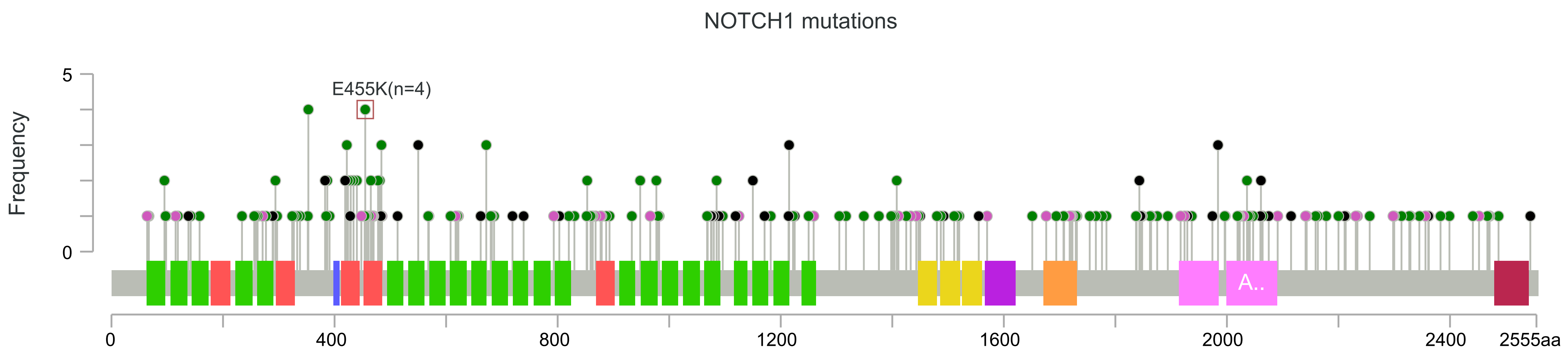

NOTCH1 mutations
Frequency
5
0
E455K(n=4)
A..
0
400
800
1200
1600
2000
2400
2555aa

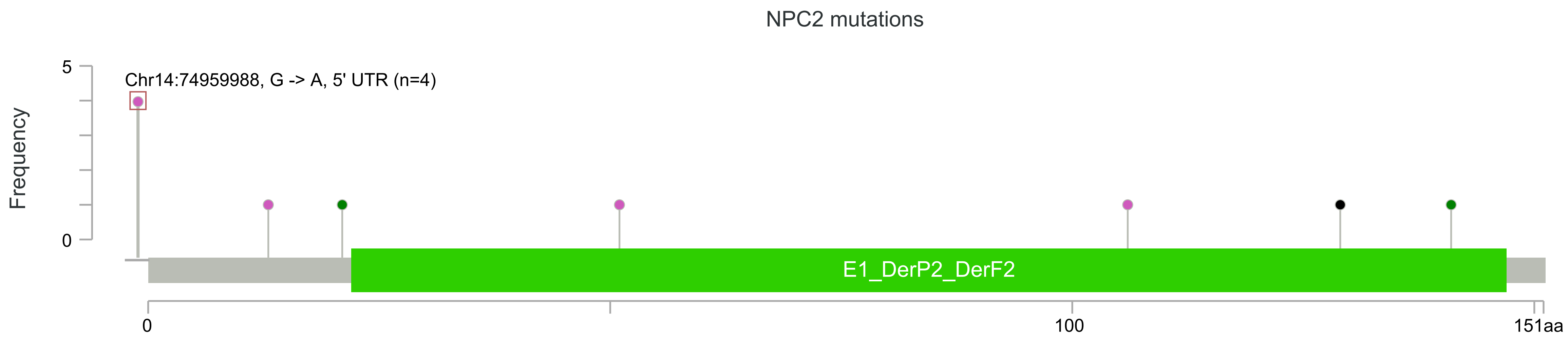

NPC2 mutations
5
0
Frequency
Chr14:74959988, G -> A, 5' UTR (n=4)
E1_DerP2_DerF2
0
100
151aa

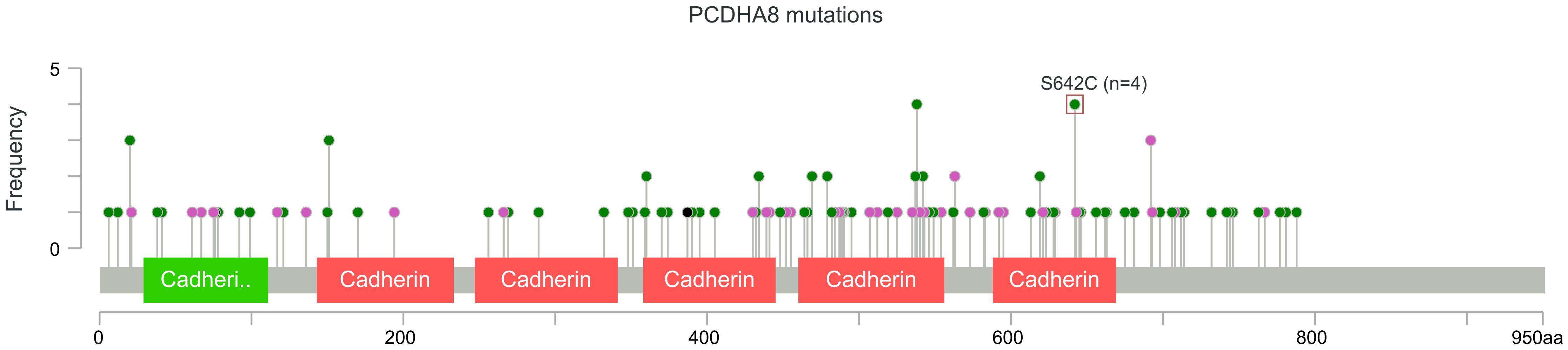
PCDHA8 mutations
Frequency
5
0
S642C (n=4)
Cadheri..
Cadherin
Cadherin
Cadherin
Cadherin
Cadherin
0
200
400
600
800
950aa

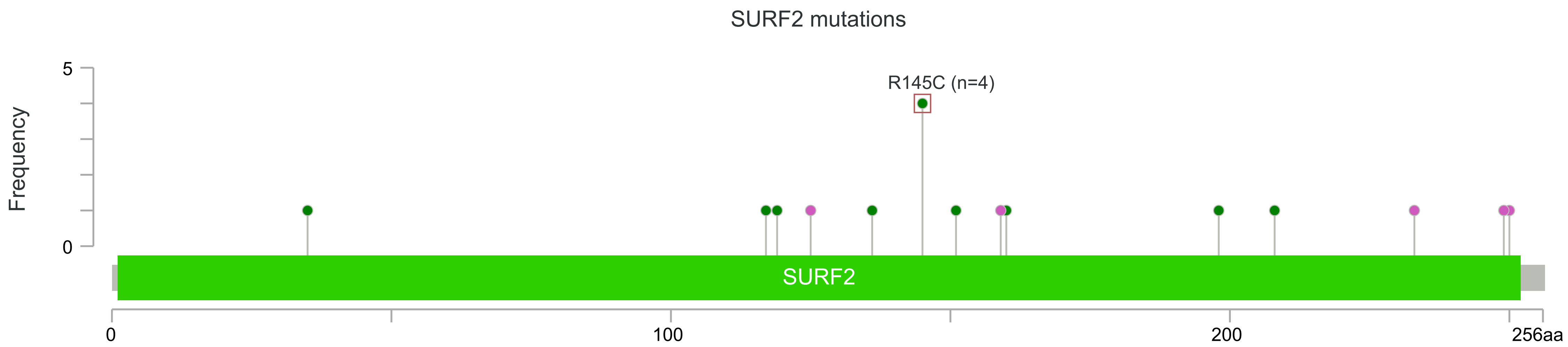
SURF2 mutations
Frequency
5
0
R145C (n=4)
SURF2
0
100
200
256aa

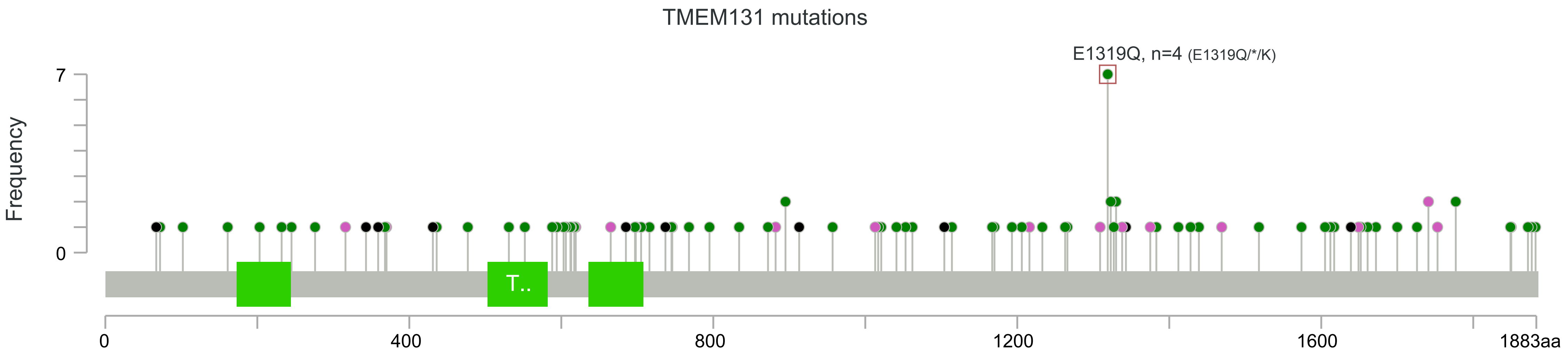

TMEM131 mutations
E1319Q, n=4 (E1319Q/*/K)
Frequency
7
0
T..
0
400
800
1200
1600
1883aa

Fig.S8

A

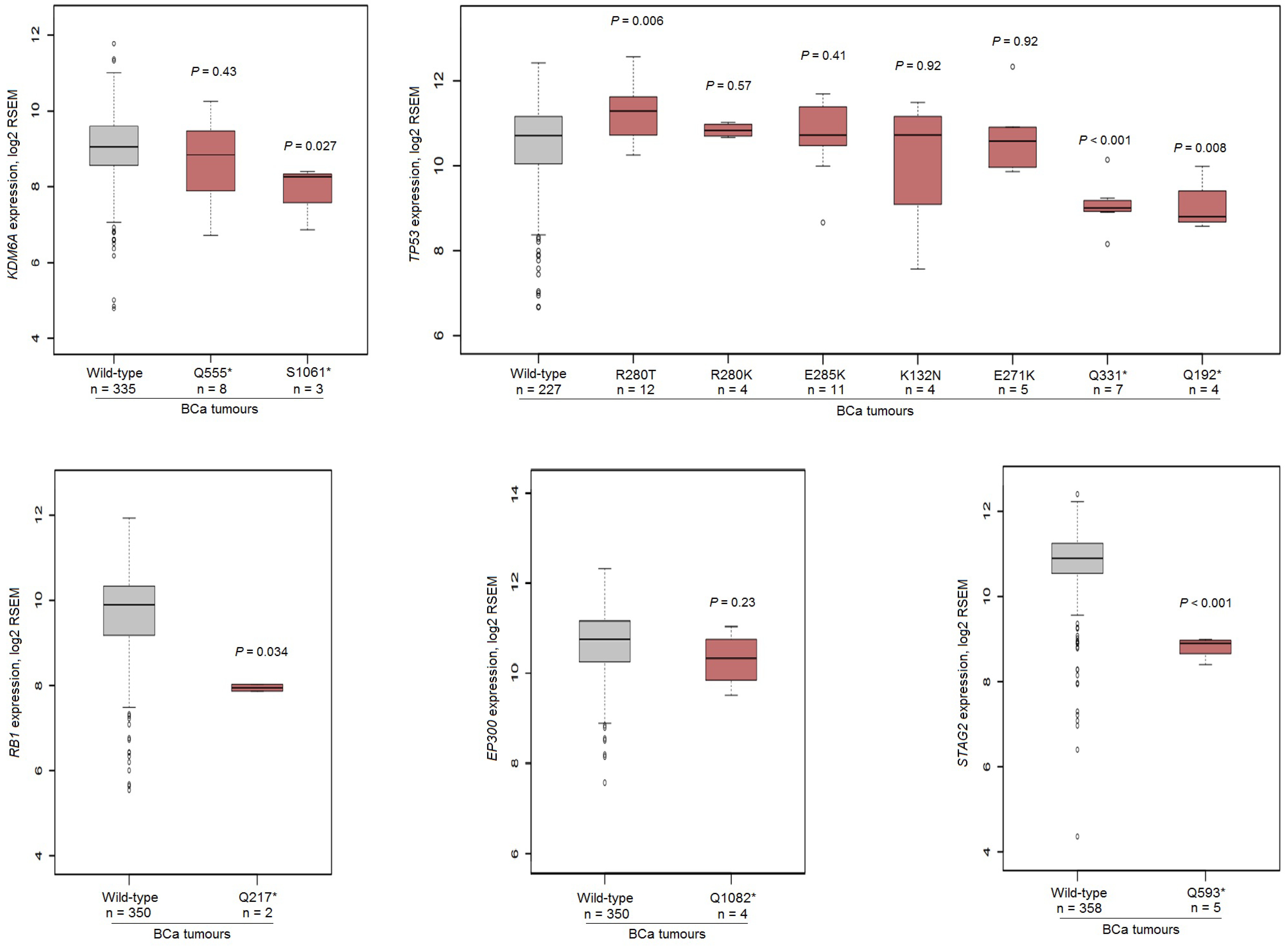
KDM6A expression, log2 RSEM
4
6
8
10
12
P = 0.43
P = 0.027
Wild-type
n = 335
Q555*
n = 8
S1061*
n = 3
BCa tumours
TP53 expression, log2 RSEM
6
8
10
12
P = 0.006
P = 0.57
P = 0.41
P = 0.92
P = 0.92
P < 0.001
P = 0.008
Wild-type
n = 227
R280T
n = 12
R280K
n = 4
E285K
n = 11
K132N
n = 4
E271K
n = 5
Q331*
n = 7
Q192*
n = 4
BCa tumours
RB1 expression, log2 RSEM
4
6
8
10
12
P = 0.034
Wild-type
n = 350
Q217*
n = 2
BCa tumours
EP300 expression, log2 RSEM
6
8
10
12
14
P = 0.23
Wild-type
n = 350
Q1082*
n = 4
BCa tumours
STAG2 expression, log2 RSEM
4
6
8
10
12
P < 0.001
Wild-type
n = 358
Q593*
n = 5
BCa tumours

B

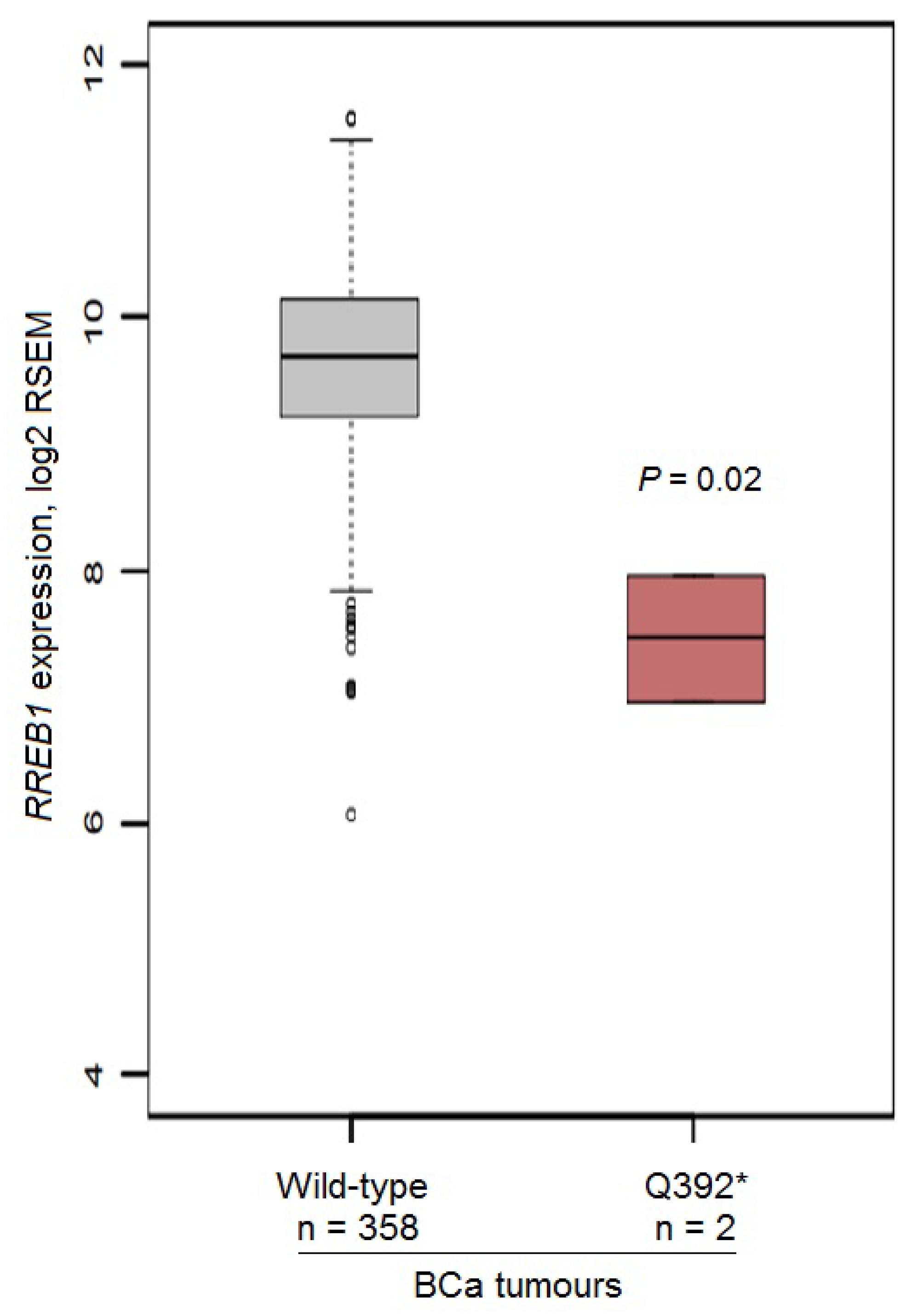
RREB1 expression, log2 RSEM
4
6
8
10
12
P = 0.02
Wild-type
n = 358
Q392*
n = 2
BCa tumours

Fig.S9

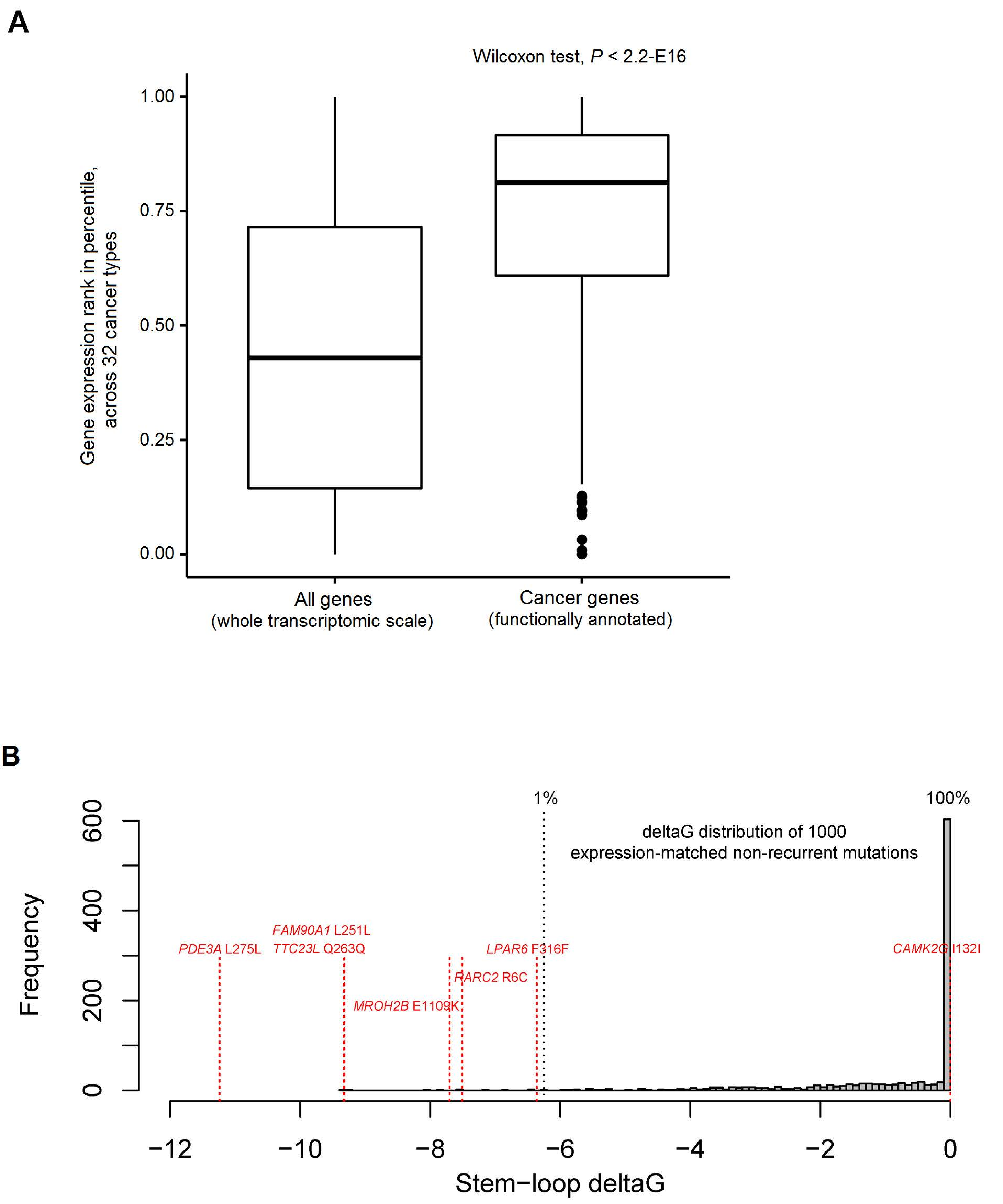
A
Wilcoxon test, P < 2.2-E16
Gene expression rank in percentile, across 32 cancer types
1.00
0.75
0.50
0.25
0.00
All genes
(whole transcriptomic scale)
Cancer genes
(functionally annotated)
B
1%
100%
deltaG distribution of 1000
expression-matched non-recurrent mutations
Frequency
600
400
200
0
PDE3A L275L
FAM90A1 L251L
TTC23L Q263Q
MROH2B E1109K
RARC2 R6C
LPAR6 F316F
CAMK2G I132I
−12
−10
−8
−6
−4
−2
0
Stem−loop deltaG

Fig. S10

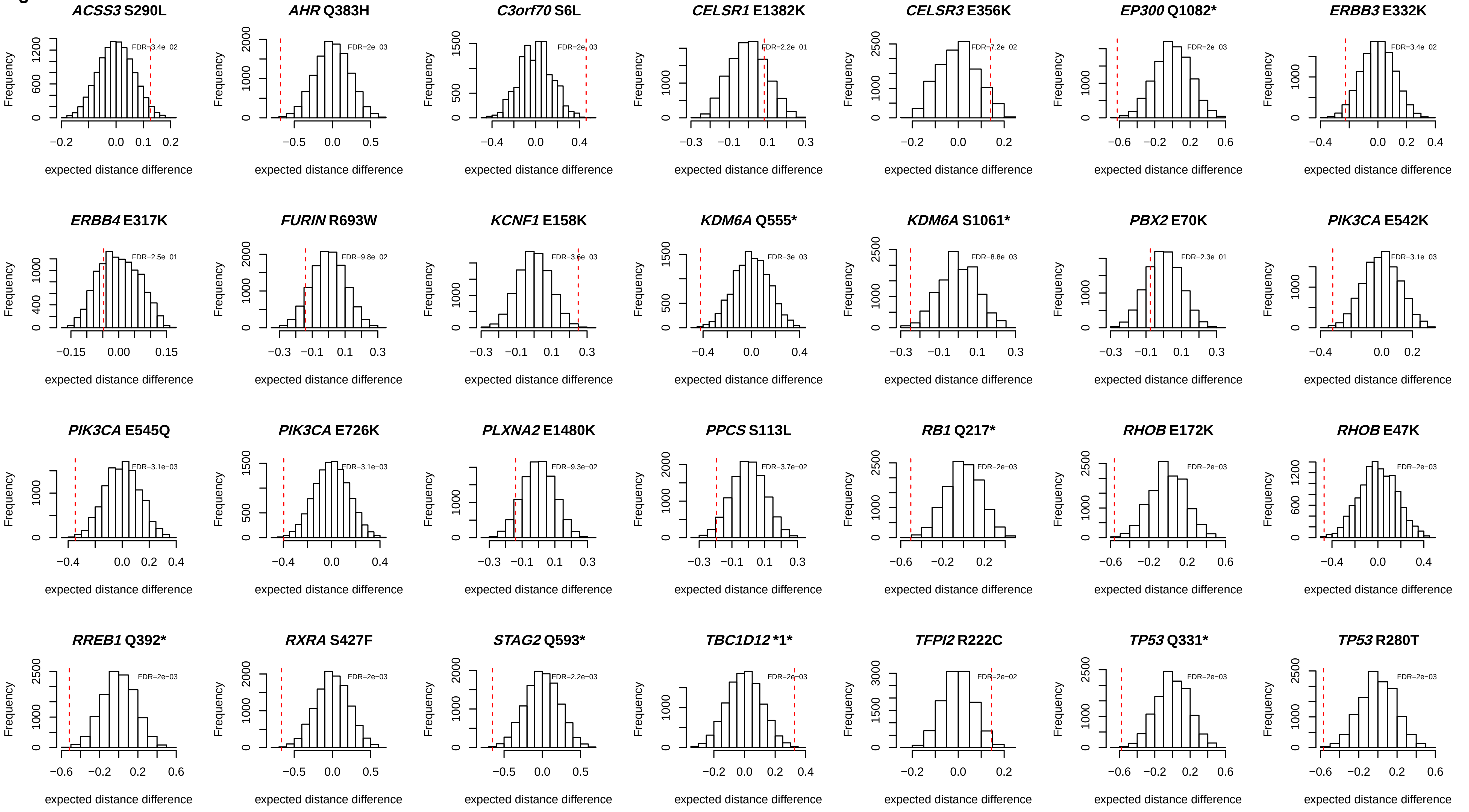
ACSS3 S290L
FDR=3.4e-02
AHR Q383H
FDR=2e-03
C3orf70 S6L
FDR=2e-03
CELSR1 E1382K
FDR=2.2e-01
CELSR3 E356K
FDR=7.2e-02
EP300 Q1082*
FDR=2e-03
ERBB3 E332K
FDR=3.4e-02
ERBB4 E317K
FDR=2.5e-01
FURIN R693W
FDR=9.8e-02
KCNF1 E158K
FDR=3.6e-03
KDM6A Q555*
FDR=3e-03
KDM6A S1061*
FDR=8.8e-03
PBX2 E70K
FDR=2.3e-01
PIK3CA E542K
FDR=3.1e-03
PIK3CA E545Q
FDR=3.1e-03
PIK3CA E726K
FDR=3.1e-03
PLXNA2 E1480K
FDR=9.3e-02
PPCS S113L
FDR=3.7e-02
RB1 Q217*
FDR=2e-03
RHOB E172K
FDR=2e-03
RHOB E47K
FDR=2e-03
RREB1 Q392*
FDR=2e-03
RXRA S427F
FDR=2e-03
STAG2 Q593*
FDR=2.2e-03
TBC1D12 *1*
FDR=2e-03
TFPI2 R222C
FDR=2e-02
TP53 Q331*
FDR=2e-03
TP53 R280T
FDR=2e-03
Frequency
expected distance difference

Fig. S11

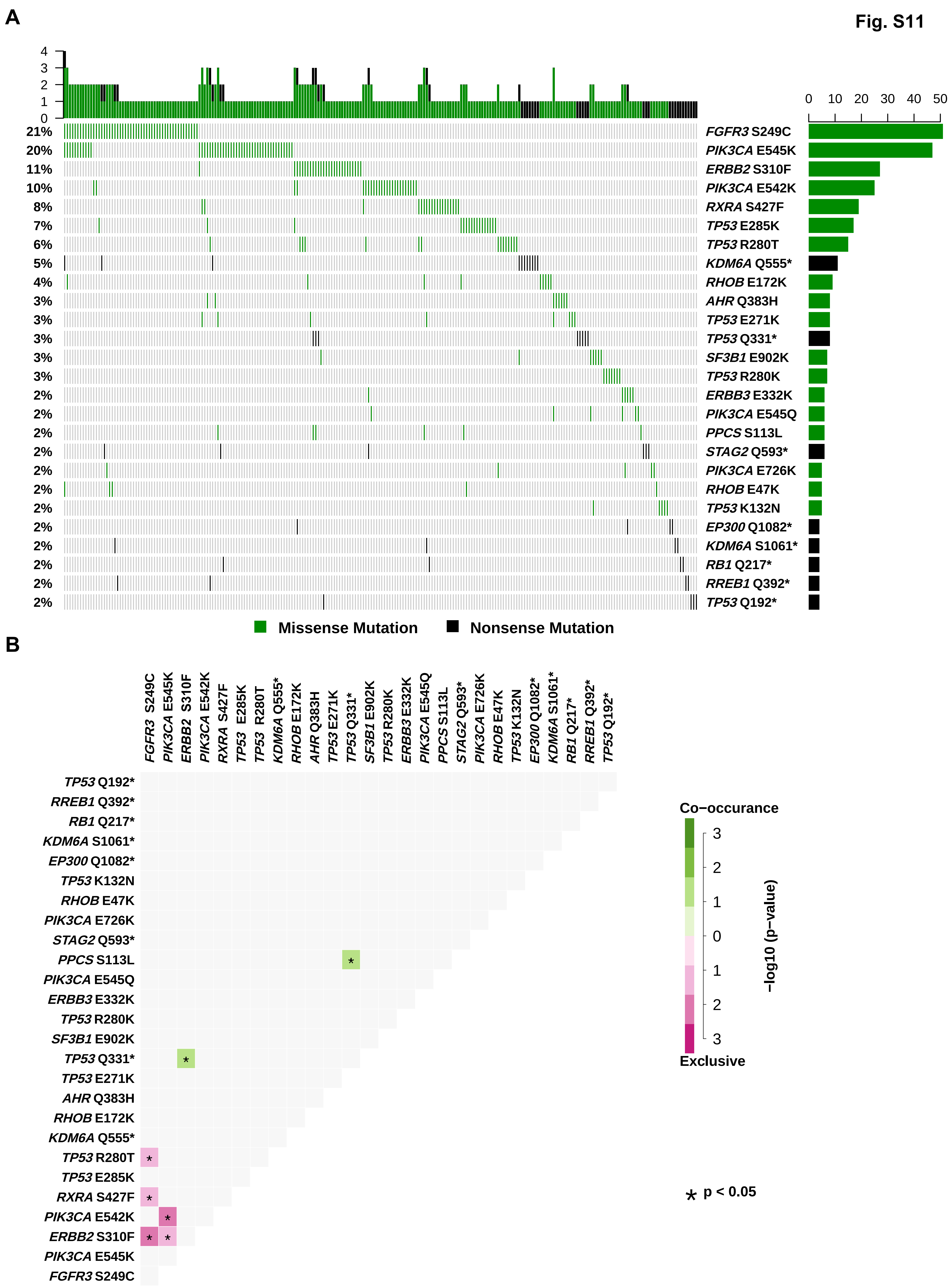
A
21%
20%
11%
10%
8%
7%
6%
5%
4%
3%
3%
3%
3%
3%
2%
2%
2%
2%
2%
2%
2%
2%
2%
2%
2%
2%
FGFR3 S249C
PIK3CA E545K
ERBB2 S310F
PIK3CA E542K
RXRA S427F
TP53 E285K
TP53 R280T
KDM6A Q555*
RHOB E172K
AHR Q383H
TP53 E271K
TP53 Q331*
SF3B1 E902K
TP53 R280K
ERBB3 E332K
PIK3CA E545Q
PPCS S113L
STAG2 Q593*
PIK3CA E726K
RHOB E47K
TP53 K132N
EP300 Q1082*
KDM6A S1061*
RB1 Q217*
RREB1 Q392*
TP53 Q192*
Missense Mutation
Nonsense Mutation
B
Co−occurance
Exclusive
−log10 (p−value)
* p < 0.05

Fig. S12

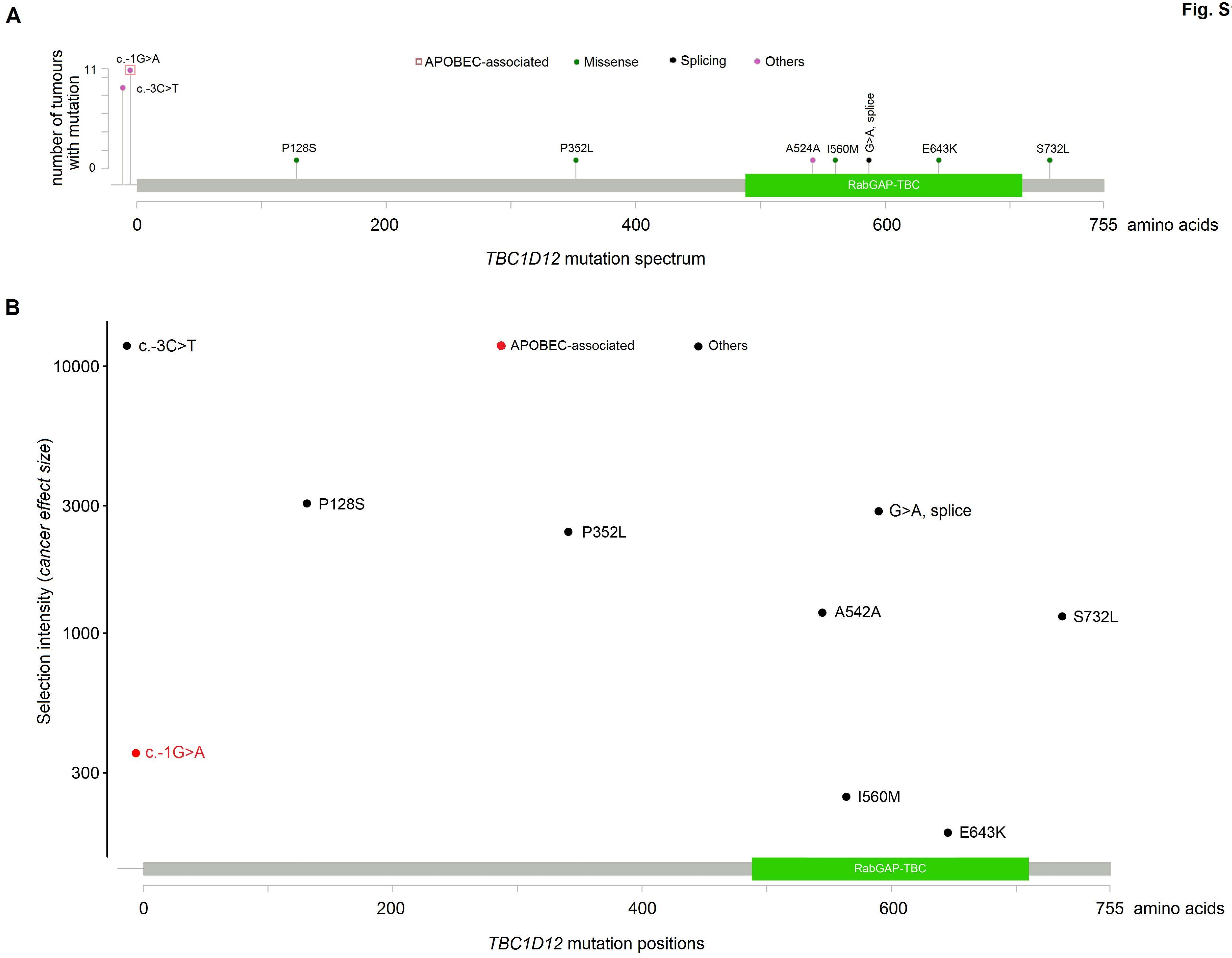
A
number of tumours with mutation
11
0
c.-1G>A
c.-3C>T
APOBEC-associated
Missense
Splicing
Others
P128S
P352L
A524A
I560M
G>A, splice
E643K
S732L
RabGAP-TBC
0
200
400
600
755 amino acids
TBC1D12 mutation spectrum
B
Selection intensity (cancer effect size)
10000
3000
1000
300
c.-3C>T
APOBEC-associated
Others
P128S
G>A, splice
P352L
A542A
S732L
c.-1G>A
I560M
E643K
RabGAP-TBC
0
200
400
600
755 amino acids
TBC1D12 mutation positions

Fig. S13

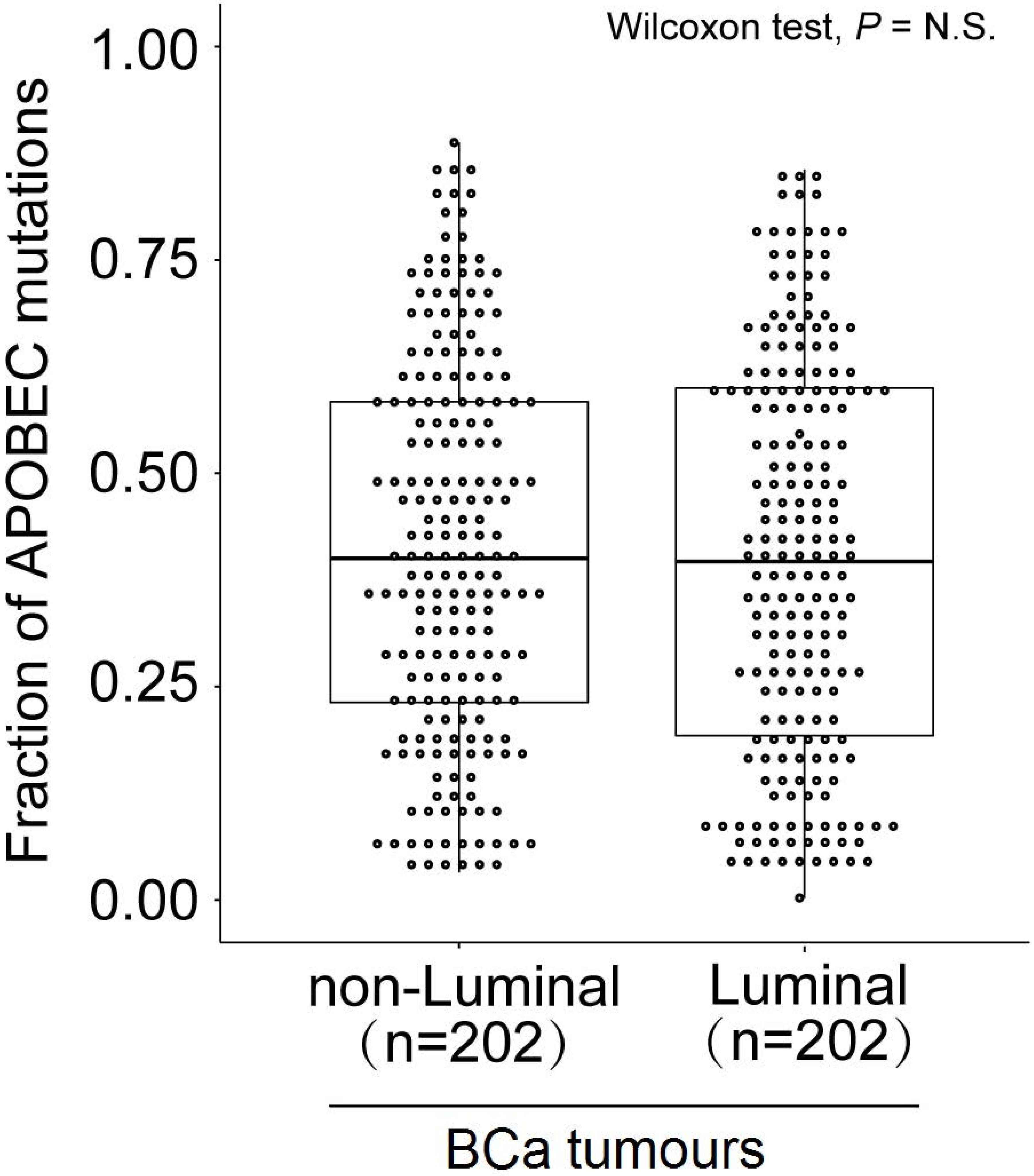

Wilcoxon test, P = N.S.
Fraction of APOBEC mutations
1.00
0.75
0.50
0.25
0.00
non-Luminal (n=202)
Luminal (n=202)
BCa tumours

Fig S14

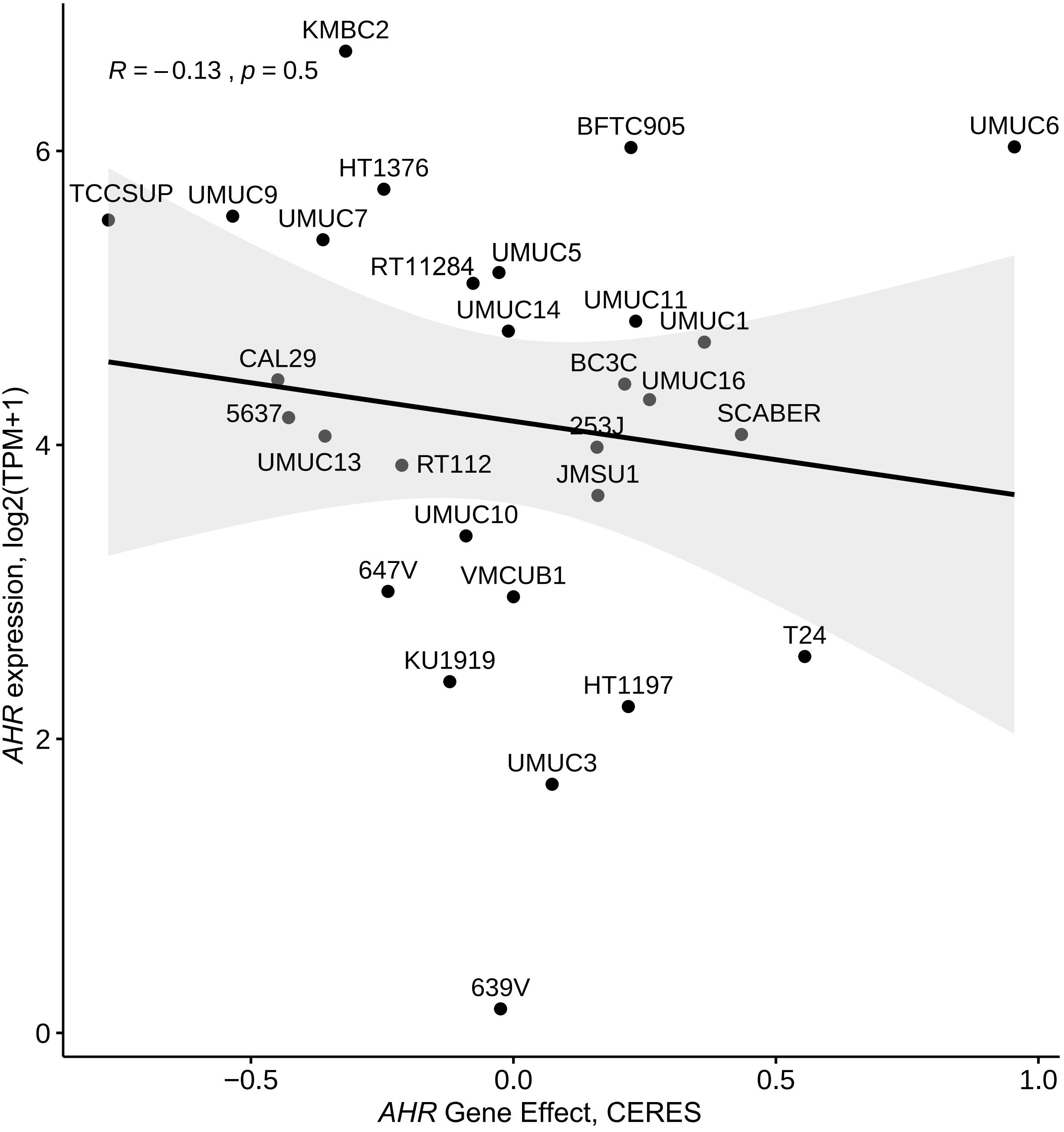

R = −0.13 , p = 0.5
KMBC2
BFTC905
UMUC6
HT1376
TCCSUP
UMUC9
UMUC7
UMUC5
RT11284
UMUC11
UMUC14
UMUC1
CAL29
BC3C
UMUC16
5637
SCABER
253J
UMUC13
RT112
JMSU1
UMUC10
647V
VMCUB1
T24
KU1919
HT1197
UMUC3
639V
0
2
4
6
−0.5
0.0
0.5
1.0
AHR expression, log2(TPM+1)
AHR Gene Effect, CERES

Fig S15

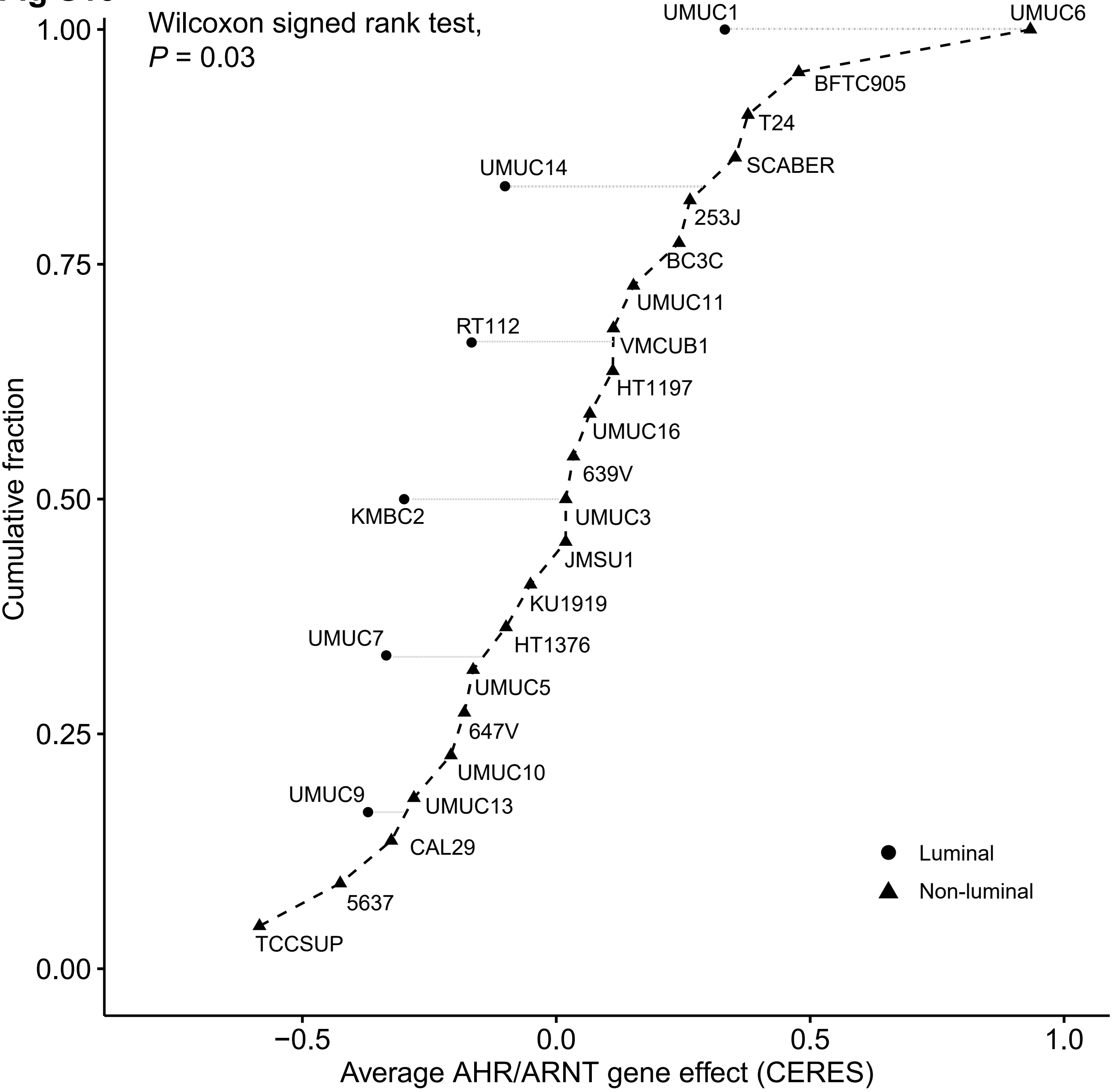
Wilcoxon signed rank test,
P = 0.03
Cumulative fraction
Average AHR/ARNT gene effect (CERES)
1.00
0.75
0.50
0.25
0.00
−0.5
0.0
0.5
1.0
UMUC1
UMUC6
BFTC905
T24
SCABER
UMUC14
253J
BC3C
UMUC11
RT112
VMCUB1
HT1197
UMUC16
639V
KMBC2
UMUC3
JMSU1
KU1919
UMUC7
HT1376
UMUC5
647V
UMUC10
UMUC9
UMUC13
CAL29
5637
TCCSUP
Luminal
Non-luminal

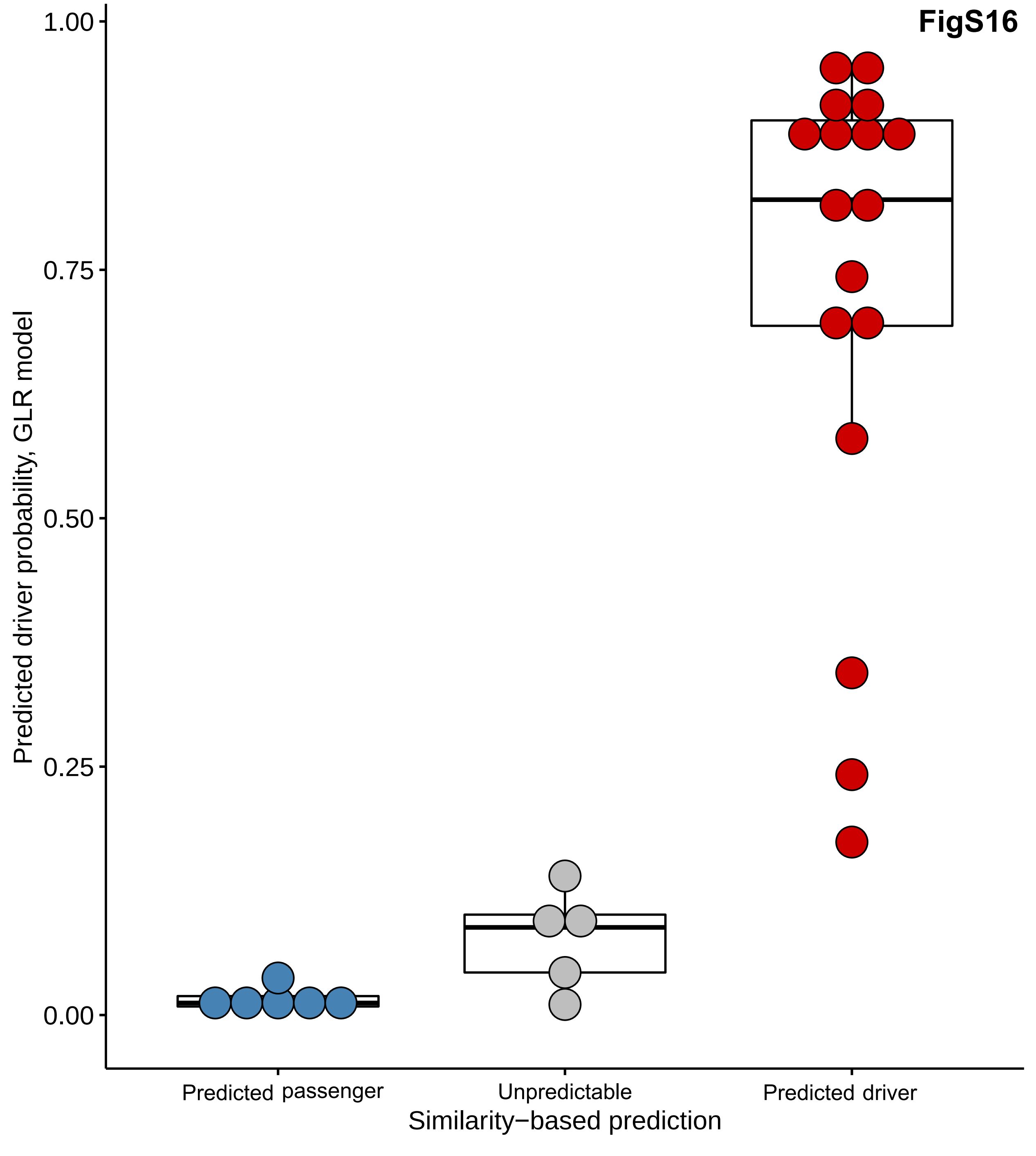
FigS16
Predicted driver probability, GLR model
1.00
0.75
0.50
0.25
0.00
Predicted passenger
Unpredictable
Predicted driver
Similarity−based prediction
